# Inhaled budesonide for COVID-19 in people at high risk of complications in the community in the UK (PRINCIPLE): a randomised, controlled, open-label, adaptive platform trial

**DOI:** 10.1016/S0140-6736(21)01744-X

**Published:** 2021-09-04

**Authors:** Ly-Mee Yu, Mona Bafadhel, Jienchi Dorward, Gail Hayward, Benjamin R Saville, Oghenekome Gbinigie, Oliver Van Hecke, Emma Ogburn, Philip H Evans, Nicholas P B Thomas, Mahendra G Patel, Duncan Richards, Nicholas Berry, Michelle A Detry, Christina Saunders, Mark Fitzgerald, Victoria Harris, Milensu Shanyinde, Simon de Lusignan, Monique I Andersson, Peter J Barnes, Richard E K Russell, Dan V Nicolau, Sanjay Ramakrishnan, F D Richard Hobbs, Christopher C Butler, PRINCIPLE Trial Collaborative Group, Ly-Mee Yu, Mona Bafadhel, Jienchi Dorward, Gail Hayward, Benjamin R Saville, Oghenekome Gbinigie, Oliver van Hecke, Emma Ogburn, Philip H Evans, Nicholas PB Thomas, Mahendra G Patel, Duncan Richards, Nicholas Berry, Michelle A Detry, Christina T Saunders, Mark Fitzgerald, Victoria Harris, Milensu Shanyinde, Simon de Lusignan, Monique I Andersson, Peter J Barnes, Richard EK Russell, Dan V Nicolau, Sanjay Ramakrishnan, FD Richard Hobbs, Christopher C Butler

**Affiliations:** a Nuffield Department of Primary Care Health Sciences, University of Oxford, Oxford, UK; b Nuffield Department of Clinical Medicine, University of Oxford, Oxford, UK; c Oxford Clinical Trials Research Unit, Botnar Research Centre, University of Oxford, Oxford, UK; d Centre for the AIDS Programme of Research in South Africa, University of KwaZulu–Natal, Durban, South Africa; e Berry Consultants, Austin, TX, USA; f Department of Biostatistics, Vanderbilt University School of Medicine, Nashville, TN, USA; g College of Medicine and Health, University of Exeter, Exeter, UK; h National Institute for Health Research Clinical Research Network, National Institute for Health Research, London, UK; i Royal College of General Practitioners, London, UK; j National Heart and Lung Institute, Imperial College, London, UK; k UQ Centre for Clinical Research, University of Queensland, Brisbane, QLD, Australia; l National Institute for Health Research Oxford Biomedical Research Centre, Oxford, UK

## Abstract

**Background:**

A previous efficacy trial found benefit from inhaled budesonide for COVID-19 in patients not admitted to hospital, but effectiveness in high-risk individuals is unknown. We aimed to establish whether inhaled budesonide reduces time to recovery and COVID-19-related hospital admissions or deaths among people at high risk of complications in the community.

**Methods:**

PRINCIPLE is a multicentre, open-label, multi-arm, randomised, controlled, adaptive platform trial done remotely from a central trial site and at primary care centres in the UK. Eligible participants were aged 65 years or older or 50 years or older with comorbidities, and unwell for up to 14 days with suspected COVID-19 but not admitted to hospital. Participants were randomly assigned to usual care, usual care plus inhaled budesonide (800 μg twice daily for 14 days), or usual care plus other interventions, and followed up for 28 days. Participants were aware of group assignment. The coprimary endpoints are time to first self-reported recovery and hospital admission or death related to COVID-19, within 28 days, analysed using Bayesian models. The primary analysis population included all eligible SARS-CoV-2-positive participants randomly assigned to budesonide, usual care, and other interventions, from the start of the platform trial until the budesonide group was closed. This trial is registered at the ISRCTN registry (ISRCTN86534580) and is ongoing.

**Findings:**

The trial began enrolment on April 2, 2020, with randomisation to budesonide from Nov 27, 2020, until March 31, 2021, when the prespecified time to recovery superiority criterion was met. 4700 participants were randomly assigned to budesonide (n=1073), usual care alone (n=1988), or other treatments (n=1639). The primary analysis model includes 2530 SARS-CoV-2-positive participants, with 787 in the budesonide group, 1069 in the usual care group, and 974 receiving other treatments. There was a benefit in time to first self-reported recovery of an estimated 2·94 days (95% Bayesian credible interval [BCI] 1·19 to 5·12) in the budesonide group versus the usual care group (11·8 days [95% BCI 10·0 to 14·1] *vs* 14·7 days [12·3 to 18·0]; hazard ratio 1·21 [95% BCI 1·08 to 1·36]), with a probability of superiority greater than 0·999, meeting the prespecified superiority threshold of 0·99. For the hospital admission or death outcome, the estimated rate was 6·8% (95% BCI 4·1 to 10·2) in the budesonide group versus 8·8% (5·5 to 12·7) in the usual care group (estimated absolute difference 2·0% [95% BCI –0·2 to 4·5]; odds ratio 0·75 [95% BCI 0·55 to 1·03]), with a probability of superiority 0·963, below the prespecified superiority threshold of 0·975. Two participants in the budesonide group and four in the usual care group had serious adverse events (hospital admissions unrelated to COVID-19).

**Interpretation:**

Inhaled budesonide improves time to recovery, with a chance of also reducing hospital admissions or deaths (although our results did not meet the superiority threshold), in people with COVID-19 in the community who are at higher risk of complications.

**Funding:**

National Institute of Health Research and United Kingdom Research Innovation.

## Introduction

There is an urgent need for effective and safe community-based treatments for COVID-19, especially for older people and those with comorbidities who are at higher risk of hospital admission and death.^1^

Inhaled corticosteroids are widely available, inexpensive, and generally safe, and have been proposed as a COVID-19 treatment because of their targeted anti-inflammatory effects in the lungs,^2^, ^3^ where they also reduce expression of ACE-2 and TMPRSS2,^4^, ^5^ which is relevant for airway epithelial cell entry by SARS-CoV-2.^6^ Inhaled steroids also reduce replication of SARS-CoV-2 in epithelial cells in vitro.^7^ Early in the COVID-19 pandemic, the low prevalence of asthma and chronic obstructive pulmonary disease among people admitted to hospital with COVID-19 led to speculation that the inhaled corticosteroids used to treat these conditions might be protective.^2^, ^3^ Furthermore, systemic corticosteroids reduce deaths in patients admitted to hospital with COVID-19,^8^, ^9^ probably because the hyperinflammatory state is responsible for the subsequent damage from SARS-CoV-2 infection.^10^ However, subgroup analyses in the RECOVERY trial suggested no benefit, and possible harm, with use of systemic corticosteroids in patients admitted to hospital not requiring oxygen.^8^ In addition, observational, population-based studies in primary care in the UK found an increased risk of COVID-19 hospital admission or death among people prescribed inhaled corticosteroids for chronic lung disease,^11^, ^12^ although residual confounding by unmeasured disease severity could not be ruled out. An efficacy trial of adults with early COVID-19 in the community found inhaled budesonide reduced COVID-19-related emergency assessments or hospital admissions, and time to self-reported recovery.^13^ However, thus far, there are no results reported from large effectiveness trials of inhaled budesonide for COVID-19.

**Research in context**

**Evidence before this study**

We did a search of PubMed on June 24, 2021, using the following search terms “(randomised OR trial) AND (budesonide OR inhaled corticosteroids OR inhaled steroids) AND (COVID* OR SARS-CoV-2 OR SARS-CoV)”, with no date or language restrictions. The search identified 44 results, one of which reported findings from a randomised controlled trial. In the STOIC phase 2, open-label trial, among adults aged 18 years and older with early, suspected COVID-19 in the community, 146 participants were randomly assigned to inhaled budesonide 800 μg twice a day until symptoms resolved, or usual care. The primary outcome of COVID-19-related urgent care or emergency department assessment, or hospital admission, was achieved in one (1%) of 70 participants receiving budesonide versus ten (14%) of 69 receiving usual care (difference in proportions 0·131 [95% CI 0·043–0·218], p=0·004). Secondary outcomes in STOIC also favoured budesonide over usual care with respect to time to self-reported recovery, symptom persistence at day 14, and resolution of fever. A search of ClinicalTrials.gov on June 24, 2021, using the terms “COVID-19” and “ciclesonide” OR “mometasone” OR “fluticasone” OR “beclometasone” OR “budesonide”, identified ten additional ongoing or completed randomised controlled trials assessing budesonide as treatment for COVID-19 studies, none of which had reported results.

**Added value of this study**

To our knowledge, PRINCIPLE is the first pragmatic randomised trial to report the effectiveness of an inhaled corticosteroid for people with COVID-19 in the community. We found that inhaled budesonide reduced time to recovery by 3 days, with a high probability of also reducing COVID-19-related hospital admissions or deaths by an absolute difference of 2%.

**Implications of all the available evidence**

PRINCIPLE is the first randomised trial to demonstrate effectiveness of inhaled budesonide to treat COVID-19 in the community, and builds on earlier evidence from the phase 2 STOIC trial. Reducing time to recovery is an important outcome for patients, whereas potential prevention of hospital admissions or deaths would lessen the burden on hospitals during COVID-19 surges. There was no evidence in the STOIC trial of a negative effect of budesonide on SARS-CoV-2 viral loads, and in PRINCIPLE there were no concerning safety signals for inhaled budesonide. Inhaled budesonide should be considered for patients with COVID-19 who are at higher risk of complications in the community.

We therefore aimed to establish the effectiveness of inhaled budesonide in reducing recovery time and rates of COVID-19-related hospital admission or death in people at high risk of an adverse outcome in the community.

## Methods

### Study design

PRINCIPLE is a multicentre, open-label, multi-arm, prospective, randomised, controlled, adaptive platform trial of interventions against COVID-19 in people aged 65 years or older or 50 years or older with comorbidities, done remotely from a central trial site and at primary care centres in the UK. The protocol is available in the appendix (pp 4–80) and on the trial website. A platform trial allows multiple treatments for the same disease to be assessed simultaneously. A master protocol defines prospective decision criteria for dropping interventions for futility, declaring interventions superior, or adding new interventions.^14^ The interventions assessed in PRINCIPLE were hydroxychloroquine, azithromycin,^15^ doxycycline,^16^ colchicine, favipiravir and, reported here, inhaled budesonide.

The UK Medicines and Healthcare products Regulatory Agency and the South Central-Berkshire Research Ethics Committee (ref 20/SC/0158) approved the trial protocol. Online consent was obtained from all participants before enrolment.

### Participants

People in the community were eligible if they were aged at least 65 years, or at least 50 years with comorbidities, and had ongoing symptoms from PCR-confirmed or suspected COVID-19 (in accordance with the UK National Health Service definition of high temperature, new, continuous cough, or change in sense of smell or taste),^17^, ^18^ which had started within the previous 14 days. Comorbidities required for eligibility in those aged 50–65 years were heart disease, hypertension, asthma or lung disease, diabetes, hepatic impairment, stroke or neurological problems, weakened immune system (eg, receiving chemotherapy), and self-reported obesity or body-mass index of at least 35 kg/m^2^. People were ineligible to be assigned to budesonide if they were already taking inhaled or systemic corticosteroids, were unable to use an inhaler, or if inhaled budesonide was contraindicated according to the British National Formulary. Initially, eligible people were recruited, screened, and enrolled through participating general medical practices, but from May 17, 2020, people across the UK could enrol online or by telephone. After patients completed a baseline and screening questionnaire, a clinician or trained research nurse confirmed eligibility using the patient's primary care medical record, accessed remotely where necessary, before conducting randomisation. To increase recruitment from ethnic minority and socially deprived communities, which have been disproportionally affected by COVID-19, we used several outreach strategies, including the appointment in September, 2020, of an expert working with ethnic minority groups; active collaborations with community, religious, and health organisations; and promotion in multiple languages through a range of media.^19^

### Randomisation and masking

Eligible, consenting participants were randomly assigned using a secure, in-house, web-based randomisation system (Sortition version 2.3) to budesonide, usual care, or other treatments. Randomisation probabilities were established using response-adaptive randomisation via regular interim analyses, which allows allocation of more participants to interventions with better observed time-to-recovery outcomes (appendix pp 140–142). Between Dec 14, 2020, and March 4, 2021, when only the budesonide and usual care groups were open, there was 1:1 allocation between each, stratified by age (<65 years *vs* ≥65 years), and presence of comorbidity (yes *vs* no). The trial team was masked to randomisation probabilities, but all participants were aware of group assignment.

### Procedures

Participants received usual care plus inhaled budesonide 800 μg twice daily for 14 days (Pulmicort Turbohaler, AstraZeneca, Luton, UK), or usual care alone. The breath-actuated inhaler was chosen because of its ease of use, and was either prescribed or issued directly by the participant's general medical practitioner, or issued centrally by the study team and delivered to the participant by urgent courier. Participants in the budesonide group were sent a video link demonstrating inhaler use, with additional telephone support where necessary. Usual care in the UK National Health Service for suspected COVID-19 in the community is largely focused on managing symptoms with antipyretics, with antibiotics only recommended if bacterial pneumonia is suspected.^20^, ^21^

Participants were followed up through an online, daily symptom diary for 28 days after randomisation, supplemented with telephone calls to non-responders on days 7, 14, and 28. The diary includes questions about illness recovery (ascertained by answering the question, “Do you feel recovered today? (ie, symptoms associated with illness are no longer a problem) yes *vs* no”), overall illness severity (a rating of how well they are feeling on a scale of 1–10, 1 being the worst and 10 being the best), individual symptom severity on a four point scale (0 being no problem to 3 being major problem), and health-care service use. Participants could nominate a trial partner to help provide follow-up data. We obtained consent to ascertain health-care use outcome data from general practice and hospital records. We aimed to provide a self-swab for SARS-CoV-2 confirmatory PCR testing, but capacity issues early in the pandemic meant testing was unavailable for some participants.

### Outcomes

The trial commenced with the primary outcome of COVID-19-related hospital admission or death within 28 days. However, hospital admission rates in the UK^22^ were lower than initially expected.^23^ Therefore, the trial management group and trial steering committee recommended amending the primary outcome to also include illness duration,^24^, ^25^ which is an important outcome for patients and has substantial economic and social impacts. This received ethical approval on Sept 16, 2020, and was implemented before performing any interim analyses. Thus, the trial has two coprimary endpoints measured within 28 days of randomisation: time to first reported recovery defined as the first instance that a participant reports feeling recovered; and hospital admission or death related to COVID-19. Decisions about COVID-19 relatedness were made after independent review of available data by two clinicians masked to treatment allocation and study identifiers.

Secondary outcomes (defined in section 3.3 of the master statistical analysis plan; appendix pp 102–109) include a binary outcome of early, sustained recovery (recovered by day 14 and remains recovered until day 28), time to sustained recovery (date participant first reports recovery and subsequently remains well until 28 days), daily rating of 1–10 of how well participants feel, time to initial alleviation of symptoms (date symptoms first reported as minor or none), time to sustained alleviation of symptoms (date symptoms first reported as minor or none and subsequently remain minor or none until 28 days), time to initial reduction of severity of symptoms (date symptom severity reported at least one grade lower), contacts with health services, hospital assessment without admission, oxygen administration, intensive care unit admission, mechanical ventilation, adherence to study treatment, WHO-5 Well-Being Index,^26^ and reports of new household infections. All time-to-event analyses used date of randomisation as baseline. We included secondary outcomes that capture sustained recovery because of the often recurrent and relapsing nature of COVID-19 symptoms. Serious adverse events other than the coprimary outcome of hospital admission or death related to COVID-19 were measured in all trial groups.

### Statistical analysis

Sample size calculation and statistical analysis are detailed in the adaptive design report (appendix pp 130–230) and the master statistical analysis plan (appendix pp 81–129). In the adaptive design report, we justify sample sizes by simulating the operating characteristics of the adaptive design in multiple scenarios, which explicitly account for response-adaptive randomisation, early stopping for futility or success, and multiple interventions. Briefly, for the primary outcome analyses, assuming a median time to recovery of 9 days in the usual care group, about 400 participants per group would provide 90% power to detect a 2-day difference in median recovery time. Assuming 5% hospital admission in the usual care group, about 1500 participants per group would provide 90% power to detect a 50% reduction in the relative risk of hospital admission or death.

The first coprimary outcome, time to first self-reported recovery, was analysed using a Bayesian piecewise exponential model. The second coprimary outcome, hospital admission or death, was analysed using a Bayesian logistic regression model. Both models were regressed on treatment group and stratification covariates (age <65 years *vs* ≥65 years and comorbidity yes *vs* no). These primary outcomes were assessed using a gate-keeping strategy to preserve the overall type I error without additional adjustments for multiple hypotheses. The hypothesis for the time to first recovery endpoint was assessed first, and if the null hypothesis was rejected, the hypothesis for the second coprimary endpoint of hospital admission or death was assessed. In the context of multiple interim analyses, the master protocol specifies that each null hypothesis is rejected if the Bayesian posterior probability of superiority exceeded 0**·**99 for the time to recovery endpoint and 0·975 (via gate keeping) for the hospital admission or death endpoint.

At the beginning of the trial, because of initial difficulties with community SARS-CoV-2 PCR testing in the UK, participants with suspected COVID-19 were included in the primary analysis population, irrespective of confirmatory testing. When testing became more accessible, the trial steering committee recommended restricting the primary analysis population to those with confirmed COVID-19. This change was included in protocol, version 7.1, on Feb 22, 2021, and approved on March 15, 2021, before any interim budesonide results were disclosed to the trial management group. Therefore, the prespecified primary analysis population includes all eligible SARS-CoV-2-positive participants randomly assigned to budesonide, usual care, and other interventions, from the start of the platform trial until the budesonide group was closed, on March 31, 2021. This population includes participants randomly assigned to usual care before the budesonide group opened, who might differ from concurrently assigned participants because of changes in the inclusion or exclusion criteria (eg, current inhaled corticosteroid use was only added as an exclusion criterion when the budesonide group opened), and changes over time in circulating SARS-CoV-2 or usual care, including increasing availability of vaccinations. Therefore, the primary analysis models include parameters to adjust for this temporal drift in the trial population, by estimating the primary endpoint in the usual care group across time via Bayesian hierarchical modelling. We also accounted for potential temporal drift by conducting a prespecified sensitivity analysis of the primary outcomes using the concurrent assigned population; defined as all SARS-CoV-2-positive participants randomly assigned during the time period when the budesonide group was active. To establish the applicability of our results to situations where PCR testing might not be readily available, we also did secondary analyses of time to recovery and COVID-19-related hospital admission or death among the overall study population, irrespective of SARS-CoV-2 status.

Analysis of all secondary outcomes and prespecified subgroup analyses were done in SARS-CoV-2-positive participants eligible for budesonide and concurrently assigned to budesonide or usual care—the concurrent randomisation and eligible SARS-CoV-2-positive population. Secondary time-to-event outcomes were analysed using Cox proportional hazard models, and binary outcomes were analysed using logistic regression, adjusting for comorbidity, age, duration of illness, and vaccination status. Because of the high proportion of participants contributing to the analysis of primary outcomes (95·3%), we did not explore the potential impact of missing data. All model assumptions were checked and validated.

The primary outcomes were assessed in all participants, excluding those who were ineligible, withdrew consent, had no diary information, or who recovered on day 0. Analyses were done using R (version 3.6.0) and Stata (version 16.1). An independent trial steering committee and data monitoring and safety committee provided trial oversight. The trial is registered at the ISRCTN registry (ISRCTN86534580) and is ongoing.

### Role of the funding source

The funder had no role in study design, data collection, data analysis, data interpretation, or writing of the report.

## Results

The first participant was randomised into PRINCIPLE on April 2, 2020. Enrolment into the budesonide group started on Nov 27, 2020. On March 31, 2021, the trial steering committee advised the trial management group to stop randomisation to budesonide because the prespecified superiority criterion had been met on time to recovery, and accumulating enough data to reach futility or superiority criteria on hospital admission or death was unlikely because of decreases in hospital admissions associated with the UK lockdown and vaccination programme.^22^

**Figure 1:**
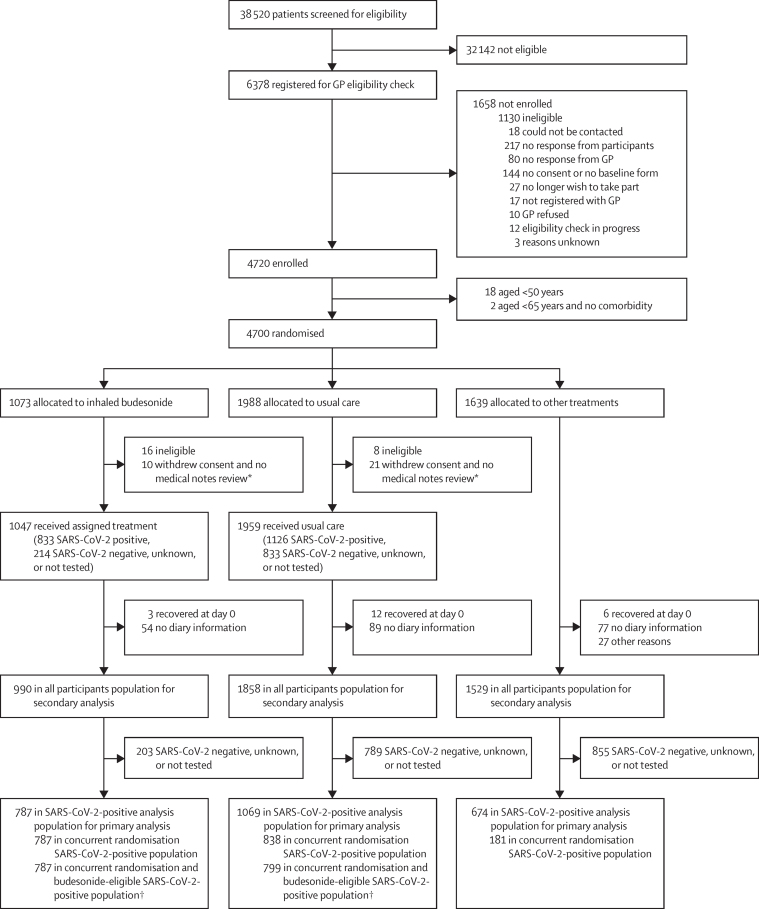
Trial profile GP=general practitioner. *Participants provided no diary information. † Analysis for secondary outcomes.

38 520 patients were screened for eligibility, of whom 4700 were randomly assigned to budesonide (n=1073), usual care alone (n=1988), or other treatments (n=1639; figure 1). 3979 (87%) of 4594 eligible participants had a SARS-CoV-2 test result available, and 2655 (67%) of the 3979 tested positive. To protect the integrity of the platform trial and other interventions, we provide descriptive summaries of only those participants randomly assigned to budesonide and usual care. In SARS-CoV-2-positive participants, the mean age was 64·2 years (SD 7·6), 1805 (92%) of 1959 participants were White, and 1581 (81%) had comorbidities. Median time from symptom onset was 6 days (IQR 4–9). Baseline characteristics were similar between the treatment groups (table 1; appendix pp 237–38). Data on inhaled corticosteroid use was only collected once the budesonide group opened; 27 (3%) of 886 participants in the concurrently randomised usual care group reported taking inhaled corticosteroids at randomisation (these participants had been randomised between usual care and other interventions because they were not eligible for budesonide). Of 969 participants randomly assigned to budesonide who provided medication use information, 772 (80%) reported taking budesonide for at least 7 days.

**Table 1:** Baseline characteristics of SARS-CoV-2-positive participants by treatment group

		**Primary analysis population**		**Concurrent randomisation analysis population**	
		Inhaled budesonide (n=833)	Usual care* (n=1126)	Inhaled budesonide (n=833)	Usual care (n=886)
Age					
	Mean (SD), years	64·7 (7·3)	63·8 (7·8)	64·7 (7·3)	64·5 (7·7)
	50–64 years	297 (36%)	475 (42%)	297 (36%)	322 (36%)
	≥65 years	536 (64%)	651 (58%)	536 (64%)	564 (64%)
Sex					
	Female	429 (52%)	586 (52%)	429 (51%)	455 (51%)
	Male	404 (48%)	540 (48%)	404 (48%)	431 (49%)
Ethnicity†					
	White	767 (92%)	1038 (92%)	767 (92%)	820 (93%)
	Mixed	9 (1%)	5 (<1%)	9 (1%)	4 (<1%)
	South Asian	43 (5%)	64 (6%)	43 (5%)	48 (5%)
	Black	6 (1%)	4 (<1%)	6 (1%)	3 (<1%)
	Other	8 (1%)	14 (1%)	8 (1%)	11 (1%)
	Missing	0	1 (<1%)	0	0
Index of multiple deprivation quintile					
	1 (most deprived)	140 (17%)	196 (17%)	140 (17%)	149 (17%)
	2	157 (19%)	187 (17%)	157 (19%)	156 (18%)
	3	164 (20%)	227 (20%)	164 (20%)	180 (20%)
	4	180 (22%)	252 (22%)	180 (22%)	202 (23%)
	5 (least deprived)	190 (23%)	264 (23%)	190 (23%)	199 (22%)
	Missing	2 (<1%)	0	2 (<1%)	0
Duration of illness before randomisation, days		6·0 (4·0–9·0)	6·0 (4·0–9·0)	6·0 (4·0–9·0)	6·0 (4·0–9·0)
Smoking status					
	Current smoker	44 (5%)	60 (5%)	44 (5%)	45 (5%)
	Former smoker	342 (41%)	460 (41%)	342 (41%)	363 (41%)
	Never smoker	440 (53%)	592 (53%)	440 (53%)	468 (53%)
	Missing	7 (1%)	14 (1%)	7 (1%)	10 (1%)
Received SARS-CoV-2 vaccination		111 (13%)	108 (10%)	111 (13%)	108 (12%)
Vaccine doses received					
	One dose	105 (13%)	100 (9%)	105 (13%)	100 (11%)
	Two doses	6 (1%)	8 (1%)	6 (1%)	8 (1%)
Comorbidity		665 (80%)	916 (81%)	665 (80%)	705 (80%)
Number of comorbidities		1 (1–2)	1 (1–2)	1 (1–2)	1 (1–2)
Comorbidities					
	Asthma, chronic obstructive pulmonary disease, or lung disease	72 (9%)	174 (15%)	72 (9%)	96 (11%)
	Diabetes	169 (20%)	251 (22%)	169 (20%)	200 (23%)
	Heart problems‡	139 (17%)	171 (15%)	139 (17%)	134 (15%)
	High blood pressure requiring medication	382 (46%)	486 (43%)	382 (46%)	388 (44%)
	Liver disease	17 (2%)	22 (2%)	17 (2%)	20 (2%)
	Stroke or other neurological problem	51 (6%)	59 (5%)	51 (6%)	43 (5%)
	Taking angiotensin-converting enzyme inhibitor§	199 (24%)	235 (21%)	199 (24%)	185 (21%)
	Missing	3 (<1%)	6 (1%)	3 (<1%)	3 (<1%)
Fever					
	No problem	414 (50%)	524 (47%)	414 (50%)	413 (47%)
	Mild problem	242 (29%)	359 (32%)	242 (29%)	290 (33%)
	Moderate problem	152 (18%)	207 (18%)	152 (18%)	153 (17%)
	Major problem	25 (3%)	36 (3%)	25 (3%)	30 (3%)
Cough					
	No problem	138 (17%)	186 (17%)	138 (16%)	134 (15%)
	Mild problem	366 (44%)	499 (44%)	366 (44%)	382 (43%)
	Moderate problem	264 (32%)	373 (33%)	264 (32%)	309 (35%)
	Major problem	65 (8%)	68 (6%)	65 (8%)	61 (7%)
Shortness of breath					
	No problem	409 (49%)	522 (46%)	409 (49%)	428 (48%)
	Mild problem	282 (34%)	420 (37%)	282 (34%)	313 (35%)
	Moderate problem	121 (15%)	165 (15%)	121 (14%)	127 (14%)
	Major problem	21 (3%)	19 (2%)	21 (3%)	18 (2%)
Muscle ache					
	No problem	217 (26%)	262 (23%)	217 (26%)	202 (23%)
	Mild problem	263 (32%)	412 (37%)	263 (32%)	326 (37%)
	Moderate problem	246 (30%)	340 (30%)	246 (29%)	265 (30%)
	Major problem	107 (13%)	112 (10%)	107 (13%)	93 (10%)
Nausea or vomiting					
	No problem	572 (69%)	771 (68%)	572 (69%)	595 (67%)
	Mild problem	160 (19%)	239 (21%)	160 (19%)	191 (22%)
	Moderate problem	79 (9%)	88 (8%)	79 (9%)	76 (9%)
	Major problem	22 (3%)	28 (2%)	22 (3%)	24 (3%)
Feeling generally unwell					
	No problem	28 (3%)	46 (4%)	28 (3%)	31 (3%)
	Mild problem	293 (35%)	380 (34%)	293 (35%)	291 (33%)
	Moderate problem	369 (44%)	507 (45%)	369 (44%)	393 (44%)
	Major problem	143 (17%)	183 (16%)	143 (17%)	171 (19%)
	Missing	0	10 (1%)	0	0
Diarrhoea					
	No problem	614 (74%)	822 (73%)	614 (74%)	655 (74%)
	Mild problem	137 (16%)	200 (18%)	137 (16%)	152 (17%)
	Moderate problem	65 (8%)	68 (6%)	65 (8%)	57 (6%)
	Major problem	17 (2%)	26 (2%)	17 (2%)	22 (2%)
	Missing	0	10 (1%)	0	0
Taken antibiotics since illness started		61 (7%)	77 (7%)	61 (7%)	70 (8%)
	Missing	0	1 (<1%)	0	0
Use of health-care services					
	General practitioner	212 (25%)	290 (26%)	212 (25%)	219 (25%)
	Other primary care services	91 (11%)	96 (9%)	91 (11%)	88 (10%)
	NHS 111	93 (11%)	125 (11%)	93 (11%)	94 (11%)
	Accident and emergency	17 (2%)	19 (2%)	17 (2%)	15 (2%)
	Other	25 (3%)	30 (3%)	25 (3%)	25 (3%)
WHO-5 Well-Being Index¶		45·7 (25·3)	46·1 (26·1)	45·7 (25·3)	45·1 (26·3)
	Missing	0	2 (<1%)	0	0

Data are n (%), median (IQR), or mean (SD).

* Includes participants assigned before the inhaled budesonide group was open.

† Data on ethnicity were collected retrospectively via notes review before July, 2020.

‡ Includes angina, heart attack, heart failure, atrial fibrillation, and valve problems.

§ Includes ramipril, lisinopril, perindopril, captopril, or enalapril.

¶ Includes five items relating to wellbeing measured on a five-point scale; a total score is computed by summing the scores to the five individual questions to give a raw score of 0–25, which is then multiplied by 4 to give the final score from 0, representing the worst imaginable wellbeing, to 100, representing the best imaginable wellbeing.

**Figure 2:**
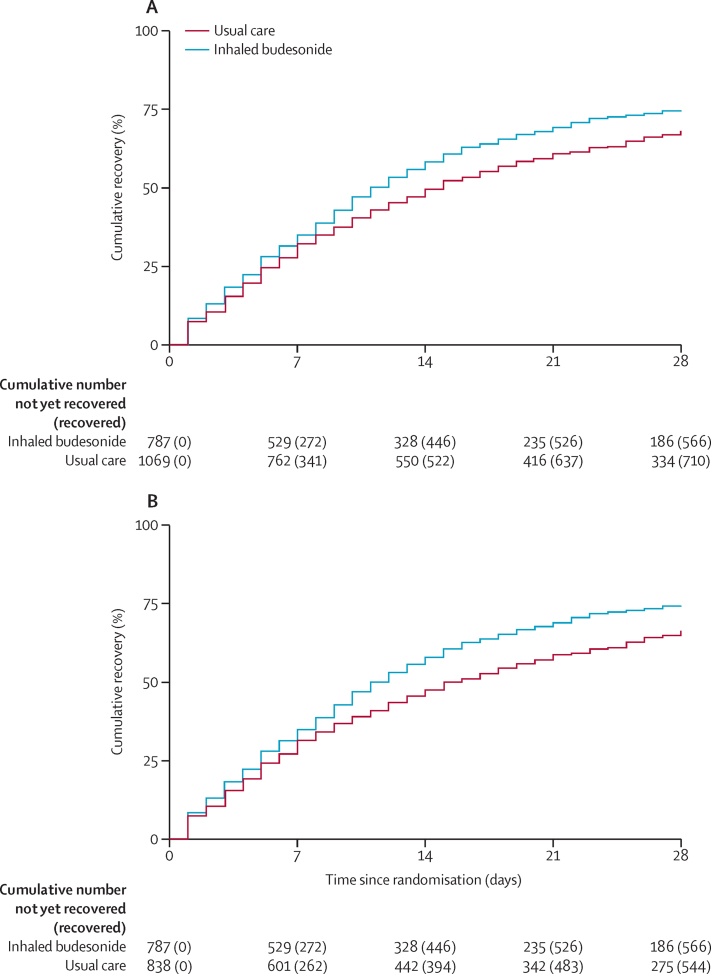
Time to first reported recovery (A) SARS-CoV-2-positive primary analysis population. (B) Concurrent randomisation SARS-CoV-2-positive population.

The Bayesian primary analysis model includes data from 2530 (95%) of 2655 SARS-CoV-2-positive participants who provided follow-up data and were randomly assigned to inhaled budesonide (n=787), usual care alone (n=1069), and other treatments (n=674). In the primary analysis population, the observed median time to first recovery was 11 days (5–not reached) in the inhaled budesonide group compared with 15 days (6–not reached) in the usual care group (figure 2A). Based on the Bayesian primary analysis model, there was evidence of a benefit in time-to-first-recovery in the budesonide group versus usual care group, with a hazard ratio of 1·21 (95% Bayesian credible interval [BCI] 1·08–1·36), an estimated 11·8 days (95% BCI 10·0–14·1) versus 14·7 days (12·3–18·0), and estimated median benefit of 2·94 days (95% BCI 1·19–5·11). The probability of superiority was greater than 0·999, which met the prespecified superiority threshold (table 2). The treatment effect was consistent in the concurrent randomisation and overall study population (table 2, figure 2B).

**Table 2:** Primary outcomes (model-based estimates)

	**Inhaled budesonide (95% BCI)**	**Usual care (95% BCI)**	**Estimated benefit median time to recovery or hospital admission or death rate (95% BCI)**	**Hazard ratio or odds ratio (95% BCI)**	**Probability of superiority**
**Primary analysis—SARS-CoV-2-positive participants**					
Number of participants	787	1069	..	..	..
Time to first reported recovery, days*	11·8 (10·0 to 14·1)	14·7 (12·3 to 18·0)	2·94 (1·19 to 5·11)	1·21 (1·08 to 1·36)	>0·999
Hospital admission or death at 28 days†	6·8% (4·1 to 10·2)	8·8% (5·5 to 12·7)	2·0% (−0·2 to 4·5)	0·75 (0·55 to 1·03)	0·963
**Secondary analysis—all participants**					
Number of participants	990	1858	..	..	..
Time to first reported recovery, days*	10·9 (8·9 to 13·2)	13·3 (11·1 to 16·7)	2·54 (1·00 to 4·54)	1·18 (1·07 to 1·30)	>0·999
Hospital admission or death at 28 days†	5·8% (3·4 to 8·6)	7·3% (4·5 to 10·6)	1·5% (−0·3 to 3·6)	0·78 (0·57 to 1·04)	0·953
**Sensitivity analysis—concurrent randomisation population**					
Number of participants	787	838	..	..	..
Time to first reported recovery, days*	11·7 (9·8 to 14·2)	15·0 (12·5 to 18·3)	3·26 (1·46 to 5·43)	1·24 (1·10 to 1·39)	>0·999
Hospital admission or death at 28 days†	6·6% (3·8 to 10·1)	8·9% (5·2 to 13·1)	2·2% (0·0 to 4·9)	0·73 (0·53 to 1·00)	0·975

BCI=Bayesian credible interval.

* Estimated benefit in median times to recovery are derived from a Bayesian piecewise exponential model adjusted for age and comorbidity at baseline, with 95% BCI; a positive value in estimated benefit in median time to recovery (or hazard ratio >1) corresponds to a reduction in time to recovery in days with budesonide compared with usual care; treatment superiority is declared if probability of superiority is ≥0·99 versus usual care.

† Estimated absolute percentage differences in hospital admission or death were derived from a Bayesian logistic regression model adjusted for age and comorbidity at baseline, with 95% BCI; a positive value in the estimated percentage difference (or odds ratio <1) favours budesonide; treatment superiority is declared if probability of superiority is ≥0·975 versus usual care.

In the primary analysis population, 72 (9%) of 787 participants were admitted to hospital or died due to COVID-19 in the inhaled budesonide group (71 hospital admissions, of whom five died, and one death without hospital admission) compared with 116 (11%) of 1069 in the usual care group (114 hospital admissions, of whom nine died, and two deaths without hospital admission). In the Bayesian primary analysis model comparing hospital admissions or death between the budesonide group and usual care group, the odds ratio was 0·75 (95% BCI 0·55 to 1·03), with an estimated rate of 6·8% (95% BCI 4·1 to 10·2) versus 8·8% (5·5 to 12·7), and an estimated absolute percentage difference of 2·0% (95% BCI –0·2 to 4·5; table 2). The probability of superiority was 0·963, which was below the predefined superiority threshold of 0·975. Results were similar in the concurrent randomisation population (probability of superiority 0·975) and the overall study population (probability of superiority 0·953; table 2).

Analysis of secondary outcomes (table 3), using the concurrent randomisation and eligible SARS-CoV-2-positive population (787 in the budesonide group and 799 in the usual care group), showed evidence of a benefit with budesonide in early sustained recovery, the daily illness severity rating over 28 days (appendix p 231), the WHO-5 Well-Being Index, health-care service use, oxygen administration, time to sustained recovery (appendix p 232), time to sustained alleviation of all symptoms (appendix p 233), and time to reduction of symptom severity (appendix p 234). There was no clear evidence of benefit for any other secondary outcomes.

**Table 3:** Secondary outcomes

		**Inhaled budesonide**	**Usual care**	**Estimated treatment effect (95% CI)**	**p value**
Early sustained recovery		251/781 (32%)	173/794 (22%)	1·48 (1·26 to 1·75)*	<0·0001
Sustained recovery		462/787 (59%)	390/799 (49%)	..	..
	Time to sustained recovery, days	23 (9 to not reached)	28 (15 to not reached)	1·39 (1·21 to 1·59)†	<0·0001
Alleviation of all symptoms		630/701 (90%)	666/732 (91%)	..	..
	Time to alleviation of all symptoms, days	4 (2 to 9)	5 (2 to 10)	1·07 (0·96 to 1·19)†	0·26
Sustained alleviation of all symptoms		579/701 (83%)	597/731 (82%)	..	..
	Time to sustained alleviation of all symptoms, days	8 (3 to 24)	12 (5 to 26)	1·13 (1·01 to 1·27)†	0·037
Initial reduction of severity of symptoms		662/786 (84%)	650/797 (82%)	..	..
	Time to initial reduction of severity of symptoms, days	7 (3 to 14)	8 (3 to 20)	1·19 (1·07 to 1·32)†	0·0019
Illness severity rating (1 worst, 10 best), mean (SD) [n]					
	Day 7	7·0 (1·8) [747]	6·6 (1·9) [759]	0·33 (0·14 to 0·52)‡	0·0001
	Day 14	7·9 (1·7) [745]	7·5 (1·7) [763]	0·37 (0·17 to 0·57)‡	<0·0001
	Day 21	8·4 (1·5) [623]	7·9 (1·6) [612]	0·38 (0·15 to 0·61)‡	0·0001
	Day 28	8·4 (1·5) [759]	8·2 (1·5) [772]	0·19 (−0·07 to 0·44)‡	0·16
WHO-5 Well-Being Index, mean (SD) [n]					
	Day 14	42·5 (25·0) [713]	39·4 (24·4) [724]	2·97 (0·64 to 5·30)‡	0·013
	Day 28	54·6 (25·1) [713]	52·0 (24·8) [721]	2·36 (0·03 to 4·69)‡	0·047
Self-reported contact with at least one health-care service		416/778 (54%)	466/787 (59%)	0·90 (0·83 to 0·98)*	0·017
General practioner reported contact with at least one health-care service		305/602 (51%)	351/607 (58%)	0·87 (0·79 to 0·97)*	0·010
New infections in household		197/772 (26%)	214/782 (27%)	0·93 (0·79 to 1·10)*	0·40
Prescription of antibiotics		42/550 (8%)	53/543 (10%)	0·78 (0·53 to 1·15)*	0·24
Hospital assessment without admission		22/786 (3%)	22/797 (3%)	1·01 (0·57 to 1·82)*	>0·99
Oxygen administration		50/774 (7%)	73/785 (9%)	0·69 (0·49 to 0·98)*	0·039
Mechanical ventilation		13/776 (2%)	14/784 (2%)	0·94 (0·44 to 1·98)§	>0·99
Intensive care unit admission		10/771 (1%)	21/779 (3%)	0·48 (0·23 to 1·01)§	0·068
Duration of hospital admission, days, median (IQR) [n]		9·5 (5 to 28) [70]	10 (4 to 29) [95]	−0·70 (−6·34 to 4·94)¶	0·81
WHO ordinal scale of clinical progression					
	Not admitted to hospital	715/787 (91%)	701/799 (88%)	0·73 (0·53 to 1·01)‖	0·056
	Admitted to hospital without need for supplemental oxygen	17/787 (2%)	21/799 (3%)	..	..
	Admitted to hospital with need for supplemental oxygen	36/787 (5%)	56/799 (7%)	..	..
	Admitted to hospital with need for non-invasive positive pressure ventilation or high-flow nasal cannula	0/787	1/799 (<1%)	..	..
	Admitted to hospital with need for mechanical ventilation or extracorporeal membrane oxygenation	13/787 (2%)	10/799 (1%)	..	..
	Death	6/787 (1%)	10/799 (1%)	..	..

Data are n/N (%) or median (IQR) unless otherwise stated. Patients with data not available were not included in analyses.

* Relative risks adjusted for age, comorbidity at baseline, duration of illness, and vaccination status at baseline.

† Estimated hazard ratio derived from a Cox proportional hazard model adjusted for age, comorbidity at baseline, duration of illness, and vaccination status at baseline, with 95% CI.

‡ Mixed-effects model adjusting for age, comorbidity, duration of illness, vaccination status at baseline, and time; participant was fitted as a random effect; WHO-5 score was also adjusted for the score at baseline.

§ Unadjusted relative risks due to low event rate.

¶ Adjusted difference in medians derived from quantile regression adjusted for age, comorbidity at baseline, duration of illness, and vaccination status at baseline.

‖ Proportional odds ratio derived from ordinal logistic regression adjusted for age, comorbidity at baseline, duration of illness, and vaccination status at baseline.

**Figure 3:**
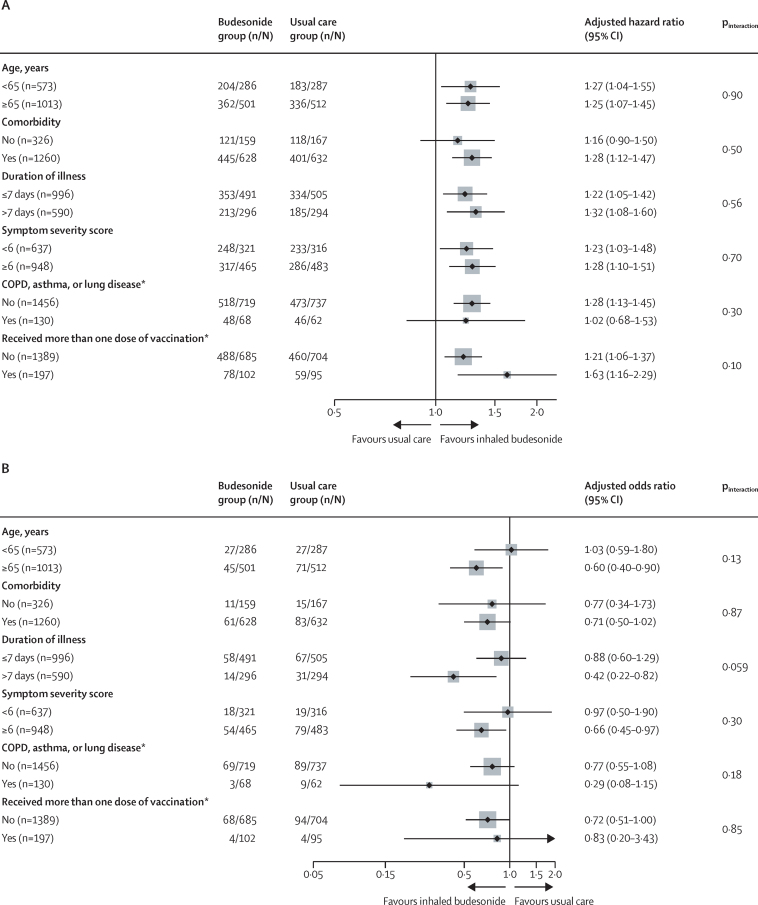
Forest plot of subgroup analysis of time to first reported recovery (A) and COVID-19-related hospital admission or death (B) in the concurrent randomisation and budesonide-eligible SARS-CoV-2-positive population COPD=chronic obstructive pulmonary disease. *Not prespecified.

In the prespecified subgroup analyses, there was no evidence that symptom duration before randomisation, baseline symptom severity score, age, or comorbidity modified the effect of budesonide on time to first reported recovery or hospital admission or death (figure 3). In post-hoc subgroup analyses, there was no evidence that the effect of budesonide differed by vaccination status or chronic lung disease status (figure 3), although numbers were small, particularly for chronic lung disease because those already using inhaled corticosteroids were ineligible for randomisation to budesonide. Regarding serious adverse events, there were two hospital admissions unrelated to COVID-19 in the budesonide group and four in the usual care group (appendix p 241).

## Discussion

This analysis from a platform, randomised trial involving people in the community with COVID-19 at increased risk of an adverse outcome, found that participants using inhaled budesonide recovered an estimated 2·94 days sooner, had a greater sense of wellbeing while recovering, and once recovered, more often remained well (sustained recovery). The budesonide group did not meet the prespecified superiority threshold for the COVID-19-related hospital admission or death outcome before data cutoff, but this might have been due to the rapid decrease in rate of hospital admissions or deaths in March and April, 2021, in the UK, because of the vaccination programme and lockdown measures. Overall, the consistency of these findings across both primary and secondary endpoints provides the strongest evidence thus far of an effective, safe, cheap, and readily available treatment for COVID-19 in the community.

PRINCIPLE is the largest randomised trial thus far to assess inhaled budesonide for community treatment of COVID-19. Our results are consistent with the STOIC phase 2 trial of 146 adults, in which inhaled budesonide reduced COVID-19-related emergency assessments or hospital admissions, compared with usual care, and self-reported recovery favoured budesonide by 1 day.^13^ Several randomised trials have shown that systemic corticosteroids reduce mortality among people admitted to hospital with COVID-19,^8^, ^9^ with the RECOVERY trial finding greatest benefit in mechanically ventilated patients, with no benefit or possibly harm in patients admitted to hospital not requiring oxygen.^8^

The pragmatic design of the PRINCIPLE trial allowed for efficient analysis of the effectiveness of budesonide as an early, standalone intervention as it might be used in the community. We focused on patients at increased risk of complications, explaining the higher proportion who were admitted to hospital than in other community COVID-19 trials.^24^, ^27^ We used routine electronic health records to confirm hospital admission or death, and obtained primary outcome data on more than 95% of participants. We did secondary analyses of the coprimary outcomes in patients with suspected COVID-19 but without PCR-confirmed SARS-CoV-2 infection, because this reflects community testing conditions early in the UK pandemic, and low availability of SARS-CoV-2 testing might necessitate early empirical treatment in other community and low-resource settings. Furthermore, variation in PCR testing sensitivity, particularly when the test is self-administered, means that some participants will have had false negative tests.^28^ Primary outcome estimates were similar in the SARS-CoV-2-positive population, all participants irrespective of SARS-CoV-2 status, and the concurrent randomisation SARS-CoV-2-positive population.

Similar to other large COVID-19 platform trials,^8^, ^29^ we used a pragmatic, open-label design because we aimed to assess the addition of budesonide to usual care, rather than to assess benefit of budesonide compared with a placebo. Our study therefore answers a question that is immediately relevant to policy makers—what would be the effect, compared with usual care, of introducing inhaled budesonide for community treatment of COVID-19? However, inhalers have been documented to have placebo effects in chronic respiratory conditions, which could have affected our self-reported time-to-recovery outcome. We used this outcome because it was of greatest interest to our patient and public contributors and is best ascertained by direct patient report, rather than by surrogate measures. We found no evidence of a placebo effect in analyses of other (pill) treatments in this trial platform,^15^, ^16^ and the hospital admissions or deaths outcome is less likely to be influenced by placebo effects.

This trial has provided evidence of a safe and cheap community treatment for COVID-19 that reduces symptom burden and enhances sustained recovery over 28 days, with a high probability of also reducing the need for hospital admission, albeit just below the prespecified superiority threshold in the primary analysis population. With ongoing, severe COVID-19 surges occurring globally, the need for effective, accessible treatments in the community that can reduce illness duration and prevent overburdening of hospitals and health-care services remains an urgent global priority. Inhaled budesonide is available in many primary care settings and is included in the WHO list of essential medicines.^30^ Further work is needed to establish how budesonide affects COVID-19 pathophysiology, the effectiveness of other inhaled corticosteroids, and the effect on so-called long COVID. A small proportion of our study population had received a SARS-CoV-2 vaccine. Although we found no evidence of a difference in the effect of budesonide by vaccination status, this analysis was probably underpowered. Further work is needed to establish the effect of budesonide among fully vaccinated people with COVID-19.

Our study provides evidence that inhaled budesonide is an effective and safe treatment for people with COVID-19 in the community who are at increased risk of adverse outcomes.

## Data sharing

Data can be shared with qualifying researchers who submit a proposal with a valuable research question as assessed by a committee formed from the trial management group, including senior statistical and clinical representation. A contract should be signed.

## Declaration of interests

MB reports grants from AstraZeneca, personal fees from AstraZeneca, Chiesi, and GlaxoSmithKline; and is a member of advisory boards for Albus Health and ProAxsis, outside the submitted work. DR reports being a former employee of GlaxoSmithKline, outside the submitted work. BRS, NB, MAD, MF, and CS report grants from The University of Oxford, for the University of Oxford's grant from the UK National Institute for Health Research (NIHR) and for statistical design and analyses for the PRINCIPLE trial during the conduct of the study. SdL is Director of the Oxford–Royal College of General Practitioners (RCGP) Research and Surveillance Centre and reports that through his university he has had grants outside the submitted work from AstraZeneca, GlaxoSmithKline, Sanofi, Seqirus, and Takeda for vaccine-related research, and membership of advisory boards for AstraZeneca, Sanofi, and Seqirus. MIA reports grants and personal fees from Prenetics outside the submitted work. PJB reports grants and personal fees from AstraZeneca and Boehringer Ingelheim; and personal fees from Teva and Covis, during the conduct of the study. REKR reports grants from AstraZeneca and personal fees from Boehringer Ingelheim, Chiesi UK, and GlaxoSmithKline, during the conduct of the study. SR reports grants and non-financial support from Oxford respiratory NIHR Biomedical Research Centre (BRC), during the conduct of the study; and non-financial support from AstraZeneca and personal fees from the Australian Government Research Training Program, outside the submitted work. FDRH and CCB report grants from UK Research and Innovation, during the conduct of the study. All other authors declare no competing interests.
