# Supplementary material for: Inhaled budesonide for COVID-19 in people at high risk of complications in the community in the UK (PRINCIPLE): a randomised, controlled, open-label, adaptive platform trial
Source: Lancet. 2021 Sep 4;398(10303):843–55. doi: 10.1016/S0140-6736(21)01744-X (PMC8354567; doi:10.1016/S0140-6736(21)01744-X)
Supplement: Supplementary appendix [file mmc1.pdf]

THE LANCET

Supplementary appendix

This appendix formed part of the original submission and has been peer reviewed. We post it as supplied by the authors.

Supplement to: Yu L-M, Bafadhel M, Dorward J, et al. Inhaled budesonide for COVID-19 in people at high risk of complications in the community in the UK (PRINCIPLE): a randomised, controlled, open-label, adaptive platform trial. *Lancet* 2021; published online Aug 10. http://dx.doi.org/10.1016/S0140-6736(21)01744-X.

Supplementary Appendix Content

Appendix 1: PRINCIPLE Collaborative Group ………………………………………………………………...2

Appendix 2: PRINCIPLE Master Trial Protocol version 7.1 ……………………………………………….4

Appendix 3: Master Statistical Analysis Plan version 3.1 ……………………………………………….81

Appendix 4: PRINCIPLE Adaptive Design Report version 4.0 130

Figure S1: Estimated mean and 95% confidence interval of daily rating of feeling well over 28 days by treatment arm (concurrent randomisation and eligible population) ………………………………………………………………………………….231

Figure S2: Summary and results of the time to sustained recovery (concurrent randomisation and eligible population) ………………………………………………………232

Figure S3: Secondary Time-to-Event Outcomes (the concurrent randomisation and eligible SARS-CoV-2 positive population)

a) Time to alleviation of all symptoms, and each symptom …………………………..233
b) Time to sustained alleviation of all symptoms, and each symptom …………..233
c) Time to initial reduction of severity of all symptoms, and each symptom ...234

Figure S4: Secondary Time-to-Event Outcomes (the concurrent randomisation and eligible population, irrespective of SARS-CoV-2 status)

a) Time to alleviation of all symptoms, and each symptom …………………………..235
b) Time to sustained alleviation of all symptoms, and each symptom …………..235
c) Time to initial reduction of severity of all symptoms, and each symptom ...236

Table S1: Baseline characteristics for participants irrespective of SARS-CoV-2 status by treatment group population…………………………………………………………. .237

Table S2: Secondary Outcomes (concurrent randomisation and eligible population irrespective of SARS-CoV-2 status) ………………………………………………239

Table S3: Reasons for hospitalisation unrelated to COVID-19 ……………………………………..241

Appendix 1

PRINCIPLE COLLABORATIVE GROUP

PRINCIPLE Trial Management Group (TMG)

Julie Allen[1], Monique Andersson[2], Nick Berry[3*], Emily Bongard[1], Aleksandra Borek[1], Christopher C Butler[1] (Chair), Simon de Lusignan[1], Jienchi Dorward[1,4], Philip H Evans[5,6], Filipa Ferreira[1], Oghenekome Gbinigie[1], Jenna Grabey[1], Gail Hayward[1], FD Richard Hobbs[1], Susan Hopkins[7], David Judge[1], Mona Koshkouei[1], Martin J Llewelyn[8], Emma Ogburn[1], Mahendra G. Patel[1], Dan Richards-Doran[1], Heather Rutter[1], Benjamin R. Saville[2,9], Hannah Swayze[1], Nicholas PB Thomas[5,10], Manasa Tripathy[1], Sarah Tonkin-Crine[1,11], Jared Robinson[1], Sharon Tonner[1], Oliver Van Hecke[1], Ly-Mee Yu[1]
* TMG membership ended when NB became unblinded and joined the SAC

Trial Management Group affiliations

1. Nuffield Department of Primary Care Health Sciences, University of Oxford, Oxford, UK
2. Department of Microbiology, Oxford University Hospitals NHS Trust, Oxford, UK
3. Berry Consultants, Texas, USA,
4. Centre for the AIDS Programme of Research in South Africa (CAPRISA), University of KwaZulu–Natal, Durban, South Africa.
5. National Institute for Health Research, Clinical Research Network
6. College of Medicine and Health, University of Exeter
7. Public Health England, London, UK
8. Brighton and Sussex Medical School, University of Sussex, Brighton, UK
9. Department of Biostatistics, Vanderbilt University School of Medicine, Tennessee, USA
10. Royal College of General Practitioners, London, UK
11. National Institute for Health Protection Research Unit in Healthcare Associated Infections and Antimicrobial Resistance, Oxford, UK

Statistical Analysis Committee

Nick Berry, Michelle A. Detry (Chair), Christina Saunders, Mark Fitzgerald

Trial Steering Committee

Carol Green, Phil Hannaford, Paul Little (Chair), Tim Mustill, Matthew Sydes

Data Monitoring and Safety Committee

Deborah Ashby (Chair), Nick Francis, Simon Gates, Gordon Taylor, Patrick White

Principle Trial Coordinating Office

Co-Chief Investigators: CC Butler, FDR Hobbs

Trial management: E Ogburn (coordinator), H Swayze, E Bongard, J Allen, S Tonner, R Edeson, J Brooks, R Edwards, N Maeder, S Barrett, S Brann, A Maloney, K Dempster, J de Henau, J Robinson, N Begum, Micheal McKenna, Clare Bateman, Rebecca Lowe

Clinical Team: H Rutter (Coordinator), K Madronal, B Mundy, B Ianson, I Noel, B Thompson, O Gbinigie, J Dorward, G Hayward, O van Hecke, N Jones, H van der Westhuizen, K Kotze

Data and Programming Team: D Judge, J Grabey, L Castello, D Watt, R Zhao

Statistics: B Saville, L-M Yu, N Berry, M Detry, V Harris, C Saunders, M Fitzgerald, J Mollison, M Shanyinde

Oxford-Royal College of General Practitioner's Research and Surveillance Centre: S de Lusignan (coordinator) M Tripathy, F Ferreira

National Institute for Health Research Clinical Research Network Coordinating Centre: PH Evans, NPB Thomas, H Collins, K Priddis, L Owen, K Hannaby, B Drew

Appendix 2

Trial Title: Platform Randomised trial of INterventions against COVID-19 In older peoPLE

Internal Reference Number / Short title: PRINCIPLE
Ethics Ref: 20/SC/0158
IRAS Project ID: 281958
EudraCT Number: 2020-001209-22
Date and Version No: 22nd February 2021 version 7.1

© Copyright: The University of Oxford and Oxford University Hospitals NHS Foundation Trust 2019

Platform Randomised trial of INterventions against COVID-19 In older peoPLE (PRINCIPLE): Overview

Background: There is an urgent need to identify effective treatments for SARS-CoV-2 infection that helps people recover quicker and reduces the need for hospital admission. We have established an open, adaptive, platform trial to evaluate treatments suitable for use in the community for treating COVID-like-illness that might help people recover sooner and prevent hospitalisation.

Eligibility and randomisation: This protocol describes a randomised trial for people in the community at higher risk of an adverse outcome from possible or confirmed SARS-CoV-2 infection, defined in accordance with the United Kingdom's National Health Service syndromic case definition (https://www.nhs.uk/conditions/coronavirus-covid-19/symptoms/). Participants are randomised to receive either usual care or a trial treatment in addition to usual care (see Intervention Specific Appendices). Participants can take part in the study if they are eligible to be randomised to at least one intervention arm, as well as the Usual Care arm.

Platform trial: A "platform trial" is a trial in which multiple treatments for the same disease are tested simultaneously. New interventions can be added or replace existing ones during the course of the trial in accordance with pre-specified criteria.

Response adaptive randomisation: The initial randomisation ratio is fixed 1:1 for a comparison between two trial arms, but the trial has the capability for these proportions to be altered according to participants' responses to interventions. Pre-specified decision criteria allow for dropping a treatment for futility, declaring a treatment superior, or adding a new treatment to be tested. If at any point a treatment is deemed superior to the usual care arm, the superior treatment may replace the usual care arm as the new standard of care. In the context of the COVID-19 pandemic, the trial may continue as long as the pandemic persists.

Outcomes: The trial has co-primary endpoints: 1) Time taken to self-reported recovery from randomisation; and 2) hospitalisation and/or death. The main objective of the trial is to assess the effectiveness of the interventions in reducing time to recovery and in reducing the incidence of hospitalisation and/or death.

Key secondary outcomes include: Hospital assessment without admission; Oxygen administration; Intensive Care Unit admission; Mechanical ventilation (components of the WHO Clinical Progression Ordinal Scale); Duration of hospital admission; Duration of severe symptoms; Sustained recovery; Contacts with the health services; Consumption of antibiotics; Effects in those with a positive test for COVID-19 infection; WHO Well-being Index; daily rating of how well participant feels; Safety.

See *supplementary material C* for details of objectives and outcome measures.

Efficient study design: All enrolment (screening, informed consent, eligibility review and baseline data) and follow-up procedures (daily diary, data capture of hospitalisations and deaths) can be performed and captured online on the trial website or by telephone with a member of the trial

© Copyright: The University of Oxford and Oxford University Hospitals NHS Foundation Trust 2019

team. Randomisation is online and automatic following eligibility confirmation. Participant packs and medications are sent from the central study team directly to the participant.

Data to be recorded: We will capture demographic features including ethnicity and care home residency at baseline. In the online daily diary (completed for 28 days)/ during telephone calls, participants or their Study Partners will rate the severity of symptoms including how well they are feeling, record contacts with the health services (including hospital admission), record medication use, and new infections in the household. The WHO-5 Wellbeing Index, a five-question instrument, will assess wellbeing at baseline and on days 14 and 28. Follow-up beyond 28 days after randomisation will be by accessing electronic medical records and by participant questionnaire for information relevant to the longer term consequences of COVID-19.

Numbers to be randomised: The trial will continue until either superiority or futility is claimed for each intervention. We estimate that approximately 400 participants per arm (800 participants total if only a single intervention vs. Usual Care) will be required to provide 90% power for detecting an approximate difference of 2 days in median recovery time in the primary analysis population. We estimate that approximately 1500 participants per arm (3000 participants total if only a single intervention vs. usual care) will be required to provide 90% power for detecting a 50% reduction in the relative risk of hospitalisation and/or death in the primary analysis population.

To enquire about the trial, contact the PRINCIPLE Trial Team:

PRINCIPLE Trial
Nuffield Department of Primary Care Health Sciences
Radcliffe Primary Care
Radcliffe Observatory Quarter, Woodstock Road
Oxford
OX2 6GG

Email Address: principle@phc.ox.ac.uk

Tel: 0800 1385451
Website: www.principletrial.org

© Copyright: The University of Oxford and Oxford University Hospitals NHS Foundation Trust 2019

1. BACKGROUND and RATIONALE

We urgently need to know whether potential treatments for COVID-19-like-illness that are suitable for use in the community might help affected individuals recover more quickly and reduce the risk of hospitalisation and/or death.(1) PRINCIPLE is a platform trial designed to efficiently evaluate potential treatments for people with COVID-19-like-illness in the community, and who may be at higher risk of poorer outcomes. Eligible participants are those who meet the United Kingdom's National Health Service syndromic case definition (https://www.nhs.uk/conditions/coronavirus-covid-19/symptoms/), who are being managed in the community, and who are aged 65 and over, or 18 to 64 and experiencing shortness of breath as part of COVID-19 illness, or aged 18-64 with certain comorbidities (2-6)

The platform trial has the flexibility to allow additional interventions to be added in, or to replace existing interventions according to pre-specified criteria. If at any point a treatment is deemed superior to the usual care arm, the superior treatment may replace the usual care arm as the new standard of care. All approved intervention arms are outlined in Intervention Specific Appendices (ISAs).

The trial has co-primary endpoints: 1) Time taken to self-reported recovery from randomisation; and 2) Hospitalisation and/or death. The main objective of the trial is to assess the effectiveness of the respective interventions in reducing time to recovery and in reducing the incidence of hospitalisation and/or death.

The primary analysis will include all participants as specified in the master statistical analysis plan and the adaptive design report. Clinical data, and information from swab and blood tests, where available, will be used to classify participants according to aetiology.

2. TRIAL DESIGN AND PROCEDURES

PRINCIPLE is an open, prospective, individually randomised, platform, response adaptive, controlled clinical trial in community care.

2.1 Participant Identification

2.1.1 Trial Participants

The trial aims to include symptomatic participants with confirmed, or possible COVID-19 who meet the current NHS case definition for possible COVID-19, and who are well enough to remain in the community. This definition can be found here: https://www.nhs.uk/conditions/coronavirus-covid-19/symptoms/. Participants must be aged 65 and over, **OR** aged 18 to 64 and experiencing shortness of breath as part of COVID-19 illness, **OR** aged 18-64 with certain comorbidities.

Participants experiencing shortness of breath have a greater risk of severe and critical disease outcomes with COVID-19 (5).

The study is for people who have ongoing symptoms.

© Copyright: The University of Oxford and Oxford University Hospitals NHS Foundation Trust 2019

2.1.2 Inclusion Criteria

Inclusion requires the following:

1. Participant or their legal representative, is willing and able to give informed consent for participation in the study, and is willing to comply with all trial procedures

2. Suspected COVID-19 using the NHS syndromic definition, OR symptoms consistent with COVID-19* and with a positive test for SARS-CoV-2 infection within the past 14 days

3. Symptoms must have started within the past 14 days and be ongoing

AND

4. Participant is aged 65 or over **OR**

Participant is aged 18- 64, and is experiencing shortness of breath as part of COVID-19 illness **OR**

Participant is aged 18-64 and has any of the following underlying health conditions

a) Known weakened immune system due to a serious illness or medication (e.g. chemotherapy);
b) Known heart disease and/or a diagnosis of high blood pressure
c) Known chronic lung disease (e.g. asthma)
d) Known diabetes
e) Known mild hepatic impairment;
f) Known stroke or neurological problem;
g) Self-report obesity or body mass index ≥35 kg/m^2

*These symptoms may include, but are not limited to, shortness of breath, general feeling of being unwell, muscle pain, diarrhoea and vomiting.

2.1.3 Exclusion Criteria

- Patient currently admitted in hospital
- Almost recovered (generally much improved and symptoms now mild or almost absent)
- Judgement of the recruiting clinician deems ineligible.
- Previous randomisation to an arm of the PRINCIPLE trial

Additional exclusions specific to each intervention arm are listed in the ISAs. For participation, participants must be eligible to be randomised to at least one intervention arm as well as the Usual Care arm.

2.2 Trial procedures

2.2.1 Recruitment

Recruitment is possible through a variety of mechanisms:

© Copyright: The University of Oxford and Oxford University Hospitals NHS Foundation Trust 2019

2.2.2 Face to face

Attending clinicians including video consultations, including research nurses or other health care professionals, at general medical practices, paramedic services, hospital emergency departments, clinical care hubs, Hospital at Home facilities, care of the elderly services, pharmacies, social care services, residential and nursing homes, or any health and social care facility, can facilitate recruitment into the trial. They can do this by discussing the study with potentially eligible participants, guiding them through informed consent procedures, collection of baseline data, completion of screening questions, collecting information for eligibility assessment, and randomising the participant. If required and appropriate, licensed prescribers may prescribe the medication appropriate to the group to which the participant is randomised. Alternatively, health care professionals may revert to the PC-CTU to complete the activity, including eligibility confirmation and issue of study medication and materials.

2.2.3 Remote recruitment

i) All Health, health related, and Social Care professionals will be able to give information verbally or via a trial text, email, poster, social media post, adverts, media release, leaflet or letter, to potential study participants and their study partners. They may also direct patients to the online study information and the study website.

ii) Potential participants may present directly to the study team via the website or by the study telephone contact. The study team can provide information about joining the trial and guide them through the consent and enrolment process.

iii) A General Practice may be contacted by a potential participant or the practice may contact patients, by text (or by letter), who may match the trial eligibility criteria, through running searches of their database. They will then direct patients to the trial enrolment website or seek verbal consent to be contacted by the trial team.

iv) NHS Digital will provide the PRINCIPLE trial with a daily list of contact details from the COVID-19 testing Pillar 2 data, for patients receiving a positive test result for SARS-Co-V2 infection, via a secure transfer system. NHS Digital will apply an age filter to ensure only the details of those within the age range of the trial are passed on to PRINCIPLE. The trial team will make a limited number (maximum of 3) of attempts to telephone, text or email these patients to provide them with information about the trial, to invite them to consider taking part, and to enrol them if they provide full informed consent and are deemed eligible at screening.

Patient details will be provided in accordance with section 251 under the General Notice under the Health Service Control of Patient Information Regulations 2002, which applies only in England and Wales, providing patient information without consent for COVID-19 public health, surveillance and research purposes. The notice provides a temporary legal basis to avoid a breach of confidentiality for COVID-19 purposes.

v) Join Dementia Research (JDR) - We will also be using JDR as a recruitment tool. This is an on-line self-registration service that enables volunteers with memory problems or dementia, carers of those with memory problems or dementia and healthy volunteers to register their interest in taking part in research.

© Copyright: The University of Oxford and Oxford University Hospitals NHS Foundation Trust 2019

For all recruitment models:

- Study Partner: at screening the potential participant will be asked to provide contact details for a Study Partner, to assist in completing trial procedures and to provide information on their behalf where necessary, but this is not a requirement for trial participation. However, it is strongly encouraged that participants who may be frailer and/or lack capacity to consent make use of a study partner to facilitate their participation. In addition to family member or friend, the study partner may also be a carer or other suitable person.
- Participants may be asked if they wish to enrol in additional studies that do not conflict with the main PRINCIPLE trial. Those who do not screen as eligible for PRINCIPLE may be alerted to the possibility of participating in other approved trials.

2.3 Screening

An online screening, eligibility and consent procedure is used. If online access is not possible, a member of the trial team collects this information during a telephone call. A trial free-phone number enables participants to contact the trial team for further information and study participation support. Participants are screened after they have read the PIS by completing an online eligibility questionnaire.

2.4 Informed Consent

If participants meet the screening criteria, they will be asked to provide informed consent and a screening trial ID number will be assigned to them. Remote, paperless online/telephone consent is required, and appropriate during the pandemic. Participants will be able to download their consent form, or it may be printed by the central study team and delivered to participants with their study materials if they so prefer.

Written and summary versions of the PIS and ICF will be presented to participants detailing no less than: the exact nature of the trial; the known side-effects and risks involved in taking part. It will be clear that the participant is free to withdraw from the study at any time. A summary, pictorial PIS is available which can be read by those feeling very unwell, lack capacity or have low reading comprehension skills. Adequate time will be given to the participant to consider the information and to ask any questions about the trial before deciding whether to participate. After consent, the participant will enter online baseline information, including their address, contact details and those of a Study Partner.

Population groups such as care home residents have been amongst those hardest hit by the pandemic and therefore stand to benefit the most from any effective treatments. However, some care home residents lack capacity to consent to research themselves. If the recruiting clinician deems a care home resident lacks capacity to consent then a personal or professional legal representative (England and Wales only) will be asked to provide consent for those lacking capacity to consent for themselves. A personal legal representative is defined as a person not connected with the conduct of the trial who is suitable to act as the legal representative by virtue of their relationship with the adult. A professional legal representative may be a doctor responsible for the medical treatment of the adult if they are independent of the study, or a person nominated by the healthcare provider. In all instances, a personal legal representative will

© Copyright: The University of Oxford and Oxford University Hospitals NHS Foundation Trust 2019

be sought first and a professional legal representative sought only if a personal legal representative cannot be identified. A professional legal representative will be sought in order not to deny access to research to older adults who may not have personal legal representatives. Only residents of care homes who lack capacity to consent will be recruited, adults who lack capacity to consent will not be recruited from the wider community. Legal Guardians and recruiting clinicians will not endeavour to obtain consent for or recruit into the trial residents who, in addition to their lack of capacity, have a quality of life which can reasonably be considered as not acceptable to the potential participant.

The legal representative will be provided with information about the trial and made aware of the following:

- They are being asked to give consent on behalf of the incapacitated adult,
- They are free to decide whether they wish to make this decision or not, and
- They are being asked to consider what the adult would want, and to set aside their own personal views when making this decision.

2.5 Eligibility Assessment

Eligibility of those who have provided appropriate consent can be checked at study sites or centrally by a medically qualified clinician or a research nurse, who is suitably trained and experienced and has been delegated this responsibility, and who has appropriate access to the participant's summary care record or relevant medical information. If a participant's summary care record cannot be accessed centrally, the clinician/delegate will contact the participant's primary care medical practice for information relevant to confirming eligibility. Participants will not be randomised to an arm if an exclusion criterion to that arm applies to them, but will need to have no exclusions relevant to at least one intervention and the usual care arm.

2.6 Randomisation

Participants will be randomised using a fully validated and compliant web-based randomisation system called Sortition. Once deemed eligible, the clinician or a member of the trial team will randomise the participant, to one of the arms they are eligible for (at least two arms, usual care and at least one intervention), automatically by Sortition. Full details of response adaptive randomisation are described in section 5.2.2.

The participant, legal representative if applicable, trial team and participant's GP will be notified electronically of the treatment allocation. If the participant does not have an email address, they will be notified by telephone. The research team may also send the GP or Care Home an email or letter via secure systems, containing personally identifiable data and treatment allocation.

2.7 Blinding and code-breaking

© Copyright: The University of Oxford and Oxford University Hospitals NHS Foundation Trust 2019

PRINCIPLE is an open-label trial. The participant, legal representative if applicable, and the recruiting clinician will know the participant's allocation. Therefore, no unblinding or code breaking is required. However, those managing the data will be blind to participant allocation.

The trial team and recruiting clinicians will be blinded to emerging results. During the course of the trial, only the unblinding statisticians and the independent members of the Data Monitoring and Safety Committee will have access to the unblinded interim results.

2.8 Baseline Assessments

Once randomised, study medication (if so randomised), and a participant pack will be sent to participants, from their general practice, study team, Public Health England (PHE) or other approved central service (or collected from a general practice or pharmacy). Participants may be offered a swab test as part of standard care. Where possible, and availability of sampling kits allows, one sample will be taken as close to study entry as possible to assess COVID-19 status and other viral aetiologies. While the aim is to have a swab result for all patients, where swabs are unavailable, patients may still participate and be included in the primary intention to treat analysis only.

2.9 Subsequent Visits

There is no requirement for participants to have a face-to-face visit as part of trial participation. Those participants randomised to an unlicensed medication will receive a call from the study team within one day of randomisation, to reaffirm consent, to explain when to call the 24 hour safety phone line, what to do in an emergency, and to answer any other trial questions. This information is also included in the participant information sheet and the participation pack that they will receive. All subsequent measurements consist of self-completed questionnaires online or through telephone calls, and primary care and/or hospital record searches. We will ascertain relevant data from primary care and/or hospital medical records about length of hospital stay, oxygen therapy, and ICU admission and ventilation, if applicable.

Participants will be sent a link to their online diary, which they will be asked to complete for 28 days. They will be asked to rate the severity of symptoms, record contacts with the health services (including hospital admission), record medication use and new infections in the household. It is becoming increasingly apparent the COVID-19 infection may have a considerable negative impact on well-being (7) and so the five questions of WHO-5, validated for measuring wellbeing over time, will be presented at baseline and on days 14 and 28. We will not ask for WHO-5 questions to be completed for participants who lack capacity. We will capture ethnicity and care home residency at baseline and day 28 (if missed at baseline).

All participants receive a call from the trial team on day 2/3 to reaffirm consent, to confirm that they have received a participant pack, and trial medication (if randomised to a trial medication), and to explain that they should complete the daily diary for 28 days even if they feel better or their swab result is negative. The trial team calls participants/study partners on days 7, 14 and 28 if they do not have internet access or have *not* completed their diary for at least 2 consecutive days prior to the call. No more than six contact attempts will be made at each of these follow-up points. For those on unlicensed medication, if the participant/study partner does not answer the

© Copyright: The University of Oxford and Oxford University Hospitals NHS Foundation Trust 2019

calls and hasn't completed online diaries, their GP will be contacted to allow us to monitor any potential side-effects associated with the medication.

We will seek consent from participants to contact them on a monthly basis for up to 12 months after enrolment (via email, text message or phone call) to collect information about any ongoing symptoms, hospitalisations and well-being. We will re-consent those already enrolled in the trial.

In addition to the swab being undertaken as part of the national RCGP RSC surveillance programme with PHE, trial participants will also be asked to consent to the trial team accessing a blood sample result. The study team will obtain the result from RCGP RSC/PHE.

The RCGP RSC will report to the central trial office at regular intervals about healthcare contacts in the participant's clinical records, as they are able to download this information centrally. This will be used as confirmation and a back-up for information obtained directly from study participants and other data sources outlined above. If obtaining data is not possible using this route, the GP surgery will be contacted to request a limited notes review. Participant records will be accessed up to twelve months following enrolment to ascertain follow up data from enrolment to day 28. Data will be collected as close to real time as possible; RCGP RSC, EMIS and NHS Digital and other sources of routinely collected data will be utilised if required To investigate the impact of trial interventions on the longer-term effects of COVID-19, we will use these data collection methods to follow-up participants, for up to 10 years.

2.10 Qualitative Sub-study

A qualitative sub-study will be nested within the trial, to capture data to understand how patients conceptualise their illness and how they respond to taking medication(s) as part of the trial. Once participants have completed the trial, we will interview their respective clinicians to explore their views of taking part in trials during a pandemic. Healthcare professionals will also be asked about their experiences of taking part in the trial. See *supplementary material F* for further details. Participants who lack capacity will not be invited to participate in the qualitative sub study.

2.11 Early Discontinuation/Withdrawal of Participants

Each participant, or their legal representative on the participant's behalf, has the right to withdraw from the study at any time. For those that lack capacity, expression of dissent in any form will be taken as an indication they do not wish to be included and they will be withdrawn. In addition, the Investigator may discontinue a participant from the study at any time if the Investigator considers it necessary for any reason including:

- Ineligibility (either arising during the study or retrospectively having been overlooked at screening)
- Withdrawal of consent

The reason for withdrawal will be recorded on the CRF. Data that has already been collected about the participant will be kept and used.

2.12 Definition of End of Trial

© Copyright: The University of Oxford and Oxford University Hospitals NHS Foundation Trust 2019

Last data capture of last participant, when: no further suitable interventions are available and/or COVID-19 is no longer prevalent. March 2022 has been decided as the formal end date at this stage, but this date may need to be amended depending on circumstances prevailing at the time.

3 TRIAL INTERVENTIONS

IMP information can be found in the relevant ISAs.

In general, re-packaging and issuing of medication can be completed by: the patient's registered GP surgery or treatment and assessment facility; an accredited licensed central facility; an online, community or hospital pharmacy, and The Primary Care Clinical Trials Unit (as approved by the MHRA). Distribution of trial packs to participants will be tracked via courier or call/text message. Clinicians can prescribe trial medications that can be issued in the community and pharmacies can issue medication to the patient by community pharmacy services 'on-line pharmacy' services, NHS volunteers, or it can be collected from the pharmacy by the participant or someone on their behalf.

To record presence of symptoms and severity, as well as adherence to trial treatment, participants will receive a daily email asking them to complete an online diary where they will record their symptoms and medicines use. If incomplete, the trial team will contact the participant and/or their Study Partner to obtain the data. Section 2.9 explains the additional oversight of those participants receiving unlicensed medication. A risk-adapted approach will be used for drug accountability. Accountability logs will be kept by PC-CTU when they ship drug.

4 SAFETY REPORTING

All symptoms, medication side-effects and SAEs will be collected from participant daily diaries, calls to participants/Study Partners, medical records, notes reviews and RCGP data downloads. SAE information will be analysed as part of the interim and whole trial analysis and will be reviewed at each Data Safety & Monitoring Committee meeting.

4.1 Procedures for Reporting Adverse Events and Serious Adverse Events

The severity of events and symptoms will be assessed by participants in daily diaries on the following scale: no problem/mild problem/moderate problem/major problem.

	Participant reported symptom rating
No problem	Symptom not experienced
Mild problem	Short-lived or mild symptoms; medication may be required. No limitation to usual activity
Moderate problem	Moderate limitation in usual activity. Medication may be required.
Major problem	Considerable limitation in activity. Medication or medical attention required.

i. **AE reporting**

© Copyright: The University of Oxford and Oxford University Hospitals NHS Foundation Trust 2019

AEs will be monitored daily for participants allocated to either hydroxychloroquine or treatments not licensed in the UK, whilst they are taking the drug, to allow successful safety monitoring of these less familiar treatments. Participants will be free to withdraw from taking the treatment if they perceive they have an intolerable AE.

For each treatment not licensed in the UK, the following AEs from the start of medication until the specified follow-up period, will be assessed by a clinician for causality and severity (definitions below): i) pre-defined AEs detailed in the ISA that are rated by the participant as 'moderate' and ii) other reported 'major' AEs.

Participants will also be provided with a Trial Wallet Emergency Card detailing potential side-effects and a 24-hour contact telephone line, manned by a clinical team, enabling them to report any moderate or major AEs they experience whilst taking the drug. The clinician will contact the participant directly within 24 hrs of becoming aware of a major AE reported in their daily diary or on the Freephone number, to advise the participant on the appropriate clinical care, as well as notifying the participant's GP about the event. In the event of a medical emergency, trial participants will be instructed to show this card to the clinician they see.

ii. AE Severity Assessment (for assessing clinician):

	Clinical assessment of severity
**GRADE 1 (Mild)**	Short-lived or mild symptoms; medication may be required. No limitation to usual activity
**GRADE 2 (Moderate)**	Moderate limitation in usual activity. Medication may be required.
**GRADE 3 (Severe)**	Considerable limitation in activity. Medication or medical attention required.

iii. SAEs

Hospitalisation and/or death due to confirmed or possible SARS-Cov-2 infection is a primary outcome, we will collect this data using a risk-adapted approach and will not report such SAEs. SAEs other than hospitalisation or death due to COVID-19 must be reported for all treatments.

SAEs must be reported by the person who has discovered the SAE or nominated delegate within 24 hours of becoming aware of the event. The sponsor or delegate will ensure it is reviewed by the CI or other delegated personnel for relatedness and expectedness as soon as possible taking into account the reporting time for a potential SUSAR according to the relevant competent authority. If the event has not resolved, at the 28 day time point the SAE will be reviewed again to see if resolution has occurred. If the event is considered 'resolved' or 'resolving' no further follow up is required. If not, the event must be followed up until such a time point.

All SAEs that have not resolved by the end of the study, or that have not resolved upon discontinuation of the participant's participation in the study, must be followed until any of the following occurs:

• The event resolves

© Copyright: The University of Oxford and Oxford University Hospitals NHS Foundation Trust 2019

• The event stabilises

• The event returns to “baseline”, if a “baseline” value/status is available

• The event can be attributed to agents other than the study intervention or to factors unrelated to study conduct

• It becomes unlikely that any additional information can be obtained (participant or health care practitioner refusal to provide additional information, lost to follow-up after demonstration of due diligence with follow-up efforts)

See Supplementary Material D for definitions of adverse events

4.1.1. Other events exempt from immediate reporting as SAEs

Hospitalisations will be defined as at least a one night admission to hospital. Hospitalisation for a pre-existing condition, including elective procedures planned prior to study entry, which has not worsened, does not constitute an SAE, and standard supportive care for the disease under study are not SAEs and do not require SAE reporting.

4.1.2. Procedure for immediate reporting of Serious Adverse Events

- Trial team will complete an SAE report form for all reportable SAEs.
- GP practice/trial team/RCGP will provide additional, missing or follow up information in a timely fashion.
- If necessary the participant may be contacted to provide additional, missing or follow up information as required.

The CI or delegate will review the SAE once reported, collect as much information and report to the Sponsor within the timeframe according to the PC-CTU SOPs.

4.1.3 Expectedness and Causality

For SAEs that require reporting, expectedness of SARs will be determined according to the relevant RSI section of the Summary of Product Characteristics/IB. The RSI will be the current Sponsor and MHRA approved version at the time of the event occurrence.

Assessment of Causality

The relationship of each serious adverse event to the trial medication must be determined by a medically qualified individual according to the following definitions:

- **Unrelated** – where an event is not considered to be related to the IMP
- **Possibly** – although a relationship to the IMP cannot be completely ruled out, the nature of the event, the underlying disease, concomitant medication or temporal relationship make other explanations possible.
- **Probably** – the temporal relationship and absence of a more likely explanation suggest the event could be related to the IMP.

© Copyright: The University of Oxford and Oxford University Hospitals NHS Foundation Trust 2019

- **Definitely** – the known effects of the IMP, its therapeutic class or based on challenge testing suggest that the IMP is the most likely cause.

All AEs/SAEs labelled possibly, probably or definitely will be considered as related to the IMP.

4.2 SUSAR Reporting

All SUSARs will be reported by the sponsor delegate to the relevant Competent Authority and to the REC and other parties as applicable. For fatal and life-threatening SUSARS, this will be done no later than seven calendar days after the Sponsor or delegate is first aware of the reaction. Any additional relevant information will be reported within eight calendar days of the initial report. All other SUSARs will be reported within 15 calendar days.

Principal Investigators will be informed of all SUSARs for the relevant IMP for all studies with the same Sponsor, whether or not the event occurred in the current trial.

4.3 Development Safety Update Reports

The DSUR will be developed and submitted annually on the anniversary date that the trial receives Clinical Trial Authorisation +60 days. Due to the nature of this trial and the importance of sharing the science of COVID-19 and the drug, internationally, we expect to produce reports to the UK Government and regulatory agency more frequently upon request.

5 STATISTICS

5.1 Master Statistical Analysis Plan (M-SAP)

Details of the statistical design and methods will be described in a Master Statistical Analysis Plan (M-SAP), in which an appendix to the M-SAP titled "Adaptive Design Report" (ADR) provides complete specifications for the primary analysis and pre-specified adaptive algorithm. In addition, the M-SAP will be accompanied by arm-specific appendices to describe any planned deviations from the M-SAP. A broad overview of the design and primary analyses is provided below.

5.2 Open Adaptive Platform Trial

PRINCIPLE is an open, adaptive, platform trial to evaluate emerging treatments for symptomatic COVID-19-like illness. A "platform trial" is a trial in which multiple treatments for the same disease are tested simultaneously. The backbone of the trial is an adaptive clinical trial design. Pre-specified decision criteria allow for dropping a treatment for futility, declaring a treatment superior, or adding a new treatment to be tested. If at any point a treatment is deemed superior to the Usual Care arm, the superior treatment may replace the Usual Care arm as the new standard of care. Because the process of dropping and adding treatments may be on-going for an indefinite period of time, platform trials may be better conceived of as a process rather than a singular clinical trial. In the context of the COVID-19 pandemic, the trial may continue as long as the pandemic persists.

The PRINCIPLE trial will begin as a two arm, 1:1 randomised trial but will have the capability to add additional interventions over time. The evaluation of any new interventions will be governed

© Copyright: The University of Oxford and Oxford University Hospitals NHS Foundation Trust 2019

by this master protocol and M-SAP (including adaptive algorithm and decision criteria), with any planned deviations from the master protocol and M-SAP to be specified in arm-specific appendices. The inclusion of any new interventions will require additional arm-specific appendices to the master protocol and M-SAP.

5.2.1 Co-Primary Endpoints & Analyses

There are two co-primary endpoints. The first co-primary endpoint is time to recovery from possible COVID-19 infection within 28 days from randomisation, where time to recovery is defined as the first instance that a participant reports feeling recovered. The second co-primary endpoint is hospital admission or death related to possible or confirmed COVID-19 within 28 days from randomisation. Unless otherwise specified in the Intervention Specific Appendices (ISA), the co-primary outcomes will be evaluated using a "gate-keeping" strategy. For a given treatment, the hypothesis for the time to recovery endpoint will be evaluated first, and if the recovery null hypothesis is rejected, the hypothesis for the second co-primary endpoint of hospitalisation/death will be evaluated. This gate-keeping strategy preserves the overall Type I error of the primary endpoints without additional adjustments for multiple hypotheses. In addition, the gate-keeping structure reflects the clinical belief that an intervention is unlikely to demonstrate benefit on the hospitalisation/death endpoint without first demonstrating benefit on the time to recovery endpoint.

The primary outcome of time to recovery is defined as the first instance that a participant reports feeling recovered. The corresponding primary analysis for this outcome is a Bayesian piecewise exponential model, with time to recovery regressed on treatment and stratification covariates (age, comorbidity). Let θ_j denote the log hazards ratio comparing the hazards of recovery for participants in treatment group *j* versus participants in the Usual Care arm. A corresponding Bayesian posterior distribution will be derived for the estimated log hazards ratio. The first co-primary analysis for intervention *j* will test the following hypothesis:

$$H_{10}:\ \theta_j \leq 0$$
$$H_{11}:\ \theta_j > 0$$

If the Bayesian posterior probability of superiority (a log hazards ratio greater than 0 corresponding to quicker recovery) for a treatment versus Usual Care is sufficiently large (e.g. ≥ 0.99), the null hypothesis will be rejected and the intervention will be deemed superior to Usual Care with respect to time to recovery. The exact threshold of the superiority decision criterion (e.g. 0.99) will be determined *a priori* via simulation to control the one-sided Type I error of the study at approximately 0.025, and will be specified in the Adaptive Design Report (Appendix to the M-SAP). The Adaptive Design Report will also specify appropriate methodology for the primary analysis when the Usual Care arm is replaced by a superior treatment, and for when the comparison of a treatment versus Usual Care includes non-concurrent randomisations.

The second co-primary endpoint is hospital admission or death due to possible SARS-CoV-2 infection. The corresponding analysis will be a Bayesian generalised linear model of hospitalisation/death regressed on treatment and stratification covariates (age, comorbidity). Let δ_j denote the log odds ratio comparing the odds of hospitalisation/death for persons in treatment group *j* versus persons in the Usual Care arm. A corresponding Bayesian posterior distribution will be derived for the estimated log odds ratio. If the first co-primary endpoint

© Copyright: The University of Oxford and Oxford University Hospitals NHS Foundation Trust 2019

hypothesis (for time to recovery) is rejected for intervention *j*, the second co-primary hypothesis for intervention *j* be tested:

$$H_{20}: \delta_j \leq 0$$
$$H_{21}: \delta_j > 0$$

If the Bayesian posterior probability of superiority on hospitalisation/death for a treatment versus Usual Care is sufficiently large (e.g. ≥ 0.99), the null hypothesis will be rejected and the intervention will be deemed superior to Usual Care with respect to hospitalisation/death. The exact threshold of the superiority decision criterion (e.g. 0.99) will be determined *a priori* via simulation to control the one-sided Type I error of the study at approximately 0.025, and will be specified in the M-SAP.

5.2.2 Adaptive Design

The pre-specified design will allow adaptations to the trial based on the observed co-primary endpoint data. These adaptations include the declaration of success or futility of an intervention at an interim analysis, the addition or removal of treatment arms, and changes in the randomisation probabilities. Adaptations will occur at a given interim analysis if pre-specified conditions are satisfied. The adaptive algorithm will be documented in the Adaptive Design Report, including pre-specified criteria for decisions regarding futility or effectiveness of interventions and/or replacing interventions in the trial.

5.2.3 Interim Analyses

Precise timing of the first interim analysis and frequency of subsequent interim analyses will be specified in the Adaptive Design Report, based on both simulations and logistical considerations. At each interim analysis, all enrolled intervention arms will be evaluated for success and futility on both co-primary endpoints using the Bayesian primary analyses. These interim analyses will maintain the gate-keeping sequential order by first evaluating the hypothesis for time to recovery, and if the recovery endpoint null hypothesis is rejected, subsequently evaluating the hypothesis for hospitalisation and/or death. If the Bayesian posterior probability of superiority of a given intervention versus Usual Care is sufficiently large for a given endpoint (e.g. ≥ 0.99) within the gate-keeping structure, superiority will be declared versus Usual Care with respect to that endpoint.

If the Bayesian posterior probability of a clinically meaningful treatment effect is sufficiently small (e.g. < 0.01) for the first co-primary endpoint (time to recovery), the intervention arm may be dropped from the study for futility. If there are no other intervention arms available, the trial may be suspended; otherwise accrual continues to the remaining treatment arms. The exact futility thresholds will be pre-specified in the Adaptive Design Report and determined via simulation.

5.2.4 Allocation & Response Adaptive Randomisation

Initially, randomisation will be fixed 1:1 for a comparison between two trial arms, with stratification by age (less than 65, greater than or equal to 65), and comorbidity (yes/no). If a second experimental intervention arm is added to the study, randomisation allocation will be modified and the additional intervention will be included in the interim analyses (with evaluation

© Copyright: The University of Oxford and Oxford University Hospitals NHS Foundation Trust 2019

for success and futility) as detailed in the Adaptive Design Report. If there are at least 3 arms (2 intervention arms plus Usual Care) in the study, each interim analysis may incorporate modified randomisation probabilities via response adaptive randomisation (RAR). Full details for implementing RAR will be provided in the Adaptive Design Report; the general idea is to allocate more participants to the intervention arms that have the best observed outcomes. Except for the CTU programmer, the rest of the trial team are blinded to the RAR ratios.

5.2.5 Sample Size Justification

Given the open perpetual trial structure, the trial does not have a finite ending based on sample size. Rather, the trial will continue until either superiority or futility is claimed for each intervention, or until the pandemic expires in the population. We estimate that approximately 400 participants per arm (800 participants total if only a single intervention vs. Usual Care) will be required to provide 90% power for detecting a hazard ratio of 1.3 (approximate difference of 2 days in median recovery time). This calculation is based on the assumption of an exponential distribution for time to recovery with a median of 9 days in the Usual Care arm, with some adjustments for missing data and multiple interim analyses. On average, we expect fewer participants to be required when there is a large treatment benefit or complete lack of benefit. For example, if the true hazard ratio is 1.5 (3 day benefit in median time to recovery), on average only 150 subjects per arm are required to provide sufficient power. The primary advantage of the adaptive design is the ability to adapt the sample size to the observed data, thus addressing the primary hypothesis as quickly and as efficiently as possible.

We estimate that approximately 1500 participants per arm (3000 participants total if only a single intervention vs. usual care) will be required to provide 90% power for detecting a 50% reduction in the relative risk of hospitalisation and/or death. This calculation is based on the assumption of an underlying 5% combined hospitalisation and/or death rate in the Usual Care arm, with an intervention lowering the hospitalisation and/or death rate to 2.5%, with some adjustments for the multiple interim analyses. We expect fewer participants to be required to detect a 50% reduction if the event rate in the Usual Care arm is greater than 5%.

5.2.6 Virtual Trial Simulations

Because of the adaptive platform trial structure, there exists no simple formula(s) to calculate power and Type I error (false positive rate). Hence, virtual trial simulations will be used to fully characterize and quantify the power and Type I error of the design. These simulations will be conducted prior to the first interim analysis (with results described in the Adaptive Design Report), and will be used to optimize the adaptive decision criterion and RAR parameters. The simulations will include a comprehensive evaluation of trial performance across a wide range of assumptions (e.g. underlying distribution of outcome in Usual Care arm, treatment effect, accrual rates, etc.). This will include summaries regarding the number of subjects required to make a success or futility conclusions for each intervention. Complete details of the simulations will be provided in the Adaptive Design Report.

5.2.7 Procedure for Accounting for Missing, Unused, and Spurious Data.

Full details of handling missing data will be specified in the M-SAP.

© Copyright: The University of Oxford and Oxford University Hospitals NHS Foundation Trust 2019

5.3 Primary Analysis Population

The primary analysis population is defined as all randomised participants with a COVID-19 positive test, according to the groups they were randomly allocated to as specified in the M-SAP. All other analysis populations will be defined in the M-SAP.

5.4 Procedures for Reporting Unplanned Deviation(s) from the Master Statistical Analysis Plan

Analyses will be carried out in accordance with the M-SAP and corresponding appendices. Any additional analysis that is not specified in the M-SAP/appendices or any unplanned deviation(s) from the M-SAP/appendices will be specified in the Statistical Report. Reasons for these changes will be documented and authorised by the Chief Investigator.

5.5 Qualitative sub-study analysis

Audio-recordings of interviews will be transcribed verbatim and transcripts analysed using thematic analysis. Patient and HCP interviews transcripts will be analysed separately but findings will be compared and triangulated if deemed appropriate. Thematic analysis allows the research team to take a pragmatic approach to data collection, remaining grounded in the data but ensuring that the analysis answers the research objectives. NVivo software will be used to assist with the organisation and coding of data. Codes will be compared with one another to create categories, grouping similar codes together. A thematic framework will be developed to code all data and represent key themes for both sets of interviews.

6 DATA MANAGEMENT

The data management aspects of the study are summarised here with details fully described in the Data Management Plan.

6.1 Source Data

Source documents are where data are first recorded. These include, but are not limited to, hospital records (from which medical history and previous and concurrent medication may be summarised into the CRF), clinical and office charts, laboratory and pharmacy records, diaries, microfiches, radiographs, and correspondence.

If a participant fails to complete data online and after six attempts at contacting the participant/Study Partner, the RCGP RSC may be utilised to obtain missing data. Data collected will include participant identifiable information and will be accessed at the University of Oxford according to PC-CTU Information Governance policies and GDPR. Data will only be held for the duration it is required, this will be reviewed annually.

CRF entries will be considered source data if the CRF is the site of the original recording (e.g. there is no other written or electronic record of data). All documents will be stored safely in confidential conditions. On all study-specific documents, other than the signed consent, the participant will be referred to by the study participant number/code, not by name.

© Copyright: The University of Oxford and Oxford University Hospitals NHS Foundation Trust 2019

6.2 Access to Data

Direct access will be granted to authorised representatives from the Sponsor and host institution for monitoring and/or audit of the study to ensure compliance with regulations.

6.3 Data Recording and Record Keeping

In accordance with the principles of Good Clinical Practice and the recommendations and guidelines issued by regulatory agencies, the design, conduct and analysis of this trial is focussed on issues that might have a material impact on the wellbeing and safety of study participants (in the community with suspected or confirmed SARS-CoV-2 infection) and the reliability of the results that would inform the care for future patients.

The critical factors that influence the ability to deliver these quality objectives are:

- to minimise the burden on thousands of busy clinicians working in an overstretched primary care setting and undertaking research during a major epidemic
- to ensure that suitable patients have access to the trial medication
- to provide information on the study to patients and clinicians in a timely and readily digestible fashion but without impacting adversely on other aspects of the trial or the patient's care
- to collect comprehensive information on the mortality and disease status

In assessing any risks to patient safety and well-being, a key principle is that of proportionality. Risks associated with participation in the trial must be considered in the context of usual care. At present, there are no proven treatments for COVID-19 for clinicians in the community to prescribe safely with a sound evidence base.

Although data entry should be mindful of the desire to maintain integrity and audit trails, in the circumstances of this epidemic, the priority is on the timely entry of data that is sufficient to support reliable analysis and interpretation about treatment effects.

The Investigators will maintain appropriate medical and research records for this trial, in compliance with the requirements of the Medicines for Human Use (Clinical Trial) Regulations 2004, ICH E6 GCP and regulatory and institutional requirements for the protection of confidentiality of volunteers. The Chief Investigator, Principal Investigator, Co-Investigators, clinical team, including Clinical Research Nurses, and other authorised members of the trial team will have access to records. The Investigators will permit authorised representatives of the sponsor, and regulatory agencies to examine (and when required by applicable law, to copy) clinical records for the purposes of quality assurance reviews, audits and evaluation of the study safety and progress. The software used for the trial is described in *supplementary material E*.

7 QUALITY ASSURANCE PROCEDURES

The study will be conducted in accordance with the current approved protocol, GCP, relevant regulations and PC-CTU Standard Operating Procedures. All PIs, coordinating centre staff and site staff will receive training in trial procedures according to GCP where required. Regular monitoring will be performed according to GCP using a risk-based approach. Data will be evaluated for compliance with the protocol and accuracy in relation to source documents where possible.

© Copyright: The University of Oxford and Oxford University Hospitals NHS Foundation Trust 2019

The PC-CTU Trial Management Group will be responsible for the monitoring of all aspects of the trial's conduct and progress and will ensure that the protocol is adhered to and that appropriate action is taken to safeguard participants and the quality of the trial itself. The TMG will be comprised of individuals responsible for the trial's day to day management and will meet regularly throughout the course of the trial.

7.1 Risk assessment and Monitoring

A risk assessment and monitoring plan will be prepared before the study opens and will be reviewed as necessary over the course of the study to reflect significant changes to the protocol or outcomes of monitoring activities. Monitoring will be performed by the PC-CTU Quality Assurance Manager or delegate. The level of monitoring required will be informed by the risk assessment.

7.2 Trial committees

The responsibilities of each group are as follows:

- Data Monitoring and Safety Committee (DMSC) - to review the data at each interim analysis as described in the Statistical Analysis section, as the updates to the randomisation scheme occur in order to ensure that the process is working correctly and to review and monitor the accruing data to ensure the rights, safety and wellbeing of the trial participants. Composition, and roles and responsibilities of the DMSC are detailed in the DMSC charter.
- Trial Steering Committee (TSC) - the Trial Steering Committee ensure the rights, safety and wellbeing of the trial participants. They will make recommendations about how the study is operating, any ethical or safety issues and any data being produced from other relevant studies that might impact the trial. Composition, and roles and responsibilities of the TSC are detailed in the TSC charter.
- Trial Management Group (TMG) - is responsible for the day-to-day running of the trial, including monitoring all aspects of the trial and ensuring that the protocol is being adhered to. It will include Co-Investigators and will meet weekly in the first instance. . Composition, and roles and responsibilities of the TMG are detailed in the TMG charter.
- A core project team (PT) from within the TMG will meet weekly or as required for operational decision making (met daily at the start of the trial).

8 PROTOCOL DEVIATIONS

A study related deviation is a departure from the ethically approved study protocol or other study document or process (e.g. consent process or administration of study intervention) or from Good Clinical Practice (GCP) or any applicable regulatory requirements. Any deviations from the protocol will be documented in a protocol deviation form and filed in the study master file.

A PC-CTU SOP is in place describing the procedure for identifying non-compliances, escalation to the central team and assessment of whether a non-compliance /deviation may be a potential Serious Breach.

9 SERIOUS BREACHES

© Copyright: The University of Oxford and Oxford University Hospitals NHS Foundation Trust 2019

A “serious breach” is a breach of the protocol or of the conditions or principles of Good Clinical Practice which is likely to affect to a significant degree –

(a) the safety or physical or mental integrity of the trial subjects; or
(b) the scientific value of the research.

In the event that a serious breach is suspected the Sponsor must be contacted within one working day. In collaboration with the CI, the serious breach will be reviewed by the Sponsor and, if appropriate, the Sponsor will report it to the approving REC committee and the relevant NHS host organisation within seven calendar days.

10 ETHICAL AND REGULATORY CONSIDERATIONS

10.1 Declaration of Helsinki

The Investigator will ensure that this study is conducted in accordance with the principles of the Declaration of Helsinki.

10.2 Guidelines for Good Clinical Practice

The Investigator will ensure that this trial is conducted in accordance with relevant regulations and with Good Clinical Practice.

10.3 Approvals

Following Sponsor approval, the protocol, informed consent form, participant information sheets and any proposed informing material will be submitted to an appropriate Research Ethics Committee (REC), regulatory authorities, and host institution(s) for written approval. The PI and coordinating centres for each country will ensure and confirm correct regulatory approvals are gained prior to recruitment.

The Investigator will submit and, where necessary, obtain approval from the above parties for all substantial amendments to the original approved documents.

10.4 Other Ethical Considerations

If a particular arm is deemed futile and dropped, no further participants will be randomised to this arm and anyone who is currently on this arm will be informed it has been dropped.
Once a particular intervention has been declared superior and effective, that will become the comparator arm (i.e. standard care).

Some participant’s, due to their co-morbidities, will be exempt from prescription charges. Medication is most often sent from PC-CTU and so prescription charges will not apply. All participants will receive a £20 voucher to cover any prescriptions and other expenses they may incur as a consequence of study participation.

Participants who lack capacity to consent for themselves will only be recruited after consultation with their legal representative. Any sign of dissent in any form from the participant who lacks consent will be taken as an indication they do not wish to be involved and they will be withdrawn.

© Copyright: The University of Oxford and Oxford University Hospitals NHS Foundation Trust 2019

Only residents of care homes who lack capacity to consent will be recruited, adults who lack capacity to consent will not be recruited from the wider community.

10.5 Reporting

The CI shall submit once a year throughout the clinical trial, or on request, an Annual Progress Report to the REC, HRA (where required), host organisation, funder (where required) and Sponsor. In addition, an End of Trial notification and final report will be submitted to the MHRA, the REC, host organisation and Sponsor.

10.6 Transparency in Research

Prior to the recruitment of the first participant, the trial will have been registered on a publicly accessible database. Results will be uploaded to the European Clinical Trial (EudraCT) Database within 12 months of the end of trial declaration by the CI or their delegate. Where the trial has been registered on multiple public platforms, the trial information will be kept up to date during the trial, and the CI or their delegate will upload results to all those public registries within 12 months of the end of the trial declaration.

10.7 Participant Confidentiality

The study will comply with the General Data Protection Regulation (GDPR) and Data Protection Act 2018, which require data to be de-identified as soon as it is practical to do so. The processing of the personal data of participants will be minimised by making use of a unique participant study number only on all study documents and any electronic database(s). All documents will be stored securely and only accessible by study staff and authorised personnel. The study staff will safeguard the privacy of participants' personal data.

10.8 Expenses and Benefits

All participants will be reimbursed with a £20 voucher, to cover the payment of a prescription, should they incur this as a result of study participation, and a token of recognition of giving their time and contribution to the study. A proportion of people with the co-morbidities outlined and in the over 50 age-range , are not required to pay for prescriptions. Furthermore, medication is most often sent from the PC-CTU, and so prescription charges will not apply. Participants who complete a telephone interview as part of the qualitative sub-study will be reimbursed with a (second) £20 voucher for their time to participate.

11 FINANCE AND INSURANCE

11.1 Funding

The study is funded by the UKRI/NIHR via an MRC call.
The Department of Health & Social Care have provided the following drugs free of charge for trial Use: Hydroxychloroquine, Favipiravir.

11.2 Insurance

The University has a specialist insurance policy in place, which would operate in the event of any participant suffering harm as a result of their involvement in the research (Newline Underwriting

© Copyright: The University of Oxford and Oxford University Hospitals NHS Foundation Trust 2019

Management Ltd, at Lloyd's of London). NHS indemnity operates in respect of the clinical treatment that is provided.

11.3 Contractual arrangements

Appropriate contractual arrangements will be put in place with all third parties.

12 PUBLICATION POLICY

The Investigators (those listed on the protocol and others to be decided at publication) will be involved in reviewing drafts of the manuscripts, abstracts, press releases and any other publications arising from the study. Authors will acknowledge the study funders. Authorship will be determined in accordance with the ICMJE guidelines and other contributors will be acknowledged.

13 DEVELOPMENT OF A NEW PRODUCT/ PROCESS OR THE GENERATION OF INTELLECTUAL PROPERTY

Ownership of IP generated by employees of the University vests in the University. The University will ensure appropriate arrangements are in place as regards any new IP arising from the trial.

14 ARCHIVING

Archiving will be done according to the UOXF PC-CTU SOP and study specific working instructions.

© Copyright: The University of Oxford and Oxford University Hospitals NHS Foundation Trust 2019

15 REFERENCES

1. Kim PS, Read SW, Fauci AS. Therapy for Early COVID-19: A Critical Need. JAMA. 2020;324(21):2149-50.
2. Livingston E, Bucher K. Coronavirus Disease 2019 (COVID-19) in Italy. JAMA. 2020.
3. Wu Z, McGoogan JM. Characteristics of and Important Lessons From the Coronavirus Disease 2019 (COVID-19) Outbreak in China: Summary of a Report of 72314 Cases From the Chinese Center for Disease Control and Prevention. JAMA. 2020.
4. Zhou F, Yu T, Du R, Fan G, Liu Y, Liu Z, et al. Clinical course and risk factors for mortality of adult inpatients with COVID-19 in Wuhan, China: a retrospective cohort study. Lancet. 2020.
5. Zheng Z, Peng F, Xu B, Zhao J, Liu H, Peng J, et al. Risk factors of critical & mortal COVID-19 cases: A systematic literature review and meta-analysis. J Infect. 2020;81(2):e16-e25.
6. Paules CI, Marston HD, Fauci AS. Coronavirus Infections-More Than Just the Common Cold. JAMA. 2020.
7. Topp CW, Ostergaard SD, Sondergaard S, Bech P. The WHO-5 Well-Being Index: a systematic review of the literature. Psychother Psychosom. 2015;84(3):167-76.
8. Neil M Ferguson DL, Gemma Nedjati-Gilani, Natsuko Imai, Kylie Ainslie, Marc Baguelin, Sangeeta Bhatia, Adhiratha Boonyasiri, Zulma Cucunubá, Gina Cuomo-Dannenburg, Amy Dighe, Ilaria Dorigatti, Han Fu, Katy Gaythorpe, Will Green, Arran Hamlet, Wes Hinsley, Lucy C Okell, Sabine van Elsland, Hayley Thompson, Robert Verity, Erik Volz, Haowei Wang, Yuanrong Wang, Patrick GT Walker, Caroline Walters, Peter Winskill, Charles Whittaker, Christl A Donnelly, Steven Riley, Azra C Ghani. Impact of non-pharmaceutical interventions (NPIs) to reduce COVID-19 mortality and healthcare demand 2020 [Available from: https://www.imperial.ac.uk/media/imperial-college/medicine/sph/ide/gida-fellowships/Imperial-College-COVID19-NPI-modelling-16-03-2020.pdf.
9. Wang M, Cao R, Zhang L, Yang X, Liu J, Xu M, et al. Remdesivir and chloroquine effectively inhibit the recently emerged novel coronavirus (2019-nCoV) in vitro. Cell Res. 2020;30(3):269-71.
10. Devaux CA, Rolain JM, Colson P, Raoult D. New insights on the antiviral effects of chloroquine against coronavirus: what to expect for COVID-19? Int J Antimicrob Agents. 2020:105938.
11. Colson P, Rolain JM, Lagier JC, Brouqui P, Raoult D. Chloroquine and hydroxychloroquine as available weapons to fight COVID-19. Int J Antimicrob Agents. 2020:105932.
12. Gao J, Tian Z, Yang X. Breakthrough: Chloroquine phosphate has shown apparent efficacy in treatment of COVID-19 associated pneumonia in clinical studies. Biosci Trends. 2020.
13. Colson P, Rolain JM, Raoult D. Chloroquine for the 2019 novel coronavirus SARS-CoV-2. Int J Antimicrob Agents. 2020;55(3):105923.
14. Mackenzie AH. Dose refinements in long-term therapy of rheumatoid arthritis with antimalarials. Am J Med. 1983;75(1A):40-5.
15. Yao X, Ye F, Zhang M, Cui C, Huang B, Niu P, et al. In Vitro Antiviral Activity and Projection of Optimized Dosing Design of Hydroxychloroquine for the Treatment of Severe Acute Respiratory Syndrome Coronavirus 2 (SARS-CoV-2). Clin Infect Dis. 2020.
16. Mahevas M, Tran VT, Roumier M, Chabrol A, Paule R, Guillaud C, et al. Clinical efficacy of hydroxychloroquine in patients with covid-19 pneumonia who require oxygen: observational comparative study using routine care data. BMJ. 2020;369:m1844.
17. Tang W, Cao Z, Han M, Wang Z, Chen J, Sun W, et al. Hydroxychloroquine in patients with mainly mild to moderate coronavirus disease 2019: open label, randomised controlled trial. BMJ. 2020;369:m1849.
18. Mehra MR, Desai SS, Ruschitzka F, Patel AN. Hydroxychloroquine or chloroquine with or without a macrolide for treatment of COVID-19: a multinational registry analysis. Lancet. 2020.
19. Geleris J, Sun Y, Platt J, Zucker J, Baldwin M, Hripcsak G, et al. Observational Study of Hydroxychloroquine in Hospitalized Patients with Covid-19. N Engl J Med. 2020.
20. Boulware DR, Pullen MF, Bangdiwala AS, Pastick KA, Lofgren SM, Okafor EC, et al. A Randomized Trial of Hydroxychloroquine as Postexposure Prophylaxis for Covid-19. N Engl J Med. 2020.

© Copyright: The University of Oxford and Oxford University Hospitals NHS Foundation Trust 2019

21. Chen J, Liu D, Liu L, Liu P, Xu Q, Xia L, et al. [A pilot study of hydroxychloroquine in treatment of patients with moderate COVID-19]. Zhejiang Da Xue Xue Bao Yi Xue Ban. 2020;49(2):215-9.
22. Gautret P, Lagier JC, Parola P, Hoang VT, Meddeb L, Mailhe M, et al. Hydroxychloroquine and azithromycin as a treatment of COVID-19: results of an open-label non-randomized clinical trial. Int J Antimicrob Agents. 2020:105949.
23. Retallack H, Di Lullo E, Arias C, Knopp KA, Laurie MT, Sandoval-Espinosa C, et al. Zika virus cell tropism in the developing human brain and inhibition by azithromycin. Proc Natl Acad Sci U S A. 2016;113(50):14408-13.
24. E Bosseboeuf MA, T Nhan, JJ de Pina, JM Rolain, D Raoult, et al. **Azithromycin inhibits the replication of Zika virus.** J Antivirals Antiretrovirals. 2018;10:6-11.
25. Madrid PB, Panchal RG, Warren TK, Shurtleff AC, Endsley AN, Green CE, et al. Evaluation of Ebola Virus Inhibitors for Drug Repurposing. ACS Infect Dis. 2015;1(7):317-26.
26. Bacharier LB, Guilbert TW, Mauger DT, Boehmer S, Beigelman A, Fitzpatrick AM, et al. Early Administration of Azithromycin and Prevention of Severe Lower Respiratory Tract Illnesses in Preschool Children With a History of Such Illnesses: A Randomized Clinical Trial. JAMA. 2015;314(19):2034-44.
27. Villarino AV, Kanno Y, O'Shea JJ. Mechanisms and consequences of Jak-STAT signaling in the immune system. Nat Immunol. 2017;18(4):374-84.
28. Hodge S, Hodge G, Holmes M, Jersmann H, Reynolds PN. Increased CD8 T-cell granzyme B in COPD is suppressed by treatment with low-dose azithromycin. Respirology. 2015;20(1):95-100.
29. Hodge S, Hodge G, Jersmann H, Matthews G, Ahern J, Holmes M, et al. Azithromycin improves macrophage phagocytic function and expression of mannose receptor in chronic obstructive pulmonary disease. Am J Respir Crit Care Med. 2008;178(2):139-48.
30. Hodge S, Reynolds PN. Low-dose azithromycin improves phagocytosis of bacteria by both alveolar and monocyte-derived macrophages in chronic obstructive pulmonary disease subjects. Respirology. 2012;17(5):802-7.
31. Baines KJ, Wright TK, Gibson PG, Powell H, Hansbro PM, Simpson JL. Azithromycin treatment modifies airway and blood gene expression networks in neutrophilic COPD. ERJ Open Res. 2018;4(4).
32. Mandell LA, Wunderink RG, Anzueto A, Bartlett JG, Campbell GD, Dean NC, et al. Infectious Diseases Society of America/American Thoracic Society consensus guidelines on the management of community-acquired pneumonia in adults. Clin Infect Dis. 2007;44 Suppl 2:S27-72.
33. Kawai S, Ochi M, Nakagawa T, Goto H. Antimicrobial therapy in community-acquired pneumonia among emergency patients in a university hospital in Japan. J Infect Chemother. 2004;10(6):352-8.
34. Gleason PP, Meehan TP, Fine JM, Galusha DH, Fine MJ. Associations between initial antimicrobial therapy and medical outcomes for hospitalized elderly patients with pneumonia. Arch Intern Med. 1999;159(21):2562-72.
35. Wu C, Liu Y, Yang Y, Zhang P, Zhong W, Wang Y, et al. Analysis of therapeutic targets for SARS-CoV-2 and discovery of potential drugs by computational methods. Acta Pharm Sin B. 2020.
36. Kim HS, Luo L, Pflugfelder SC, Li DQ. Doxycycline inhibits TGF-beta1-induced MMP-9 via Smad and MAPK pathways in human corneal epithelial cells. Invest Ophthalmol Vis Sci. 2005;46(3):840-8.
37. Clemens DL, Duryee MJ, Sarmiento C, Chiou A, McGowan JD, Hunter CD, et al. Novel Antioxidant Properties of Doxycycline. Int J Mol Sci. 2018;19(12).
38. Hsu AT, Barrett CD, DeBusk GM, Ellson CD, Gautam S, Talmor DS, et al. Kinetics and Role of Plasma Matrix Metalloproteinase-9 Expression in Acute Lung Injury and the Acute Respiratory Distress Syndrome. Shock. 2015;44(2):128-36.
39. NICE. COVID-19 rapid guideline: managing suspected or confirmed pneumonia in adults in the community. NICE; 2020.
40. Nicolau DV, Bafadhel M. Inhaled corticosteroids in virus pandemics: a treatment for COVID-19? Lancet Respir Med. 2020.
41. BTS/SIGN. BTS/SIGN British Guideline on the Management of Asthma. British Thoracic Society; 2019.
42. GINA. Global Strategy for Asthma Management and Prevention (2019 update). 2019.

© Copyright: The University of Oxford and Oxford University Hospitals NHS Foundation Trust 2019

43. Bloom CI, Elkin SL, Quint JK. Changes in COPD inhaler prescriptions in the United Kingdom, 2000 to 2016. Int J Chron Obstruct Pulmon Dis. 2019;14:279-87.
44. Artigas A, Camprubi-Rimblas M, Tantinya N, Bringue J, Guillamat-Prats R, Matthay MA. Inhalation therapies in acute respiratory distress syndrome. Ann Transl Med. 2017;5(14):293.
45. Ortiz-Diaz E, Li G, Kor D, Gajic O, Akca O, Adesanya A, et al. Preadmission Use of Inhaled Corticosteroids Is Associated With a Reduced Risk of Direct Acute Lung Injury/Acute Respiratory Distress Syndrome. CHEST. 2011;140(4):912A.
46. Festic E, Carr GE, Cartin-Ceba R, Hinds RF, Banner-Goodspeed V, Bansal V, et al. Randomized Clinical Trial of a Combination of an Inhaled Corticosteroid and Beta Agonist in Patients at Risk of Developing the Acute Respiratory Distress Syndrome. Crit Care Med. 2017;45(5):798-805.
47. Yamaya M, Nishimura H, Deng X, Sugawara M, Watanabe O, Nomura K, et al. Inhibitory effects of glycopyrronium, formoterol, and budesonide on coronavirus HCoV-229E replication and cytokine production by primary cultures of human nasal and tracheal epithelial cells. Respir Investig. 2020;58(3):155-68.
48. Merad M, Martin JC. Pathological inflammation in patients with COVID-19: a key role for monocytes and macrophages. Nature Reviews Immunology. 2020;20(6):355-62.
49. Barnes PJ. Inhaled Corticosteroids. Pharmaceuticals (Basel). 2010;3(3):514-40.
50. Peters MC, Sajuthi S, Deford P, Christenson S, Rios CL, Montgomery MT, et al. COVID-19 Related Genes in Sputum Cells in Asthma: Relationship to Demographic Features and Corticosteroids. Am J Respir Crit Care Med. 2020.
51. Inhaled corticosteroids downregulate the SARS-CoV-2 receptor ACE2 in COPD through suppression of type I interferon - preliminary report. 13 June 2020 (pre-print).
52. Lan J, Ge J, Yu J, Shan S, Zhou H, Fan S, et al. Structure of the SARS-CoV-2 spike receptor-binding domain bound to the ACE2 receptor. Nature. 2020;581(7807):215-20.
53. Hoffmann M, Kleine-Weber H, Schroeder S, Krüger N, Herrler T, Erichsen S, et al. SARS-CoV-2 Cell Entry Depends on ACE2 and TMPRSS2 and Is Blocked by a Clinically Proven Protease Inhibitor. Cell. 2020;181(2):271-80.e8.
54. Matsuyama S, Kawase M, Nao N, Shirato K, Ujike M, Kamitani W, et al. The inhaled corticosteroid ciclesonide blocks coronavirus RNA replication by targeting viral NSP15. bioRxiv. 2020:2020.03.11.987016.
55. Yamaya M, Nishimura H, Deng X, Sugawara M, Watanabe O, Nomura K, et al. Inhibitory effects of glycopyrronium, formoterol, and budesonide on coronavirus HCoV-229E replication and cytokine production by primary cultures of human nasal and tracheal epithelial cells. Respir Investig. 2020.
56. Reyes AZ, Hu KA, Teperman J, Wampler Muskardin TL, Tardif J-C, Shah B, et al. Anti-inflammatory therapy for COVID-19 infection: the case for colchicine. Annals of the Rheumatic Diseases. 2020:annrheumdis-2020-219174.
57. Rodriguez-Nava G, Trelles-Garcia DP, Yanez-Bello MA, Chung CW, Trelles-Garcia VP, Friedman HJ. Atorvastatin associated with decreased hazard for death in COVID-19 patients admitted to an ICU: a retrospective cohort study. Crit Care. 2020;24(1):429.
58. Deftereos SG, Giannopoulos G, Vrachatis DA, Siasos GD, Giotaki SG, Gargalianos P, et al. Effect of Colchicine vs Standard Care on Cardiac and Inflammatory Biomarkers and Clinical Outcomes in Patients Hospitalized With Coronavirus Disease 2019: The GRECCO-19 Randomized Clinical Trial. JAMA Netw Open. 2020;3(6):e2013136.
59. Scarsi M, Piantoni S, Colombo E, Airo P, Richini D, Miclini M, et al. Association between treatment with colchicine and improved survival in a single-centre cohort of adult hospitalised patients with COVID-19 pneumonia and acute respiratory distress syndrome. Ann Rheum Dis. 2020;79(10):1286-9.
60. Lopes MIea. Beneficial effects of colchicine for moderate to severe COVID-19: an interim analysis of a randomized, double-blinded, placebo controlled clinical trial. medRxiv preprint. 2020.
61. Chiu L, Chow R, Chiu N, Lo C-H, Aggarwal R, Lee J, et al. Colchicine use in patients with COVID-19: a systematic review and meta-analysis. medRxiv. 2021:2021.02.02.21250960.
62. Tardif J-C, Bouabdallaoui N, L'Allier PL, Gaudet D, Shah B, Pillinger MH, et al. Efficacy of Colchicine in Non-Hospitalized Patients with COVID-19. medRxiv. 2021:2021.01.26.21250494.

© Copyright: The University of Oxford and Oxford University Hospitals NHS Foundation Trust 2019

63. Piantoni S, Colombo E, Airò P, Tincani A, Brucato A, Franceschini F, et al. The rationale for the use of colchicine in COVID-19: comments on the letter by Cumhur Cure M et al. Clin Rheumatol. 2020;39(8):2489-90.
64. Piantoni S, Colombo E, Furloni R, Andreoli L, Brucato A, Imazio M, et al. Anti-inflammatory action of colchicine in hospitalised patients with COVID-19. Response to: 'Colchicine treatment in community healthcare setting to prevent severe COVID-19' by Della-Torre et al. Annals of the Rheumatic Diseases. 2020:annrheumdis-2020-218806.
65. Piantoni S, Patroni A, Toniati P, Furloni R, Franceschini F, Andreoli L, et al. Why not to use colchicine in COVID-19? An oldanti-inflammatory drug for a novel auto-inflammatory disease. Rheumatology. 2020;59(7):1769-70.
66. Prieto-Pena D, Dasgupta B. Biologic agents and small-molecule inhibitors in systemic autoimmune conditions: an update. Pol Arch Intern Med. 2020.
67. Dalbeth N, Lauterio TJ, Wolfe HR. Mechanism of action of colchicine in the treatment of gout. Clin Ther. 2014;36(10):1465-79.
68. Rodrigues TS, de Sá KSG, Ishimoto AY, Becerra A, Oliveira S, Almeida L, et al. Inflammasomes are activated in response to SARS-CoV-2 infection and are associated with COVID-19 severity in patients. J Exp Med. 2021;218(3).
69. Stewart S, Yang KCK, Atkins K, Dalbeth N, Robinson PC. Adverse events during oral colchicine use: a systematic review and meta-analysis of randomised controlled trials. Arthritis Research & Therapy. 2020;22(1):28.
70. Levy M, Spino M, Read SE. Colchicine: a state-of-the-art review. Pharmacotherapy. 1991;11(3):196-211.
71. Chen C, Zhang Y, Huang J, Yin P, Cheng Z, Wu J, et al. Favipiravir versus Arbidol for COVID-19: A Randomized Clinical Trial. medRxiv. 2020:2020.03.17.20037432.
72. Udwadia ZF, Singh P, Barkate H, Patil S, Rangwala S, Pendse A, et al. Efficacy and Safety of Favipiravir, an Oral RNA-Dependent RNA Polymerase Inhibitor, in Mild-to-Moderate COVID-19: A Randomized, Comparative, Open-Label, Multicenter, Phase 3 Clinical Trial. Int J Infect Dis. 2020.
73. Ivashchenko AA, Dmitriev KA, Vostokova NV, Azarova VN, Blinow AA, Egorova AN, et al. AVIFAVIR for Treatment of Patients With Moderate Coronavirus Disease 2019 (COVID-19): Interim Results of a Phase II/III Multicenter Randomized Clinical Trial. Clinical Infectious Diseases. 2020.
74. Khamis F, Al Naabi H, Al Lawati A, Ambusaidi Z, Al Sharji M, Al Barwani U, et al. Randomized controlled open label trial on the use of favipiravir combined with inhaled interferon beta-1b in hospitalized patients with moderate to severe COVID-19 pneumonia. International Journal of Infectious Diseases. 2021;102:538-43.
75. Cai Q, Yang M, Liu D, Chen J, Shu D, Xia J, et al. Experimental Treatment with Favipiravir for COVID-19: An Open-Label Control Study. Engineering. 2020;6(10):1192-8.
76. Pilkington V, Pepperrell T, Hill A. A review of the safety of favipiravir – a potential treatment in the COVID-19 pandemic? Journal of Virus Eradication. 2020;6(2):45-51.
77. Sanders JM, Monogue ML, Jodlowski TZ, Cutrell JB. Pharmacologic Treatments for Coronavirus Disease 2019 (COVID-19): A Review. JAMA. 2020;323(18):1824-36.
78. Coomes EA, Haghbayan H. Favipiravir, an antiviral for COVID-19? Journal of Antimicrobial Chemotherapy. 2020;75(7):2013-4.
79. Arshad U, Pertinez H, Box H, Tatham L, Rajoli RK, Curley P, et al. Prioritisation of potential anti-SARS-CoV-2 drug repurposing opportunities based on ability to achieve adequate plasma and target site concentrations derived from their established human pharmacokinetics. medRxiv. 2020:2020.04.16.20068379.
80. Kaptein SJF, Jacobs S, Langendries L, Seldeslachts L, ter Horst S, Liesenborghs L, et al. Favipiravir at high doses has potent antiviral activity in SARS-CoV-2−infected hamsters, whereas hydroxychloroquine lacks activity. Proceedings of the National Academy of Sciences. 2020;117(43):26955-65.
81. Driouich J-S, Cochin M, Lingas G, Moureau G, Touret F, Petit PR, et al. Favipiravir antiviral efficacy against SARS-CoV-2 in a hamster model. bioRxiv. 2020:2020.07.07.191775.

© Copyright: The University of Oxford and Oxford University Hospitals NHS Foundation Trust 2019

82. Cevik M, Tate M, Lloyd O, Maraolo AE, Schafers J, Ho A. SARS-CoV-2, SARS-CoV, and MERS-CoV viral load dynamics, duration of viral shedding, and infectiousness: a systematic review and meta-analysis. The Lancet Microbe.
83. CDC. https://www.cdc.gov.tw/File/Get/ht8jUiB_MI-aKnlwstwzvw.
84. Sissoko D, Laouenan C, Folkesson E, M'Lebing AB, Beavogui AH, Baize S, et al. Experimental Treatment with Favipiravir for Ebola Virus Disease (the JIKI Trial): A Historically Controlled, Single-Arm Proof-of-Concept Trial in Guinea. PLoS Med. 2016;13(3):e1001967.

© Copyright: The University of Oxford and Oxford University Hospitals NHS Foundation Trust 2019

16 APPENDIX A: SCHEDULE OF PROCEDURES

Procedures	Participant contacts							
	Visit timing Day 0	Day 0	Day 0	Day 0	Daily Day 1-28 incl	Day 28-12 months (monthly contact)	Day 29-12mths	Up to 10 years
	Screening completed by participant online/phone	Eligibility completed by participant online/phone	Baseline completed by participant online/phone	Eligibility completed by Clinician online/phone	Symptom Diaries completed by participant online/phone	Contacted by study team if consent provided	Retrospective data collection by study team	By data extraction from clinical records
Informed consent	X	X	X	X	X			
Demographics	X	X	X				X	
Medical history	X	X	X	X			X	
Swab as part of the RCGP RSC/PHE national surveillance programme	When available, preferably by self-swabbing at study entry							
Concomitant medications		X					X	
Eligibility assessment	X	X						

© Copyright: The University of Oxford and Oxford University Hospitals NHS Foundation Trust 2019

Randomisation				X				
Dispensing of trial drugs				X	X			
Questionnaire					X	X		
WHO 5 Well Being Index	X				Day 14 and day 28	X		
Telephone interview (for subset of patient participants)					X			
Compliance					X			
Adverse event assessments					X*		X	
Optional SARS-CoV-2 blood test as part of the RCGP RSC/PHE national surveillance programme							X	
Evidence of sequalae and health care utilisation						X		X

© Copyright: The University of Oxford and Oxford University Hospitals NHS Foundation Trust 2019

* Patient reported AEs will be assessed by a clinician for certain IMPs, as specified in the Intervention Specific Appendices. These treatments include HCQ (no longer an active treatment arm) and drugs that are not licensed for use in the UK.

© Copyright: The University of Oxford and Oxford University Hospitals NHS Foundation Trust 2019

17 APPENDIX B: AMENDMENT HISTORY

Amendment No.	Protocol Version No.	Date issued	Author(s) of changes	Details of Changes made
1 (SA1)	1.1		Emma Ogburn; Chris Butler; Gail Hayward	Inclusion criteria: change 'known heart disease' to 'Known heart disease and/or hypertension'; Exclusion criteria: exclude patients taking the following drugs: penicillamine, amiodarone, ciclosporin, chloroquine. Update section 9.6 to include vision changes and lowering of blood sugar. Update change in Funder and update Investigator list to reflect UKRI funder bid.
2 (SA2)	2.0		Emma Ogburn; Chris Butler; Gail Hayward, Hannah Swayze	Inclusion of TSC; central facility to distribute patient packs; addition of third arm; update of secondary outcomes to include WHO wellbeing questions; qualitative sub study; sign posting to other RCGP RSC study; eligibility confirmation by research nurse.
3 (SA3)	2.1		Hannah Swayze; Chris Butler; Emma Ogburn; Gail Hayward	Trial rationale; secondary outcomes to include blood test; 14 days of covid-19 symptoms; call to participant at day 2; poster
4 (SA4)	*2.1*		*No changes to the protocol*	
5 (SA5)	3.0		Hannah Swayze; Chris Butler; Emma Ogburn; Gail Hayward	Updated Azithromycin information; broadening of inclusion criteria; first interim analysis; primary analysis details; care home materials; administrative and typographical updates; study partner letter; recruitment via social media, care homes and pharmacies; GPs prescribe trial medication; eligibility to at least one intervention arm as well as the

© Copyright: The University of Oxford and Oxford University Hospitals NHS Foundation Trust 2019

				Usual Care arm; ICF may be sent to participants.
6 (SA6)	4.0		Chris Butler; Emma Ogburn; Gail Hayward; Ben Saville; Ly-Mee Yu; Hannah Swayze	Updating inclusion criteria; updating the rationale and evidence for safety of hydroxychloroquine; inclusion of a new arm, doxycycline; AE reporting for hydroxychloroquine arm; typographical clarifications.
7 (NS1)	4.0		*No changes to the protocol*	
8 (SA7)	5.0		Chris Butler; Emma Ogburn; Ben Saville; Ly-Mee Yu; Hannah Swayze	Including a second primary outcome, time to recovery, change to sample size estimation, new eligibility criteria: obesity, formatting changes, blood test process.
9 (SA8)	5.0		*No changes to the protocol*	
10 (SA9)	5.0		*No changes to the protocol*	
11 (NS2)	5.0		*No changes to the protocol*	
12 (SA10)	6.0		Chris Butler; Emma Ogburn; Hannah Swayze	Addition of inhaled corticosteroid treatment arm, enrolment to additional trials, long-term follow-up, access to NHS Digital Pillar 2 test data, removal of investigators, additional trial contact with participants for up to 12 months, changes to objectives/outcomes/ time-points, removal of sampling from study
13 (NS3)	6.1		Sharon Tonner	Removal of patient already taking a treatment arm medication as an exclusion
14 (NS4)	6.1		*No changes to the protocol*	
15 (SA11)	6.2		Sharon Tonner, Hannah Swayze	Inclusion of patients who lack capacity to consent, discontinuation of azithromycin arm
16 (SA12)	7.0		Chris Butler; Emma Ogburn; Hannah Swayze;	Addition of colchicine treatment arm. Data management proportional approach. Discontinuation of doxycycline

© Copyright: The University of Oxford and Oxford University Hospitals NHS Foundation Trust 2019

			Emily Bongard; Julie Allen; Jienchi Dorward; Oliver Van Hecke	arm. Stylistic and typographical updates. Funding arrangements. Inclusion criteria - to include those aged 18 or over. Rephrasing secondary outcome: daily rating of how well participant feels. Statistical analysis.
17 (SA13)	7.1		Chris Butler; Emma Ogburn; Hannah Swayze; Emily Bongard; Ly-Mee Yu; Jienchi Dorward	Addition of safety monitoring procedures for drugs that are not licensed in the UK. Addition of safety as a secondary endpoint. Addition of favipiravir treatment arm. Stylistic and typographical updates. GP Contact for safety monitoring. In-kind contributions. Using JDR as a recruitment tool. The primary analysis population defined as those with a COVID-19 positive test. Drug accountability for favipiravir.

Lists details of all protocol amendments whenever a new version of the protocol is produced.

Protocol amendments must be submitted to the Sponsor for approval prior to submission to the REC committee, HRA (where required) or MHRA.

© Copyright: The University of Oxford and Oxford University Hospitals NHS Foundation Trust 2019

18 APPENDIX C: USUAL CARE ARM

1. Background and rationale

COVID-19 disproportionately affects people with comorbidities, more severe illness, and who are older. The disease causes considerable morbidity and mortality in this population group in particular, and is having a devastating effect on people's health, and society in the UK and internationally.(2, 3, 6, 8) So far, there are no specific treatments for COVID-19 that have been proven in rigorous clinical trials to be effective and that can be used in the community. Clinicians managing possible COVID-19 in the community will make clinical judgements about best treatment based on the clinical situation, but care is usually supportive to begin with, unless patients deteriorate and require hospital admission https://www.nice.org.uk/guidance/ng163). The National Institute for Health and Care Excellence does not recommend the immediate use of antibiotics unless there are signs of pneumonia (https://www.nice.org.uk/guidance/ng163).

This Usual Care arm will follow current NHS care provision, and provides a control against which the effect of new interventions that are added to usual care can be assessed. If a new trial intervention plus usual care is found to be superior to usual care alone, then the usual care alone arm will be dropped, and the intervention that is found to be most effective will become the standard of care within the trial.

2. Changes to outcome measures

None

3. Detail of intervention

Participants randomised to the usual care arm will receive usual clinical care as per NHS care delivery practice.

a. Investigational Medicinal Product (IMP) description

Not applicable

b. Storage of IMP

Not applicable

4. Safety reporting

Mechanisms for safety reporting are outlined in the trial protocol.

© Copyright: The University of Oxford and Oxford University Hospitals NHS Foundation Trust 2019

19 APPENDIX D: USUAL CARE PLUS HYDROXYCHLOROQUINE ARM (DISCONTINUED)

1. Background and rationale

a. Evidence for potential Hydroxychloroquine benefits in COVID-19

A candidate intervention for COVID-19, a drug called hydroxychloroquine, has become available following early evaluation in some studies in China.(9, 10) Hydroxychloroquine is a hydroxylated version of the drug chloroquine.(10, 11) Both agents are commonly in use as anti-malarials, and are used in a variety of auto-immune diseases. They have received significant recent interest as potential modifiers of disease activity in COVID-19. (10, 12, 13) Hydroxychloroquine is already available within the NHS on prescription for other indications, and has a generally benign safety profile.(14) Chloroquine is available to buy in the UK over the counter in some formulations and is used as antimalarial prophylaxis and treatment.

Chloroquine is known to block virus infection by increasing endosomal pH required for virus/cell fusion, as well as interfering with the glycosylation of cellular receptors of SARS-CoV-2.(5) Besides its antiviral activity, chloroquine has an immune-modulating activity, which may synergistically enhance its antiviral effect *in vivo*.(11) Chloroquine is widely distributed in the whole body, including lungs, after oral administration.(10) The EC_{90} value of chloroquine against the 2019-nCoV in Vero E6 cells was 6.90 μM in one study (9) which can be clinically achievable as demonstrated in the plasma of rheumatoid arthritis patients who received 500 mg administration.(14)

Hydroxychloroquine has been found to be effective against intracellular micro-organisms including malaria and intracellular bacteria *Coxiella burnetii* and *Tropheryma Whipplei*.(11) Both chloroquine and hydroxychloroquine have been shown to have *in vitro* antiviral activity against SARS coronavirus in a number of studies.(11) Most recently activity against SARSCOV2 was shown to be greater for hydroxychloroquine than chloroquine (15).

Key publications that have relevance to the safety and rationale for use of hydroxychloroquine in the PRINCIPLE Trial:

1. *The Mahévas study* was an observational study that assessed whether hydroxychloroquine reduced the need for transfer to ICU in patients already sick enough to be hospitalised.(16) It focussed on sicker patients with hypoxic pneumonia, some requiring ITU care. It did not find a difference in transfers to ICU. So the question and population in the Mahevas study are very different compared to PRINCIPLE. Most importantly, unlike PRINCIPLE, the Mahevas study is ***not a randomised clinical tri***al. Numbers were relatively small (n=181), and it is at high risk of bias due to the observational design.

Regarding safety, those receiving hydroxychloroquine were prescribed 600mg per day, whereas the dose in the PRINCIPLE trial is 400mg per day; 18% of those who received hydroxychloroquine in the Mahévas study were also on azithromycin (which can be arrhythmogenic), and this combination is not possible in PRINCIPLE because of the additive risk. Moreover, PRINCIPLE excludes several other drug combinations that could be arrhythmogenic. In the Mahevas study, eight patients (10%) who were taking hydroxychloroquine experienced electrocardiographic changes that required discontinuation of hydroxychloroquine. Critically, those in the control

© Copyright: The University of Oxford and Oxford University Hospitals NHS Foundation Trust 2019

group did not have ECGs done, so we don't know if there was indeed a difference between groups, and we cannot therefore attribute the ECG changes to hydroxychloroquine. COVID-19 itself, or drug interactions, may well have been underlying reasons. The authors state, "Although hydroxychloroquine is considered safe in the context of systemic lupus erythematosus, these adverse events might be explained by the use of high dose hydroxychloroquine in patients older than 75 years with renal impairment and frequent drug interactions. We cannot rule out the possibility that these cardiac effects attributed to hydroxychloroquine were caused by COVID-19, especially given electrocardiograms were unavailable during follow-up in the control group."

2. *The Tang study* was a hospital-based, randomised study and included 150 patients; randomisation was done using sealed envelopes.(17) The trial found no difference in the proportion of patients with two sequential negative swab results.

Regarding safety,75 participants received hydroxychloroquine 1200 mg daily for 3 days and then 800 mg for either 2 or 3 weeks. Again, the dose used in this study was much higher that the dose being used in PRINCIPLE (initially three times, and subsequently twice as high as PRINCIPLE). However, 63% and 64% of patients in the hydroxychloroquine and control groups respectively also received other antiviral agents. In PRINCIPLE, we are not evaluating the combination of antiviral agents and hydroxychloroquine. Importantly, this study did not find evidence of cardiac arrhythmias associated with hydroxychloroquine use. The authors sate, "Events of cardiac arrhythmia, such as prolonged QT interval were not observed in our trial, possibly because of the relatively mild to moderate disease of patients investigated or the short term period of follow-up."

3. *The Mehra study* published in the Lancet on 22.05.2020 reported an association between hydroxychloroquine use and cardiac events and mortality amongst patients hospitalised with COVID-19.(18) The *observational* study design is inherently susceptible to bias, the study data integrity has been queried given the homogeneity of the baseline characteristics, the adequacy of the adjustment for confounders cannot be assessed from the published methods, and the registries used are in a different patient population compared to PRINCIPLE. Patients were much sicker and more advanced in the illness than in PRINCIPLE. The authors themselves state that "*Randomised clinical trials will be required before any conclusion can be reached regarding benefit or harm of these agents (hydroxychloroquine and chloroquine) in COVID-19 patients*." The authors also state "These data do not apply to the use of any treatment regimen used in the ambulatory, out-of-hospital setting." This study has proved hugely controversial on social media, with a number of methodological and data integrity concerns already raised, for example:

1. There were inadequate adjustments for known and measured confounders (disease severity, temporal effects, site effects, dose used).
2. The authors have not adhered to standard practices in the machine learning and statistics community. They have not released their code or data. There is no data/code sharing and availability statement in the paper. The Lancet was among the many signatories on the Wellcome statement on data sharing for COVID 19 studies.
3. There was no ethics review.
4. There was no mention of the countries or hospitals that contributed to the data source, no acknowledgments to their contributions. A request to the authors for information on the contributing centres was denied.
5. Data from Australia are not compatible with government reports (too many cases for

© Copyright: The University of Oxford and Oxford University Hospitals NHS Foundation Trust 2019

just five hospitals, more in-hospital deaths than had occurred in the entire country during the study period). Surgisphere (the data company) have since claimed this was an error of classification.

6. Data from Africa indicate over 40% of all COVID-19 cases and deaths in the continent occurred in *Surgisphere*-associated hospitals which had sophisticated electronic patient data recording, and patient monitoring able to detect and record "non-sustained [at least 6 secs] or sustained ventricular tachycardia or ventricular fibrillation". This seems unlikely.
7. Unusually small reported variances in baseline variables, interventions and outcomes between continents
8. Mean daily doses of hydroxychloroquine that are 100 mg higher than FDA recommendations, whilst 66% of the data are from North American hospitals.
9. Implausible ratios of chloroquine to hydroxychloroquine use in some continents.
10. The tight 95% confidence intervals reported for the hazard ratios are unlikely. For instance, for the Australian data this would need about double the numbers of recorded deaths that were reported in the paper.

This paper has now been retracted, and the data cannot be verified.

4. *The Geleris study* was an observational study of 1,376 consecutive COVID-19 patients at a New York hospital to determine whether hydroxychloroquine use was associated with intubation or death, as a primary composite outcome.(19) 811 (58.9%) of these patients received hydroxychloroquine. The authors excluded patients who were intubated, died, or who were transferred to another facility within 24 hours after presentation to the emergency department from the analyses. A propensity score matching model (C-statistic of 0.81) was used to ensure that groups were similar at baseline.

Regarding safety, multivariable adjusted analyses with inverse probability weighting revealed no significant association between treatment with hydroxychloroquine and intubation or death (HR 1.04 (95% CI 0.82 – 1.32)). Whilst the patient population in this study is different to that of PRINCIPLE, it is interesting that the findings contrast with those of a recent Lancet study published by Mehra *et al*. One possible reason for the difference is that patients receiving interventions like hydroxychloroquine in the study by Mehra *et al* were sicker than those in the study's control group. This may have arisen through use of crude measures to account for baseline disease severity (qSOFA score and SpO2 < 94%) in their propensity score matching model, and may also explain the big differences seen in patients requiring mechanical ventilation between controls (7.7%) and those in intervention groups (20-21.6%).

5. *Boulware* and colleagues conducted a Covid-19 postexposure prophylaxis, placebo controlled randomised trial of hydroxychloroquine in 821 asymptomatic patients; 11.8% of those taking hydroxychloroquine vs 14.3 of those taking placebo experienced a new illness compatible with COVID-19 (absolute difference -2.4%) but this difference was not statistically significant, indicating no evidence of benefit from the hydroxychloroquine. (20)

Regarding safety, while side effects were more common with hydroxychloroquine than with placebo (40.1% vs. 16.8%), no serious adverse reactions were reported.

© Copyright: The University of Oxford and Oxford University Hospitals NHS Foundation Trust 2019

Earlier studies of hydroxychloroquine for COVID-19

1. *Chen* and colleagues conducted a *randomised* controlled trial to test the effectiveness of hydroxychloroquine in 30 adult patients who tested positive for COVID-19 in China.(21) Patients in the treatment group received 400mg of hydroxychloroquine for 5 days, while the control group received usual care. The result of a nasopharyngeal swab on Day 7 was used as the primary outcome. The intention- to- treat analysis revealed that the treatment group did not differ from the control group in the number of patients testing negative for COVID-19 on Day 7 (13 versus 14 patients), nor the duration of illness (all P>0.05).

Regarding safety, the authors report three adverse events in the control group (one patient with abnormal liver function and anaemia, and one patient with abnormal renal function), and four adverse events in the treatment group (two patients with diarrhoea, one with lethargy, and one patient with abnormal liver function tests), which the authors argue were not linked to treatment with HCQ. One patient in the treatment group deteriorated significantly and thus HCQ was stopped on Day 4 of the treatment. This study was under-powered according to their own calculations.

2. Gautret and colleagues presented the results of an open- label, non-randomised trial with 36 patients diagnosed with COVID-19 in French hospitals.(22) Six participants were asymptomatic, 22 had upper respiratory tract infection symptoms, and eight had lower respiratory tract infection symptoms. The twenty patients in the treatment group received HCQ 200mg three times a day for 10 days. Patients declining to take part in the study and not meeting the inclusion criteria were assigned to the control group and received usual care. Six of the patients in the treatment group additionally received azithromycin to prevent bacterial superinfection. The primary outcome was SARS- CoV-2 carriage at Day 6 on nasopharyngeal swabs. Patients treated with hydroxychloroquine were significantly more likely to test negative for SARS- CoV-2 on Day 6 compared with controls (70% versus 12.5% virologically cured, p<0.001). All patients treated with hydroxychloroquine and azithromycin tested negative on Day 6.

Regarding safety, the authors did not report any safety data, stating that this would follow in a subsequent publication. Aside from a lack of adverse event reporting, there are many problems with the study methodology including the non-randomized design, under-powered sample size, lack of intention-to-treat analysis, and absence of medium to long-term follow-up data.

3. Chen and colleagues conducted a *randomised* clinical trial of adult patients admitted to hospital with confirmed COVID-19.(7) Sixty two patients were randomly assigned to usual care (n=31) or hydroxychloroquine (200 mg BD) for five days in addition to usual care (n=31). The authors report that there were ‘significant differences’ in time to clinical recovery (TTCR) between the two groups, with TTCR defined as the return of body temperature and cough relief, maintained for more than 72 hours. They also report that all four patients who ‘progressed to severe disease’ were in the control group. The reporting of empirical data by the authors is limited and unclear. They did not include a power calculation, but presumably this study was under-powered to detect differences between groups. No medium to long-term follow-up data is presented.

Regarding safety, the authors report that two mild adverse events occurred (a rash and a headache), both of which were in patients receiving hydroxychloroquine. No patients receiving usual care experienced adverse events.

© Copyright: The University of Oxford and Oxford University Hospitals NHS Foundation Trust 2019

In summary

The large scale hospital based Recovery trial has recently announced that they found no benefit from hydroxychloroquine (as yet unpublished). No safety concerns have been reported by the Principle Trial. A post exposure prophylaxis study found no benefit from hydroxychloroquine, but also found no safety concerns. These studies address a different research question and focus on different patient populations in comparison to the Principle Trial. Evidence about early treatment of COPVID-19 in the community is urgently needed: the potential application of the findings of the PRINCIPLE Trial of community treatment is considerable, and the 'reach' of the study is now nation-wide. Our study population are patients in the community and our trial question is about early treatment. Outcome data from studies with sicker hospitalised patients may not apply to our study population

A key, controversial observational study (Mehra et al) reported that those taking hydroxychloroquine had worse outcomes and suffered more cardiac events than those not taking hydroxychloroquine. However, major doubts have been expressed about the data integrity of this study and insufficient detail in the paper to judge the adequacy of the methods employed to adjust for the inevitable confounders in an observational study. Hydroxychloroquine is not a licensed drug for treating COVID-19. Patients doing well are therefore less likely to be prescribed this drug. When a patient is causing their clinical team more concern or their condition is deteriorating, the chances of them being prescribed hydroxychloroquine will be greater. Adjustment for potential confounders has been inadequate in the observational studies. Critically, these studies cannot adjust for the clinician's sense of how the patient is faring over time. The Mehra study has been retracted and can't be relied upon.

The deficiencies and differences in all of these studies highlight the need for well-conducted, adequately powered randomised clinical trials, to provide definitive evidence of the safety and effectiveness of hydroxychloroquine for the early community treatment COVID-19 illness. PRINCIPLE will assess whether hydroxychloroquine is safe and effective if given earlier in the course of illness and in patients with milder symptoms not requiring hospital admission.

2. Eligibility criteria specifically related to hydroxychloroquine

Inclusion criteria:

Exclusion criteria:

- Pregnancy;
- Breastfeeding;
- Known severe hepatic impairment;
- Known severe renal impairment;
- Known porphyria;
- Type 1 diabetes or insulin dependent Type 2 Diabetes mellitus ;
- Known G6PD deficiency;
- Known myasthenia gravis;
- Known severe psoriasis;
- Known severe neurological disorders (especially those with a history of epilepsy—may lower seizure threshold)
- Previous adverse reaction to, or currently taking, hydroxychloroquine or chloroquine

© Copyright: The University of Oxford and Oxford University Hospitals NHS Foundation Trust 2019

Patients currently taking the following drugs: penicillamine, amiodarone, ciclosporin, digoxin: the following antimicrobials; azithromycin, clarithromycin, erythromycin, ciprofloxacin, levofloxacin, moxifloxacin, ketoconazole, itraconazole, or mefloquine: the following antidepressants; amitriptyline, citalopram, desipramine, escitalopram, imipramine, doxepin, fluoxetine, wellbutrin, venlafaxine; the following antipsychotics or mood stabilizers; haloperidol, droperidol, lithium, quetiapine, thioridazine, ziprasidone: methadone: sumatriptan, zolmitriptan

- Known congenital or documented QT prolongation
- Known retinal disease

3. Outcome measures related to hydroxychloroquine

There are no outcome measures related specifically to this usual care plus hydroxychloroquine arm

4. Detail of intervention

Participants randomised to the usual care plus hydroxychloroquine arm will receive usual clinical care as per NHS guidelines, plus a course of oral hydroxychloroquine 200mg twice daily for seven days.

a. Investigational Medicinal Product (IMP) description

Hydroxychloroquine sulphate 200 milligram (mg) tablets. The tablets are for oral administration. One tablet (200mg) hydroxychloroquine to be taken twice daily for 7 days by mouth (14 tablets in total).
Special instructions: Each dose should be taken with a meal or glass of milk. Antacids may reduce absorption of hydroxychloroquine so it is advised that a 4-hour interval be observed between taking hydroxychloroquine and an antacid.
This is the standard therapeutic dose for its normal indication in adults which is for the treatment of rheumatoid arthritis, discoid and systemic lupus erythematosus, and dermatological conditions caused or aggravated by sunlight.

The Marketing Authorisation holder is Zentiva Pharma UK Limited Guildford Surrey GU1 4YS United Kingdom. Marketing authorisation number is PL 17780/0748.

b. Storage of IMP

: Stored at room temperature in locked cupboards in restricted access rooms in the Nuffield Department of Primary Care Health Sciences; in locked cupboards in restricted access rooms in GP Practices; in Pharmacies.

For hydroxychloroquine, GP practices can order a supply of trial medication from Public Health England using the existing ImmForm process. GPs will be provided with an envelope by the trial team which will be labelled appropriately for trial medication, and they will add the patient's details to this label. This pack, containing instructions on using the medication will be provided to the patient or their representative.

© Copyright: The University of Oxford and Oxford University Hospitals NHS Foundation Trust 2019

c. SmPC precautions and concomitant medication

Hydroxychloroquine: Hydroxychloroquine will be used for short-term use (7 days) in this trial. The SmPC and precautions listed below focus on longer term chronic use.

i. Precautions

Hydroxychloroquine might lower blood sugar levels in some people. If participants develop these symptoms, they will be advised in the Patient Information documents to eat something sweet and seek clinical advice if the symptoms continue.

Hydroxychloroquine occasionally causes blurred vision, which typically resolves once the medication is stopped. Participants will be advised via the Participant Information documents that if they develop any problems with vision, they should stop taking the medication immediately, seek clinical advice, and not drive or operate any heavy machinery.

ii. Concomitant medication

Hydroxychloroquine sulfate has been reported to increase plasma digoxin levels. Serum digoxin levels should be closely monitored in participants receiving concomitant treatment.

Hydroxychloroquine sulfate may also be subject to several of the known interactions of chloroquine even though specific reports have not appeared. These include: potentiation of its direct blocking action at the neuromuscular junction by aminoglycoside antibiotics; inhibition of its metabolism by cimetidine which may increase plasma concentration of the antimalarial; antagonism of effect of neostigmine and pyridostigmine; reduction of the antibody response to primary immunisation with intradermal human diploid-cell rabies vaccine.

As with chloroquine, antacids may reduce absorption of hydroxychloroquine so it is advised that a four hour interval be observed between hydroxychloroquine and antacid dosaging.

As hydroxychloroquine may enhance the effects of a hypoglycaemic treatment, a decrease in doses of insulin or antidiabetic drugs may be required.

Halofantrine prolongs the QT interval and should not be administered with other drugs that have the potential to induce cardiac arrhythmias, including hydroxychloroquine. Also, there may be an increased risk of inducing ventricular arrhythmias if hydroxychloroquine is used concomitantly with other arrhythmogenic drugs, such as amiodarone and moxifloxacin.

An increased plasma ciclosporin level was reported when ciclosporin and hydroxychloroquine were co-administered.

Hydroxychloroquine can lower the convulsive threshold. Co-administration of hydroxychloroquine with other antimalarials known to lower the convulsion threshold (e.g. mefloquine) may increase the risk of convulsions. Also, the activity of anti-epileptic drugs might be impaired if co-administered with hydroxychloroquine. In a single-dose interaction study, chloroquine has been reported to reduce the bioavailability of praziquantel. It is not known if there is a similar effect when hydroxychloroquine and praziquantel are co-administered. Per extrapolation, due to the similarities in structure and pharmacokinetic parameters between hydroxychloroquine and chloroquine, a similar effect may be expected for hydroxychloroquine.

© Copyright: The University of Oxford and Oxford University Hospitals NHS Foundation Trust 2019

There is a theoretical risk of inhibition of intra-cellular α-galactosidase activity when hydroxychloroquine is co-administered with agalsidase.

iii. Pregnancy and Breastfeeding

A moderate amount of data on pregnant women (between 300 – 1000 pregnancy outcomes), including prospective studies in long-term use with large exposure, have not observed a significant increased risk of congenital malformations or poor pregnancy outcomes. Hydroxychloroquine crosses the placenta. Only limited non-clinical data are available for hydroxychloroquine, data on chloroquine have shown developmental toxicity at high supratherapeutic doses and a potential risk of genotoxicity in some test systems. Therefore, hydroxychloroquine sulfate should be avoided in pregnancy except when, in the judgement of the physician, the individual potential benefits outweigh the potential hazards. Careful consideration should be given to using hydroxychloroquine during lactation, since it has been shown to be excreted in small amounts in human breast milk, and it is known that infants are extremely sensitive to the toxic effects of 4-aminoquinolines.
Pregnancy and breastfeeding are exclusion criteria for the hydroxychloroquine arm of the PRINCIPLE trial.

5. Safety reporting

Hydroxychloroquine: has a well-documented safety profile and is a commonly used medication in a primary care setting (see above).

Common symptoms of hydroxychloroquine include abdominal pain; appetite decreased; diarrhoea; emotional lability; headache; nausea; skin reactions; vision disorders; and vomiting.
Mechanisms for safety reporting are outlined in the trial protocol.

We will call all participants randomised to hydroxychloroquine on day 7 to ask about cardiovascular AEs. Our team of clinicians will review any AEs relating to cardiovascular symptoms from the day 7 call, and assess whether these may be related to hydroxychloroquine. If AEs are thought to be related and it's deemed necessary by the assessing clinician, the participant's GP will be contacted to arrange a face-to-face visit for further clinical evaluation.

© Copyright: The University of Oxford and Oxford University Hospitals NHS Foundation Trust 2019

20 APPENDIX E: USUAL CARE PLUS AZITHROMYCIN ARM (DISCONTINUED)

1. Background and rationale

a. Evidence for potential Azithromycin benefits in COVID-19

Atypical macrolides, especially Azithromycin, have activities that may be beneficial in the treatment of COVID-19 patients, and especially those in the at-risk or age range of the PRINCIPLE trial.

Firstly, Azithromycin appears to have some anti-viral mechanisms. In COVID-19, Azithromycin appears to inhibit viral replication and therefore reduces shedding. In the small open observational trial of Gautret *et al* the addition of azithromycin to hydroxychloroquine (HCQ) (at 200 tds for 10 days) in 6 of the 14 HCQ subjects of the total 36 COVID-19 patients in the study significantly reduced viral shedding at 3 days to 15% (one subject) versus 70% in the HCQ arm and 95% in the indirect control arm, with no shedding at 6 days in the combination arm versus 50% and 90% respectively.(22) Azithromycin was also used in some Chinese observational and interventional studies.

Azithromycin has also been shown to be active ***in vitro*** against Zika and Ebola viruses,(23-25) and to prevent severe respiratory tract infections when administrated to patients suffering viral infection.(26) Inhibition of viral infections by azithromycin may be linked to its suppressive effect on the production of viral interferon.(27) Longer term administration of low dose azithromycin in COPD has been shown to suppress proinflammatory cytokine production, potentiate macrophage phagocytosis and anti-inflammatory cytokine expression.(28-30) Azithromycin use is also associated with a decrease in the expression of human HLA (human leukocyte antigen) complex molecules in the respiratory tract, including HLA-A, HLA-B, HLA-DPA1, HLA-DRA, HLA-DRB4.(31)

b. Importance of treating CAP or CAP risk in the elderly or immuno-compromised

An important secondary pathway to severe illness and death with COVID-19 may be secondary infection and sepsis in the immune-compromised state, especially secondary community or hospital acquired pneumonia. Older people are more susceptible to pneumonia because of comorbidities, a weakened immune system and are therefore more likely to die.(32) The onset of pneumonia in the elderly can often be rapid, and for severe pneumonia, the prognosis is poor: as many as one in five will die.(32) Severe pneumonia is more prevalent the older you are and in those with more serious underlying diseases.(33) The leading cause of death is respiratory insufficiency. Death has been shown to increase in those not responding to initial antimicrobials, and consequently, the initial selection of the agent is important.
Common causative organisms in the elderly admitted to the hospital with pneumonia include *Haemophilus influenza, Staphylococcus aureus, Streptococcus pneumoniae,* and *Mycoplasma pneumoniae*. In severe pneumonia, *S. aureus, Klebsiella pneumoniae*, and *Pseudomonas aeruginosa* have been identified as common causative organisms. Older patients often have polymicrobial infections, which may be a factor in non-responders. Assessment of 12,945 US

© Copyright: The University of Oxford and Oxford University Hospitals NHS Foundation Trust 2019

Medicare inpatients over 65 with pneumonia found that initial treatment with a second-generation cephalosporin plus macrolide ([HR, 0.71; 95% CI, 0.52-0.96), a non-pseudomonas third-generation cephalosporin plus a macrolide (HR, 0.74; 0.60-0.92), or a fluoroquinolone alone (HR, 0.64; 0.43-0.94) was associated with lower 30-day mortality.(34)

For CAP management NICE guidance currently recommends Amoxycillin 500mg tds combined with Clarithromycin 500mg bd for 5 days or, in penicillin sensitive, Clarithromycin 500mg bd for 5 days or Doxycycline 200mg stat then 100mg daily for the next 4 days. They also recommend starting therapy within 4 hours. The identification of the early stages of pneumonia in older patients can prove challenging since traditional symptoms and signs, including fever, may be lacking.

Azithromycin will have at least as broad a spectrum of action as clarithromycin in terms of bacterial infections and the additional potential anti-viral activity which has not been observed for other macrolides like Clarithromycin. It will also cover atypical organisms.

2 Changes to outcome measures

The addition of this usual care plus azithromycin arm will not require any changes to outcome measures

3 Eligibility criteria specifically related to azithromycin

Inclusion criteria: No changes

Exclusion criteria:

- Pregnancy
- Breastfeeding
- Known severe hepatic impairment;
- Known severe renal impairment;
- Known myasthenia gravis;
- Previous adverse reaction to, or currently taking, azithromycin or other macrolides or ketolides
- Patients taking the following drugs: hydroxychloroquine or chloroquine, sotalol, amiodarone, ciclosporin, digoxin, bromocriptine, cabergoline, ergotamine, ergometrine, methysergide or any ergot derivatives.
- Already taking antibiotics for an acute condition
- Known congenital or documented QT prolongation
- Known allergy to soya or peanut due to the risk of hypersensitivity reactions

4 Detail of intervention

Participants randomised to the usual care plus azithromycin arm will receive usual clinical care as per NHS guidelines, plus a course of oral azithromycin 500mg daily for three days. We will use the IMP distribution methods described in the protocol to deliver IMP to participants.

© Copyright: The University of Oxford and Oxford University Hospitals NHS Foundation Trust 2019

a. Investigational Medicinal Product (IMP) description

Azithromycin 250mg capsules. Participants in this arm will take 500 mg (two capsules) once daily for 3 days. The capsules are for oral administration.

Special instructions:
Azithromycin must be taken at least 1 hour before or 2 hours after antacids as this affects overall bioavailability. Azithromycin must be taken at least 1 hour before or 2 hours after food.

The marketing authorisation holder is: Teva UK Limited, Brampton Road, Hampden Park, Eastbourne, East Sussex, BN22 9AG, UK.
Marketing authorisation number: PL 00289/1570

b. Storage of IMP

Azithromycin: Stored at room temperature in locked cupboards in restricted access rooms in the Nuffield Department of Primary Care Health Sciences; in locked cupboards in restricted access rooms in GP Practices; in Pharmacies.

c. SmPC precautions and concomitant medication

i. Precautions

Azithromycin is a commonly prescribed antibiotic with an established safety profile. The SmPC advises caution using azithromycin in the following conditions:
Elderly people with proarrhythmic conditions due to the risk of developing cardiac arrhythmia and torsades de pointes including patients with congenital or documented QT prolongation; receiving treatment with other active substances known to prolong QT interval such as anti-arrhythmics (e.g. amiodarone and sotalol), cisapride, and fluoroquinolones such as moxifloxacin and levofloxacin; known hypokalaemia and hypomagnesaemia; significant hepatic or renal impairment; patients with neurological or psychiatric disorders; myasthenia gravis. Azithromycin as other with the use of nearly all antibacterial agents, alters the normal flora of the colon leading to overgrowth of Clostridium difficile which can lead to Clostridium difficile associated diarrhoea.

ii. Concomitant medications

Effects of other medicinal products on azithromycin:

Antacids

In a pharmacokinetic study investigating the effects of simultaneous administration of antacids and azithromycin, no effect on overall bioavailability was seen, although the peak serum concentrations were reduced by approximately 25%. In patients receiving both azithromycin and antacids, the medicinal products should not be taken simultaneously. Azithromycin must be taken at least 1 hour before or 2 hours after the antacids.

Co-administration of azithromycin prolonged-release granules for oral suspension with a single 20 ml dose of co-magaldrox (aluminium hydroxide and magnesium hydroxide) did not affect the rate and extent of azithromycin absorption.

Co-administration of a 600 mg single dose of azithromycin and 400 mg efavirenz daily for 7 days did not result in any clinically significant pharmacokinetic interactions.

© Copyright: The University of Oxford and Oxford University Hospitals NHS Foundation Trust 2019

Fluconazole

Co-administration of a single dose of 1200 mg azithromycin did not alter the pharmacokinetics of a single dose of 800 mg fluconazole. Total exposure and half-life of azithromycin were unchanged by the coadministration of fluconazole, however, a clinically insignificant decrease in C_{max} (18%) of azithromycin was observed.

Nelfinavir

Co-administration of azithromycin (1200 mg) and nelfinavir at steady state (750 mg three times daily) resulted in increased azithromycin concentrations. No clinically significant adverse effects were observed and no dose adjustment is required.

Rifabutin

Coadministration of azithromycin and rifabutin did not affect the serum concentrations of either medicinal product.

Neutropenia was observed in subjects receiving concomitant treatment of azithromycin and rifabutin. Although neutropenia has been associated with the use of rifabutin, a causal relationship to combination with azithromycin has not been established.

Terfenadine

Pharmacokinetic studies have reported no evidence of an interaction between azithromycin and terfenadine. There have been rare cases reported where the possibility of such an interaction could not be entirely excluded; however, there was no specific evidence that such an interaction had occurred.

Cimetidine

In a pharmacokinetic study investigating the effects of a single dose of cimetidine, given 2 hours before azithromycin, on the pharmacokinetics of azithromycin, no alteration of azithromycin pharmacokinetics was seen.

Effect of azithromycin on other medicinal products:
Ergotamine derivatives

Due to the theoretical possibility of ergotism, the concurrent use of azithromycin with ergot derivatives is not recommended.

Digoxin and colchicine (P-gp substrates)

Concomitant administration of macrolide antibiotics, including azithromycin, with P-glycoprotein substrates such as digoxin and colchicine, has been reported to result in increased serum levels of the P-glycoprotein substrate. Therefore, if azithromycin and P-gp substrates such as digoxin are administered concomitantly, the possibility of elevated serum concentrations of the substrate should be considered.

Coumarin-Type Oral Anticoagulants

In a pharmacokinetic interaction study, azithromycin did not alter the anticoagulant effect of a single 15-mg dose of warfarin administered to healthy volunteers. There have been reports received in the post-marketing period of potentiated anticoagulation subsequent to co-administration of azithromycin and coumarin-type oral anticoagulants. Although a causal relationship has not been established, consideration should be given to the frequency of

© Copyright: The University of Oxford and Oxford University Hospitals NHS Foundation Trust 2019

monitoring prothrombin time when azithromycin is used in patients receiving coumarin-type oral anticoagulants.

Cyclosporin

In a pharmacokinetic study with healthy volunteers that were administered a 500 mg/day oral dose of azithromycin for 3 days and were then administered a single 10 mg/kg oral dose of cyclosporin, the resulting cyclosporin C_{max} and AUC_{0-5} were found to be significantly elevated. Consequently, caution should be exercised before considering concurrent administration of these drugs. If coadministration of these drugs is necessary, cyclosporin levels should be monitored and the dose adjusted accordingly.

Theophylline

There is no evidence of a clinically significant pharmacokinetic interaction when azithromycin and theophylline are co-administered to healthy volunteers. As interactions of other macrolides with theophylline have been reported, alertness to signs that indicate a rise in theophylline levels is advised.

Trimethoprim/sulfamethoxazole

Coadministration of trimethoprim/sulfamethoxazole DS (160 mg/800 mg) for 7 days with azithromycin 1200 mg on Day 7 had no significant effect on peak concentrations total exposure or urinary excretion of either trimethoprim or sulfamethoxazole. Azithromycin serum concentrations were similar to those seen in other studies.

Zidovudine

Single 1000 mg doses and multiple 1200 mg or 600 mg doses of azithromycin had little effect on the plasma pharmacokinetics or urinary excretion of zidovudine or its glucuronide metabolite. However, administration of azithromycin increased the concentrations of phosphorylated zidovudine, the clinically active metabolite, in peripheral blood mononuclear cells. The clinical significance of this finding is unclear, but it may be of benefit to patients.

Azithromycin does not interact significantly with the hepatic cytochrome P450 system. It is not believed to undergo the pharmacokinetic drug interactions as seen with erythromycin and other macrolides. Hepatic cytochrome P450 induction or inactivation via cytochrome-metabolite complex does not occur with azithromycin.

Astemizole, alfentanil

There are no known data on interactions with astemizole or alfentanil. Caution is advised in the co-administration of these medicines with azithromycin because of the known enhancing effect of these medicines when used concurrently with the macrolid antibiotic erythromycin.

Atorvastatin

Coadministration of atorvastatin (10 mg daily) and azithromycin (500 mg daily) did not alter the plasma concentrations of atorvastatin (based on a HMG CoA-reductase inhibition assay).

However, post-marketing cases of rhabdomyolysis in patients receiving azithromycin with statins have been reported.

Carbamazepine

© Copyright: The University of Oxford and Oxford University Hospitals NHS Foundation Trust 2019

In a pharmacokinetic interaction study in healthy volunteers, no significant effect was observed on the plasma levels of carbamazepine or its active metabolite in patients receiving concomitant azithromycin.

Cisapride

Cisapride is metabolized in the liver by the enzyme CYP 3A4. Because macrolides inhibit this enzyme, concomitant administration of cisapride may cause the increase of QT interval prolongation, ventricular arrhythmias and torsades de pointes.

Cetirizine

In healthy volunteers, coadministration of a 5-day regimen of azithromycin with cetirizine 20 mg at steady-state resulted in no pharmacokinetic interaction and no significant changes in the QT interval.

Didanosins (Dideoxyinosine)

Coadministration of 1200 mg/day azithromycin with 400 mg/day didanosine in 6 HIV-positive subjects did not appear to affect the steady-state pharmacokinetics of didanosine as compared with placebo.

Efavirenz

Coadministration of a 600 mg single dose of azithromycin and 400 mg efavirenz daily for 7 days did not result in any clinically significant pharmacokinetic interactions.

Indinavir

Coadministration of a single dose of 1200 mg azithromycin had no statistically significant effect on the pharmacokinetics of indinavir administered as 800 mg three times daily for 5 days.

Methylprednisolone

In a pharmacokinetic interaction study in healthy volunteers, azithromycin had no significant effect on the pharmacokinetics of methylprednisolone.

Midazolam

In healthy volunteers, coadministration of azithromycin 500 mg/day for 3 days did not cause clinically significant changes in the pharmacokinetics and pharmacodynamics of a single 15 mg dose of midazolam.

Sildenafil

In normal healthy male volunteers, there was no evidence of an effect of azithromycin (500 mg daily for 3 days) on the AUC and C_{max} of sildenafil or its major circulating metabolite.

Triazolam

In 14 healthy volunteers, coadministration of azithromycin 500 mg on Day 1 and 250 mg on Day 2 with 0.125 mg triazolam on Day 2 had no significant effect on any of the pharmacokinetic variables for triazolam compared to triazolam and placebo.

© Copyright: The University of Oxford and Oxford University Hospitals NHS Foundation Trust 2019

iii. Fertility, pregnancy and lactation

Pregnancy

There are no adequate data from the use of azithromycin in pregnant women. In reproduction toxicity studies in animals azithromycin was shown to pass the placenta, but no teratogenic effects were observed. The safety of azithromycin has not been confirmed with regard to the use of the active substance during pregnancy. Therefore azithromycin should only be used during pregnancy if the benefit outweighs the risk.

5 Safety reporting

Mechanisms for safety reporting are outlined in the protocol. In brief, we will collect symptoms and side effects of azithromycin from symptom diaries and participant telephone calls.

Common symptoms of azithromycin include diarrhoea, abdominal pain, nausea and flatulence. It may also cause headache, dizziness, insomnia, altered taste, pins and needles, changes in vision or hearing, rash, itching, joint pains or fatigue.

© Copyright: The University of Oxford and Oxford University Hospitals NHS Foundation Trust 2019

21 APPENDIX F: USUAL CARE PLUS DOXYCYCLINE ARM (DISCONTINUED)

1. Background and rationale

a. Evidence for potential doxycycline benefits in COVID-19

Doxycycline may be beneficial in the treatment of COVID-19 patients, and especially those in the at-risk or age range of the PRINCIPLE trial.
The rationale for testing doxycycline is based on three reasons:
Firstly, doxycycline may have direct antiviral activity against SARS-CoV-2 based on computer modelling. Analysing all the proteins encoded by SARS-CoV-2 genes and then predicting potential targets by performing target-based virtual ligand screening, doxycycline ranked in the group of compounds with the highest binding affinity to 3CLpro (3-chymotrypsin-like protease). 3CLpro is the main protease in SARS- CoV-2 which is critical in the life-cycle of the virus (35).

Secondly, doxycycline has known anti-inflammatory effects in various human diseases by inhibiting mitogen-activated protein kinase (MAPK) and SMAD pathways (36), as well as potent antioxidant properties(37). Doxycycline reduces the hyperinflammation associated with severe COVID-19 by antagonising metalloproteinases such as MMP9 that are linked with lung injury, including SARS and ARDS(38).

Lastly, from extensive experience in other infectious diseases, doxycycline has broad antimicrobial activity and is efficacious against a broad spectrum of bacteria including atypical bacteria and other pathogens including intracellular plasmodia, chlamydia, rickettsia, and RNA viruses like Dengue fever and chikungunya.

b. Importance of treating CAP or CAP risk in the elderly or immuno-compromised

An important secondary pathway to severe illness and death with COVID-19 may be secondary infection and sepsis in the immune-compromised state, especially secondary community or hospital acquired pneumonia. Older people are more susceptible to pneumonia because of comorbidities, a weakened immune system and are therefore more likely to die.(32) The onset of pneumonia in the elderly can often be rapid, and for severe pneumonia, the prognosis is poor: as many as one in five will die.(32) Severe pneumonia is more prevalent the older you are and in those with more serious underlying diseases.(33) The leading cause of death is respiratory insufficiency. Death has been shown to increase in those not responding to initial antimicrobials, and consequently, the initial selection of the agent is important. Common causative organisms in the elderly admitted to the hospital with pneumonia include *Haemophilus influenza, Staphylococcus aureus, Streptococcus pneumoniae* and less commonly, atypical organisms, such as *Mycoplasma pneumoniae* and *Klebsiella pneumoniae*. All these organisms fall under doxycycline's antimicrobial spectrum.

We are aware that currently NICE, in their COVID-19 rapid guideline, advocates that clinicians offer oral doxycycline for treatment of suspected pneumonia in people who can or wish to be

© Copyright: The University of Oxford and Oxford University Hospitals NHS Foundation Trust 2019

treated in the community if: the likely cause is bacterial or; it is unclear whether the cause is bacterial or viral and symptoms are more concerning or; they are at high risk of complications (older or frail patients, pre-existing comorbidity or have a history of severe illness following previous lung infection).(39) Doxycycline will have at least as broad a spectrum of action as azithromycin in terms of bacterial infections with the potential anti-viral and anti-inflammatory effects.

Doxycycline for acute cough and community acquired pneumonia is recommended in the British National Formulary at a dose of Doxycycline 200mg stat then 100mg daily for the next 4 days. However, its use in COVID-19 is not proven and therefore important to address in this trial. Given the potential anti-inflammatory properties of doxycycline, we will use a slightly extended 7 day course.

2. Changes to outcome measures

The addition of this usual care plus doxycycline arm will not require any changes to outcome measures

3. Eligibility criteria specifically related to doxycycline

Inclusion criteria: No changes
Exclusion criteria:

- Pregnancy
- Breastfeeding
- Myasthenia gravis
- Systemic lupus erythematosus
- Previous adverse reaction to, or currently taking, doxycycline or other tetracyclines
- Sucrose intolerance (i.e. rare hereditary problems of fructose intolerance, glucose galactose malabsorption or sucrose-isomaltase insufficiency)
- Already taking antibiotics for an acute condition
- Patients taking the following drugs: ciclosporin, retinoids (acitretin, alitretinoin, isotretinoin, tretinoin), methotrexate, ergotamine, methoxyflurane, lithium.

4. Detail of intervention

Participants randomised to the usual care plus doxycycline arm will receive usual clinical care as per NHS guidelines, plus a course of oral doxycycline for 7 days. We will use the IMP distribution methods described in the protocol to deliver IMP to participants.

a. Investigational Medicinal Product (IMP) description

Doxycycline 100mg capsules. Participants in this arm will take 200mg on the first day (as a single dose or in divided doses with a twelve hour interval) followed by 100mg a day for 6 days (7 day course in total). The capsules are for oral administration.

© Copyright: The University of Oxford and Oxford University Hospitals NHS Foundation Trust 2019

Special instructions:
Capsules should be swallowed whole with plenty of fluid, while sitting or standing. Capsules should be taken during meals, well before going to bed. Due to the risk of photosensitivity, patients should be advised to avoid exposure to sunlight or sun lamps.

The marketing authorisation holder is:

Accord-UK Ltd (Trading style: Accord), Whiddon Valley, Barnstaple, Devon, EX32 8NS
Marketing authorisation number: PL 0142/0407

b. Storage of IMP

Doxycycline: Stored at room temperature in locked cupboards in restricted access rooms in the Nuffield Department of Primary Care Health Sciences; in locked cupboards in restricted access rooms in GP Practices; in Pharmacies.

c. SmPC precautions and concomitant medication

i. Precautions

Doxycycline is a commonly prescribed antibiotic with an established safety profile. The SmPC states that in elderly patients “doxycycline may be prescribed in the usual dose with no special precautions. No dosage adjustment is necessary in the presence of renal impairment”.

ii. Concomitant medications

Warfarin
There have been reports of prolonged prothrombin time in patients taking warfarin and doxycycline. Tetracyclines depress plasma prothrombin activity and reduced dosage of concomitant anti-coagulants may be necessary

5. Safety reporting

Mechanisms for safety reporting are outlined in the protocol. In brief, we will collect symptoms and side effects from symptom diaries and participant telephone calls.

Common side effects of doxycycline include: Angioedema; diarrhoea; headache; Henoch-Schönlein purpura; hypersensitivity; nausea/vomiting; pericarditis; skin and photosensitivity reaction; dyspnoea; hypotension; peripheral oedema; tachycardia.

© Copyright: The University of Oxford and Oxford University Hospitals NHS Foundation Trust 2019

22 APPENDIX G: USUAL CARE PLUS INHALED CORTICOSTEROID (ICS) ARM

1. Background and rationale

a. Evidence for potential benefits of inhaled corticosteroids in COVID-19 illness

Inhaled corticosteroids (ICS) are a commonly prescribed class of medication throughout the world. They are reasonably cheap and have been used widely for the last 60 years. The inhaled action and type2 pneumocyte target of COVID make ICS a potential therapeutic agent in COVID-19(40). They have been shown to be very effective in improving asthma and COPD care over the long term, where the recommendation is that most, if not all, patients with asthma should be prescribed an inhaled corticosteroid(41),(42) and up to 90% of patients with COPD in the UK are prescribed ICS(43). The rationale of ICS is to reduce the inflammatory process that underlies exacerbations, which can be triggered by viruses in asthma and COPD. Systemic corticosteroids have been found to be effective at reducing mortality amongst hospitalised patients with COVID-19 [46, 47], but it is not known whether pre-hospital treatment with ICS is also beneficial.

Further evidence is as described below:

Evidence from the ARDS literature
ICS in patients at risk of acute respiratory distress syndrome (ARDS) have been shown to improve physiology and reduce inflammatory markers(44). In patients admitted to hospital at risk of ARDS or acute lung injury, there was an almost 50% reduction of ARDS in patients that were using ICS pre-admission, even controlling for covariates such as age, gender and chronic respiratory disease(45). Moreover, this ICS effect can also be seen to improve pulmonary physiology(46).

Potential mechanism of efficacy
Recently published *in vitro* data suggest a role for ICS inhibition of coronavirus replication in infected epithelial cells(47), whilst there is an indication that there is accelerated hyperinflammation at the onset of SARS-CoV-2 infection(48), which potentially can be modified by anti-inflammatory therapy. This suggests a plausible mechanism for ICS efficacy against COVID-19 in which ICS has a dual role: firstly, toning down the inflammatory "runaway train" (ARDS-like) response affecting a minority of COVID-19 patients; and secondly, inhibiting viral replication. It has long been known that the ICS effect on epithelial cells is as a direct consequence of gene transcription(49), and investigation of gene expression of ACE2 and TMPRSS2 in the sputum of asthmatic patients has very recently demonstrated lower expression of these key receptors in the presence of ICS(50). Furthermore, ICS attenuates expression of the ACE2 receptor in human and murine in vitro and in vivo models(51). This is of relevance as the SARS-CoV-2 mechanism of action is upon direct action of the ACE2 receptor, a receptor highly expressed on epithelial cells in the oral mucosa and type 2 alveolar cells and the serine protease TMPRSS2 for SARS-CoV-2 spike protein priming(52, 53). Furthermore, there is experimental evidence that inhaled corticosteroids inhibit coronavirus replication in vitro(54, 55). SARS-CoV-2 binds to cells via the angiotensin converting enzyme 2 (ACE2) receptor. ACE2 is highly expressed on epithelial cells in the oral mucosa and type 2 alveolar epithelial cells. The use of inhaled corticosteroids as a therapy suggests it would target the cells of interest. Furthermore, the primary action of the inhaled steroids is on the type 2 pneumocytes where viral replication is going to be at its most, where we know that ACE2 receptor expression is high.

© Copyright: The University of Oxford and Oxford University Hospitals NHS Foundation Trust 2019

2. Changes to outcome measures

The addition of this arm will not require any changes to outcome measures.

3. Eligibility criteria specifically related to ICS

Inclusion criteria:

Age criteria: Patients aged ≥65 years, or Patients aged 50-64 years and meeting at least one of the following criteria:

- Known weakened immune system due to a serious illness or medication (e.g. chemotherapy);
- Known heart disease and/or a diagnosis of high blood pressure;
- Known asthma or lung disease;
- Known diabetes;
- Known mild hepatic impairment;
- Known stroke or neurological problem;
- Self-report obesity or body mass index ≥35 kg/m^2

Exclusion criteria:

- A known allergy to inhaled corticosteroids
- Any known contraindication to inhaled corticosteroids (as per SmPC, *patients with rare hereditary problems of galactose intolerance, the Lapp lactase deficiency or glucose-galactose malabsorption should not take this medicine. Lactose, the excipient in the product, contains small amounts of milk proteins and can therefore cause allergic reactions*).
- Patient currently prescribed inhaled or systemic corticosteroids
- Unable to administer inhaler

4. Detail of intervention

Participants randomised to the usual care plus ICS arm will receive usual clinical care as per NHS guidelines, plus inhaled corticosteroids for 14 days. We will use the IMP distribution methods described in the protocol to deliver IMP to participants.

a. Investigational Medicinal Product (IMP) description

The IMP is the inhaled corticosteroid budesonide (dose 400mcg, Pulmicort turbohaler®). Inhaled budesonide comes in a polyethylene container consisting of a white cover screwed onto a brown bottom plate. Inside this is the inhaler with its main parts: a mouthpiece, a dosing mechanism and a substance store. The device will have 50 actuations of 400mcg/actuation. This product has marketing authorisation in the UK (PL 17901/0164) and is manufactured by AstraZeneca UK Ltd, 600 Capability Green, Luton, LU1 3LU, UK. This IMP will be taken as 2 puffs twice a day for 14 days.

b. Storage of IMP

© Copyright: The University of Oxford and Oxford University Hospitals NHS Foundation Trust 2019

Stored at room temperature in locked cupboards in restricted access rooms in the Nuffield Department of Primary Care Health Sciences; in locked cupboards in restricted access rooms in GP practices; in Pharmacies

c. SmPC precautions and concomitant medication

iii. Precautions

Budesonide is a commonly prescribed inhaled steroid with an established safety profile.

iv. Concomitant medications

Largely, there is no restriction to concomitant medications using inhaled budesonide. The SmPC states that concomitant treatment with ketoconazole, HIV protease inhibitors or other potent CYP3A inhibitors may increase systemic budesonide levels, but that this is of little clinical significance for a short term treatment of 2 weeks, which is the duration of IMP use in the trial.

5. Safety reporting

Mechanisms for safety reporting are outlined in the protocol. In brief, we will collect symptoms and side effects from symptom diaries and participant telephone calls.

Common and/or potential side effects from IMP include:

- Cough immediately after inhaling
- Mouth and throat pain
- Hoarse voice
- Oral candidiasis (thrush)

These are all reversible upon ceasing IMP.

© Copyright: The University of Oxford and Oxford University Hospitals NHS Foundation Trust 2019

23 APPENDIX H: USUAL CARE PLUS COLCHICINE

1. Background and rationale

a. Evidence for potential benefits of colchicine in COVID-19 illness

Colchicine is licenced and widely used in the UK for the treatment of acute gout and has been investigated as a possible treatment for COVID-19. Reyes and colleagues (56) have summarised existing clinical evidence for colchicine for COVID-19 thus:

"A retrospective single-centre study of 87 ICU patients with COVID-19 demonstrated a lower risk of death in patients on colchicine (adjusted HR 0.41, 95% CI 0.17 to 0.98).(57) The Greek Effects of Colchicine in COVID-19 (GRECCO-19) trial was the first prospective open-label randomised trial evaluating colchicine versus usual care in early hospitalised patients. This study of 105 patients found a significant reduction in the primary clinical outcome of a two-point deterioration on WHO disease severity scale.(58) An Italian study compared 122 hospitalised patients who received colchicine plus standard-of-care (lopinavir/ritonavir, dexamethasone or hydroxychloroquine) with 140 hospitalised patients receiving standard-of-care alone. Colchicine had a significant mortality benefit versus controls (84% vs 64% survival).(59) A third prospective study randomised 38 hospitalised COVID-19 patients to colchicine or placebo in a double-blinded manner.(60) Patients receiving colchicine had less need for supplemental oxygen at day 7 (6% vs 39%) and were more likely to be discharged at day 10 (94% vs 83%). Colchicine subjects also had greater reduction of CRP, and no increase in serious adverse events."

More recently, a systematic review and meta-analysis (in preprint) supports the notion that colchicine lowers the risk of mortality (HR of 0.25, 95% CI [0.09, 0.66], six studies, n=5,033) However, the summary point estimate from the three included RCTs showed a signal towards mortality benefit that was not statistically significant among patients receiving colchicine versus placebo (OR 0.49, 95% CI [0.20, 1.24]).(61)

The COLCORONA randomised clinical trial has now reported in a pre-print.(62) It randomised 4488 patients to treatment with colchicine (0.5 mg twice daily for 3 days and once daily thereafter) or placebo for 28 days. The primary endpoint occurred in 4.7% of the patients in the colchicine group and 5.8% of those in the placebo group (odds ratio, 0.79; 95.1% confidence interval (CI), 0.61 to 1.03; P=0.08). Among the 4159 patients with PCR-confirmed COVID-19, the primary endpoint occurred in 4.6% and 6.0% of patients in the colchicine and placebo groups, respectively (odds ratio, 0.75; 95% CI, 0.57 to 0.99; P=0.04). In these patients with PCR-confirmed COVID-19, the odds ratios were 0.75 (95% CI, 0.57 to 0.99) for hospitalization due to COVID-19, 0.50 (95% CI, 0.23 to 1.07) for mechanical ventilation, and 0.56 (95% CI, 0.19 to 1.66) for death. Serious adverse events were reported in 4.9% and 6.3% in the colchicine and placebo groups (P=0.05); pneumonia occurred in 2.9% and 4.1% of patients (P=0.02). Diarrhoea was reported in 13.7% and 7.3% in the colchicine and placebo groups (P<0.0001).

This large-scale study of early treatment in those 40 years and over with symptoms of more severe illness or comorbidity suggests that colchicine treatment early on in the illness reduces the need for hospitalisation or COVID-19. However, the study did not assess impact on recovery, so we

© Copyright: The University of Oxford and Oxford University Hospitals NHS Foundation Trust 2019

don't know from this study if colchicine reduced symptom burden. This is important as those receiving colchicine, predictably, experienced more gastrointestinal side-effects. The study did not recruit to target and some of the findings are not statistically significant. However, these findings need replication before this drug can be considered for routine use for COVID-19.

Another Phase 3 trial in Canadian pre-hospital and hospital settings is investigating colchicine paired with aspirin or interferon beta (ACTCOVID). The trial is still in progress (www2.phri.ca/ACT-COVID-19/).

b. Potential mechanism of action

Colchicine is a broad-spectrum anti-inflammatory agent.(63-66) Colchicine inhibits cellular transport and mitosis by binding to tubulin and preventing its polymerisation as part of the cytoskeleton transport system.(67) Several of the biological therapies that have been studied and/or used in the setting of severe COVID-19 target some of the same pathways as colchicine, including IL-1β (ie, anakinra) and IL-6 (ie, tocilizumab and sarilumab). Colchicine differs from these agents in having pleotropic mechanisms of action, being less potent on any single target, and being an oral agent. Potential benefits of colchicine compared to these biological therapies when used in the midst of cytokine storm, are that colchicine is not immunosuppressive, is not known to increase risk of infection, and is inexpensive.

There is evidence that the inflammasome is activated in COVID-19 and that the degree of activation is correlated with disease severity.(68) Inflammasomes are key components of effective host immune responses to pathogens. Excessive inflammasome activation (specifically NLRP3 inflammasome) is implicated in chronic inflammatory diseases such as inflammatory bowel disease, rheumatoid arthritis and gout, and with ARDS and ALI (acute lung injury) pathology following respiratory viral infections. Additionally, colchicine may have relevance to COVID-19 associated inflammatory pathology that include: inhibition of neutrophil chemotaxis in response to cytokines, inhibition of NFkB activation (a protein complex that controls transcription of DNA, cytokine production and cell survival) or expression, inhibition of neutrophil adhesion to endothelium, inhibition of neutrophil respiratory burst and reactive oxygen species generation, reduced TNF receptor expression on macrophages and endothelial cells and increased TGFβ expression(67) Of note though is that many of these latter actions of colchicine occur at much lower concentrations that are required for NLRP3 inflammasome activation in response to MSU crystals. Symptoms such as fever, joint and muscle ache, and headache may be ameliorated by a general anti-inflammatory action.

Therefore, when used early in the course of COVID-19, colchicine may prevent the progression from inflammatory activation to a hyperinflammatory state. The potential benefits of colchicine may therefore be maximised when used in the community, where earlier treatment could alleviate symptom burden, and prevent disease progression, hospitalisation and adverse outcomes.

2. Changes to outcome measures

The addition of the usual care plus colchicine arm will not require any changes to outcome measures.

© Copyright: The University of Oxford and Oxford University Hospitals NHS Foundation Trust 2019

3. Eligibility criteria specifically related to colchicine

Inclusion criteria: No changes required

Exclusion criteria:

- Hypersensitivity to the active substance or to any of the excipients listed in section (Lactose, Pregelatinised Maize Starch, Stearic Acid, Purified Talc, Purified Water, Ethanol 96%)
- Known or suspected pregnancy
- Breastfeeding
- Women of childbearing potential (premenopausal female that is anatomically and physiologically capable of becoming pregnant*) and not prepared to use highly effective contraception for the 28 day duration of follow up in the study**
- Known blood dyscrasias
- Known severe renal impairment or requiring dialysis
- Known severe hepatic impairment
- Currently taking any of the following drugs: colchicine, clarithromycin, erythromycin, ketoconazole, itraconazole, voriconazole, HIV protease inhibitors (e.g. ritonavir, atazanavir), cobicistat, verapamil, diltiazem, cyclosporin, quinidine, disulfiram, grapefruit juice
- Inflammatory bowel disease or chronic diarrhoea

* As recorded by the participant on the screening form and confirmed on Day 3 telephone call

Highly effective methods have typical-use failure rates of less than 1% and include male or female sterilisation and long-acting reversible contraceptive (LARC) methods (intrauterine devices and implants). Women using another method of contraception, such as a combined hormonal method, progestogen only pill or injection, are eligible* ***if *they are willing to use an additional barrier method (e.g. male condom) for the 28 day duration of follow-up in the trial.*

The Patient Information Sheet Appendix, which the participant must read prior to providing informed consent, will clearly state the exclusion criteria listed above and the participant will be asked if they meet any of these exclusion criteria at the screening stage of the trial. The assessing clinician will then review the participant's responses against their medical record to confirm eligibility.

4. Detail of intervention

Participants randomised to the usual care plus colchicine arm will receive usual clinical care as per NHS guidelines, plus 500 micrograms of colchicine to be taken each day for 14 days. We will use the IMP distribution methods described in the protocol to deliver IMP to participants.

Pharmacokinetic modelling shows that for a given dose of colchicine plasma exposure is greater in older (especially female) people than younger people; the target patient population for this study will be predominantly older subjects. Colchicine is also subject to accumulation in leucocytes- a target cell in this COVID19. Taking these factors into account and given the relatively

© Copyright: The University of Oxford and Oxford University Hospitals NHS Foundation Trust 2019

narrow therapeutic index of colchicine, the dosing regimen will be 500 microgrammes daily for 14 days. A loading dose such as that used in the COLCORONA study and the use of a higher daily dose increases the risk of dose related adverse drug reactions (ADRs). Given the outpatient setting for this study with limited opportunity for laboratory and clinical monitoring, the proposed dose is expected to achieve a balance between clinically meaningful exposure in target cells while minimising the risk of ADRs. Some other studies have treated for longer than 14 days (7) but the natural history of COVID19 is that in most cases the course of disease for that individual has been established within 2 weeks.

It is acknowledged that the total dose of colchicine administered in this study (7 mg) is modestly greater than that recommended for treatment of acute gout (6 mg) but the proposed regimen has been designed to minimise the risk of ADRs.

Note: The British National Formulary advises a maximum total of 6mg per treatment course for acute gout (1mg less than the total for this study). However, the treatment course for gout is given over up to three days at 500mcg 2-4 times per day initially. In the PRINCIPLE Trial, the treatment will be spread over two weeks at a lower daily dose.

We propose a shorter duration and no loading dose compared to the COLCORONA study (62), given the incidence of side-effects found in that study, and that by two weeks, most patients with COVID-19 have either recovered or been hospitalised. Therefore, the window of opportunity for a positive benefit is mainly over two weeks, and a shorter duration without a loading dose will minimize risk of side-effects, while offering potential benefit. There are no dose-findings studies for colchicine in COVID-19. Our proposed dosing regime is based on expert pharmacological opinion and an appraisal of side-effects balanced against potential benefit in a large-scale community study without face-to-face recruitment and monitoring.

a. Investigational Medicinal Product (IMP) description

Colchicine 500 microgram (µg) tablets. The tablets are for oral administration. One tablet to be taken daily by mouth for 14 days (14 tablets in total).

Special instructions: Tablets should be swallowed whole with a glass of water.

Manufacturer:
The Marketing Authorisation holder is:
Accord-UK Ltd
(Trading style: Accord)
Whiddon Valley
Barnstaple
Devon
EX32 8NS
Marketing authorisation number is: PL 0142/0918

Labelling and QP release:
Vertical Pharma Resources Ltd (trading as IPS Pharma), 41 Central Avenue, West Molesey, KT8 2QZ, UK

© Copyright: The University of Oxford and Oxford University Hospitals NHS Foundation Trust 2019

Authorisation number: WDA(H) 32879

b. Storage of IMP

Colchicine: This medicine does not need any special storage conditions, but we will ask participants to store the medication at room temperature. The medication will be stored in locked cupboards in restricted access rooms in the Nuffield Department of Primary Care Health Sciences; in locked cupboards in restricted access rooms in GP Practices; in Pharmacies.

c. SmPC Precautions, concomitant medications, pregnancy and lactation

i. SmPC Precautions

Colchicine is a commonly prescribed drug in UK primary care and has a well-described safety profile due to its regulatory assessments for the authorisation in gout. Typical treatment doses for acute gout are 500 micrograms 2–4 times a day until symptoms relieved, maximum 6 mg per course.

Colchicine is teratogenic in animal studies and contraindicated in patients with severe renal (including patients undergoing haemodialysis) or severe hepatic impairments. Colchicine may cause severe bone marrow depression (agranulocytosis, aplastic anaemia, thrombocytopenia) and blood cell dyscrasia.

Colchicine is potentially toxic with a narrow therapeutic window.
Symptoms of acute overdosage may be delayed (3 hours on average): nausea, vomiting, abdominal pain, haemorrhagic gastroenteritis, volume depletion, electrolyte abnormalities, leucocytosis, hypotension in severe cases. The second phase with life threatening complications develops 24 to 72 hours after drug administration: multisystem organ dysfunction, acute renal failure, confusion, coma, ascending peripheral motor and sensory neuropathy, myocardial depression, pancytopenia, dysrhythmias, respiratory failure, consumption coagulopathy.

Patients at particular risk of toxicity are those with renal or hepatic impairment, gastrointestinal or cardiac disease, and patients at extremes of age. However, colchicine has a good safety profile when used according to the established therapeutic guidelines, and toxicity is rare if the recommended doses are not exceeded.

In PRINCIPLE, we exclude patients with known severe renal and known liver impairment, and have used a cautious dosing regimen, with no loading dose and low daily dose, to minimise risk in participants with other less severe co-morbidities. Our dosing schedule is also shorter in duration than the 30 days used in the large scale, remotely managed COLCORONA trial.(62) In addition, we will mitigate the risk of toxicity by asking each participant taking colchicine the number of tablets remaining via their diary entry to ensure drug accountability.

ii. Concomitant medications

Colchicine is a substrate for both CYP3A4 and the transport protein P-gp. In the presence of CYP3A4 or P-gp inhibitors, the concentrations of colchicine in the blood increase. Toxicity,

© Copyright: The University of Oxford and Oxford University Hospitals NHS Foundation Trust 2019

including fatal cases, have been reported during concurrent use of CYP3A4 or P-gp inhibitors such as macrolides (clarithromycin and erythromycin), ciclosporin, ketoconazole, itraconazole, voriconazole, HIV protease inhibitors, calcium channel blockers (**verapami**l and **diltiazem**) and disulfiram.
Colchicine is contraindicated in patients with renal or hepatic impairment who are taking a P-gp inhibitor (e.g. ciclosporin, verapamil or quinidine) or a strong CYP3A4 inhibitor (e.g. ritonavir, atazanavir, indinavir, clarithromycin, telithromycin, itraconazole or ketaconazole).
Therefore, patients using the above medications will not be eligible for enrolment into the colchicine arm of PRINCIPLE.

iii. Fertility, pregnancy and lactation

Pregnancy and breast-feeding are exclusions for the colchicine arm.

Women of childbearing potential (premenopausal female that is anatomically and physiologically capable of becoming pregnant) and not prepared to use highly effective contraception for the 28 day duration of follow up in the study are excluded from the trial.

5. Safety reporting

Mechanisms for safety reporting are outlined in the protocol. In brief, we will collect symptoms and side effects from symptom diaries and participant telephone calls.

<u>Common and/or potential side effects that may be associated with colchicine include:</u>

Abdominal pain, diarrhoea*, nausea, vomiting.

A meta-analysis of 35 randomised trials of colchicine versus placebo found that the most common and significant adverse effect was diarrhoea. The only other adverse effect that occurred at a greater frequency than placebo was a set of pooled gastrointestinal symptoms including nausea, vomiting, diarrhoea, abdominal pain, loss of appetite, and bloating.(69, 70)

<u>Rare side effects that may be associated with colchicine:</u>

Agranulocytosis; alopecia; bone marrow disorders; gastrointestinal haemorrhage; kidney injury; liver injury; menstrual cycle irregularities; myopathy; nerve disorders; rash; sperm abnormalities; thrombocytopenia

** Side effects that may be associated with COVID-19*

We will report SAEs as defined in the main protocol for hospitalisation and/or death. Participants record symptoms and adverse events on their diary card. Events rated as a 'major problem' will be assessed by a clinician for potential reporting as an SAE.

Drug Accountability

© Copyright: The University of Oxford and Oxford University Hospitals NHS Foundation Trust 2019

We will telephone all participants on Day 3 after randomisation to confirm that they have received their medication. For those receiving a trial treatment, 3 attempts are made to contact the participant to confirm receipt of the medication.

If we are unable to contact patients in the colchicine group, we will confirm and log IMP receipt by checking the patient's daily diary, where they are asked on a daily basis whether they have taken their trial treatment and how many tablets they have left. We can also check via the DHL portal, whether the participant pack containing the medication has been received by the participant, for additional confirmation. IMP receipt will be logged on the central IMP log.

© Copyright: The University of Oxford and Oxford University Hospitals NHS Foundation Trust 2019

24 APPENDIX I: USUAL CARE PLUS FAVIPIRAVIR

1. Background and rationale

a. Evidence for potential benefits of favipiravir in COVID-19 illness

Small clinical trials assessing favipiravir for treatment of COVID-19 have been published, but results are inconclusive.

A randomised controlled trial in China of 240 adults hospitalised with moderate to severe COVID-19 compared favipiravir (1600mg BD day 1; 600mg BD days 2-7) with umifenovir (200mg TDS).(71) In the per-protocol analysis, there was no difference in the primary outcome of clinical recovery by day 7 between the two arms. In a post-hoc analysis restricted to patients with moderate COVID-19, 70/98 (71.43%) had recovered by day 7 in the favipiravir arm, vs 62/111 (55.86%) in the umifenovir arm ($p = 0.0199$). In other post-hoc analyses, time to cessation of fever ($p < 0.0001$) and cough ($p < 0.0001$) was shorter in the favipiravir arm. Adverse events were similar between the two arms, apart from hyperuricaemia which occurred in 13.8% of participants who received favipiravir vs 2.5% among those receiving umifenovir ($p < 0.001$).(71)

A trial in India of 150 patients with mild to moderate COVID-19 compared favipiravir (1800 mg BD day 1; 800 mg BD days 2-14) with usual care.(72) The primary outcome of time to viral clearance was not significantly different between the two arms (favipiravir 5 days [95% CI 4-7] vs usual care 7 days [95% CI 5-8, P=0.129), but there was a significant decrease in the secondary outcome of time to clinical cure in the favipiravir arm (favipiravir 3 days [95% CI 3- 4] vs usual care 5 days [95% CI 4-6], P=0.030). Adverse events were more common in the favipiravir arm (36% vs 8%); these were mainly hyperuricaemia (16.4%) and abnormal liver function tests (6.8%); 76.9% were mild and 23.1% were moderate.

A trial in Russia among 60 adults hospitalised with moderate COVID-19 compared usual care (n=20) vs lower dose favipiravir (1600 mg BD day 1; 600 mg BID days 2–14) (n=20) vs higher dose favipiravir (1800 mg BD day 1; 800 mg BD days 2–14) (n=20).(73) Viral clearance was higher in the favipiravir arms vs usual care by day 5 (25/40 [62.5%] vs 6/20 [30.0%], $p = 0.018$), although this difference was non-significant by day 10 (37/40 [92.5%] vs 16/20 [80.0%], $p = 0.155$). Temperature normalisation was quicker in the favipiravir arms (2 days [IQR 1–3] vs 4 days [IQR 1–8], $p = .007$). 17.5% of patients in the favipiravir arm experienced adverse drug reactions including gastrointestinal disturbances and raised liver function tests. Two patients discontinued the drug early.

A trial in Oman randomised 89 adults hospitalised with moderate to severe COVID-19 to receive favipiravir (1600 mg OD day 1; 600 mg BD days 2-10) combined with inhaled interferon beta-1b, versus hydroxychloroquine. There was no difference between the two groups with regard to the primary outcomes of inflammatory markers at discharge, length of hospital stay, transfer to ICU, or mortality. Adverse events were not reported.(74)

A smaller, non-randomised study among adults hospitalised with COVID-19 in China compared 35 patients treated with favipiravir (1600 mg BD day 1; 600 mg BD days 2-14) with 45 historical controls who were treated with lopinavir/ritonavir (400mg/100mg BD). All patients also

© Copyright: The University of Oxford and Oxford University Hospitals NHS Foundation Trust 2019

received aerosolised IFN-α. Viral clearance was quicker in the favipiravir cohort (median 4 days, IQR 2.5-9) compared to the lopinavir/ritonavir cohort (11 days, IQR 8-13, p <0.001). Improvements in CT chest imaging at 14 days were higher in the favipiravir group (91.4% vs 62.2%, p = 0.004).(75)

These mixed findings highlight the need for a large randomised controlled trial of favipiravir to treat COVID-19 in the community.

b. Potential mechanism of efficacy

Favipiravir is an oral antiviral that is licensed in Japan for use against novel and re-emerging influenzae, and has been used in clinical trials for Ebola (76).(76) Like remdesivir, it is a nucleoside analogue which selectively inhibits viral RNA polymerase, and has been shown to have in vitro activity against a range of RNA viruses (77, 78), including SARS-CoV-2.(9) Favipiravir was one of seven antiviral agents reported to achieve plasma concentrations at least double the reported concentrations required to inhibit 90% of SARS-CoV-2 replication in vitro.(79) In animal models, high dose favipiravir was found to reduce viral titres and lung pathology in SARS-CoV-2 infected hamsters.(80, 81) Given that upper respiratory tract SARS-CoV-2 viral loads peak in the first 3-5 days of illness,(82) antiviral treatments for COVID-19 may be of particular value early in the course of the disease.

2. Changes to outcome measures

The addition of the *usual care plus favipiravir arm* will require the introduction of the secondary outcome measure, safety.

3. Eligibility criteria specifically related to favipiravir

Inclusion criteria: No changes

Exclusion criteria:

- Aged <50 years
- Known or suspected pregnancy
- Breastfeeding
- Women of childbearing potential (premenopausal female that is anatomically and physiologically capable of becoming pregnant*), or male with a partner of childbearing potential, not willing to use highly effective contraceptive** for 28 day duration of the trial.
- Known allergy to favipiravir
- Currently taking favipiravir
- Known history of gout
- Known severe liver disease

* As recorded by the participant on the screening form and confirmed on Day 1 by a call between clinician and participant

© Copyright: The University of Oxford and Oxford University Hospitals NHS Foundation Trust 2019

** Highly effective methods have typical-use failure rates of less than 1% and include male or female sterilisation and long-acting reversible contraceptive (LARC) methods (intrauterine devices and implants) OR If a couple are using another method of contraception, such as a combined hormonal method, progestogen only pill or injection, they are only eligible if they are willing to use an additional barrier method (e.g. male condom) for the 28 day duration of follow-up in the trial.

Note: a barrier method on its own is not sufficient.

The Favipiravir Patient Information Sheet Appendix, which the participant must read prior to providing informed consent, will clearly state the exclusion criteria listed above and the participant will be asked if they meet any of these exclusion criteria at the screening stage of the trial including whether they have any known history of gout or known severe liver disease. The assessing clinician will then review the participant's responses against their medical record to confirm eligibility.

4. Detail of intervention

Participants randomised to the usual care plus favipiravir arm will receive usual clinical care as per NHS guidelines, plus one batch of favipiravir for five days. We will use the IMP distribution methods described in the protocol to deliver IMP to participants.

a. Investigational Medicinal Product (IMP) description

Favipiravir 200 milligram (mg) tablets. The tablets are for oral administration. Nine tablets (1800mg) favipiravir to be taken twice a day on day one, and then four tablets (800mg) twice daily for four days (50 tablets in total).

This product is not licensed for use in the UK.

Manufacturer:
It is manufactured by FujifilmToyama Chemical Company Ltd., TOYAMA CHEMICAL CO., LTD. 2-5, Nishishinjuku 3-chome, Shinjuku-ku, Tokyo 160-0023, Japan.
Manufacturirng Licence No. NHI Number 87625
MA Approval No from outside the EEA: 22600AMX0053000 Japan

Importer, Labelling and QP release:
IPS Pharma, 41 Central Ave, East Molesey, West Molesey KT8 2QZ (32879)

b. Storage of IMP

All study medication is to be kept in a dry area, stored at 1° to 30°C (59° to 86°F) and shielded from direct light in locked cupboards in restricted access rooms in the Nuffield Department of Primary Care Health Sciences; in locked cupboards in restricted access rooms in GP practices; in Pharmacies. The IMP is stable at 1-30°C.

c. Precautions, concomitant medications, pregnancy and lactation

© Copyright: The University of Oxford and Oxford University Hospitals NHS Foundation Trust 2019

i. Precautions

Favipiravir has been used in over 30 clinical trials and has a favourable safety profile.(76) A review of six phase 2 and 3 controlled trials, including 4299 participants and 175 person-years-of-follow-up, found no statistically significant differences in overall proportion of AEs, SAEs, discontinuations due to AEs or LFT elevations between favipiravir and placebo or other treatment arms.(76) However, there was evidence of mild to moderate, asymptomatic, uric acid elevations among patients receiving favipiravir, although these generally returned to normal by 21 days. Overall, follow up times were short (5-21 days) and participants were generally young.

Patients with gout or a history of gout, and patients with hyperuricaemia (blood uric acid level may increase, and symptoms may be aggravated) are excluded.

The participant must avoid excessive exposure to sunlight or artificial ultraviolet light.

ii. Concomitant medications

Restrictions to paracetamol use (limiting daily use in adults to no more than 3000 mg/day) have been incorporated into all clinical study protocols. Participants will be advised they can't consume more than 6 paracetamol tablets in 24 hours.

iii. Pregnancy and breastfeeding

Evidence from animal models suggests that favipiravir has teratogenic potential, and there are no human studies of its use among pregnant or lactating women. The Japanese drug safety bureau advise that women of child-bearing potential should use effective contraception for up to 7 days after the end of treatment (https://www.cdc.gov.tw/File/Get/ht8jUiB_MI-aKnlwstwzvw). Male patients should use the most effective form of contraception (see eligibility criteria for examples) for up to seven days after the end of treatment if they have a female partner of child-bearing potential. Men should also avoid intercourse with pregnant women.

5. Safety reporting

a. Side-effects

Mechanisms for safety reporting are outlined in the protocol. In brief, we will collect symptoms and side effects from symptom diaries and participant telephone calls.

Common and/or potential side effects from IMP include:

- Diarrhoea*[1]
- Nausea[1]
- Headache*[1]
- Urinary Tract Infections[1]
- Vomiting[1]
- Raised liver enzymes
- Elevated uric acid concentrations

*side-effects also seen with COVID-19

© Copyright: The University of Oxford and Oxford University Hospitals NHS Foundation Trust 2019

These are all reversible upon ceasing IMP.

Certain pre-defined moderate (defined above[1]) AEs experienced in the 5 days of favipiravir drug administration, will be reviewed daily and will be assessed by a clinician until resolution.

A systematic review suggests that liver changes do not differ between favipiravir and placebo/other treatments (76). To mitigate the risk of elevated uric acid concentrations, we exclude people with gout. Evidence shows mild to moderate, asymptomatic, uric acid elevations, which return to normal after stopping the medication (76).

We will report SAEs as defined in the main protocol for hospitalisation and/or death.

b. Reference Safety Information (RSI)

See section 7.13 of the Investigator Brochure. No serious adverse reactions are considered expected for the purpose of expedited reporting of suspected unexpected serious adverse reactions (SUSAR).

c. Drug Accountability

We telephone all participants on Day 3 after randomisation to confirm that they have received their medication and read the instructions on the medication card. For those receiving a trial treatment, 3 attempts are made to contact the participant to confirm receipt of the medication.

If we are unable to contact patients in the favipiravir group, we will confirm and log IMP receipt by checking the patient's daily diary, where they are asked on a daily basis whether they have taken their trial treatment, the number of tablets taken and the number of tablets remaining. We can also check via the DHL portal, whether the participant pack containing the medication has been received by the participant, for additional confirmation. IMP receipt will be logged on the central IMP log.

If a participant decides that they no longer wish to take their medication, we will provide a pre-paid envelope so that they can return the medication to the trial team, via courier.

d. Risk/Benefit Assessment

The UK COVID-19 Therapeutics Advisory Panel recommends including favipiravir into the PRINCIPLE platform with an 1800mg loading dose, followed by an 800mg BD maintenance dose, based on a review of efficacy and safety data.

i. Risks

Phase I studies and pharmacokinetic studies of favipiravir have indicated increased blood levels of uric acid and elevation of liver aminotransferases in some individuals (76). In addition, animal studies have indicated potential teratogenicity and the drug is distributed in sperm (83). Taking this evidence into account and to ensure patient safety we will exclude known pregnancy, breastfeeding, severe liver disease and known history of gout, and require participants to use adequate contraception for the duration of the treatment and 28 days of follow-up.

ii. Benefits

The benefits of favipiravir have been shown in several phase I-III studies to significantly alleviate influenza symptoms with a good safety profile (83). From the studies detailed

© Copyright: The University of Oxford and Oxford University Hospitals NHS Foundation Trust 2019

below, the frequency of adverse events was 386/1472 (26.2%) with favipiravir versus 227/894 (25.4%) with placebo and there were no differences in the incidence of any specific adverse event between groups.

A Phase I/II study in type A or B influenza using favipiravir doses 1800mg bd for 1 day followed by 800mg bd for 4 days (101 patients) or 2400mg/600mg/600mg for 1 day followed by 600mg tds for 4 days (82 patients) or placebo (88 patients).

Two Phase III studies in type A or B influenza using favipiravir doses 1800mg bd for 1 day followed by 800mg bd for 4 days (301 and 526 patients respectively) or placebo (322 and 169 patients respectively).

A global phase III study in type A or B influenza using favipiravir 1200mg/400mg for 1 day followed by 400mg bd for 4 days or oseltamivir 75mg bd for 5 days (377 and 380 patients respectively).

A phase II study in type A or B influenza using favipiravir doses 1000mg bd for 1 day followed by 400mg bd for 4 days (88 patients) or 1200mg bd for 1 day followed by 800mg bd for 4 days (121 patients) or placebo (124 patients).

Favipiravir was also used for Ebola virus disease, especially in the JIKI trial in Guinea (84). Doses (2400mg/2400mg/1200mg on Day 1 followed by 1200mg bd) were significantly higher than proposed for the current study. No drug related grade 3 or 4 clinical events were observed. In 41 of the 48 patients who survived, biochemical abnormalities of renal and liver function rapidly improved on treatment; 7 of 48 patients saw transient rises in one marker but all subsequently normalised despite continuing favipiravir. Biochemical abnormalities in patients who died were attributed to severe viral infection.

In London institutions such as Royal Free Hospital and Great Ormond Street Hospital (GOSH), favipiravir has been used as post-exposure prophylaxis for Ebola virus, for chronic norovirus infection, refractory influenza infection, astrovirus, respiratory syncytial virus and seasonal coronavirus. Occasional asymptomatic elevation of liver aminotransferase levels were observed, but no serious adverse events related to the drug. This includes in patients with immunodeficiencies who have received several months-worth of treatment (FLARE Trial).

Favipiravir has been used clinically in several viral infections and has shown beneficial treatment effects in both influenza and COVID-19 studies, as well as having a good safety profile.

© Copyright: The University of Oxford and Oxford University Hospitals NHS Foundation Trust 2019

25. Supplementary Material

A. Abbreviations

AE	Adverse event
AR	Adverse reaction
CI	Chief Investigator
CRF	Case Report Form
CT	Clinical Trials
CTA	Clinical Trials Authorisation
CTRG	Clinical Trials and Research Governance
DMSC	Data Monitoring Committee / Data Monitoring and Safety Committee
DSUR	Development Safety Update Report
GCP	Good Clinical Practice
GP	General Practitioner
HRA	Health Research Authority
HCP	Healthcare professional
IB	Investigators Brochure
ICF	Informed Consent Form
ICH	International Conference on Harmonisation
IMP	Investigational Medicinal Product
ISA	Intervention Specific Appendix
MHRA	Medicines and Healthcare products Regulatory Agency
NHS	National Health Service
NIHR	National Institute of Health Research
RES	Research Ethics Service
PHE	Public Health England
PI	Principal Investigator
PIL	Participant/ Patient Information Leaflet
R&D	NHS Trust Research and Development Department
RCGP RSC	Royal College of General Practitioners Research Surveillance Centre
REC	Research Ethics Committee
RSI	Reference Safety Information
SAE	Serious Adverse Event
SAR	Serious Adverse Reaction
SDV	Source Data Verification
SMPC	Summary of Medicinal Product Characteristics
SOP	Standard Operating Procedure
TSC	Trial Steering Committee
SUSAR	Suspected Unexpected Serious Adverse Reactions
TMF	Trial Master File

© Copyright: The University of Oxford and Oxford University Hospitals NHS Foundation Trust 2019

B. Key Trial Contacts

**Chief Investigator**	Professor Chris Butler<br>Nuffield Department of Primary Care Health Sciences<br>Gibson Building<br>Radcliffe Observatory Quarter<br>Woodstock Road<br>Oxford<br>OX2 6GG<br>christopher.butler@phc.ox.ac.uk
**Sponsor**	Joint Research Office<br>1st floor, Boundary Brook House<br>Churchill Drive,<br>Headington<br>Oxford OX3 7GB<br>ctrg@admin.ox.ac.uk<br>Tel: +44 (0)1865572224<br>Fax: +44 (0)1865572228
**Funder(s)**	UKRI/NIHR
**Clinical Trials Unit**	Primary Care Clinical Trials Unit,<br>Nuffield Department of Primary Care Health Sciences<br>Radcliffe Observatory Quarter<br>Woodstock Road<br>Oxford<br>OX2 6GG<br>principle@phc.ox.ac.uk<br>01865 289296
**Statistician**	Dr Ben Saville,<br>Berry Consultants,<br>Austin, Texas, USA,<br>And<br>Department of Biostatistics,<br>Vanderbilt University School of Medicine,<br>Nashville, Tennessee,<br>USA.<br><br>Dr Ly-Mee Yu<br>Primary Care Clinical Trials Unit,<br>Nuffield Department of Primary Care Health Sciences<br>Radcliffe Observatory Quarter<br>Woodstock Road<br>Oxford<br>OX2 6GG
**Committees**	**DMSC Chair:**<br>Prof. Deborah Ashby<br>Chair in Medical Statistics and Clinical Trials<br>Director of the School of Public Health<br>Imperial College London Faculty of Medicine, School of Public Health,<br>153 Medical School<br>St Mary's Campus<br>Imperial College London<br>deborah.ashby@imperial.ac.uk

© Copyright: The University of Oxford and Oxford University Hospitals NHS Foundation Trust 2019

	(0)20 7594 8704<br><br>**DMSC Members:** Prof Simon Gates<br>Cancer Research Clinical Trials Unit (CRCTU)<br>Institute of Cancer and Genomic Sciences<br>University of Birmingham<br>Edgbaston<br>Birmingham<br>B15 2TT<br>S.Gates@bham.ac.uk<br><br>Prof Gordon Taylor<br>College House,<br>University of Exeter, St Luke's Campus,<br>Heavitree Road,<br>Exeter, EX1 2LU, UK<br>g.j.taylor@exeter.ac.uk<br><br>Prof Nick Francis<br>Primary Care and Population Science,<br>University of Southampton, Southampton, UK.<br>Nick.Francis@soton.ac.uk<br><br>Dr Patrick White<br>7th Floor<br>Capital House<br>Guy's<br>United Kingdom<br>Patrick.white@kcl.ac.uk
	**TSC Chair**<br>Prof Paul Little,<br>Primary Care and Population Science,<br>University of Southampton, Southampton, UK.<br>P.Little@soton.ac.uk<br><br>**TSC Members**<br>Prof Philip Hannaford,<br>NHS Professor of Primary Care<br>University of Aberdeen, Aberdeen, UK<br>p.hannaford@abdn.ac.uk<br><br>Prof Matt Sydes,<br>Professor of Clinical Trials & Methodology,<br>MRC Clinical Trials Unit, University College of London, London, UK<br>m.sydes@ucl.ac.uk<br><br>*PPI representatives*<br>Ms Carol Green<br>Mr Tim Mustill

© Copyright: The University of Oxford and Oxford University Hospitals NHS Foundation Trust 2019

C. Objectives and Outcome Measures

	Objectives	Outcome Measures	Timepoint (s)
Primary	To assess the effectiveness of trial treatments in reducing<br>1) Time to recovery, for patients<br>2) Hospitalisation and/or death.	1) Time to self-reported recovery, defined as the first instance that a participant reports feeling recovered from possible COVID-19, and<br>2) Hospitalisation and/or death	Within 28 days of randomisation<br>Patient report, Study Partner report, medical records, Daily online symptom scores
Secondary	To explore whether trial treatment will affect<br>1) Participant reported illness severity, reported by daily rating of how well participant feels.<br>2) Duration of severe symptoms and symptom recurrence<br>3) Contacts with the health services<br>4) Consumption of antibiotics<br>5) Hospital assessment without admission<br>6) Oxygen administration<br>7) Intensive Care Unit admission<br>8) Mechanical ventilation<br>9) Duration of hospital admission<br>10) Negative effects on well being<br>11) New infections in household<br>12) To determine if effects are specific to those with a	1-3. Participant reports daily and monthly (after 28 days) symptoms.<br>4. Contacts with health services reported by patients and/or captured by reports of patients' medical records if the practice is a member of the RCGP RSC network<br>5. Bi-weekly reports from participants' primary care medical records<br>6-10. Patient report/carer report/medical record in primary and secondary care<br>11. WHO-5 Well Being Index<br>12. Reports of new infections in the household (from daily questionnaire)<br>13. Swab test results will indicate an "Intention to Treat Infected" group within the overall cohort for sub analysis. Blood test results on recovery (optional) for	Daily online symptom scores.<br>Telephone call or text on days 2, 7, 14 and 28 and once a month for 12 months if data is not obtained through the online diary.<br><br>GP notes review if available through Oxford RCGP RSC network; otherwise, other sources of routinely collected data after 28 days. Medical notes review for up to 10 years.<br><br>HES/ONS/EMIS/Medical record data linkage after 28 days if patients have been assessed in hospital<br><br>Swab result from medical records, the supporting laboratory and/or convalescent blood test result for evidence of historic COVID-19

© Copyright: The University of Oxford and Oxford University Hospitals NHS Foundation Trust 2019

	positive test for SARS-CoV-2	evidence of historic COVID-19	WHO 5 Well Being Index at baseline, day 14, and day 28 and monthly for up to 12 months, either via online diary or telephone
	13) To investigate the safety of treatments that are not licensed in the UK	Evaluation of overall safety of drugs by the monitoring of adverse events (AEs as defined in the ISAs)	For the duration of the treatment course and a defined period after the treatment finishes (see ISAs)
Qualitative sub-study	1. To explore patients' experiences of consulting, being tested and taking (trial) medication for possible COVID-19.<br>2. To explore healthcare professionals' views of taking part in research during pandemics.	1. Telephone interviews with patients.<br>2. Telephone interviews with healthcare professionals.	1. After 28 days.<br>2. Once practice has completed recruitment.
Intervention(s)	All trial interventions are detailed in the Appendices. Further interventions may be added or replaced during the course of the trial, subject to suitable interventions becoming available and all necessary approvals being obtained.		
Comparator	In the first instance, this will be a two-arm trial, with the intervention arm being usual care plus a trial drug and the comparator being usual care. There will be no placebo control in this study. Additional arms may be added as the trial progresses. These will be detailed in the Appendices. If an intervention arm is shown to be superior, then this will become the new standard of care. However, the primary analysis of subsequent interventions will correspond to the comparison versus the original Usual Care arm.		

© Copyright: The University of Oxford and Oxford University Hospitals NHS Foundation Trust 2019

D. Adverse Events

Definitions

Adverse Event (AE)	Any untoward medical occurrence in a participant to whom a medicinal product has been administered, including occurrences which are not necessarily caused by or related to that product.
Adverse Reaction (AR)	An untoward and unintended response in a participant to an investigational medicinal product which is related to any dose administered to that participant.<br><br>The phrase "response to an investigational medicinal product" means that a causal relationship between a trial medication and an AE is at least a reasonable possibility, i.e. the relationship cannot be ruled out.<br><br>All cases judged by either the reporting medically qualified professional or the Sponsor as having a reasonable suspected causal relationship to the trial medication qualify as adverse reactions.
Serious Adverse Event (SAE)	A serious adverse event is any untoward medical occurrence that:<br>• results in death<br>• is life-threatening<br>• requires inpatient hospitalisation or prolongation of existing hospitalisation<br>• results in persistent or significant disability/incapacity<br>• consists of a congenital anomaly or birth defect*.<br><br>Other 'important medical events' may also be considered a serious adverse event when, based upon appropriate medical judgement, the event may jeopardise the participant and may require medical or surgical intervention to prevent one of the outcomes listed above.<br><br>NOTE: The term "life-threatening" in the definition of "serious" refers to an event in which the participant was at risk of death at the time of the event; it does not refer to an event which hypothetically might have caused death if it were more severe.<br><br>*NOTE: Pregnancy is not, in itself an SAE. In the event that a participant or his/her partner becomes pregnant whilst taking part in a clinical trial or during a stage where the foetus could have been exposed to the medicinal product (in the case of the active substance or one of its metabolites having a long half-life) the pregnancy should be followed up by the investigator until delivery for congenital abnormality or birth defect, at which point it would fall within the definition of "serious".

© Copyright: The University of Oxford and Oxford University Hospitals NHS Foundation Trust 2019

Serious Adverse Reaction (SAR)	An adverse event that is both serious and, in the opinion of the reporting Investigator, believed with reasonable probability to be due to one of the trial treatments, based on the information provided.
Suspected Unexpected Serious Adverse Reaction (SUSAR)	A serious adverse reaction, the nature and severity of which is not consistent with the Reference Safety Information for the medicinal product in question set out:<br>• in the case of a product with a marketing authorisation, in the approved summary of product characteristics (SmPC) for that product<br>• in the case of any other investigational medicinal product, in the approved investigator's brochure (IB) relating to the trial in question.

NB: To avoid confusion or misunderstanding the difference between the terms "serious" and "severe", the following note of clarification is provided: "Severe" is often used to describe intensity of a specific event, which may be of relatively minor medical significance. "Seriousness"

E. Data Recording and Record Keeping

The data will be entered into the CRFs in an electronic format by the participant, Trial Partner or trial team (using OpenClinica™ database via Sentry). OpenClinica™ is stored on a secure server – data will be entered in a web browser and then transferred to the OpenClinica Database by encrypted (Https) transfer. OpenClinica™ meets FDA part 11B standards. This includes safety data, laboratory data and outcome data. Safety data will also be collected through electronic diaries which are stored on a secure server.

Sentry is an online secure data entry system developed in-house at PC-CTU and hosted at Oxford. It is designed to collect sensitive data, such as participant and Trial Partner contact details, and securely retain them separate form a trial's clinical data. Sentry can also act as a central participant portal to manage online eligibility, eConsent and ePRO - acting as an intermediary between the participant and the clinical databases. Sentry is accessed via a secure HTTPS connection and all stored sensitive data is encrypted at rest to AES-256 standards. Participant and Trial Partner data will be kept and stored securely for as long as it's required by the study and reviewed on annual basis.

F. Qualitative Sub-study

With consent, participants will be contacted for a telephone interview within three months after they complete their day 28 follow up. The researcher will provide study information over the telephone and the Interview Patient PIS, and ICF will be available on the study website and emailed to participants if requested.

© Copyright: The University of Oxford and Oxford University Hospitals NHS Foundation Trust 2019

Once a practice has completed patient recruitment and one of their patients has been interviewed, we may ask 1-2 healthcare professionals who would be willing to share their experiences of taking part in the trial. Healthcare professionals will include clinicians and non-clinicians with the main criteria for inclusion in interviews being that HCP participants should have carried out trial activities in their practice. Potential HCP participants will be contacted in person or by email by the practice contact. They will be provided with the Interview HCP Invitation Email, Interview HCP PIS and Interview HCP ICF by email.

Patients recruited to both the intervention and usual care arms will be purposively sampled across the recruiting period with approximately 15-20 patients in each arm (30-40 interviews in total). We will seek to obtain maximum variation in age and symptom severity (as reported in daily diary at baseline). When the research team receives responses from HCPs, they will collect basic demographics to purposively select participants based on practice location, practice size, practice patient recruitment and job role. We aim to complete 20-25 interviews with HCPs.

All participants will only be required to take part in a single interview. Patient participant interviews will follow a semi-structured topic guide (Interview Patient Topic Guide) and ask about reasons for consulting and illness perceptions prior to the consultation, experiences of the consultation, the COVID-19 testing process (if applicable, and result if the participant has been notified) and medication adherence. The topic guide will be informed by the Common Sense Model which describes how people perceive and cope with symptoms of illness.
HCP interviews will follow the Interview HCP Topic Guide and will ask about experiences of carrying out trial activities, recruiting patients and the work required to set up a clinical trial during a pandemic.

Interviews with patient participants are expected to last approximately 30-45 minutes and interviews with HCPs are expected to last 15-30 minutes.

Data Collection:
Each interview will be audio-recorded with the participant’s permission. Recordings will allow verbatim transcription of interviews. Transcription will be completed by an independent transcription company. Once transcribed and transcripts are checked, audio-recordings will be deleted. Transcripts will be labelled with a unique participant number and will omit any identifiable data either identifying the participant or their general practice.

© Copyright: The University of Oxford and Oxford University Hospitals NHS Foundation Trust 2019

Primary Care
Clinical Trials Unit

PRINCIPLE
Platform Randomised trial of INterventions against COVID-19 In older peoPLE

MASTER STATISTICAL ANALYSIS PLAN

Platform Randomised trial of INterventions against COVID-19 In older peoPLE

Internal Reference Number / Short title: PRINCIPLE

Ethics Ref: 20/SC/0158

IRAS Project ID: 281958

EudraCT Number: 2020-001209-22

Based on protocol version 7.1 22nd February 2021

Version 3.1 Date: 30th March 2021

Version History

	Version Date:	Signature	Date
Written by	Jill Mollison		30th March 2021
Reviewed by	Ly-Mee Yu		31st March 2021
Reviewed by	Ben Saville		30th March 2021
Approved by:	Professor Chris Butler		30th March 2021

Version History

Version:	Version Date:	Changes:
0.1	25th March 2020	MS initial draft
0.2	31st March 2020	Updated post JM initial comments
0.3	12 May 2020	JM/UG writing SAP
0.4	10 Aug<br>4th August 2020	Updated to protocol version 4.0<br>NW updating SAP
0.5	11th August 2020	Merging of 2 0.4 versions
0.6	1st October 2020	Updates following comments from JG, ST and CB<br>Update primary outcomes and sample size<br>Remove references to RCGP data as this is stored in the notes review
0,7	15th October 2020	Updates following meetings with JG, ST, LMY<br>Remove much of text from introduction/DMC sections and refer to protocol/DMC charter<br>Update analysis of binary outcomes to logistic regression
0.8	29th October 2020	Updates following review from LMY<br>Health service use split into participant reported and GP reported health service use<br>Addition of sustained recovery outcome<br>Addition of appendix III – analysis of HYDROXYCHLOROQUINE arm
0.9	30th October 2020	Further update to definition of sustained recovery. Also changed the definition of duration of severe symptoms.
0.10	3rd November 2020	Update following comments from CB

0.11	4th November 2020	Incorporated Jienchi Dorward & Ben Saville's comments
0.12	6th November 2020	Incorporated Richard Hobbs' comments and made changes following meeting with LMY, RH, CB, BS, JD:<br><br>Updated definition of sustained recovery<br>Change 'consumption' to 'prescription' for antibiotics<br>Addition of WHO ordinal scale
0.13	18th November 2020	Amending typos<br><br>Addition of appendices detailing analysis of azithromycin and doxycycline<br><br>Addition of subgroup analyses
0.14	24th November 2020	Update to protocol v6.0<br>New outcome: new infections in the household<br>Addition of appendix detailing analysis of inhaled corticosteroid arm<br><br>Update subgroup analysis section<br><br>Accept changes from BS and CB from previous version
0.15	26th November 2020	Updates following comments from Philip Hannaford – update to ICS dose and subgroup analyses to include deaths and deaths/hospitalisation combined
1.0	1st December 2020	Change to version 1.0 for signing off
1.1	20th January 2021	Remove viral shedding outcome<br>Update timing of swab results<br>Clarification of censoring for time to event outcomes

		Addition of moderation analyses
1.2	9th February 2021	Updated objectives to protocol version 6.3 30.12.2020.<br>Inclusion of Addendum 1.1 and clarification of analysis populations.<br>Updated derivation of: hospitalisation primary outcome (hierarchy of data sources); duration of hospital admission;<br>Definition of last contact date for time to event outcomes<br>Schedule of procedures and flow diagram of trial participants updated<br>Updated timing of swab results<br>Updated handling missing data
2.0	10th February 2021	Signatures
2.1	26th February 2021	Updated to reflect protocol V7.0, addition of colchicine, target population, Appendices. Addition of new secondary outcome
2.2	16th March 2021	Updated to reflect protocol V7.1. Addition of safety as a secondary endpoint. Addition of favipiravir treatment arm. The primary analysis population defined as those with a COVID-19 positive test.
3.0	16th March 2021	Change to version 3.0 for signing
3.1	30th March 2021	Addition of sensitivity analysis specific to Budesonide. Clarification of analysis population for sensitivity analysis. Definition of Vaccination status and addition as covariate, update to definition of sustanined

		recovery (secondary outcome). Moved derivation of COVID-19 test status to section 2.2.4

CORRECTION (12 July 2021):

On Page 23 of the this M-SAP,

Sustained recovery will be derived as being recovered within the first 14 days and reports feeling recovered for the next 14 days. Where the participant has only call data, they require to have reported feeling recovered at both 14 days and 28 days. Where participants have got incomplete diary data for days 14-28 and no call data at 28 days, they are classified as otherwise.

This definition was referred to the definition of Early sustained recovery and will be update in the new version of the M-SAP to

Early sustained recovery will be derived as being recovered within the first 14 days and reports feeling recovered for the next 14 days. Where the participant has only call data, they require to have reported feeling recovered at both 14 days and 28 days. Where participants have got incomplete diary data for days 14-28 and no call data at 28 days, they are classified as otherwise.

TABLE OF CONTENTS

MASTER STATISTICAL ANALYSIS PLAN 1

TABLE OF CONTENTS 6

1 INTRODUCTION 10

1.1 PREFACE 10
1.2 PURPOSE AND SCOPE OF THE PLAN 10
1.3 TRIAL OVERVIEW 11
1.4 OBJECTIVES 11

2 TRIAL DESIGN 15

2.1 ADAPTIVE DESIGN 15
2.2 DEFINITIONS 15
2.2.1 Follow-up period 15
2.2.2 Last contact date 15
2.2.3 Duration of illness prior to randomisation 16
2.2.4 Derivation of COVID-19 test status 16
2.2.5 Vaccination status 16
2.3 OUTCOMES 16
2.3.1 Primary outcome 16
2.3.2 Secondary outcomes 16
2.4 TARGET POPULATION 18
2.5 TREATMENTS 18
2.6 SAMPLE SIZE 18
2.7 RANDOMISATION AND BLINDING IN THE ANALYSIS STAGE 19

3 DATA MANAGEMENT AND DERIVATION OF OUTCOMES 20

3.1 SOURCES OF DATA 20
3.2 PRIMARY OUTCOMES 20
3.2.1 Time to recovery 20
3.2.2 Hospital admission or death 21
3.3 SECONDARY OUTCOMES 22
3.3.1 Patient reported illness severity 22
3.3.2 Duration of severe symptoms 22
3.3.3 Contacts with health services between randomisation and day 28 of follow-up. 24
3.3.4 Prescribing of antibiotics between randomisation and day 28 of FU 25
3.3.5 Hospital assessment without admission 25
3.3.6 Oxygen administration 26
3.3.7 Intensive care unit admission 26
3.3.8 Mechanical ventilation. 27
3.3.9 Duration of hospital admission 27
3.3.10 Negative effect on well-being (WHO-5) 28
3.3.11 WHO Ordinal Scale of Clinical Progression 28
3.3.12 New infections in the household 28

3.3.13 Safety of treatments not licensed in the uk 28

4 ANALYSIS – GENERAL CONSIDERATIONS 29

4.1 PARTICIPANT THROUGHPUT 29
4.2 DESCRIPTIVE STATISTICS 29
4.3 CHARACTERISTICS OF PARTICIPANTS 29
4.4 DESCRIPTION OF AVAILABLE DATA 30
4.5 COMPLIANCE WITH MEDICATION 30
4.6 DEFINITION OF POPULATION FOR ANALYSIS 30
4.6.1 Primary Analysis Population 30
4.6.2 Concurrent Randomised and Eligible Analysis population 31
4.6.3 Concurrent Randomisation Analysis Population 31
4.6.4 Safety Analysis Population 31
4.7 COMPARATIVE ANALYSIS 31
4.8 POOLING OF INVESTIGATIONAL SITES 31
4.9 DATA MONITORING COMMITTEE AND INTERIM ANALYSES 31

5 PRIMARY ANALYSIS 31

5.1 PRIMARY OUTCOMES 32
5.1.1 Summary of Bayesian Analyses to be conducted by Statistical Analysis Committee 32

6 SECONDARY ANALYSIS 32

6.1 PRIMARY OUTCOME ON OVERALL POPULATION 32
6.2 SECONDARY OUTCOMES 33
6.2.1 Patient reported illness severity 33
6.2.2 Duration of severe symptoms 33
6.2.3 Time to Sustained recovery 33
6.2.4 Time to Sustained symptoms alleviation 33
6.2.5 Contacts with health care services 34
6.2.6 Prescribing of antibiotics 34
6.2.7 Hospital assessment without admission 34
6.2.8 Oxygen administration 34
6.2.9 Intensive care unit admission 34
6.2.10 Mechanical ventilation 35
6.2.11 Duration of hospital admission 35
6.2.12 Well-being 35
6.2.13 WHO Ordinal Scale of Clinical Progression 35
6.2.14 New infections in the Household 35
6.2.15 Safety of treatments not licensed in the uk 36
6.3 HANDLING MISSING DATA 36
6.4 MULTIPLE COMPARISONS AND MULTIPLICITY 36
6.5 MODEL ASSUMPTIONS 36

7 SENSITIVITY ANALYSIS OF CO-PRIMARY OUTCOMES USING CONCURRENT RANDOMISED ANALYSIS POPULATION 36

8 SUBGROUP ESTIMATES 36

9 MODERATION ANALYSIS 36

10 SAFETY ANALYSIS 37

10.1 ADVERSE EVENTS 37

11 VALIDATION 37

12 CHANGES TO THE PROTOCOL OR PREVIOUS VERSIONS OF SAP 37

13 APPENDICES 39

13.1 APPENDIX I. SCHEDULE OF PROCEDURES 39
13.2 APPENDIX II. FLOW DIAGRAM OF TRIAL PARTICIPANTS 42
13.3 APPENDIX III: PLANS FOR ANALYSIS OF HYDROXYCHLOROQUINE 43
Population of Analysis 43
Descriptive statistics 43
Primary Outcome Analysis 43
Specific sensitivity and secondary analysis of Primary Outcome 43
Concurrent Randomisation analysis population 43
SECONDARY OUTCOME ANALYSIS 44
Analysis of Safety Data 44
13.4 APPENDIX IV: PLANS FOR ANALYSIS OF AZITHROMYCIN 45
Population of Analysis 45
Descriptive statistics 45
Primary Outcome Analysis 45
Specific sensitivity and secondary analysis of Primary Outcome 45
Concurrent Randomisation analysis population 45
Secondary Outcome Analysis 45
Analysis of Safety Data 45
13.5 APPENDIX V: PLANS FOR ANALYSIS OF DOXYCYCLINE 46
Population of Analysis 46
Descriptive statistics 46
Primary Outcome Analysis 46
Specific sensitivity and secondary analysis of Primary Outcome 46
Concurrent Randomisation analysis population 46
Secondary Outcome Analysis 46
Analysis of Safety Data 46
13.6 APPENDIX VI: PLANS FOR ANALYSIS OF INHALED CORTICOSTEROID BUDESONIDE 47
Population of Analysis 47
Descriptive statistics 47
Primary Outcome Analysis 47
Specific sensitivity and secondary analysis of Primary Outcome 47
Secondary Outcome Analysis 47
Analysis of Safety Data 47
13.7 APPENDIX VII: PLANS FOR ANALYSIS OF COLCHICINE 48
Population of Analysis 48
Descriptive statistics 48
Primary Outcome Analysis 48

Specific sensitivity and secondary analysis of Primary Outcome *48*
Secondary Outcome Analysis *48*
Analysis of Safety Data *48*
13.8 APPENDIX VIII: PLANS FOR ANALYSIS OF FAVIPIRAVIR 49
Population of Analysis *49*
Descriptive statistics *49*
Primary Outcome Analysis *49*
Specific sensitivity and secondary analysis of Primary Outcome *49*
Secondary Outcome Analysis *49*
Analysis of Safety Data *49*

1 Introduction

1.1 Preface

Chief Investigator: Professor Chris Butler

Co-study lead: Professor Richard Hobbs

Senior Trial Manager(s): Dr Hannah Swayze, Dr Emma Ogburn, Dr Emily Bongard, Julie Allen

Trial Manager: Jared Robinson

Data manager: Jenna Grabey

Lead Trial Statistician(s): Dr Ly-Mee Yu and Dr Ben Saville

There are two teams of unblinded statisticians involved in the PRINCIPLE trial. The unblinded statisticians in Oxford Primary Care Clinical Trials Unit (PC-CTU) are responsible for data management, derivation of outcomes, transfer of data to the Statistical Analysis Committee (SAC) of Berry Consultants and analysis of the secondary and safety outcomes. The unblinded SAC is responsible for the interim analysis and to provide a summary of results for the Data Monitoring Safety Monitoring Committee (DMSC) members in an interim analysis report. The SAC will be responsible for the co-primary analyses and any related sensitivity and subgroup analyses requiring a similar Bayesian framework.

This version of the Statistical Analysis Plan was written based on protocol version 7.1 22nd February 2021, and may be updated in the light of further amendments to the study protocol.

1.2 Purpose and scope of the plan

This Master Statistical Analysis Plan (M-SAP) will detail the statistical design and methods of the PRINCIPLE trial. It will include an appendix titled “Adaptive Design Report” (ADR), which will provide complete specifications for the primary analyses and pre-specified adaptive algorithm. In addition, the M-SAP will be accompanied by arm-specific appendices to describe any planned deviations from the M-SAP. Plans for the analysis of qualitative outcomes is beyond the scope of this statistical analysis plan, and therefore will not be covered in this M-SAP.

Analyses-related decisions may need to be made based on the observed data, such as a review of the distribution of outcome data. These decisions will be made prior to the proposed statistical analyses.

The plan draws on statistical guidance ICH Harmonised Tripartite Guideline: Statistical Principles for Clinical Trials and PSI Guidelines for the Standard Operating Procedures (SOP) for Good Statistical Practice in Clinical Research, the CONSORT statement for operating trials and PC-CTU statistical SOPs.

Analyses will be carried out in accordance with the M-SAP and corresponding appendices. Any additional analysis that is not specified in the M-SAP/appendices or any unplanned deviation(s) from the M-SAP/appendices will be specified in the Statistical Report. Reasons for these changes will be documented and authorised by the Chief Investigator.

Due to the nature of the design of this trial, results for specific treatments will be analysed while the trial is ongoing. This will be done with prior agreement from the Trial Steering Committee (TSC) and DMSC, and the trial team will remain blind to these analyses until such time as the TSC, informed by data and advice from the DMSC, advise that findings should be declared.

1.3 Trial overview

PRINCIPLE is an open, adaptive, platform trial to evaluate emerging treatments for COVID-like-illness. A "platform trial" is a trial in which multiple treatments for the same disease are tested simultaneously. The backbone of the trial is an adaptive clinical trial design. Pre-specified decision criteria allow for dropping a treatment for futility, declaring a treatment superior, or adding a new treatment to be tested. If at any point a treatment is deemed superior to the Usual Care arm, the superior treatment may replace the Usual Care arm as the new standard of care within the trial. However, the primary analysis of subsequent interventions will correspond to the comparison versus the original Usual Care arm. Because the process of dropping and adding treatments may be on-going for an indefinite period of time, platform trials may be better conceived of as a process rather than a single clinical trial. In the context of the COVID-19 pandemic, the trial may continue as long as the pandemic persists.

The PRINCIPLE trial began as a two arm, 1:1 randomised trial but with the capability to add additional interventions over time. The evaluation of any new interventions is governed by the master protocol and M-SAP (including adaptive algorithm and decision criteria), with any planned deviations from the master protocol and M-SAP to be specified in arm-specific appendices. The inclusion of any new interventions will require additional arm-specific appendices to the master protocol and M-SAP.

1.4 Objectives

The primary and secondary objectives as well as time points to evaluate these outcome measures as stated in the protocol.

	Objectives	Outcome Measures	Timepoint (s)
Primary	To assess the effectiveness of trial treatments in reducing<br><br>1) Time to recovery, for patients	1) Time to self-reported recovery, defined as the first instance that a participant reports feeling recovered from possible COVID-19, and	Within 28 days of randomisation<br><br>Patient report, Study Partner report, daily online symptom scores
	2) Hospitalisation and/or death.	2) Hospitalisation and/or death	Within 28 days of randomisation<br><br>Patient report, Study Partner report, medical records
Secondary	To explore whether trial treatment will affect<br><br>1) participant-reported illness severity reported by daily rating of how well participant feels<br>2) Duration of severe symptoms and symptom recurrence	Participant reports of daily and monthly (after 28 days) symptoms.	Daily online symptom scores.<br><br>Telephone call or text on days 2, 7, 14 and 28 and once a month for 12 months if data is not obtained through the online diary
	3) Contacts with the health services	Contacts with health services reported by patients and/or captured by reports in patients' medical records if the practice is a member of the RCGP RSC network	GP notes review if available through Oxford RCGP RSC network; otherwise, other sources of routinely collected data after 28 days. Medical notes review for up to 10 years.
	4) Prescription of antibiotics	Bi-weekly reports from participants' primary care medical records	

	Objectives	Outcome Measures	Timepoint (s)
	5) Hospital assessment without admission<br>6) Oxygen administration<br>7) Intensive Care Unit admission<br>8) Mechanical ventilation<br>9) Duration of hospital admission	Patient report/carer report/medical record in primary and secondary care	HES/ONS/EMIS/Medical record data linkage after 28 days if patients have been assessed in hospital
	10) Negative effects on well being	WHO-5 Well Being Index	WHO 5 Well Being Indat baseline, day 14, and day 28 and monthly for up to 12 months, either via online diary or telephone
	11) New infections in household	Reports of new infections in the household (from daily questionnaire)	Within 28 days of randomisation
	12) To determine if effects are specific to those with a positive test for SARS-CoV-2	Swab test results will indicate an "Intention to Treat Infected" group within the overall cohort for sub analysis. Blood test results on recovery (optional) for evidence of historic COVID-19	Swab result from medical records, the supporting laboratory and/or convalescent blood test for evidence of historic COVID-19
	13) To investigate the safety of treatments that are not licensed in the UK	Evaluation of overall safety of drugs by the monitoring of adverse events (AEs as defined in the Intervention specific Appendices (ISAs))	For the duration of the treatment course and a defined period after the treatment finishes.
Qualitative sub-study<br><br>(not covered in this M-SAP)	1. To explore patients' experiences of consulting, being tested and taking	1. Telephone interviews with patients.	1. After 28 days.

	Objectives	Outcome Measures	Timepoint (s)
	(trial) medication for suspected COVID-19.<br><br>2. To explore healthcare professionals' views of taking part in research during pandemics.	2. Telephone interviews with healthcare professionals.	2. Once practice has completed recruitment.
Intervention(s)	All trial interventions are detailed in the Appendices of the protocol. Further interventions may be added or replaced during the course of the trial, subject to suitable interventions becoming available and all necessary approvals being obtained.		
Comparator	PRINCIPLE began as a two-arm trial, with the intervention arm being Usual Care without the addition of a trial drug. There will be no placebo control in this study. Additional arms may be added as the trial progresses. These will be detailed in the Appendices of the protocol. If an intervention arm is shown to be superior, then this will become the new standard of care. However, the primary analysis of subsequent interventions will correspond to the comparison versus the original Usual Care arm.		

2 Trial design

PRINCIPLE is an open, adaptive, platform trial to evaluate emerging treatments of COVID-like-illness. A "platform trial" is a trial in which multiple treatments for the same disease are tested simultaneously. The backbone of the trial is an adaptive clinical trial design. Pre-specified decision criteria allow for dropping a treatment for futility, declaring a treatment superior, or adding a new treatment to be tested. If at any point a treatment is deemed superior to the control arm, the superior treatment will replace the control arm as the new standard of care, and all subsequent treatments may be compared to the new standard of care within the trial. Because the process of dropping and adding treatments may be on-going for an indefinite period of time, platform trials may be better conceived of as a process rather than a single clinical trial. In the context of the COVID-19 pandemic, the trial may continue as long as the pandemic persists, and while there is a need to evaluate treatments for acute respiratory tract infections in the community.

The PRINCIPLE trial began as a 1:1 randomised trial of standard care versus standard care plus hydroxychloroquine but with the capability to add additional interventions over time. The evaluation of any new interventions is governed by the master protocol, including adaptive and decision criteria. In addition, the inclusion of any new interventions will require amendments and/or supplements to the protocol and M-SAP.

2.1 Adaptive design

The pre-specified design will allow adaptations to the trial based on the observed data. These adaptations include the declaration of success or futility of an intervention at an interim analysis, the addition or removal of treatment arms, and changes in the randomisation probabilities. Adaptations will occur at a given interim analysis if pre-specified conditions are satisfied. The adaptive algorithm will be documented in the Adaptive Design Report, including pre-specified criteria for decisions regarding futility or effectiveness of interventions and/or replacing interventions in the trial.

2.2 Definitions

2.2.1 Follow-up period

Patients will be followed from date of randomisation to 28 days by daily symptom diary and/or telephone, and review of their medical records. Participant records will be accessed up to 3 months following randomisation to ascertain outcome data up to 28 days from randomisation. Participants will be followed up on a monthly basis for up to 12 months after enrolment (via email, text message or phone call) to collect information about any ongoing symptoms, hospitalisations and well-being.

2.2.2 Last contact date

In the derivation of time to event outcomes, the last contact date will be defined as the maximum of;

- Last diary entry
- Last call CRF completed
- Date of hospital admission
- Date of hospital discharge

2.2.3 Duration of illness prior to randomisation

Duration of illness prior to randomisation will be derived using the date the participant reported starting to feel unwell (Screening CRF) and date of randomisation. If date patient started to feel unwell (screening CRF) is missing, the date the screening form was completed will be used instead.

2.2.4 Derivation of COVID-19 test status

Participants will be categorised as being infected or not, based on any swab result obtained 14 days or less prior to randomisation and up to and including 7 days following randomisation. Swab result data is collected in the screening CRF, baseline CRF, Lab results CRF and notes review CRF.

Any swab result obtained 14 days or less prior to randomisation and up to and including 7 days following randomisation will be used to classify participants into three groups. A positive test result at any time will put them into group 1, regardless of other negative results within the window specified;

1. COVID-19 positive test result
2. COVID-19 Negative test result
3. No swab result available

2.2.5 Vaccination status

Vaccination status will be categorized as “Yes" vs. “other", where “yes" indicates at least one COVID-19 vaccination dose was given prior to randomisation and “other" indicates either no vaccination was previously given or no vaccine information is available.

2.3 Outcomes

2.3.1 Primary outcome

There are two co-primary outcomes as listed below:

- Time to recovery from suspected COVID-19 infection within 28 days from randomisation, where recovery is defined as the first instance that a participant reports feeling recovered.
- Hospital admission or death related to suspected or confirmed COVID-19 within 28 days from randomisation

2.3.2 Secondary outcomes

2.3.2.1 Patient reported illness severity

Participants are asked to rate how well they are feeling overall each day on a scale of 1-10 (1 being the worst and 10 being the best). This is captured on the patients’ daily diaries and the Call CRF.

2.3.2.2 Duration of severe symptoms and symptom recurrence

Participants are asked to rate their symptoms of fever, cough, shortness of breath, muscle ache, and nausea/vomiting on a four point scale from 0=no problem, 1=mild problem, 2=moderate problem and 3=major problem.

This is captured on patients' daily diaries and Call CRF and is only collected for participants who responded "No" to "Do you feel recovered today?"

The outcomes to be analysed will be time to alleviation of symptoms, time to initial reduction in severe symptoms, time to sustained recovery and time to sustained alleviation of symptoms.

2.3.2.3 Contacts with health services between randomisation and day 28 of follow-up (FU).

This is captured on patients' daily diaries, telephone call CRFs, and medical notes review

2.3.2.4 Prescription of antibiotics between randomisation and day 28 of FU

Notes review at 28 days will record information regarding prescription of antibiotics. Hospital assessment without admission overnight

This is captured on patients' daily diaries, telephone call CRFs, notes review and hospitalisation and death CRF.

2.3.2.5 Oxygen Administration

This is captured in patients' daily diaries, telephone call CRF, Notes review and hospitalisation and death CRF.

2.3.2.6 Intensive care unit admission

This is captured in patients' daily diaries, telephone call CRF, Notes review, and hospitalisation and death CRF.

2.3.2.7 Mechanical ventilation

This is captured in patients' daily diaries, telephone call CRF, Notes review and hospitalisation and death CRF.

2.3.2.8 Duration of hospital admission

Patient report/carer report/medical record in primary care and hospital care in relation to duration of hospital admission between date of randomisation and day 28 of follow-up.

This is captured in patients' daily diaries, Call CRF, Notes review and hospitalisation and death CRF.

2.3.2.9 Negative effects on well-being (WHO-5)

The WHO-5 well-being index is collected from daily diaries or telephone call at baseline, day 14 and day 28, and monthly for up to 12 months.

2.3.2.10 New infections in the Household

This is collected in the daily diary and telephone call CRF through the question "has anybody else in your house become unwell today with a respiratory illness?"

2.3.2.11 Safety of treatments not licensed in the UK

Medication side-effects and SAEs will be collected from participant daily diaries, calls to participants/study partners, medical records, notes reviews and RCGP data downloads.

2.4 Target population

The trial aims to include symptomatic participants with confirmed, or possible COVID-19 who meet the current NHS case definition for possible COVID-19, and who are well enough to remain in the community. This definition can be found here:
https://www.nhs.uk/conditions/coronavirus-covid-19/symptoms/.
Participants must be aged 65 and over, OR aged 18 to 64 and experiencing shortness of breath as part of COVID-19 illness, OR aged 18-64 with certain comorbidities.

Participants experiencing shortness of breath have a greater risk of severe and critical disease outcomes with COVID-19.

The study is for people who have ongoing symptoms.

See protocol for detailed inclusion and exclusion criteria.

2.5 Treatments

Based on version 7.1 of the protocol the main randomisation will be between the following treatment arms (although not all treatments may be available at any one time and not all participants are eligible for all treatments). However, the trial design accommodates treatments being added and dropped as appropriate.

- Usual care
- Usual care plus hydroxychloroquine, 200mg twice daily for 3 days (discontinued)
- Usual care plus azithromycin, 500mg once daily for 3 days (discontinued)
- Usual care plus doxycycline, 200mg on day 1 followed by 100mg daily for 6 days (discontinued)
- Usual care plus the inhaled corticosteroid, budesonide, 400mcg daily (as two puffs bd) for 14 days
- Usual care plus colchicine, 500 microgram (µg) once daily for 14 days Usual care plus favipiravir 1800mg twice a day on day one, and then 800mg twice a day for four days.

Subsequent reference to a treatment group refers to treatment plus usual care, and subsequent reference to usual care group refers to the usual care without a study drug.

2.6 Sample size

Given the open perpetual trial structure, the trial does not have a prespecified end based on sample size. Rather, the trial will continue until either superiority or futility is claimed for each intervention,

or until the pandemic expires in the population. We estimate that approximately 400 participants per arm (800 participants total if only a single intervention vs. Usual Care) will be required to provide 90% power for detecting a hazard ratio of 1.3 in the primary population (approximate difference of 2 days in median recovery time). This calculation is based on the assumption of an exponential distribution for time to recovery with a median of 9 days in the Usual Care arm, with some adjustments for missing data and multiple interim analyses. On average, we expect fewer participants to be required when there is a large treatment benefit or complete lack of benefit. For example, if the true hazard ratio is 1.5 (3 day benefit in median time to recovery), on average only 150 subjects per arm are required to provide sufficient power. The primary advantage of the adaptive design is the ability to adapt the sample size to the observed data, thus addressing the primary hypothesis as quickly and as efficiently as possible.

We estimate that approximately 1500 participants per arm (3000 participants total if only a single intervention vs. usual care) will be required to provide 90% power for detecting a 50% reduction in the relative risk of hospitalisation and/or death in the primary analysis population. This calculation is based on the assumption of an underlying 5% combined hospitalisation and/or death rate in the Usual Care arm, with an intervention lowering the hospitalisation and/or death rate to 2.5%, with some adjustments for the multiple interim analyses. We expect fewer participants to be required to detect a 50% reduction if the event rate in the Usual Care arm is greater than 5%.

Virtual trial simulations

Because of the adaptive platform trial structure, there exists no simple formula(s) to calculate power and Type I error (false positive rate). Hence, virtual trial simulations will be used to fully characterize and quantify the power and Type I error of the design. These simulations will be conducted prior to the first interim analysis (with results described in the Adaptive Design Report), and will be used to optimize the adaptive decision criterion and RAR parameters. The simulations will include a comprehensive evaluation of trial performance across a wide range of assumptions (e.g. underlying distribution of outcome in Usual Care arm, treatment effect, accrual rates, etc.). This will include summaries regarding the number of subjects required to make a success or futility conclusions for each intervention. Complete details of the simulations will be provided in the Adaptive Design Report.

2.7 Randomisation and blinding in the analysis stage

Participants will be randomised using a fully validated and compliant web-based randomisation system called Sortition. Once deemed eligible, the clinician or a member of the trial team will randomise the participant. The randomisation process will take only a few moments via the online system and will not delay trial participation. Participants will be randomised to the arm/arms they are eligible for (at least two arms, usual care and at least one intervention), automatically by Sortition.

Initially, randomisation will be fixed 1:1 for a comparison between two trial arms, with stratification by age (less than 65, greater than or equal to 65), and comorbidity (yes/no). If a second intervention arm is added to the study, randomisation allocation will be modified and the additional intervention will be included in the interim analyses (with evaluation for success and futility) as detailed in the Adaptive Design Report. If there are at least 3 arms (2) treatment and Usual Care) in the study, each interim analysis may incorporate modified randomisation probabilities via response adaptive

randomisation (RAR). Full details for implementing RAR will be provided in the Adaptive Design Report; the general idea is to allocate more participants to the intervention arms that have the best observed outcomes. Except for the CTU programmer, the rest of the trial team are blinded to RAR ratios.

PRINCIPLE is an open-label trial. The participant, a person guiding the participant through the randomisation process, and the participants' primary care clinician will know the participant's allocation. Therefore, no unblinding or code breaking is required in the event of a relevant emergency. However, those managing the data will be blind to participant allocation.

The trial team and recruiting clinicians will be blinded to emerging results. During the course of the trial, only the unblinding statisticians and independent members of the Data Monitoring and Safety Committee will have access to the unblinded interim results.

3 Data management and Derivation of outcomes

3.1 Sources of Data

Data is collected from multiple sources. For the derivation of outcomes, should data be collected from more than one source, we will specify which source should be utilised. For example, if data for an outcome are obtained from both daily diary and telephone calls, the daily diary data will be utilised first.

(i) Death and hospitalisation CRF
(ii) Online daily diaries for 28 days, and then monthly up to 12 months
(iii) Telephone call CRF at day 7, 14 and 28, and then monthly up to 12 months
(iv) Lab results CRF
(v) Notes review CRF
(vi) When available, the Secondary Uses Services data, which is a collection of healthcare data in England provided by NHS Digital, and other sources of hospital data.

Data management for derivation of primary and secondary outcomes will be carried out by PC-CTU statisticians, and primary outcomes (and any data that are relevant to the analysis of the primary outcomes) data transferred to SAC. The PC-CTU unblinded statistician will ensure that data transfer to the SAC for each treatment comparison includes only participants that meet the definition of the analysis population as defined in Section 4.6.

3.2 Primary outcomes

3.2.1 Time to recovery

The first primary outcome is time to recovery from suspected COVID-19 infection within 28 days from randomisation, where recovery is defined as the first instance that a participant reports feeling recovered.

Time (in days) taken to self-reported recovery will be computed as time to reported "Yes" to the question "Do you feel recovered today (I.e. symptoms associated with illness are no longer a problem)?". The variable is recorded as (**WELLYN**=1). This will be calculated as date from randomisation to date (VISDAT_P) of participant self-report of recovery if using the patient diary. If the call CRF is being used then the date to feeling fully recovered is '**WELLDAT**'. Where patient recall of date of feeling recovered (as recorded on the call CRF) is prior to date of randomisation, the date of feeling recovered will be assumed to be day 0.

If a participant has more than one date for when they reported feeling recovered (i.e. relapse or more than one data source completed), the date of first reported recovery will be taken.

If the participant is in hospital on the date of feeling recovered then this will still be counted as a recovery (hospitalisation recorded in any 1 or more of the following sources: notes review; death and hospitalisation CRF; daily diaries; call CRF).

Participants who withdraw or are lost to follow-up will be censored at their last contact date (definition 2.2.2).

Participants who die will be censored at 28 days.

3.2.2 Hospital admission or death

The second primary outcome is hospital admission or mortality related to suspected or confirmed COVID-19 measured within 28 days from randomisation.

3.2.2.1 Derivation of mortality

Mortality related to suspected COVID-19 within 28 days of randomisation

Data collected via the death and hospitalisation CRF and the notes review will be used to derive the primary outcome. If the following is true from either source then the patient will be recorded as having death related to suspected COVID-19 within 28 days of randomisation.

DDYN =1 AND date of death (**DDDAT**) ≤28 days from randomisation AND COVID-19 contributed to death [**DD_CV19=1**].

If death is recorded as "Yes" but the COVID-19 related variable is missing, then the outcome will be cross checked with the SAE data. Any death not related to suspected COVID-19 should be recorded as an SAE.

3.2.2.2 Derivation of hospital admission

Hospital admission within 28 days will be defined as an overnight stay in hospital and likely to be related to COVID-19. This can be recorded from the daily diaries, calls at days 7, 14 and 28, notes review CRF, death and hospitalisation CRF and SUS data. The data sources will be considered in the following hierarchy; (1) Patient reported (daily diaries, calls, hospitalisation CRF), (2) Notes review, (3) SUS data. The SUS data will only be used if no data can be obtained from sources 1 and 2. SUS data will be adjudicated by two clinicians blind to randomised group as to whether the hospital admission

is likely to be related to COVID-19. Hospital admissions on the day of randomisation will be assumed to have occurred post randomisation and will be included in the analysis.

3.2.2.2.1 Death and hospitalisation CRF

Participant attended hospital [**HOSP_HOOCCUR = 1**] AND attendance likely related to COVID-19 [**CVYN** = 1] AND [number of days in hospital > 1 or not yet discharged [**HOSP_HOENYN=0**] AND date they were admitted to hospital (**HOSP_HOSTDAT**) is ≤28 days from date of randomisation.

3.2.2.2.2 Daily diary data

Have you attended hospital in the last 24 hours [**HOSP_HOOCCUR** = 1] AND attendance likely related to COVID-19 [**CVYN** = 1] AND admitted overnight [**HOSP_NIGHTYN** = 1] AND first date of overnight hospital stay (**HOSP_HOSTDAT**) is ≤28 days from date of randomisation.

3.2.2.2.3 Calls at 7, 14, 28 days

Admitted to hospital [**HOSP_HOOCCUR** = 1] AND was the admission related to suspected COVID-19 infection [**CVYN** = 1] AND admitted overnight [**HOSP_NIGHTYN** = 1] AND first date of overnight hospital stay (**HOSP_HOSTDAT**) is ≤28 days from date of randomisation.

3.2.2.2.4 Notes review

Participant attended hospital [**HOSP_HOOCCUR** = 1] AND attendance likely related to COVID-19 [**CVYN** = 1] AND admitted overnight [**HOSP_NIGHTYN**=1] AND date they went to hospital is ≤28 days from date of randomisation.

If either hospital admission (as derived above) OR mortality (as derived above) occurs then this primary outcome has occurred (Primary outcome = 1).

Early versions of online daily diaries and call CRF did not include a qualification for suspected COVID-19 infection so these will be cross checked with the SAE data. Any hospitalisation or death not thought to be related to suspected COVID-19 should be recorded as an SAE

3.3 Secondary outcomes

3.3.1 Patient reported illness severity

Participants are asked to rate how well they are feeling each day on a scale of 1-10 (1 being the worst and 10 being the best). This is captured on the participants' daily diaries and the Call CRF.

Day 7, 14 and 28 data will be obtained from the call CRF if not available in the daily diary.

Four variables will be derived; illness severity on day 7, illness severity on day 14, illness severity on day 21 and illness severity on day 28. If day 7, 14 or 28 is not available from the daily diaries, then data from the call CRF will be used. If no data available from diaries or calls on days 7, 14, 21 and 28 illness severity will be regarded as missing for that time point.

3.3.2 Duration of severe symptoms

Participants are asked to rate their symptoms of fever, cough, shortness of breath, muscle ache, nausea/vomiting, diarrhoea, and generally feeling unwell on a four point scale from 0=no problem, 1=mild problem, 2=moderate problem and 3=major problem until they feel recovered.

‘Severe’ symptoms will be defined as a score of 3 (major) on the four point rating scale.

3.3.2.1 Time to alleviation of symptoms

Time to alleviation of symptoms will be defined as the time from randomisation to symptoms being rated as mild or none. For those who have call data only, the time to alleviation will be defined as the day the call was made. For those who had symptoms rated at baseline as mild or none but reported moderate or major symptoms later, the time to alleviation of symptoms will be from the date of randomisation until those symptoms were rated as mild or severe. Patients who report none/mild symptoms at baseline (with no report of moderate or severe on any day will be censored at time 0. Participants who withdraw or are lost to follow-up will be censored at their last contact date (definition 2.2.2).

3.3.2.2 Time to initial reduction of severity of symptoms

Time to initial reduction of severity of symptoms will be defined as time to reduction in severity of each individual symptoms to at least one grade lower. For those who have call data only, the time that the symptoms reduced to at least one grade lower will be defined as the day the call was made. This will be calculated for each symptom and also for overall symptoms. Participants who withdraw or are lost to follow-up will be censored at their last contact date (definition 2.2.2). Participants who die will be censored at 28 days. Participants with symptoms rated as none at baseline will be censored at time 0.

3.3.2.3 Time to Sustained Recovery

Time to sustained recovery will be defined as the time to first reported recovery on the question ‘Do you feel recovered today?’ with subsequent responses of ‘yes’ to this question until day 28. For those who have call data only, the time to sustained recovery will be defined as the date at which they felt recovered or if this information is missing, the date the call was made. Subsequent calls must report feeling recovered. Participants who withdraw or are lost to follow-up will be censored at their last contact date (definition 2.2.2). Participants who die will be censored at 28 days. Where information from call data is not consistent with diary data, the diary data will take precedence. Where call data at days 7, 14 and 28 days report inconsistent dates of recovery, the date furthest from randomisation will be used as the date for sustained delivery (assuming recovery sustained).

In addition a binary variable shall be derived as sustained recovery or otherwise.

Sustained recovery will be derived as being recovered within the first 14 days and reports feeling recovered for the next 14 days. Where the participant has only call data, they require to have reported feeling recovered at both 14 days and 28 days. Where participants have got incomplete diary data for days 14-28 and no call data at 28 days, they are classified as otherwise.

Only participants who have reached day 28 follow-up will be included in the analysis of time to sustained recovery.

3.3.2.4 Time to Sustained symptoms alleviation

This will follow the same principle as time to initial reduction in severity of symptoms but there must be no subsequent symptom severity recorded as moderate or major. This will be calculated for individual symptoms separately and also for overall symptoms.

3.3.3 Contacts with health services between randomisation and day 28 of follow-up.

This will be split into participant reported health service use and health service use from GP records.

3.3.3.1 Participant reported health service use

Sources of data for this outcome are participant daily diaries and telephone call CRF (day 7, day 14 and day 28).

This will be presented as 2 outcomes:

1. A binary outcome indicating whether the participant has had any contact with health services during 28 days of follow-up. This will be 'yes' if any of the following are recorded as yes in the daily diary or call CRF:

    - GP (**GP_HOOCCUR**)
    - Other primary care services (**PCS_HOOCCUR**)
    - NHS 111 and other central advice resources (**NHS_HOOCCUR**)
    - A&E (**AE_HOOCCUR**)
    - Hospital (**HOSP_HOOCCUR**)
    - Other (**OTH_HOOCCUR, OTH_HOOCCUR_DEF**– free text)

2. A continuous variable of the number of health service contacts whilst alive during 28 days of FU.

    The number of health service contacts from the diary data will be the total number of times the participant has responded 'yes' to any of the following:

    GP_HOOCCUR, PCS_HOOCCUR, NHS_OOCCUR, AE_OOCUR, OTH_OOCCUR

    If the diary data is missing then the call CRF data will be utilised. The number of health service contacts from the call CRF will be the total of the following for each of the 7, 14 and 28 day calls:

GP_HOOCCURNUM, PCS_HOOCCURNUM, NHS_HOOCCURNUM, AE_HOOCCURNUM, OTH_HOOCCURNUM

3.3.3.2 Health Service Use From GP Records

Data for this outcome will come from the GP notes review after 28 days.

This will be presented as 2 outcomes:

1. A binary outcome indicating whether the participant has had any contact with health services during 28 days of FU. This will be 'yes' if any of the following are recorded as yes in the notes review:
    - GP (**GP_HOOCCUR**)
    - Other primary care services (**PCS_HOOCCUR**)
    - NHS 111 and other national resources (**NHS_HOOCCUR**)
    - A&E (**AE_HOOCCUR**)
    - Hospital (**HOSP_HOOCCUR**)
    - Other (**OTH_HOOCCUR, OTH_HOOCCUR_DEF**– free text)

2. A continuous variable of the number of health service contacts whilst alive during 28 days of FU. This will be derived by totalling the following for those with a notes review:

    GP_NUM, PCS_NUM, NHS_NUM, AE_NUM, OTH_NUM

3.3.4 Prescribing of antibiotics between randomisation and day 28 of FU

Notes review at 28 days will record information regarding prescription of antibiotics.

- Antibiotic prescribed (Yes = 1, No=1) (**ATBYN**)

This outcome will consider prescription of antibiotics whilst alive to account for truncation by death.

3.3.4.1 Derivation of outcome

Antibiotic prescribed = Yes, IF prescribed antibiotic [**ATBYN**=1] AND start date of antibiotic ≤28 days from data of randomisation. If case note review is available for a participant but no information is provided regarding antibiotics, it will be assumed that NO antibiotic was prescribed. If no notes review is available for a participant this will be recorded as missing with respect to antibiotic prescribing.

3.3.5 Hospital assessment without admission

This is captured on patients' daily diaries, Call CRF, notes review and the death and hospitalisation CRF. All sources will be considered and if any indicates a hospital assessment without admission it will be coded as such. If there are discrepancies between the sources of data the diary data will be assumed to be correct.

3.3.5.1 Derivation

Notes review, patient diary and call CRF:

Hospital assessment without admission = YES if on any day the participant reports going to hospital [**HOSP_HOOCUR** = Yes] AND on that date they were not admitted overnight [**HOSP_NIGHTYN** = No]

Death and hospitalisation CRF:

Hospital assessment without admission = YES if the patient has been admitted to hospital since they joined the trial (**HOSP_HOOCCUR**=1) and Date of admission (**HOSP_HOSTDAT**) = date of discharge (**HOSP_HOENDAT_DD**)

3.3.6 Oxygen administration

This is captured in patients' daily diaries, Call CRF, Notes review and death and hospitalisation CRF.

This outcome will reflect oxygen administration whilst alive to account for truncation due to death

3.3.6.1 Derivation

Oxygen administration = yes if patient reports receiving oxygen whilst in hospital [**OXY_HOOCCUR**=Yes] AND date of admission to hospital is ≤28 days from date of randomisation.

If oxygen use is reported via any data source then it will be counted as yes.

3.3.7 Intensive care unit admission

This is captured in patients' daily diaries, Call CRF, Notes review and the death and hospitalisation CRF. It will be calculated in 2 ways:

1. To reflect ICU admission whilst alive to account for truncation due to death prior to ICU admission. This definition of the outcome would give an estimate of the requirement for ICU between the randomised groups from a healthcare resource use perspective. This is derived as:

   Intensive care unit admission = yes if report of participant staying in ICU [ICUYN=Yes or ICU_HOOCCUR=Yes] AND date of admission to ICU is ≤28 days from date of randomisation. If intensive care unit admission is reported via any data source then it will be counted as an ICU admission.

2. To consider this outcome from a patient benefit perspective the outcome will be defined as a composite by assigning a "poor" outcome (i.e. ICU admission) to participants who die before requiring ICU admission. This is derived as:

   Intensive care unit admission = yes if report of participant staying in ICU [**ICUYN**=Yes or **ICU_HOOCCUR**=Yes] AND date of admission to ICU is ≤28 days from date of randomisation) OR participant has died.

If an ICU admission is recorded on any data source then it will be coded as an ICU admission.

3.3.8 Mechanical ventilation.

a. This outcome will reflect mechanical ventilation whilst alive to account for truncation due to death. This definition of the outcome would give an estimate of the requirement for ventilation between the randomised groups from a healthcare resource use perspective.
b. To consider this outcome from a patient benefit perspective the outcome will be defined as a composite by assigning a "poor" outcome to participants who die before requiring ventilation.

This is captured in patients' daily diaries, Call CRF, Notes review and death and hospitalisation CRF and captured from the question related to receiving mechanical ventilation.

3.3.8.1 Derivation

(a) Mechanical ventilation = yes if report of participant on mechanical ventilation [**MV_HOOCCUR**=Yes] AND date of admission to hospital is ≤28 days from date of randomisation.
(b) Mechanical ventilation = yes if participant dies OR (participant on mechanical ventilation AND date of admission to hospital is ≤28 days from date of randomisation [**MV_HOOCCUR**=Yes])

If mechanical ventilation is recorded on any data source then it will be coded as mechanical ventilation=YES.

3.3.9 Duration of hospital admission

This is captured in patient diaries, Call CRF, notes review and death and hospitalisation CRF.

The duration of hospital admission is calculated in the following ways from the different data sources:

Notes review: **HOSP_DUR**

Death and hospitalisation CRF: Difference between date of admission and date of discharge

Participant diaries: **HOSP_DUR**

Call CRF: **HOSP_NONIGHTS**

If a participant has been admitted with suspected COVID-19 more than once in the FU period of 28 days, the duration of hospital stay will be the sum of all admissions during FU, truncated at day 28.

All sources of data will be considered and if any indicates a hospital stay it will be coded as such. If there are discrepancies between the sources of data the diary data will be assumed to be correct. Only participants with a hospitalisation likely to be related to COVID-19 will be included in the analysis.

Participants admitted with likely COVID-19 and who die whilst in hospital will not be included in the estimate of mean duration and the number in each arm who die whilst in hospital will be reported in a footnote.

3.3.10 Negative effect on well-being (WHO-5)

Well-being is measured using the WHO well-being index which includes 5 items relating to well-being measured on a five point scale (scale of 5 =all of the time, 4=most of the time, 3=more than half the time, 2=less than half the time, 1=some of the time, 0=at no time). A total score is computed by summing the scores to the five individual questions to give a raw score ranging from 0 to 25 which is then multiplied by 4 to give the final score from 0 representing the worst imaginable well-being to 100 representing the best imaginable well-being. Negative effect on well-being is collected at baseline, 14 days and 28 days via the daily diary and call CRF.

From a patient benefit perspective participants who die before the measured time point will be given a score of 0 for that time point and included in the analysis.

3.3.11 WHO Ordinal Scale of Clinical Progression

There are various versions of this scale. We will use a score based on a number of factors including hospitalisation, use of oxygen, ventilation and death. It ranges from 1 (not hospitalised) to 6 (dead) and is defined as follows:

1 = Not hospitalised

2 = Hospitalised without need for supplemental oxygen

3 = Hospitalised with need for supplemental oxygen

4 = Hospitalised with need for non-invasive positive pressure ventilation or high-flow nasal cannula

5 = Hospitalised with need for mechanical ventilation or extracorporeal membrane oxygenation

6 = Death

This outcome will be derived at days 7, 14 and 28.

3.3.12 New infections in the household

This will be coded as ‘yes’ if the answer to the question “has anybody else in your house become unwell today with a respiratory illness?” is yes in the daily diary and/or the telephone call CRF at any time during the 28 days after randomisation.

3.3.13 Safety of treatments not licensed in the UK

For each treatment not licensed in the UK, the following AEs from the start of medication until the specified follow-up period, will be assessed by a clinician for causality and severity (See protocol for definitions): i) pre-defined AEs detailed in the intervention specific appendices (ISA) that are rated by the participant as 'moderate' and ii) other reported 'major' AEs. Severity will be rated by the clinician as Grade 1 (mild), grade 2 (moderate) and grade 3 (severe).Derivation of COVID-19 positive test result population

4 Analysis – General considerations

The following sections detail the final analysis for any treatment comparison within 28 days of randomisation. Methods for the primary analyses, including interim analyses, are specified in the Adaptive Design Report.

4.1 Participant throughput

The flow of participants through the trial will be reported following CONSORT and will include number of participants randomly assigned, receiving allocated treatment, followed up, withdrawn and analysed for primary outcome. Protocol deviations and information regarding screening information and number of ineligible participants randomised will be reported.

4.2 Descriptive statistics

Baseline characteristics of participants will be summarised both overall and by randomised group, including stratification factors and important prognostic, demographic and clinical characteristics. Binary and unordered categorical variables will be summarised using number, number missing and proportions. Continuous variables that are approximately normally distributed will be summarised using number, number missing, mean and standard deviation. Continuous variables that are not normally distributed or ordered categorical variables will be summarised using number, number missing, median and interquartile range.

There will be no tests of statistical significance nor confidence intervals for differences between randomised groups with respect to any baseline variable.

4.3 Characteristics of participants

Characteristics of participants to be described include age, sex (male/female/other), presence of comorbidities (Asthma COPD or other lung disease, Diabetes, Heart problems, High blood pressure, Liver disease, Stroke or other neurological problem), duration of symptoms prior to randomisation, symptoms (fever, cough shortness of breath, muscle ache and nausea/vomiting, other) rated as no problem, mild, moderate or major, medications, use of antibiotics , contact with health care services (GP, other primary care services, NHS 111, A&E, Hospital and other), test results for SARS-CoV-2 infection (not tested, missing, positive, negative), care home residency and ethnicity (collected at 28 days in addition to baseline in case these data were missed at baseline). Stratification factors will be described.

Tables of baseline characteristics will include only participants in the analysis population (as per section 4.6 i.e. excluded participants post randomisation will not be included). The number of randomised participants excluded from the analysis will be reported.

4.4 DESCRIPTION OF AVAILABLE DATA

The number of participants with available data for primary and secondary outcomes for the final analysis will be reported by treatment group.

Details describing methods for dealing with missing data with respect to the primary outcome will be described within the Adaptive Design Report (ADR).

4.5 COMPLIANCE WITH MEDICATION

Participants are asked in the online daily diaries to record whether they have taken their medication and if not, the reason why. The call CRF records the number of days they took the trial medication. For participants in randomised groups receiving medication, compliance with medication will be reported. The number of days that the allocated medication was taken will be reported along with withdrawals from treatment.

4.6 DEFINITION OF POPULATION FOR ANALYSIS

The analysis population will include all participants as defined by the protocol eligibility criteria.
As per ICH E9 guidance the following participants will be excluded from the analysis population;
(a) Participants randomised but subsequently found to be not eligible for randomization
(b) Participants previously randomised to an arm in the PRINCIPLE trial (subsequent randomisations will be excluded)
In addition, the following participants will be excluded;
(c) Participants who withdraw consent for data linkage and notes review and for whom no outcome data has been collected.

4.6.1 PRIMARY ANALYSIS POPULATION

The primary analysis population is defined as all randomised participants with a COVID-19 positive test for whom data were available with participants analysed according to the groups they were randomly allocated to, regardless of deviation from protocol.. In the setting of an adaptive platform trial with sequential overlap of treatment arms, the primary analysis for each intervention may be based on a distinct population. For each intervention that stops randomisation (due to futility) or becomes standard of care (due to superiority) based on interim analysis criteria, the date of the implementation of change in randomisation per interim decision will determine the intervention primary analysis population. For example, if randomisation is stopped for an intervention due to interim futility criteria, the final primary analysis includes all trial participants enrolled up to the date when randomisation was stopped to that intervention. If the randomisation is stopped for an intervention for other reasons (e.g. external reasons not related to the trial design), the primary analysis population will be specified in the intervention specific Appendix. Generally speaking, the primary analysis for a given intervention

will be based on complete 28-day follow-up of these participants, but the duration of follow-up may be impacted by public disclosure of interim results as determined by the Trial Management Group.

4.6.2 Concurrent Randomised and Eligible Analysis population

Analysis of the secondary outcomes for a given intervention will be based on participants who were randomised to the usual care arm during the same time frame when the intervention was actively randomised and who were eligible for randomisation to the intervention (i.e. Concurrent Randomised and Eligible Analysis Population).

4.6.3 Concurrent Randomisation Analysis Population

Sensitivity analyses of the primary analysis for a given intervention will be based on participants with a positive COVID-19 test result randomised during the same time frame when the intervention was actively being randomised, i.e. a concurrent randomisation analysis population.

4.6.4 Safety Analysis Population

Safety analysis will be conducted on the as treated population (i.e. the treatment that participants have received).

4.7 Comparative analysis

For all outcomes the primary analysis will be performed on the primary analysis population at 28 days after randomisation.

Each treatment arm will be compared with the usual care arm. If a treatment is deemed superior to usual care on both co-primary endpoints and replaces the usual care arm as the new standard of care, subsequent treatments will be compared with the original usual care arm.

There will be no formal adjustment for multiple comparisons.

4.8 Pooling of investigational sites

Data from all sites will be combined and analysed collectively. A sensitivity analysis of the primary outcomes may be carried out adjusting for geographical clinical research network (CRN) if deemed necessary.

4.9 Data Monitoring Committee And Interim Analyses

Details of the data monitoring committee and interim analyses can be found in the interim analysis report and the DMC charter.

5 PRIMARY ANALYSIS

5.1 Primary outcomes

Refer to ADR for details.

5.1.1 Summary of Bayesian Analyses to be conducted by Statistical Analysis Committee

Below is a summary of the pre-specified Bayesian analyses that may be requested from the Statistical Analysis Committee (SAC) for interventions that stop randomisation (due to futility) or become standard of care (due to superiority) based on interim analysis criteria:

B1. Bayesian interim analysis that satisfies the interim decision criteria

a. Details in ADR
b. Subgroup estimates from Bayesian model per SAP Section 8 (age<65 years, age ≥65 years without comorbidity, age ≥65 years with comorbidity)

B2. Bayesian primary analysis on “final data”

a. Details in ADR and M-SAP
b. Include subgroup estimates

B3. Sensitivity analysis: Repeat Bayesian primary analysis on concurrent randomisation analysis population

a. Details in ADR
b. Include subgroup estimates

B4. Secondary analysis: Repeat Bayesian primary analysis on overall population (i.e. regardless of COVID-19 test status). These models will be identical to the primary analysis models, but will include an additional baseline covariate and corresponding parameters for COVID-19 status (negative, positive, unknown).

a. Details in M-SAP
b. Includes subgroup estimates

The specific pre-specified Bayesian analyses required for each intervention will be specified in the Appendix.

6 SECONDARY ANALYSIS

6.1 Primary outcome on overall population

The primary outcomes will be analysed using the same method as detailed in the adaptive design report, but using the overall population with additional baseline covariate for COVID-19 test status (see B4 above).

6.2 Secondary outcomes

For all secondary outcomes, the analysis will compare each treatment arm with the usual care arm, in a pairwise comparison. For each analysis the Concurrent Randomisation and Eligible Analysis Population will be used. The analysis will be conducted on the Concurrent Randomisation and Eligible Analysis population with a COVID-19 positive test result and a secondary analysis of the secondary outcomes will be carried out on the overall Concurrent Randomisation and Eligible Analysis population with an additional covariate for COVID-19 test result status. Regression models (appropriate for each endpoint) will include randomised group (treatment/usual care) and stratification factors (age (continuous), comorbidity (Yes/no)). They will also include duration of illness at randomisation and vaccination status (as far as possible). Should it be necessary to compare more than one intervention with control at the same time, a covariate indicating which arms of the trial the participant was eligible to be randomised to will also be included. For binary outcomes with a low event rate, results will be reported descriptively by treatment group and a Chi-square test or Fisher's exact test may be used instead of the analysis detailed below. For continuous outcomes where the data are skewed, alternative non-parametric methods will be considered.

6.2.1 Patient reported illness severity

A linear mixed model will be used to analyse this outcome. The illness severity at each time point (7, 14, 21 and 28 days) will be included as the response variable, along with randomised group, age, presence of comorbidity, eligibility for treatment arm, duration of illness prior to randomisation vaccination status and time as fixed effects. Participant will be included as a random effect. Mean scores at each time point by randomised group will be described graphically.

6.2.2 Duration of severe symptoms

6.2.2.1 Time to alleviation of symptoms

Time to alleviation of symptoms will be compared between each treatment arm with the usual care arm using Cox proportional hazards model, adjusting for randomised group, age and presence of comorbidity at baseline. The model will also be adjusted for duration of illness (days) prior to randomisation and vaccination status. This will be calculated as the date of randomisation minus the start date of symptoms as reported on the screening CRF. If this date is unavailable then the date the patient registered with Principle will be used. The adjusted hazard ratio and 95% CI will be estimated from the model. A Kaplan Meier plot will also be presented. If the assumption of proportionality is not met, then an alternative survival model such as restricted mean survival method will be used.

Separate analyses will be carried out for overall and for each symptom separately.

6.2.2.2 Time to initial reduction of severity of symptoms

This will be analysed using the method described for time to alleviation of symptoms.

6.2.3 Time to Sustained recovery

This will be analysed using the method described for time to alleviation of symptoms.

6.2.4 Time to Sustained symptoms alleviation

This will be analysed using the method described for time to alleviation of symptoms.

6.2.5 Contacts with health care services

The number and percentage of participants with at least one contact with health services will be presented for treatment and usual care groups. This outcome will be analysed using a logistic regression model. Randomised group, age, presence of comorbidity, duration of illness and vaccination status will be included as covariates. The adjusted odds ratios will be reported for each pairwise comparison along with their associated 95% confidence interval and P-value. In addition, the adjusted absolute risk differences will be reported.

The number of contacts with health care services over 28 days will be analysed using a Poisson model. Randomised group, age, presence of comorbidity, duration of illness and vaccination status will be included as covariates. Adjusted incidence ratios will be presented with their 95% confidence intervals and related P value.

6.2.6 Prescribing of antibiotics

The count and percentage of participants with an antibiotic prescription will be presented for each treatment group. This outcome will be analysed using a logistic regression model. Randomised group, age, presence of comorbidity and duration of illness will be included as covariates. The adjusted odds ratios will be reported for each pairwise comparison along with their associated 95% confidence interval and P-value. In addition, the adjusted absolute risk differences will be reported.

6.2.7 Hospital assessment without admission

The count and percentage of participants with hospital assessment without admission will be presented for each treatment group. This outcome will be analysed using a logistic regression model. Randomised group, age, presence of comorbidity and duration of illness will be included as covariates. The adjusted odds ratios will be reported for each pairwise comparison along with their associated 95% confidence interval and P-value. In addition, the adjusted absolute risk differences will be reported.

6.2.8 Oxygen administration

The count and percentage of participants with oxygen administration will be presented for each treatment group. This outcome will be analysed using a logistic regression model. Randomised group, age, presence of comorbidity, duration of illness and vaccination status will be included as covariates. The adjusted odds ratios will be reported for each pairwise comparison along with their associated 95% confidence interval and P-value. In addition, the adjusted absolute risk differences will be reported.

6.2.9 Intensive care unit admission

The count and percentage of participants with intensive care unit administration will be presented for each treatment group. This outcome will be analysed using a logistic regression model. Randomised group, age, presence of comorbidity, duration of illness and vaccination status will be included as

covariates. The adjusted odds ratios will be reported for each pairwise comparison along with their associated 95% confidence interval and P-value. In addition, the adjusted absolute risk differences will be reported.

6.2.10 Mechanical ventilation

The count and percentage of participants with mechanical ventilation will be presented for each treatment group. This outcome will be analysed using a logistic regression model. Randomised group, age, presence of comorbidity, duration of illness and vaccination status will be included as covariates. The adjusted odds ratios will be reported for each pairwise comparison along with their associated 95% confidence interval and P-value. In addition, the adjusted absolute risk differences will be reported.

6.2.11 Duration of hospital admission

The mean duration of hospital admission will only be considered for those with a hospital admission and will be compared between each treatment arm with the usual care arm using a multiple linear regression model. The model will include outcome as the response variable, randomised group, age, presence of comorbidity, duration of illness and vaccination status as covariates. The mean (SD) duration will be presented in each group and the adjusted difference in means and 95% CI for each pairwise treatment arm comparison with the usual care group.

6.2.12 Well-being

The distribution of the WHO well-being index will be considered. Assuming there are not a large number of deaths or hospitalisations, a linear mixed effect model will be fitted to the data. Baseline well-being score will be fitted as a covariate in the model. Fixed effects will include randomised group, age, presence of comorbidity, duration of illness, vaccination status, time and a time x randomised group interaction. The mean (SD) well-being score at 14 and 28 days will be reported for each group and the adjusted difference in means (95% CI) for each pairwise treatment comparison with the usual care group will be presented.

6.2.13 WHO Ordinal Scale of Clinical Progression

If the data are available to calculate this scale then the following analysis will be carried out. The number and percentage of participants on each level of the scale will be presented by treatment group at days 7, 14 and 28. The outcome will be analysed using an ordinal logistic regression model, including the following covariates: randomised group; age; presence of comorbidity, duration of illness and vaccination status.

6.2.14 New infections in the Household

The count and percentage of participants with a new infection in the household will be presented for each treatment group. This outcome will be analysed using a logistic regression model. Randomised group, age, presence of comorbidity, duration of illness and vaccination status will be included as covariates. The adjusted odds ratios will be reported for each pairwise comparison along with their associated 95% confidence interval and P-value. In addition, the adjusted absolute risk differences will be reported.

6.2.15 Safety of treatments not licensed in the UK

For treatment arms that are not licensed in the UK, the number and percentage of participants reporting AEs will be reported by grade of severity.

6.3 Handling missing data

Participants who withdraw or are lost to FU will be included in the primary analysis and censored at last contact date. Participants with complete missing data (e.g. no diaries or calls) for a given endpoint will not contribute data to the respective primary analysis. The proportion of participants contributing no data to the respective primary analysis is expected to be low. Various imputation strategies will be considered when the proportion of participants contributing no data exceeds 15%.

6.4 Multiple comparisons and multiplicity

There will be no adjustment for multiplicity in the analysis of secondary outcomes.

6.5 Model assumptions

For the analysis of the secondary outcomes, model diagnostics will be checked.

7 Sensitivity analysis of co-primary outcomes using concurrent randomised analysis population

Upon conclusion of the study or intervention (and at interims as needed), a stand-alone sensitivity analysis of each co-primary analysis will be conducted for each completed intervention, in which each intervention is compared to Usual Care using the concurrent randomisation analysis population. These analyses will take the same form as the primary analysis models but may require modified priors/parameters for the temporal adjustment (carried out by the SAC and detailed in ADR and section 5.1 M-SAP). For some interventions (e.g. hydroxychloroquine), temporal adjustment may not be necessary for this sensitivity analysis.

8 Subgroup estimates

Model-based estimated treatment group differences in median time to recovery will be provided for each of the covariate subgroups (50-64 years old with comorbidities, ≥65 years old with comorbidities, and ≥65 years old without comorbidities), with 95% Bayesian credible intervals from the first co-primary analysis model. Similarly, model-based estimated differences in hospitalization rates will be provided for each of the covariate subgroups with 95% Bayesian credible intervals from the second co-primary analysis model (carried out by the SAC refer to section 5.1 M-SAP).

9 Moderation analysis

Moderation analyses of the time to recovery and death or hospitalisation outcomes will be carried out. Time to recovery will be analysed using the model specified for the analysis of time to alleviation of symptoms (section 6.2.2.1) and death or hospitalisation will be carried out using the same model used for other secondary binary outcomes (logistic regression model with randomised group, age and presence of comorbidity, duration of illness and vaccination status included as covariates). In addition the models will include an interaction between treatment group and the subgroup of interest. The P-values for the interaction effects will be presented and forest plots presented to show the effects in each subgroup and overall.

- Age group (<65/≥65 years)
- Presence of comorbidity at baseline (yes/no)
- Duration of symptoms prior to randomisation (this will be assessed as both a continuous outcome and using a cut off of ≤7 days vs. >7 days)
- Severity of symptoms score at baseline (using cut-off at ≤ 5 and > 5)

10 SAFETY ANALYSIS

10.1 Adverse events

Number and severity of serious adverse events (SAE) will be summarised across treatment arms using numbers and proportions.

11 VALIDATION

The analysis of the primary outcome will be validated by a second statistician from the SAC. The final analysis of the secondary and safety outcomes will be validated by a Senior Trial Statistician or suitably qualified delegate from Oxford PC-CTU.

12 CHANGES TO THE PROTOCOL OR PREVIOUS VERSIONS OF SAP

The current protocol (V7.1) has an outcome of ‘consumption of antibiotics’. Information regarding antibiotics has only been collected in the notes review and therefore relates to prescriptions of antibiotics rather than patient reported consumption.

Protocol version 7.1 states that Clinical data, and information from swab and blood tests, where available, will be used to classify participants according to aetiology. Blood test results have not been used to define the infected population for the intention to treat infected analyses.

13 Appendices

13.1 Appendix I. Schedule of procedures

Procedures	Participant contacts							
	Visit timing<br><br>Day 0	Day 0	Day 0	Day 0	Daily Day 1-28 incl	Day 28-12 months (monthly contact)	Day 29-12mths	Up to 10 years
	Screening completed by participant online/phone	Eligibility completed by participant online/phone	Baseline completed by participant online/phone	Eligibility completed by Clinician online/phone	Symptom Diaries completed by participant online/phone	Contacted by study team if consent provided	Retrospective data collection by study team	By data extraction from clinical records
Informed consent	X	X	X	X	X			
Demographics	X	X	X				X	
Medical history	X	X	X	X			X	

Swab<br><br>as part of the RCGP RSC/PHE national surveillance programme	When available, preferably by self-swabbing at study entry							
Concomitant medications		X					X	
Eligibility assessment	X	X						
Randomisation				X				
Dispensing of trial drugs				X	X			
Questionnaire					X	X		
WHO 5 Well Being Index	X				Day 14 and day 28	X		
Telephone interview (for subset of					X			

patient participants)								
Compliance					X			
Adverse event assessments					X*		X	
Optional SARS-CoV-2 blood test as part of the RCGP RSC/PHE national surveillance programme							X	
Evidence of sequalae and health care utilisation						X		X

13.2 Appendix II. Flow diagram of trial participants

Figure 1 Participant flow diagram

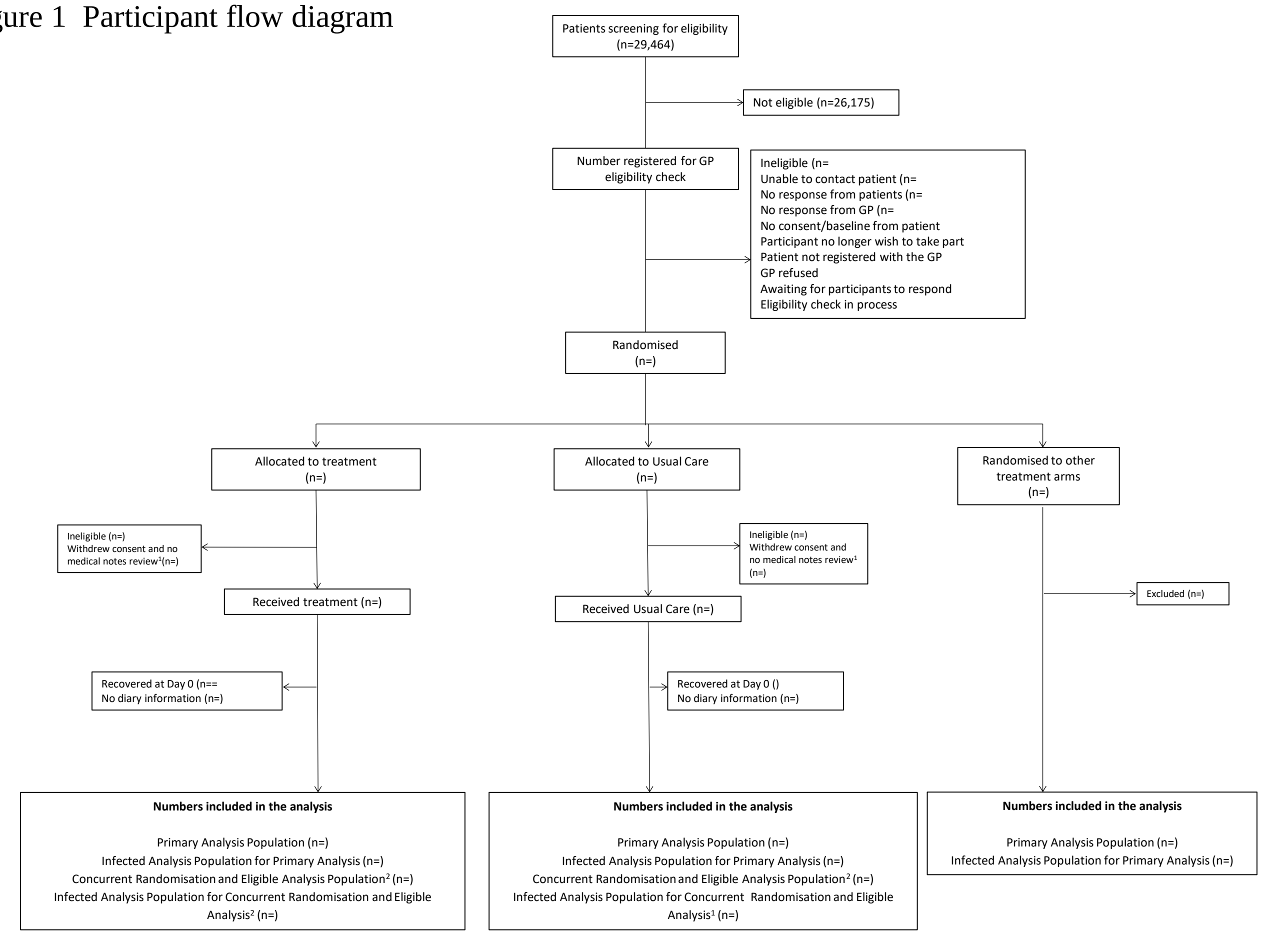

[1] Participants provided no diary information.
[2] Secondary outcomes only

13.3 APPENDIX III: Plans for analysis of Hydroxychloroquine

Population of Analysis

The primary analysis population for the co-primary outcomes is defined as all randomised participants for whom data were available with participants analysed according to the groups they were randomly allocated to, at the time of the first trial interim analysis regardless of deviation from protocol and irrespective of their COVID-19 status. Although, participants randomised to other treatment arms were included in this interim analysis, only results of the hydroxychloroquine intervention will be reported.

Sensitivity and secondary analyses will be based on all participants randomised up to the point when hydroxychloroquine stopped recruitment.

Descriptive statistics

Baseline characteristics of participants will be summarised both overall and by randomised group, for all participants up to the point when randomisation to hydroxychloroquine was stopped. Data will be summarised as described in the M-SAP.

Primary Outcome Analysis

Analysis of the co-primary outcomes, i.e. time-to-recovery from randomisation and hospitalisation or death at 28 days from randomisation, are detailed in the Adaptive Design Report. Because Hydroxychloroquine was stopped for external reasons not related to the trial design, the primary analysis for Hydroxychloroquine is based on all participants enrolled up to 30th November 2020 . This date coincides with the date that randomisation was stopped to Azithromycin per interim futility criteria, and is meant to optimise precision of the Hydroxychloroquine analysis. Data from participants allocated to the hydroxychloroquine arm, and all control participants' data available at the time of the first interim analysis will be used, so there will be more control participants in the primary analysis than participants allocated to the hydroxychloroquine arm contributing to the primary analyses. However, due to the general lack of availability of swab tests during the Hydroxychloroquine testing phase, secondary analyses on the SARS-CoV-2 positive population will not be performed.

**Analysis**	**Date last participant randomised to be included in the analysis**	**Duration Follow-up**	**Date of data transfer/lock**
**HCQ**	30th Nov 2020	28 days	11th Jan 2021

Specific sensitivity and secondary analysis of Primary Outcome

Bayesian analysis B2 and B3 (Section 5.1 M-SAP) will be requested.

Concurrent Randomisation analysis population

	**Date first participant randomised**	**Date last participant randomised**	**Duration Follow-up**	**Date of data transfer/lock**
HCQ	2nd April 2020	22nd May 2020	28 days	11th Jan 2021

SECONDARY OUTCOME ANALYSIS

Secondary outcomes will be analysed as described in the M-SAP. All secondary outcomes will be analysed as detailed in the M-SAP.

ANALYSIS OF SAFETY DATA

All serious adverse events will be reported as described in the M-SAP.

13.4 APPENDIX IV: PLANS FOR ANALYSIS OF AZITHROMYCIN

POPULATION OF ANALYSIS

The primary analysis population for the co-primary outcomes is defined as all randomised participants for whom data were available with participants analysed according to the groups they were randomly allocated to, at the time of the interim analysis regardless of deviation from protocol and irrespective of their COVID-19 status. Although, participants randomised to other treatment arms were included in this interim analysis, only results of the azithromycin intervention will be reported.

Sensitivity and secondary analyses will be based on all participants randomised between the first and last dates a patient was randomised to receive azithromycin. Only concurrent controls eligible for the azithromycin arm will be included.

DESCRIPTIVE STATISTICS

Baseline characteristics of participants will be summarised both overall and by randomised group, for all participants included in the primary analysis population. Data will be summarised as described in the M-SAP.

PRIMARY OUTCOME ANALYSIS

Analysis of the co-primary outcomes, i.e. time-to-recovery from randomisation and hospitalisation or death at 28 days from randomisation, are detailed in the Adaptive Design Report. All relevant data available at the time of the interim will be used in the primary analysis.

Analysis	Date last participant randomised to be included in the analysis	Duration Follow-up	Date of data transfer/lock
**Azithromycin**	30th Nov 2020	28 days	11th Jan 2021

SPECIFIC SENSITIVITY AND SECONDARY ANALYSIS OF PRIMARY OUTCOME

Bayesian analysis B1, B2, B3, B4 (Section 5.1 M-SAP) will be requested.

CONCURRENT RANDOMISATION ANALYSIS POPULATION

	Date first participant randomised	Date last participant randomised	Duration Follow-up	Date of data transfer/lock
**Azithromycin**	23rd May 2020	30th Nov 2020	28 days	11th Jan 2021

SECONDARY OUTCOME ANALYSIS

Secondary outcomes will be analysed as described in the M-SAP. All secondary outcomes will be analysed as detailed in the M-SAP.

ANALYSIS OF SAFETY DATA

All serious adverse events will be reported as described in the M-SAP.

13.5 APPENDIX V: PLANS FOR ANALYSIS OF DOXYCYCLINE

POPULATION OF ANALYSIS

The primary analysis population for the co-primary outcomes is defined as all randomised participants for whom data were available with participants analysed according to the groups they were randomly allocated to, at the time of the interim analysis regardless of deviation from protocol and irrespective of their COVID-19 status. Although, participants randomised to other treatment arms were included in this interim analysis, only results of the doxycycline intervention will be reported.

Sensitivity and secondary analyses will be based on all participants randomised between the first and last dates a patient was randomised to receive doxycycline. Only concurrent controls eligible for the doxycycline arm will be included.

DESCRIPTIVE STATISTICS

Baseline characteristics of participants will be summarised both overall and by randomised group, for all participants included in the primary analysis population. Data will be summarised as described in the M-SAP.

PRIMARY OUTCOME ANALYSIS

Analysis of the co-primary outcomes, i.e. time-to-recovery from randomisation and hospitalisation or death at 28 days from randomisation, are detailed in the Adaptive Design Report. All relevant data available at the time of the interim will be used in the primary analysis.

Analysis	Date last participant randomised to be included in the analysis	Duration Follow-up	Date of data transfer/lock
**Doxycycline**	14th Dec 2020	28 days	14th Jan 2021

SPECIFIC SENSITIVITY AND SECONDARY ANALYSIS OF PRIMARY OUTCOME

Bayesian analysis B1, B2, B3, B4 (Section 5.1 M-SAP) will be requested.

CONCURRENT RANDOMISATION ANALYSIS POPULATION

Analysis	Date first participant randomised	Date last participant randomised	Duration Follow-up	Date of data transfer/lock
**Doxycycline**	24th July 2020	14th Dec 2020	28 days	14th Jan 2021

SECONDARY OUTCOME ANALYSIS

Secondary outcomes will be analysed as described in the M-SAP. All secondary outcomes will be analysed as detailed in the M-SAP.

ANALYSIS OF SAFETY DATA

All serious adverse events will be reported as described in the M-SAP.

13.6 APPENDIX VI: PLANS FOR ANALYSIS OF INHALED CORTICOSTEROID BUDESONIDE

POPULATION OF ANALYSIS

The primary analysis population for the co-primary outcomes is defined as all randomised participants with a positive COVID-19 test result for whom data were available with participants analysed according to the groups they were randomly allocated to, at the time of the interim analysis regardless of deviation from protocol. Although, participants randomised to other treatment arms were included in this interim analysis, only results of the inhaled corticosteroid intervention will be reported.

Sensitivity and secondary analyses will be based on all participants randomised between the first and last dates a patient was randomised to receive inhaled corticosteroid. Only concurrent controls eligible for the inhaled corticosteroid arm will be included.

DESCRIPTIVE STATISTICS

Baseline characteristics of participants will be summarised both overall and by randomised group, for all participants included in the primary analysis population. Data will be summarised as described in the M-SAP.

PRIMARY OUTCOME ANALYSIS

Analysis of the co-primary outcomes, i.e. time-to-recovery from randomisation and hospitalisation or death at 28 days from randomisation, are detailed in the Adaptive Design Report. All relevant data available at the time of the interim will be used in the primary analysis.

SPECIFIC SENSITIVITY AND SECONDARY ANALYSIS OF PRIMARY OUTCOME

Bayesian analysis B1, B2, B3, B4(Section 5.1 M-SAP) will be requested.
Sensitivity analysis of co-primary outcomes using the concurrent randomised and eligible analysis population with a positive COVID-19 test result. Time to recovery will be analysed using Cox proportional hazards model, adjusting for randomised group, age, presence of comorbidity at baseline, duration of illness prior to randomisation and vaccination status. Death or hospitalisation will be carried out using a logistic regression model with randomised group, age and presence of comorbidity, duration of illness and vaccination status included as covariates. In addition, these models will be repeated on the concurrent randomised analysis population with a positive COVID-19 test result with additional adjustment for asthma/COPD at baseline as a surrogate for taking inhaled corticosteroid at baseline.

SECONDARY OUTCOME ANALYSIS

Secondary outcomes will be analysed as described in the M-SAP. All secondary outcomes will be analysed as detailed in the M-SAP.

ANALYSIS OF SAFETY DATA

All serious adverse events will be reported as described in the M-SAP.

13.7 Appendix VII: plans for analysis of colchicine

Population of Analysis

The primary analysis population for the co-primary outcomes is defined as all randomised participants with a positive COVID-19 test result for whom data were available with participants analysed according to the groups they were randomly allocated to, at the time of the interim analysis regardless of deviation from protocol. Although, participants randomised to other treatment arms were included in this interim analysis, only results of the colchicine intervention will be reported.

Sensitivity and secondary analyses will be based on all participants randomised between the first and last dates a patient was randomised to receive colchicine. Only concurrent controls eligible for the colchicine arm will be included.

Descriptive statistics

Baseline characteristics of participants will be summarised both overall and by randomised group, for all participants included in the primary analysis population. Data will be summarised as described in the M-SAP.

Primary Outcome Analysis

Analysis of the co-primary outcomes, i.e. time-to-recovery from randomisation and hospitalisation or death at 28 days from randomisation, are detailed in the Adaptive Design Report. All relevant data available at the time of the interim will be used in the primary analysis.

Specific sensitivity and secondary analysis of Primary Outcome

Bayesian analysis B1, B2, B3, B4 (Section 5.1 M-SAP) will be requested.

Secondary Outcome Analysis

Secondary outcomes will be analysed as described in the M-SAP. All secondary outcomes will be analysed as detailed in the M-SAP.

Analysis of Safety Data

All serious adverse events will be reported as described in the M-SAP.

13.8 APPENDIX VIII: PLANS FOR ANALYSIS OF FAVIPIRAVIR

Population of Analysis

The primary analysis population for the co-primary outcomes is defined as all randomised patients with a positive COVID-19 test result for whom data were available with participants analysed according to the groups they were randomly allocated to, at the time of the interim analysis regardless of deviation from protocol. Although, participants randomised to other treatment arms were included in this interim analysis, only results of the favipiravir intervention will be reported.

Sensitivity and secondary analyses will be based on all participants randomised between the first and last dates a patient was randomised to receive favipiravir. Only concurrent controls eligible for the favipiravir arm will be included.

Descriptive statistics

Baseline characteristics of participants will be summarised both overall and by randomised group, for all participants included in the primary analysis population. Data will be summarised as described in the M-SAP.

Primary Outcome Analysis

Analysis of the co-primary outcomes, i.e. time-to-recovery from randomisation and hospitalisation or death at 28 days from randomisation, are detailed in the Adaptive Design Report. All relevant data available at the time of the interim will be used in the primary analysis.

Specific sensitivity and secondary analysis of Primary Outcome

Bayesian analysis B1, B2, B3, B4 (Section 5.1 M-SAP) will be requested.

Secondary Outcome Analysis

Secondary outcomes will be analysed as described in the M-SAP. All secondary outcomes will be analysed as detailed in the M-SAP. AEs will be reported by grade of severity.

Analysis of Safety Data

All serious adverse events will be reported as described in the M-SAP.

Appendix 4

PRINCIPLE
Adaptive Design Report
Version 4.0

Ben Saville, Ph.D.
Nick Berry, Ph.D.

March 23, 2021

Contents

1 Trial Overview **3**
1.1 Version Control . . . 3

2 Co-primary Hypotheses **4**
2.1 Time to Recovery . . . 4
2.2 Hospital Admission or Death . . . 4

3 Co-primary Analyses **5**
3.1 Time to Recovery Analysis Model . . . 5
3.1.1 Adjustment for Temporal Changes . . . 6
3.2 Hospitalization/Death Analysis Model . . . 7
3.2.1 Adjustment for Temporal Changes . . . 7
3.3 Sensitivity Analyses . . . 8
3.4 Secondary Analyses . . . 8

4 Adaptive Design **9**
4.1 Interim Analyses for Superiority & Futility . . . 9
4.1.1 Interim Superiority . . . 9
4.1.2 Interim Futility . . . 10
4.1.3 Maximum Sample Size per Arm . . . 10
4.2 Additional Treatment Arms . . . 10
4.3 Allocation . . . 10
4.3.1 Response Adaptive Randomization . . . 11
4.3.2 Adding Interventions . . . 12
4.3.3 Comparing Interventions . . . 12
4.3.4 Standard of Care . . . 12
4.3.5 Combination Interventions . . . 12
4.3.6 Arm Suspension . . . 13
4.3.7 Eligibility Exclusions by Intervention . . . 13
4.4 Sample Size Justification . . . 13

5 Simulated Operating Characteristics **13**
5.1 Simulation Scenarios . . . 14
5.2 Operating Characteristics Base Scenarios (Three Interventions) . . . 14
5.3 OCs Additional Scenarios: Null Effect Hospitalization . . . 35
5.4 OCs Additional Scenarios: Modified Time to Recovery Rate for Usual Care Arm . . . 41
5.5 OCs Additional Scenarios: Fast Accrual . . . 51

6 Simulated Example Trials **58**
6.1 Simulated Example Trials: Three Interventions 58
6.2 Example 1 59
6.3 Example 2 67
6.4 Example 3 75
6.5 Example 4 89
6.6 Computational Details 101

1 Trial Overview

PRINCIPLE is an open, adaptive, platform trial to evaluate emerging treatments of persons with infection or suspected infection of the novel COVID-19 virus, with a focus on adults with existing comorbidities and/or older ages. The purpose of this document is to define the primary analysis and adaptive design of the PRINCIPLE trial. Complete details of all planned analyses are described in the Master Statistical Analysis Plan (M-SAP).

A "platform trial" is a trial in which multiple treatments for the same disease are tested simultaneously. The backbone of the trial is an adaptive clinical trial design. Pre-specified decision criteria allow for dropping a treatment for futility, declaring a treatment superior, or adding a new treatment to be tested. If at any point a treatment is deemed superior to Usual Care for both co-primary endpoints, the superior treatment will replace Usual Care as the new standard of care. Because the process of dropping and adding treatments may be on-going for an indefinite period of time, platform trials may be better conceived of as a process rather than a singular clinical trial. In the context of the COVID-19 pandemic, the trial may continue as long as the pandemic persists.

The PRINCIPLE trial will begin as a 1:1 randomized trial of Hydroxychloroquine versus Usual Care and will have the capability to add additional interventions over time. The evaluation of any new interventions will be governed by the master protocol, including adaptive and decision criteria. In addition, the inclusion of any new interventions will require supplementary appendices to the protocol and M-SAP.

1.1 Version Control

Multiple versions of this document (and trial design) have existed over time. Below we summarize key versions:

- Version 1.0, 09 May 2020: Initial draft design report, with primary endpoint of hospitalization
- Version 2.0, 07 July 2020: Second draft of design report, with primary endpoint changed to time to recovery
- Version 3.0, 09 September 2020: First version of completed design report, with co-primary endpoints of time to recovery and hospitalization
- Version 3.1, 22 September 2020: Minor changes to text; simulations and design unchanged
- Version 3.2, 23 October 2020: Changes to futility rules; update to accrual rates used in simulations (simulations incomplete)
- Version 3.3, 30 October 2020: Updated simulations to reflect modifications to design implemented in version 3.2
- Version 3.4, 06 November 2020: Minor changes to text (regarding interim communication channel); simulations and design unchanged
- Version 3.5, 08 March 2021: Changes to the hospitalization analysis (including temporal adjustment), model covariates (COVID swab result), and futility rules
- Version 4.0, 23 March 2021: Changed primary analysis population to those with COVID-19 positive swab test; clarified that adaptations (including RAR) are based on the primary analysis population (i.e. COVID-19 positive); changes to covariates used in both co-primary analyses (e.g. remove COVID swab); changed time intervals to 4 weeks (instead of 8 weeks) in the temporal adjustment of the hospitalization endpoint

2 Co-primary Hypotheses

The trial has two co-primary endpoints. The first co-primary endpoint is time to recovery from suspected COVID-19 infection within 28 days from randomization, where time to recovery is defined as the first instance that a participant reports feeling recovered. The second co-primary endpoint is hospital admission or death related to suspected COVID-19 infection within 28 days from randomization. The SAP defines three groups based on COVID-19 test status: 1) COVID-19 Positive; 2) COVID-19 Negative; and 3) No swab result available. The primary analysis population includes only individuals who are COVID-19 positive. A secondary analysis will replicate the primary analyses on the overall population.

Unless otherwise specified in the intervention-specific appendices, the co-primary outcomes will be analysed using a "gate-keeping" strategy. For a given treatment, time to recovery will be analysed first, and if the first null hypothesis is rejected, the second co-primary endpoint of hospitalization/death will be subsequently analysed. This gate-keeping strategy preserves the overall Type I error of the primary endpoints without additional adjustments for multiple hypotheses. In addition, the gate-keeping structure reflects the clinical belief that an intervention is unlikely to demonstrate benefit on the hospitalization/death endpoint without first demonstrating benefit on the time to recovery endpoint.

2.1 Time to Recovery

The first primary analysis is a Bayesian piecewise exponential of time to recovery regressed on intervention and baseline covariates (age, comorbidity, vaccination status) with temporal adjustment. For the purposes of modeling, age will be categorized as 1) at least 18 but less than 30 years old; 2) at least 30 but less than 50 years old; 3) at least 50 but less than 65 years old; or 4) at least 65 years old; comorbidity will indicate the presence or absence of comorbidities at baseline; and vaccination status will be categorized as "yes" vs. "other", where "yes" indicates at least one COVID-19 vaccination dose was given prior to randomization and "other" indicates either no vaccination was previously given or no vaccine information is available. Covariate categories may be collapsed for the purposes of modeling if insufficient sample size within a given category.

Let θ_j for $j > 0$ denote the log hazard ratio for time to recovery for persons on intervention j versus the Usual Care arm ($j = 0$), where $\theta_j > 0$ corresponds to faster recovery. Based on a Bayesian posterior distribution of θ_j, the primary analysis for intervention j will test the following hypothesis:

$$\begin{aligned} H_0 &: \theta_j \leq 0 \\ H_1 &: \theta_j > 0 \end{aligned} \tag{1}$$

If the Bayesian posterior probability of beneficial treatment effect (ψ_{1j} in equation 2) is greater than or equal to 0.99,

$$\psi_{1j} = \Pr(\theta_j > 0) \tag{2}$$

the null hypothesis will be rejected and the intervention will be deemed superior to Usual Care in the primary analysis population. The decision criteria controls the one-sided Type I error of each intervention at approximately 0.025.

2.2 Hospital Admission or Death

The second co-primary analysis is a Bayesian generalized linear model of the primary outcome regressed on treatment and baseline covariates (age, comorbidity, vaccination status) with temporal adjustment. For the purposes of modeling, covariate categories will be the same as the time to recovery analysis, where covariate categories may be collapsed if insufficient sample size within a

given category.

Let p_j denotes the probability of hospitalization/death for persons in treatment group j, where j=0 denotes the Usual Care arm and $j = 1$ denotes an intervention arm. A Bayesian posterior distribution will be derived for the estimated difference in probability of hospitalization/death between treatment groups. Let δ_j denote the log odds ratio of hospitalization/death comparing intervention j to Usual Care. The primary analysis for intervention j will test the following hypothesis:

$$\begin{aligned} H_0 &: \delta_j \geq 0 \\ H_1 &: \delta_j < 0 \end{aligned} \tag{3}$$

If the Bayesian posterior probability of beneficial treatment effect ψ_{2j} is greater than or equal to 0.975, with ψ_{2j} given in (4),

$$\psi_{2j} = \Pr(\delta_j < 0) \tag{4}$$

the null hypothesis will be rejected and the intervention will be deemed superior to Usual Care with respect to Hospitalization/Death in the primary analysis population. Note the decision criterion is lower than the recovery endpoint decision criterion (due to the gate-keeping structure), and controls the one-sided Type I error of each intervention at approximately 0.025 for plausible scenarios.

3 Co-primary Analyses

3.1 Time to Recovery Analysis Model

Let T_{ij} be the progression event time (in days) for time to recovery for the ith subject in arm j, where an event represents a positive outcome for the subject. We model the event times as piecewise exponential:

$$T_{ij} \sim PE(\lambda_{ij1}, \lambda_{ij2}, \lambda_{ij3}, \lambda_{ij4}), \tag{5}$$

where $\boldsymbol{\Lambda} = (\lambda_{ij1}, \lambda_{ij2}, \lambda_{ij3}, \lambda_{ij4})$ represents the set of hazard rates (events per day per subject) within each time segment s corresponding to subject i. The hazard rate for subject i on treatment j within time segment s is given by Equation (6),

$$\lambda_{ijs} = \exp(\gamma_s + \theta_j + \boldsymbol{x}_i' \boldsymbol{\beta} + \eta_{t(i)}), \tag{6}$$

where γ_s is the log hazard rate corresponding to time segment s, θ_j is the log hazard ratio for treatment relative to Usual Care, $\boldsymbol{x}_i$ is a vector of baseline covariates (age, comorbidity, vaccination status), $\boldsymbol{\beta}$ the corresponding vector of covariate parameters, and $\eta_{(i)}$ is a time drift parameter described in Section (3.1.1).

The log hazard rate corresponding to time segment s is given by equation (7),

$$\gamma_s = \alpha_1 + \alpha_2 I(s = 2) + \alpha_3 I(s = 3) + \alpha_4 I(s = 4) \tag{7}$$

where I() is an indicator function equal to 1 if the condition is satisfied and 0 otherwise, α_1 is the log hazard rate corresponding to time segment one (0-7 days), α_2 is the increment in log hazards for time segment two (8-14 days), α_3 is the increment in log hazards for time segment three (15-21 days), and α_4 is the increment in log hazards for time segment four (> 21 days). The prior distributions corresponding to the time segment parameters are given by equation (8),

$$\begin{aligned} \alpha_1 &\sim N(-2.3, 0.3^2) \\ \alpha_s &\sim N(0, 0.3^2) \text{ for } s > 1 \end{aligned} \tag{8}$$

where α_1 is centered at a hazard rate of 0.10, with increments in the log hazards centered at 0 for subsequent time segments, all with weakly informative variance.

For a time to recovery endpoint, treatment hazard ratios greater than one indicate treatment benefit. The log hazard ratio for treatment has the weak informative prior

$$\theta_j \sim N(0, 0.3^2), \tag{9}$$

and is assumed to be constant over time. The weak informative prior for the log hazard ratio places the prior mass of the HR between 0.5 and 2.0, which in line with clinical expectations for potential therapies, and also will be quickly overwhelmed with accruing data. A similar prior distribution is used for the covariate parameters given by equation (10),

$$\beta_k \sim N(0, 0.3^2) \tag{10}$$

where the covariate parameters are also assumed to be constant over time. Subjects with values of time to recovery equal to 0 days will be excluded from the analysis. Subjects with values for time to recovery greater than 28 days will be censored at 28 days. Subjects with incomplete diary data will be censored at the last date of entry. However, subjects who are censored due to death before first recovery will be censored at 28 days.

3.1.1 Adjustment for Temporal Changes

In the COVID-19 pandemic setting, there is a risk of temporal changes in the time to recovery, for example due to changes in virus prevalence, strain severity, or clinical care. Hence treatment arm comparisons based on non-concurrently randomized participants (e.g. due to staggered entry) can provide misleading results if temporal changes are not appropriately accounted for. In addition, the primary analysis for each intervention arm is based on the comparison of the intervention versus Usual Care (see exception for combination arms in Section 4.3.5). This is true even when an intervention replaces the Usual Care arm as the new standard of care. The rationale for comparing each arm to Usual Care, even when a superior arm has replaced Usual Care as the new standard of care, is because in a pandemic setting we are very interested in finding additional interventions that are superior to Usual Care (e.g. a “second best” intervention). This would be important if there are global supply shortages for the new standard of care. Hence adjustment for potential temporal changes is essential to the primary analysis. The temporal adjustment is made possible due to the overlapping enrollment across the respective treatment arms.

Bayesian methods are used to smooth the estimates across time intervals over the course of the trial. More specifically, we define 2-week time intervals from the start of the trial, and count backwards from the interval at the time of the data cut ($t = 1$) to the start of the trial, i.e. “walking backwards” in time. Let $\eta_{t(i)}$ denote the time offset parameter corresponding to the time of randomization for subject i. We set $\eta_1 = \eta_2 = 0$ corresponding to $t = 1$ and $t = 2$, i.e. for subjects randomized in the most recent two intervals. For every previous 2-week interval, the time parameter is modeled with the following Bayesian second order normal dynamic linear model (NDLM):

$$\eta_t \sim N(2\eta_{t-1} - \eta_{t-2}, \tau_\eta^2), \text{ for } t \geq 3 \tag{11}$$

The drift parameter τ_η^2 specifies the degree of smoothing over the time intervals, for which the following hyperprior distribution is used,

$$\tau_\eta^2 \sim \text{InvGamma}(a = 2, b = 0.0125) \tag{12}$$

and the inverse gamma (InvGamma) distribution is given by:

$$p(x) = \frac{b^a e^{-b/x}}{x^{a+1}\Gamma(a)} \tag{13}$$

This hyperprior distribution for τ_η^2 is equivalent to 4 intervals of data with τ_η centered at 0.079, i.e. a prior that time interval effects have small changes from one interval to the next. The individual time interval effects will be heavily shaped by the data from patients within the intervals. If there are insufficient numbers of patients within a given interval, time intervals will be collapsed as necessary to ensure stable model estimates.

Although the temporal adjustment in the primary analysis model is pre-specified, the unblinded statistical analysis committee may make adjustments to the temporal components as needed (e.g. change in prior distributions or length of intervals) to provide stable estimates of model parameters.

3.2 Hospitalization/Death Analysis Model

We define the second co-primary Bayesian analysis model for hospital admission or death related to suspected COVID-19. Among participants who have the opportunity to complete 28 days of follow-up, let Y_{ij} be the primary outcome measured at 28 days for subject i on arm j, with $j = 0$ denoting the Usual Care (control) and j denotes an intervention arm. We model the primary outcome as

$$\begin{aligned} \text{logit}(p_{ij}) &= \gamma_0 + \delta_j z_{ij} + \boldsymbol{x}'_{ij}\boldsymbol{\beta} + \eta_{t(i)} \\ Y_{ij} &\sim \text{Bernoulli}(p_{ij}) \end{aligned} \tag{14}$$

where p_{ij} is the probability of hospital admission/death for patient i on arm j; γ_0 is the log odds of hospitalization for the Usual Care arm (at reference levels of covariates); z_j is an indicator variable equal to 1 if subject i is randomized to intervention j (for $j > 0$) and 0 otherwise; δ_j is the log odds ratio of hospitalization/death comparing intervention j to Usual Care; $\boldsymbol{x}_{ij}$ is a vector of baseline covariates (age, comorbidity, vaccination status) specific to subject i and arm j; $\boldsymbol{\beta}$ is a vector of corresponding covariate log odds ratios; and $\eta_{(i)}$ is a time drift parameter described in Section (3.2.1). Note we use similar notation for some parameters from the first primary endpoint $(\boldsymbol{\beta}, \eta)$, but this is for notational convenience only, as these parameters do not represent the same population parameters across endpoints. The priors are given by:

$$\begin{aligned} \gamma_0 &\sim N(0, 2^2) \\ \delta_j &\sim N(0, 2^2) \text{ for } j \geq 1 \\ \beta_k &\sim N(0, 2^2) \text{ for } k = 1, 2, \ldots \end{aligned} \tag{15}$$

which are non-informative (disperse) prior distributions on the logit scale, and are expected to be overwhelmed with increasing number of events. If the number of events are insufficient to provide stable model estimates, the primary analysis model may be modified by removing or collapsing the baseline covariates in the model.

3.2.1 Adjustment for Temporal Changes

Similar to the time to recovery endpoint, Bayesian methods are used for the hospitalization endpoint to smooth the estimates across time intervals over the course of the trial. More specifically, we define 4-week time intervals from the start of the trial, and count backwards from the interval at the time of the data cut ($t = 1$) to the start of the trial, i.e. "walking backwards" in time. Let $\eta_{t(i)}$ denote the time offset parameter corresponding to the time of randomization for subject i. We set $\eta_1 = 0$ for subjects randomized in the most recent interval. For every previous 4-week interval, the time parameter is modeled with the following Bayesian second order normal dynamic linear model (NDLM):

$$\begin{aligned} \eta_2 &\sim N(0, \tau_\eta^2) \\ \eta_t &\sim N(2\eta_{t-1} - \eta_{t-2}, \tau_\eta^2), \text{ for } t \geq 3 \end{aligned} \tag{16}$$

The drift parameter τ_η^2 specifies the degree of smoothing over the time intervals, for which the following hyperprior distribution is used,

$$\tau_\eta^2 \sim \text{InvGamma}(a = 2, b = 0.1) \tag{17}$$

This hyperprior distribution for τ_η^2 is equivalent to 4 intervals of data with τ_η centered at 0.22, i.e. a prior that time interval effects have small changes from one interval to the next, after accounting for changes in baseline covariates. The individual time interval effects will be heavily shaped by the data from patients within the intervals.

Although the temporal adjustment in the primary analysis model is pre-specified, the unblinded statistical analysis committee may make adjustments to the temporal components as needed (e.g. change in prior distributions or length of intervals) to provide stable estimates of model parameters. For example, if there are insufficient numbers of hospitalizations within a given interval, time intervals may be collapsed as necessary to ensure stable model estimates. At the beginning of the trial, the proportion of COVID positive participants and hospitalization rates are both relatively low. Hence, the SAC may choose to combine the last two 4-week intervals (measured backwards from time of analysis) into a single 16-week time interval. Alternatively, the SAC may choose to maintain 4-week intervals, but modify the prior distributions for the last two intervals to induce higher correlation between the adjacent 4-week intervals. The goal is to provide a reasonable model of the temporal drift with respect to hospitalization/death rates.

3.3 Sensitivity Analyses

Upon conclusion of the study or intervention (and at interims as needed), the following sensitivity analyses are planned with respect to the primary outcome

- A sensitivity analysis will be conducted for each completed intervention, in which each intervention is compared to Usual Care using only concurrent randomizations in the primary analysis population. These analyses will take the same form as the primary analysis models but may require modified priors/parameters for the temporal adjustment. This analysis will include participants randomized to all regimens that are being concurrently randomized. For some interventions (e.g. hydroxychloroquine), temporal adjustment may not be necessary for the sensitivity analysis.
- Analyses evaluating the impact of missing data will be conducted as outlined in the M-SAP.

3.4 Secondary Analyses

Upon completion of each intervention (e.g. futility), the following secondary analyses may be conducted (details provided in M-SAP):

- The co-primary analyses will be replicated for the overall population, i.e. comparing time to recovery and hospitalization/death rates between treatment groups, among individuals regardless of test status. These models will be identical to the primary analysis models, but will include an additional baseline covariate and corresponding parameters for COVID-19 status (negative, positive, unknown).
- Other secondary analyses listed in the M-SAP

Each of the hypotheses for the overall population will be conducted at the same Bayesian posterior thresholds (0.99 for time to recovery and 0.975 for hospitalization) as the co-primary analyses using the gate-keeping structure. In other words, we first evaluate time to recovery in the overall population, and if significant we will evaluate the hospitalization/death endpoint in the overall population. Because other interventions may still be accruing information and using Usual Care subjects in their respective analyses, caution should be used in reporting and/or publishing results of an intervention, and will be limited to the analyses pre-specified in the M-SAP. Further guidance is provided in the PRINCIPLE Data Sharing/Access Policy.

4 Adaptive Design

The pre-specified design will allow adaptations to the trial based on the observed data. These adaptations include the declaration of superiority or futility of an arm at an interim analysis, the addition or removal of treatment arms, and changes in the randomization probabilities. All adaptations are based on the primary analysis population, and will occur at a given interim analysis if pre-specified conditions are satisfied. This adaptive design report was written while the trial was randomizing participants, but was finalized prior to conducting the first interim analysis.

4.1 Interim Analyses for Superiority & Futility

The timing of the first interim analysis will be determined by the trial management group (TMG) and statistical analysis committee (SAC), and will include logistical and operational considerations. Subsequent interim analyses will be conducted on a monthly basis provided the accrual rate is is approximately 25 or less participants per week. If accrual increases to approximately 50 participants per week, interims may be conducted every 2 weeks. If accrual increases above approximately 100 participants per week, interims may be conducted weekly. The TMG and SAC will monitor accrual rates and will have flexibility to adapt the frequency of the interim analysis to the observed accrual rate, with the goal of interim analyses occurring approximately every 100 completed observations. At each interim analysis, all enrolled intervention arms will be evaluated for superiority using the Bayesian interim primary analysis, provided that the intervention arm has at least 100 randomized participants with the opportunity to complete 28-days of follow-up for the primary endpoint. In addition, at each interim analysis all enrolled intervention arms will be evaluated for futility using the Bayesian interim primary analysis, provided that the intervention arm has at least 75 randomized participants with the opportunity to complete 28-days of follow-up for the primary endpoint.

4.1.1 Interim Superiority

If the Bayesian posterior probability of superiority of a given intervention over Usual Care is greater than or equal to 0.99 for the recovery endpoint, and greater than or equal to 0.975 for the hospitalization endpoint, superiority versus Usual Care will be declared on both endpoints, in which the superior arm will replace the Usual Care arm as the new standard of care. If a second intervention is found to be superior to the new standard of care on both endpoints, the second intervention will replace the existing standard of care. However, the primary analysis of each intervention arm (with exception for combination arms; see Section 4.3.5) will always be versus the Usual Care arm, even if participants are no longer being randomized to Usual Care.

If a decision of superiority is made for an intervention, additional enrollment and/or follow-up may continue on the randomized participants for that intervention, but any additional analyses or comparisons versus Usual Care will be considered secondary or sensitivity/exploratory analyses.

If superiority of an intervention is achieved for both primary endpoints, the DMSC will inform the Trial Steering Committee that superiority has been obtained on both endpoints, with details on size of treatment effect and probability of superiority obtained for each endpoint, and will recommend that the Trial Steering Committee (TSC) disclose the results to the Trial Management Group (TMG).

If the Bayesian posterior probability of superiority is achieved for the first co-primary endpoint (time to recovery) but not the second (hospitalization/death), the PRINCIPLE trial will continue randomizing to the Usual Care arm with allocation specified in Section (4.3.1). The DMSC will inform the TSC that superiority has been obtained on the first co-primary endpoint, with details on size of treatment effect and probability of superiority for both co-primary endpoints. The TSC will decide whether that information should be shared with the TMG based on guidelines detailed in the PRINCIPLE Data Sharing/Access Policy. In addition, interim results may be published by the TMG for a given intervention while the platform trial continues according to the PRINCIPLE Data Sharing/Access Policy.

4.1.2 Interim Futility

We define a futility rule based on the the Bayesian posterior probability of the hazard ratio from model (6). Let ω_{1j} be the model-based Bayesian posterior probability of a clinically meaningful treatment effect of the recovery endpoint, defined as the probability that the hazard ratio e_j^{θ} is at least 1.2 (equation 18). A hazard ratio of 1.2 corresponds to a 1.5 day benefit on median time to recovery, assuming an exponential distribution with median time to recovery of 9 days on the Usual Care Arm. If the probability of a meaningful effect on recovery, ω_{1j}, is less than or equal to 0.01,

$$\omega_{1j} = \Pr(e^{\theta_j} \geq 1.2) \tag{18}$$

intervention j will be dropped from the study for futility. If there are no other intervention arms available, the trial will be suspended; otherwise accrual continues to the remaining treatment arms.

If an intervention is superior for the first co-primary endpoint, a Bayesian posterior probability of a clinically meaningful treatment effect will be calculated for the second co-primary endpoint (hospitalization) using model (14). This probability, ω_{2j}, is defined as the probability that the odds ratio of hospitalization/death for intervention j relative to Usual Care is at least 1.18 (equation 19). An odds ratio of 1.18 corresponds to an absolute decrease in the probability of hospitalization/death of 0.02 (i.e. 2 percentage point decrease), assuming a hospitalization rate of 0.05 in the Usual Care arm. If ω_{2j} is less than or equal to 0.01,

$$\omega_{2j} = \Pr(e^{\delta_j} \geq 1.18) \tag{19}$$

the comparison versus Usual Care for hospitalization will be deemed futile with respect to the second co-primary analysis. However, randomization will continue to intervention j as described in Section (4.3.1).

All futility thresholds are non-binding, meaning that the SAC and DMSC may choose to override futility decisions if they mutually agree it is in the best interests of the trial. For example, suppose there is only a single active intervention and a futility threshold is met, despite there being some evidence of a small treatment benefit. This is possible given the aggressive futility rule that is meant to find treatments with larger benefit. Rather than suspend the trial due to lack of active interventions, it may be desirable to continue randomization to the remaining intervention despite the futility threshold being met, with the TSC being informed that they should consider adding additional interventions as soon as possible.

4.1.3 Maximum Sample Size per Arm

There is no cap on sample size per arm; hence interventions will remain active in the trial unless futility criteria are met or accrual is halted for budgetary or other considerations.

4.2 Additional Treatment Arms

At any point in the trial (per recommendations of the Trial Steering Committee), the trial management group may elect to add an additional intervention to the trial. The evaluation of any new interventions will be governed by the master protocol, including adaptive and decision criteria. In addition, the inclusion of any new interventions will require supplementary appendices to the protocol and M-SAP. This includes updated simulations describing the revised operating characteristics of the trial with the additional arm.

4.3 Allocation

At the start of the trial, randomization was fixed 1:1 to hydroxychloroquine (HCQ) versus Usual Care, with stratification by age (less than 65, greater than or equal to 65), and comorbidity (yes/no). Randomization was suspended to HCQ on May 23, 2020 per the Medicine and Health Care Products

Berry Consultants
Statistical Innovation

Regulatory Agency (MHRA), at which time the trial began randomizing 1:1 to Azithromycin versus Usual Care. Protocol Amendment 6 included the addition of the intervention Doxycycline, which is began enrolling around the end of July 2020. This adaptive design report was finalized prior to conducting the first interim analysis, which is expected to accomplish the following tasks:

1. Evaluate primary hypotheses for Hydroxychloroquine vs. Usual Care
   - Due to the sudden halt in accrual to hydroxychloroquine, there is only a single analysis evaluating hydroxychloroquine benefit versus Usual Care. Hence the posterior probability thresholds for hydroxychloroquine versus Usual Care equal 0.975 for BOTH of the co-primary endpoints. In addition, the analysis of hydroxychloroquine will include all subjects in the Usual Care arm up until the time of the interim analysis.
2. Evaluate early superiority/futility for all active interventions with a sufficient number of participants with the opportunity to complete 28 days of follow-up (per Section 4.1)
3. Determine subsequent randomization probabilities of active interventions

Subsequent interim analyses will evaluate superiority/futility of all active interventions with a sufficient number of participants with the opportunity to complete 28 days of follow-up (per Section 4.1), as well as determine subsequent randomization probabilities for all active interventions. An active intervention is defined as an intervention currently randomizing participants. Note Hydroxychloroquine will remain an inactive treatment regardless of the first interim results.

When there is more than one active intervention in the trial, the allocation probability to Usual Care will be set to $1/Z$, where Z is the total number of active arms in the trial (e.g., $Z = 3$ for Usual Care and two active interventions).

4.3.1 Response Adaptive Randomization

Prior to the first interim analysis, randomization will be set to $1/Z$ for each of the intervention arms. Response adaptive randomization (RAR) will be activated at the time of the first interim analysis if there at least two active interventions in the trial. When RAR is activated, the Usual Care arm will continue to receive a fixed allocation of $1/Z$. The remaining $(Z-1)/Z$ allocation probability will be divided among the intervention arms based on interim RAR probabilities. The purpose of RAR is to allocate more participants to the intervention arms with the best observed outcomes (relative to Usual Care).

For example, if there are 3 active arms (2 active intervention arms and Usual Care), the Usual Care allocation will be fixed at 1/3, and the remaining 2/3 allocation will be split among the two intervention arms via response adaptive randomization. The RAR probabilities (q_j) for the intervention arms will be proportional to the Bayesian posterior probability that a given intervention is superior to Usual Care (ψ_{1j}) with respect to the first co-primary endpoint (time to recovery) in the primary analysis population; see equation (2). The calculation of ψ_{1j} is based on the MCMC posterior samples from the Bayesian interim primary analysis. The randomization probabilities q_j of the intervention arms at a given interim are normalized so that they sum to $(Z-1)/Z$.

If superiority is achieved for a given intervention for both co-primary endpoints, the superior intervention will replace the Usual Care arm and will receive a fixed $1/Z$ allocation thereafter if if there are no other interventions that have achieved superiority on either endpoint. If superiority is achieved for a given intervention, say intervention X, with respect to the first co-primary endpoint (time to recovery) but not the second co-primary endpoint (hospitalization/death), intervention X will be considered part of the "standard of care" (SOC) along with Usual Care. In this setting, intervention X and Usual Care will be both continue randomization, in which they make up a single standard of care arm receiving a total allocation probability of $1/Z^*$. The value Z^* replaces the value Z, and is equal to 1 plus the number of active interventions that have not achieved superiority

on the recovery endpoint, such that the composite SOC is considered a single arm with respect to allocation. The allocation within the composite SOC will be shared equally among the arms with SOC designation.

For example, suppose there are 3 active interventions versus Usual Care, and intervention X is superior to Usual Care for the recovery endpoint but not the hospitalization endpoint. The standard of care now refers to both intervention X and Usual Care, and the number of arms in the trial Z^* equals 3, referring to a composite SOC (composed of intervention X and Usual Care) and 2 active interventions. The two SOC arms will receive a total of $1/(2Z^*) = 1/6$ allocation each, and the remaining 2/3 allocation is assigned to the other 2 active interventions via RAR.

4.3.2 Adding Interventions

When a new intervention is added, the RAR allocation for the new arm will be bounded between $1/(2Z)$ and 1/2 for the first 4 weeks, after which this condition is removed. The remaining allocation will be given to the standard of care (fixed $1/Z$) and the other interventions, proportional to probability of superiority on time to recovery versus Usual Care (ψ_{1j}).

4.3.3 Comparing Interventions

Although the primary analysis compares each intervention versus Usual Care, the primary analysis model also enables pairwise comparisons between interventions. These pairwise comparisons will be conducted at each interim analysis. If an intervention is superior to another intervention per criterion of Bayesian posterior probability of superiority ≥ 0.99 for recovery and ≥ 0.975 for hospitalization, the inferior intervention will be dropped from the trial, regardless of how the intervention compares to Usual Care. Upon completion of enrollment for two interventions, the same criteria will be evaluated, i.e. a Bayesian posterior probability ≥ 0.99 and ≥ 0.975 for recovery and hospitalization, respectively, for one intervention versus another intervention will indicate pairwise superiority for each co-primary endpoint, with an identical gate-keeping sequential order of hypotheses for a given pairwise comparison.

4.3.4 Standard of Care

Standard of care (SOC) will initially refer to the Usual Care arm. However, if the Usual Care arm is replaced at any point by a superior intervention (due to superiority on both co-primary endpoints), standard of care would then refer to the superior intervention. If an intervention is superior to Usual Care on the first co-primary endpoint (time to recovery) but not the second (hospitalization/death), both the Usual Care arm and intervention arm will be considered standard of care arms.

4.3.5 Combination Interventions

A combination intervention is defined as a combination of one of the existing interventions plus another treatment. The primary analysis for the combination arm will be as specified in Section (3), except when the stand-alone component of the combination has been determined to be superior to Usual Care for a given primary endpoint. In such settings, the primary analysis of the combination arm will be versus the standalone component arm. For example, if azithromycin is superior to Usual Care on the first co-primary endpoint (recovery) only, and a new combination enters the trial as a combination of azithromycin plus intervention X, the primary analysis of the combination will be to evaluate superiority versus azithromycin for the first co-primary endpoint, and superiority versus Usual Care for the second co-primary endpoint. If azithromycin is superior to Usual Care on both co-primary endpoints, the primary analysis of the combination will evaluate superiority versus azithromycin for both co-primary endpoints. Note the combination arm will use the same gate-keeping strategy for evaluating the two co-primary endpoints regardless of the comparison group.

4.3.6 Arm Suspension

If the randomization probability $q_j < 0.05$ for any intervention j at a given interim, the intervention j is suspended from the trial, which is done by setting the randomization probability q_j equal to 0 until the next interim analysis. If the next interim analysis no longer meets this threshold for a previously suspended dose, randomization to the intervention may then resume according to the algorithm. If at any point in the trial there only exist two active arms, randomization will be fixed at 1:1.

4.3.7 Eligibility Exclusions by Intervention

A participant is eligible for randomization provided the participant meets the eligibility criteria for at least two arms, one of which includes a standard of care arm. If such a participant is ineligible for one of the interventions, the randomization probabilities will be re-normalized (i.e. probabilities sum to 1.0) among the remaining arms prior to randomization. Eligibility status will be recorded for each participant as (eligible, ineligible, or unknown) for each of the treatment arms. If the number of participants who are known to be eligible for one active intervention but ineligible for another active intervention becomes substantial, the primary analysis model may be modified to to include eligibility status as a covariate.

4.4 Sample Size Justification

Given the open perpetual trial structure, the trial does not have a finite ending based on sample size. Rather, the trial will continue until either superiority or futility is claimed for an intervention, or until the pandemic expires in the population. We estimate that approximately 400 participants per arm (800 participants total if only a single intervention vs. usual care) will be required to provide 90% power for detecting a difference of 2 days in median recovery time in the primary analysis population. This calculation is based on the assumption of an exponential distribution for time to recovery with a median of 9 days in the Usual Care group, with some adjustments for missing data and multiple interim analyses. Alternative assumptions are explored in the virtual trial simulations

On average, we expect fewer participants to be required when there is a large treatment benefit or complete lack of benefit. For example, if the true benefit is a 3 day benefit in median time to recovery (6 days intervention vs. 9 days Usual Care), on average only 155 subjects per arm are required to provide sufficient power. The primary advantage of the adaptive design is the ability to adapt the sample size to the observed data, thus addressing the primary hypothesis as quickly and as efficiently as possible.

In addition, we estimate that approximately 1500 participants per arm (3000 participants total if only a single intervention vs. usual care) will be required to provide 90% power for detecting a 50% reduction in the relative risk of hospitalization/death in the primary analysis population. This calculation is based on the assumption of an underlying 5% combined hospitalization/death rate in the Usual Care arm, with an intervention lowering the hospitalization/death rate to 2.5%, with some adjustments for the multiple interim analyses. We expect fewer participants to be required to detect a 50% reduction if the event rate in the Usual Care arm is greater than 5%.

For a more accurate sample size justification (via simulation) of the adaptive platform trial design, we refer to Section 5.

5 Simulated Operating Characteristics

Because of the adaptive platform trial structure, there exists no simple formula(s) to calculate power and Type I error (false positive rate). Hence, virtual trial simulations are used to fully characterize and quantify the power and Type I error of the design. The simulations include a comprehensive evaluation of trial performance across a wide range of assumptions (e.g. underlying distribution

of outcome in control arm, treatment effect, accrual rates, etc.). This includes summaries regarding the number of subjects required to make a superiority or futility conclusions for each intervention.

Simulations below were conducted in October 2020 based on the available knowledge and trial design at the time, which is detailed in the Adaptive Design Report version 3.3 (30 October 2020). Although some modifications have been made to the trial design (e.g. futility rules, primary analysis population of COVID-19 positive participants) to address a dynamic pandemic situation, the simulations can be viewed as an approximation to the performance of the adaptive design for evaluating future interventions in the primary analysis population.

5.1 Simulation Scenarios

The time to recovery is simulated using an exponential distribution with rate parameter λ. Based on available knowledge, we expect $\lambda_0 = \log(2)/9$ for the Usual Care arm (corresponding to a median of 9 days), but also consider lower and higher values of λ corresponding to a median of 7 and 11 days. We explore different scenarios for the hazard ratio reduction proportion hospitalized relative to control; these are given by HR=(1, 1.10, 1.20, 1.30). We also explore different scenarios for the treatment effects on hospitalization rates, where the aggregate hospitalization rate is assumed to be 0.025. This is done by imposing treatment effect sizes in absolute percentage decrease relative to the Usual Care arm equal to the null (0), small (1%), medium (2%), or large (3%) treatment effects while enforcing that the average hospitalization rate for the simulated arms (including Usual Care) equals 0.025.

At the time of running these simulations, the trial had been enrolling for approximately 6 months. Hence, our simulations include the observed accrual over the first 6 months (20-70 participants per week), followed by expected accrual thereafter based on a Poisson process. Given the uncertainty in accrual due to the state of the COVID pandemic, we consider maximum accrual rates of 30 (“expected”) and 75 (“fast”) subjects per week.

Given the large number of potential scenarios based on the factors listed above, simulations are conducted on a base set of scenarios with a median time to recovery of 9 days, hazard ratios of 1.0, 1.1, 1.2, and 1.3 for time to recovery, and hospitalization treatment effects of null (0%), small (1%), medium(2%), or large(3%). We then explore additional scenarios for a small subset of the base scenarios, where we 1) cross the null hospitalization scenarios with various treatment effects for the recovery endpoint; 2) cross different SOC median time to recovery rates with various treatment effects for the recovery endpoint; and 3) simulate faster accrual of 75 participants per week with bi-weekly interim analyses.

5.2 Operating Characteristics Base Scenarios (Three Interventions)

This section explores the simulated trial operating characteristics (OCs) for the first three interventions (Hydroxychloroquine, azithromycin, doxycycline) versus Usual Care using a base set of scenarios. Given simulated virtual trials, the adaptive algorithm is applied at each interim update to conduct a virtual trial exactly as described. Various summaries are captured, including the average sample size, average trial duration (in weeks), the probability of stopping accrual for early futility or superiority, and the total probability of success, where success is defined as claiming superiority versus Usual Care. Note “power” is calculated as the proportion of simulated trials that claim superiority on the primary endpoint for scenarios with a treatment benefit for a given endpoint, and Type I error is calculated as the proportion of simulated trials that claim superiority on the primary endpoint for scenarios with no treatment benefit for a given endpoint. This is done for all combinations of accrual, treatment effect, and the assumed median time to recovery in the Usual Care arm.

Principle OCs

Berry Consultants

10/26/2020

These operating characteristics are based on 1000 simulations per scenario across a variety of assumed treatment effects for experimental arms in the trial. The true number of patients randomized per day from April 1, 2020 to October 19, 2020. After this period is over, the simulations assume a 50 patient per week accrual rate. In absence of a definitive rule, these simulations conduct an interim every 28 days. In addition, the true day of enrollment stopping of HCQ is used as well as the days for opening enrollment to the Azithromycin and Doxycycline arms. As such, up to at least October 19, 2020 (day 199) of the trial, the enrollment rates to each arm in these simulations are expected to align closely with the true enrollment rates to the arms in the trial.

Median TTR	Hazard Ratio			Power for TTR				Hospitalization Rate Benefit (%)			Power for TTR & Hosp		
SOC	HCQ	Azith	Doxy	SOC	HCQ	Azith	Doxy	HCQ	Azith	Doxy	HCQ	Azith	Doxy
9	1.0	1.0	1.0	0.952	0.017	0.031	0.022	0	0	0	0.000	0.001	0.000
9	1.0	1.0	1.1	0.623	0.016	0.022	0.370	0	0	1	0.000	0.000	0.065
9	1.0	1.0	1.2	0.117	0.014	0.018	0.883	0	0	2	0.000	0.000	0.496
9	1.0	1.0	1.3	0.011	0.013	0.012	0.989	0	0	3	0.000	0.000	0.952
9	1.0	1.1	1.0	0.592	0.014	0.401	0.030	0	1	0	0.000	0.079	0.000
9	1.0	1.1	1.2	0.108	0.013	0.383	0.878	0	1	2	0.000	0.070	0.459
9	1.0	1.1	1.3	0.013	0.014	0.238	0.986	0	1	3	0.000	0.032	0.883
9	1.0	1.2	1.0	0.096	0.011	0.904	0.016	0	2	0	0.000	0.510	0.000
9	1.0	1.2	1.1	0.085	0.012	0.900	0.326	0	2	1	0.000	0.487	0.052
9	1.0	1.2	1.2	0.037	0.015	0.879	0.865	0	2	2	0.000	0.422	0.409
9	1.0	1.2	1.3	0.007	0.012	0.798	0.986	0	2	3	0.000	0.342	0.816
9	1.0	1.3	1.0	0.013	0.015	0.987	0.012	0	3	0	0.000	0.923	0.002
9	1.0	1.3	1.1	0.015	0.015	0.985	0.183	0	3	1	0.000	0.867	0.022
9	1.0	1.3	1.2	0.003	0.015	0.987	0.741	0	3	2	0.000	0.820	0.325
9	1.0	1.3	1.3	0.000	0.015	0.978	0.972	0	3	3	0.000	0.755	0.760
9	1.1	1.1	1.1	0.451	0.180	0.412	0.357	1	1	1	0.012	0.078	0.068
9	1.2	1.2	1.2	0.042	0.540	0.883	0.848	2	2	2	0.117	0.378	0.349
9	1.3	1.3	1.3	0.003	0.839	0.970	0.947	3	3	3	0.406	0.636	0.633

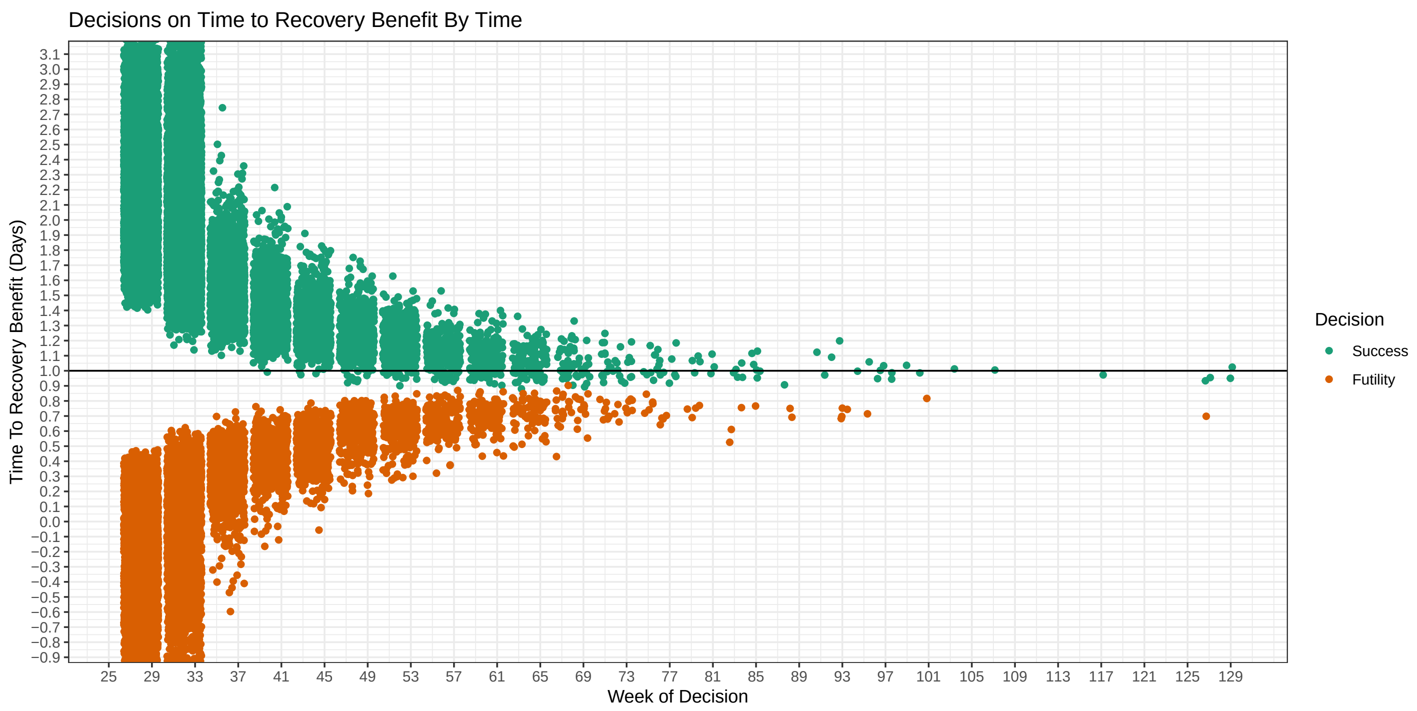

Decisions on Time to Recovery Benefit By Time
Time To Recovery Benefit (Days)
3.1
3.0
2.9
2.8
2.7
2.6
2.5
2.4
2.3
2.2
2.1
2.0
1.9
1.8
1.7
1.6
1.5
1.4
1.3
1.2
1.1
1.0
0.9
0.8
0.7
0.6
0.5
0.4
0.3
0.2
0.1
0.0
−0.1
−0.2
−0.3
−0.4
−0.5
−0.6
−0.7
−0.8
−0.9
25 29 33 37 41 45 49 53 57 61 65 69 73 77 81 85 89 93 97 101 105 109 113 117 121 125 129
Week of Decision
Decision
Success
Futility

Hazard Ratios: HCQ: 1 Azith: 1 Doxy: 1

Decisions Across Time

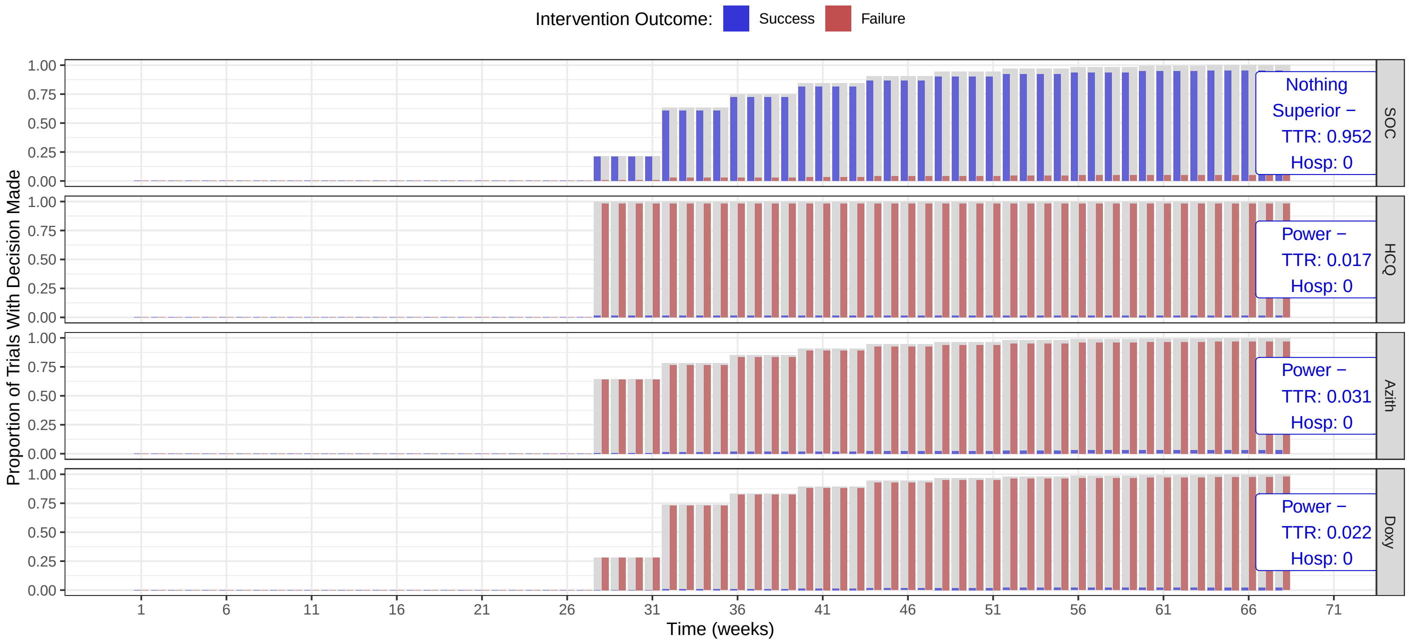

Randomization Rates

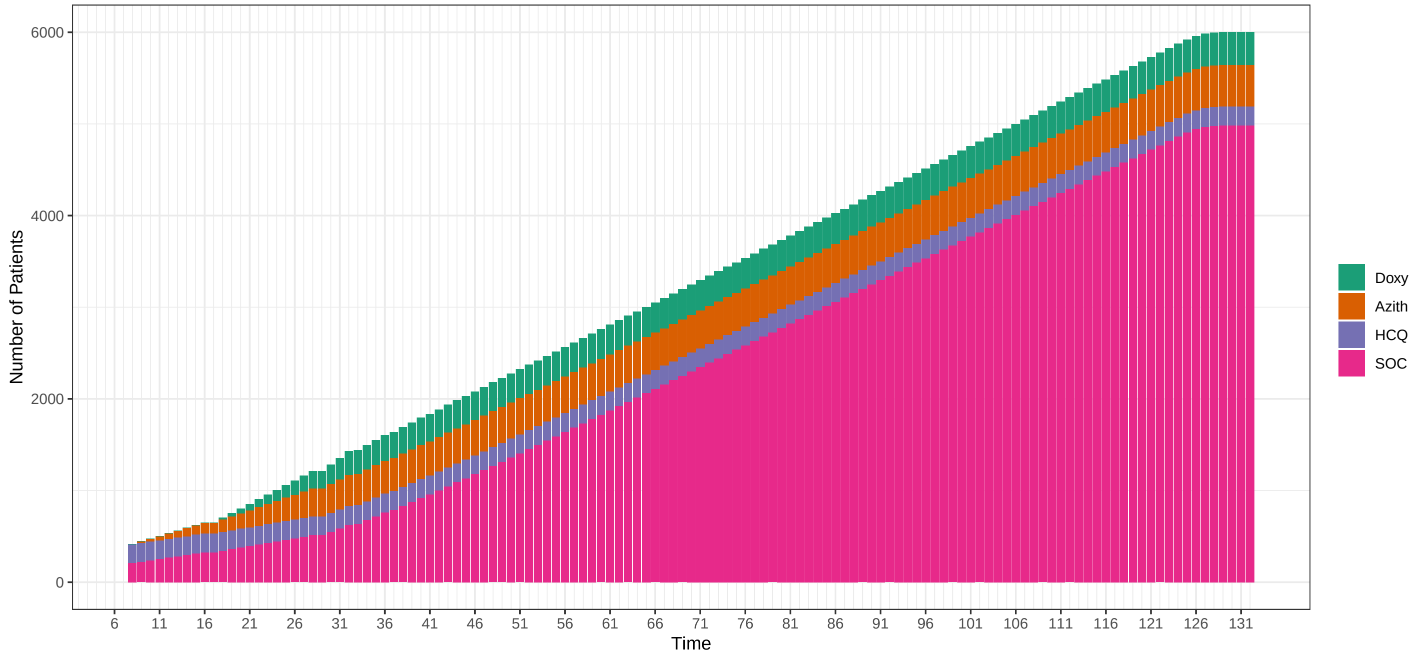

Hazard Ratios: HCQ: 1 Azith: 1 Doxy: 1.1

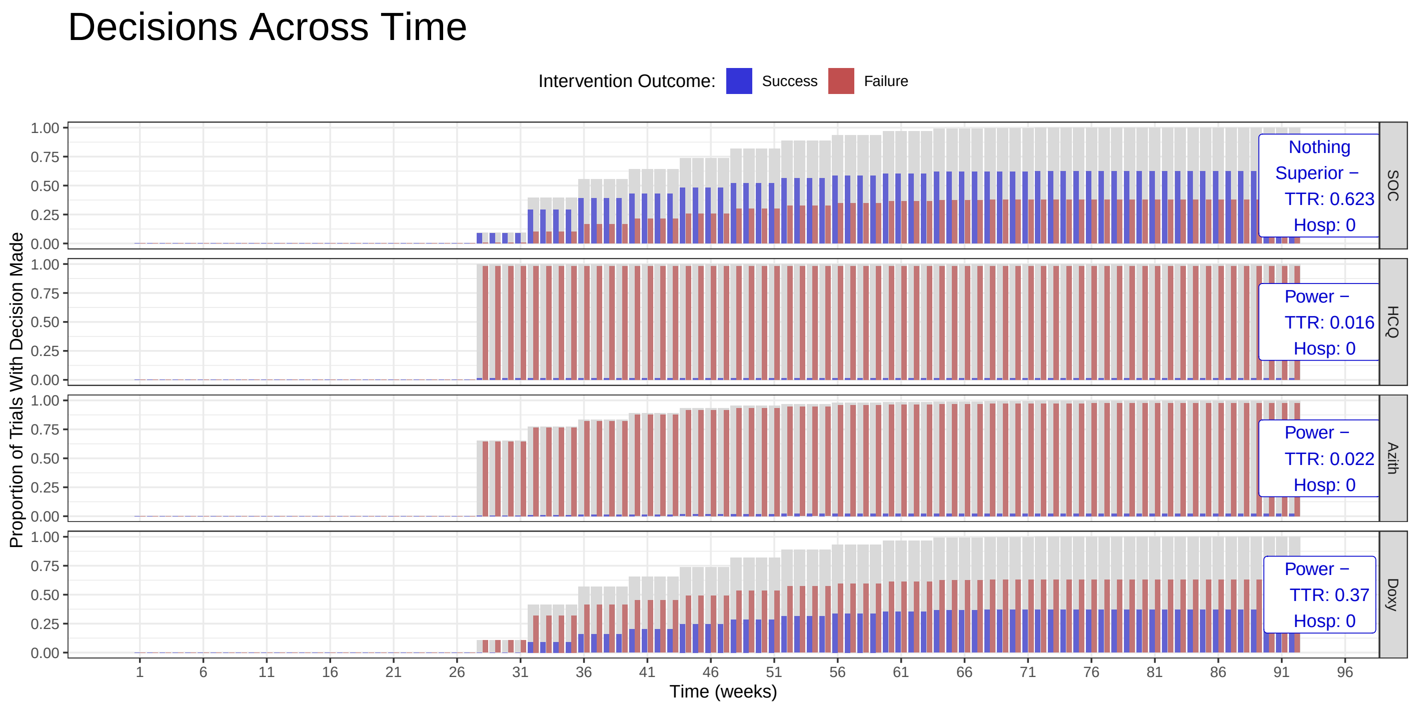

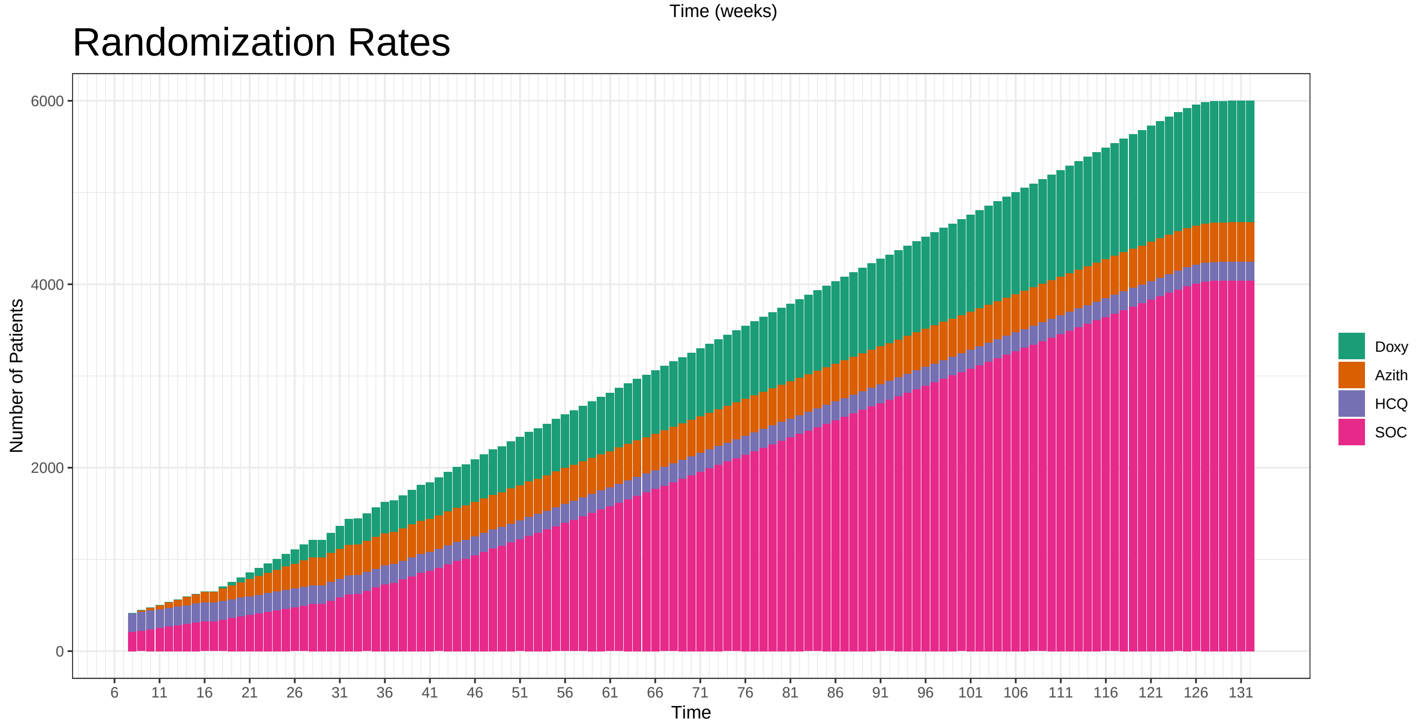

Hazard Ratios: HCQ: 1 Azith: 1 Doxy: 1.2

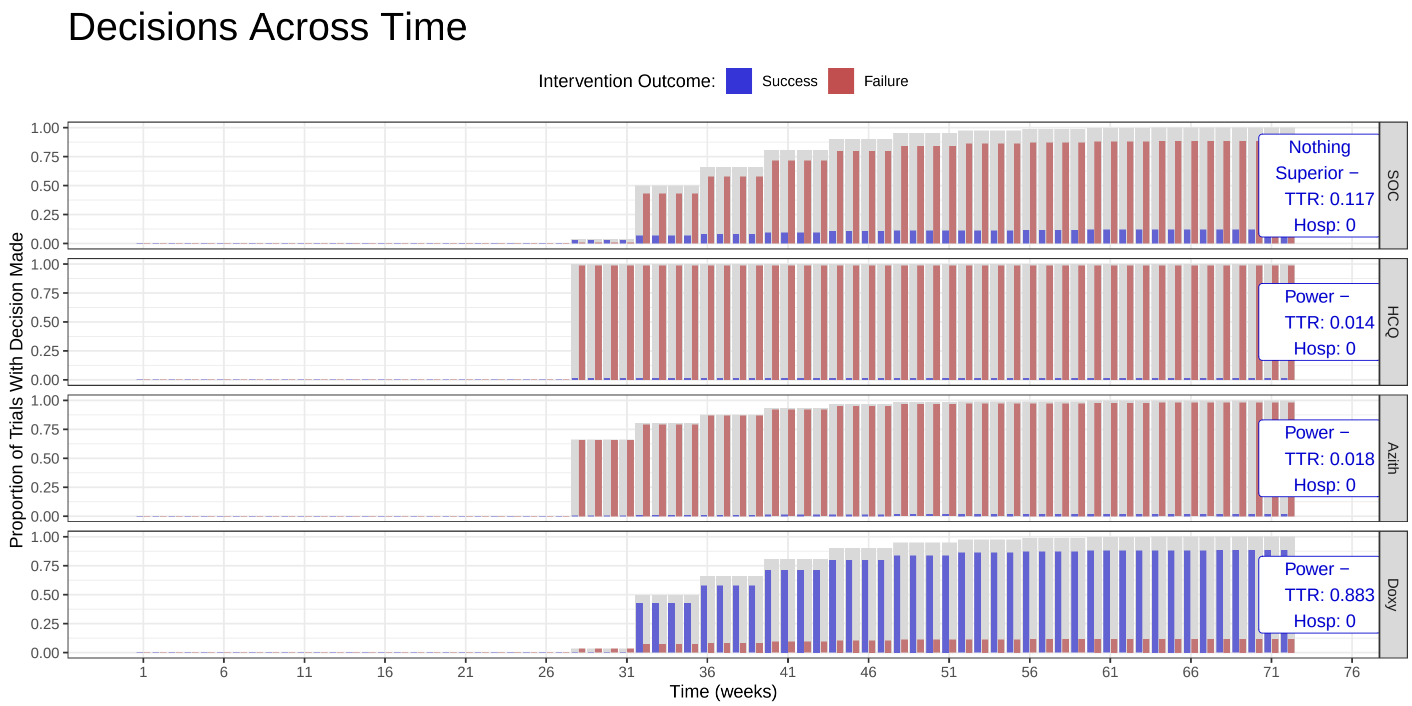

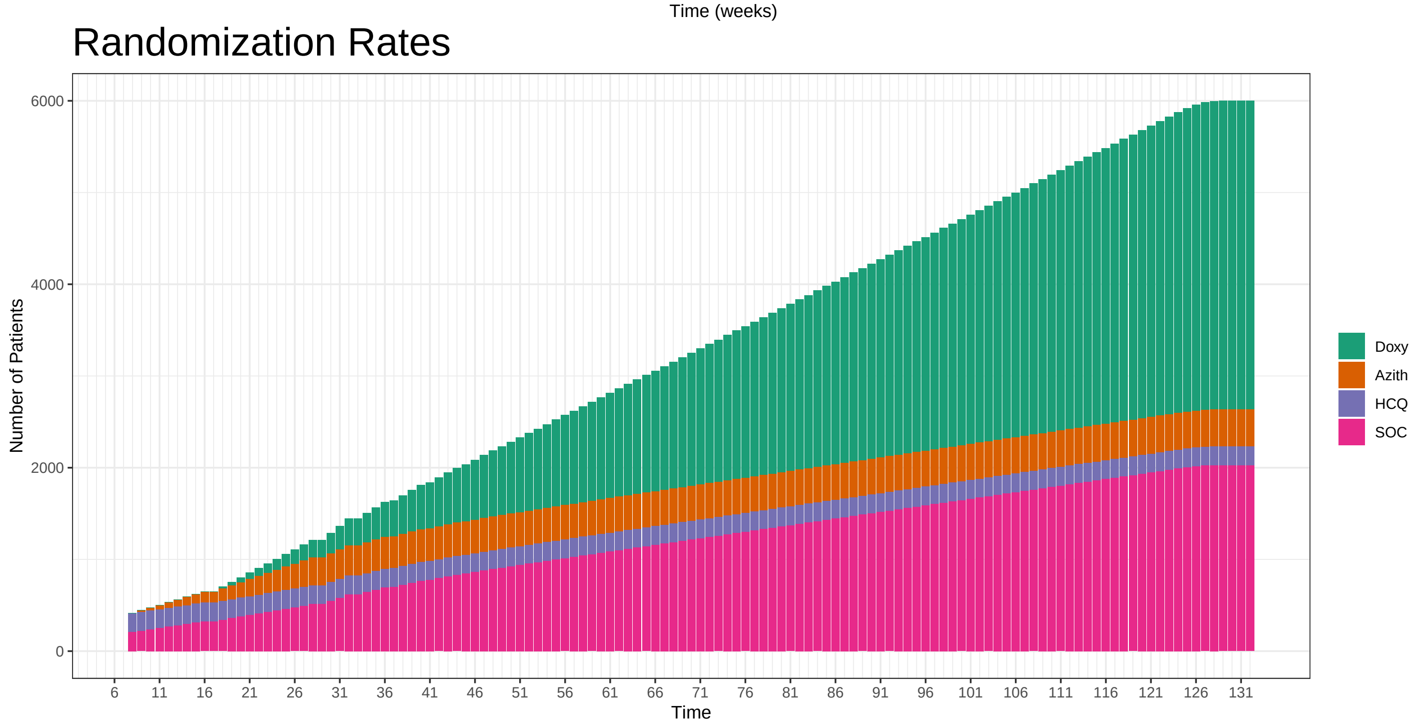

Hazard Ratios: HCQ: 1 Azith: 1 Doxy: 1.3

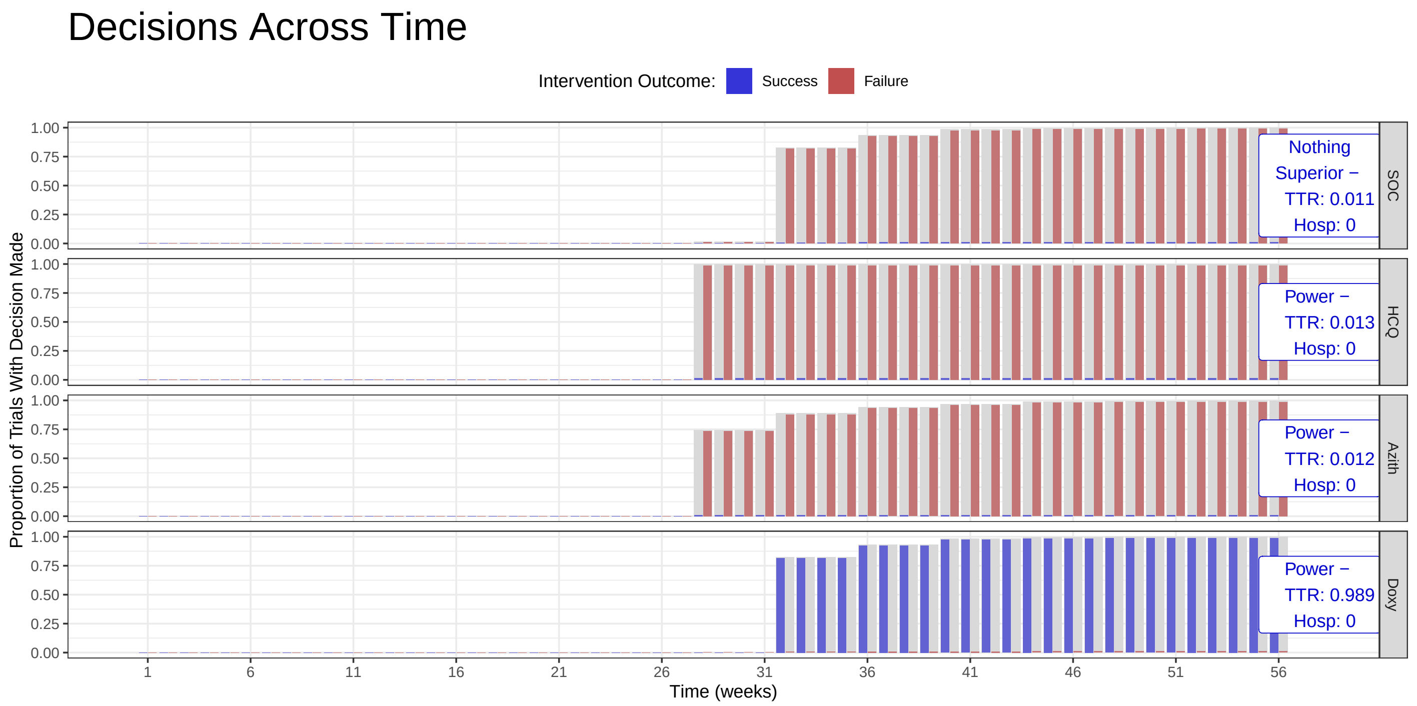

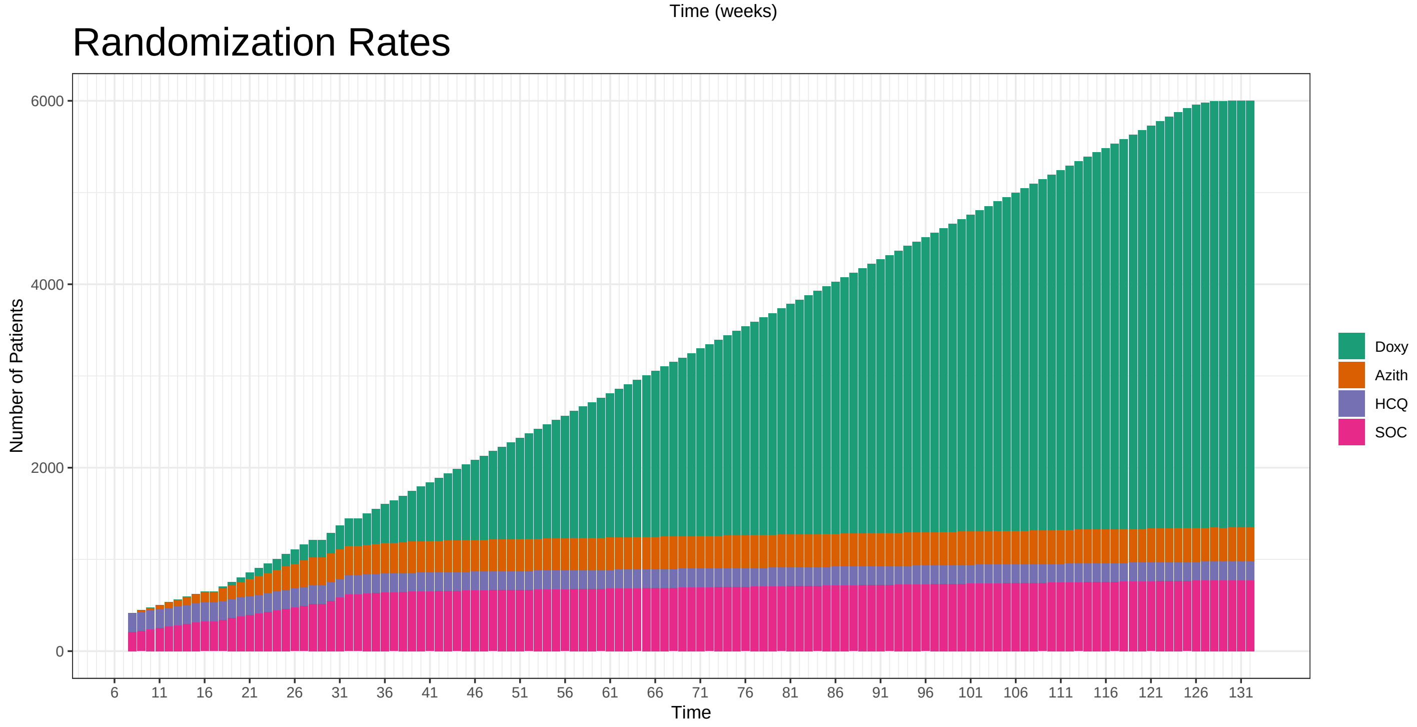

Hazard Ratios: HCQ: 1 Azith: 1.1 Doxy: 1

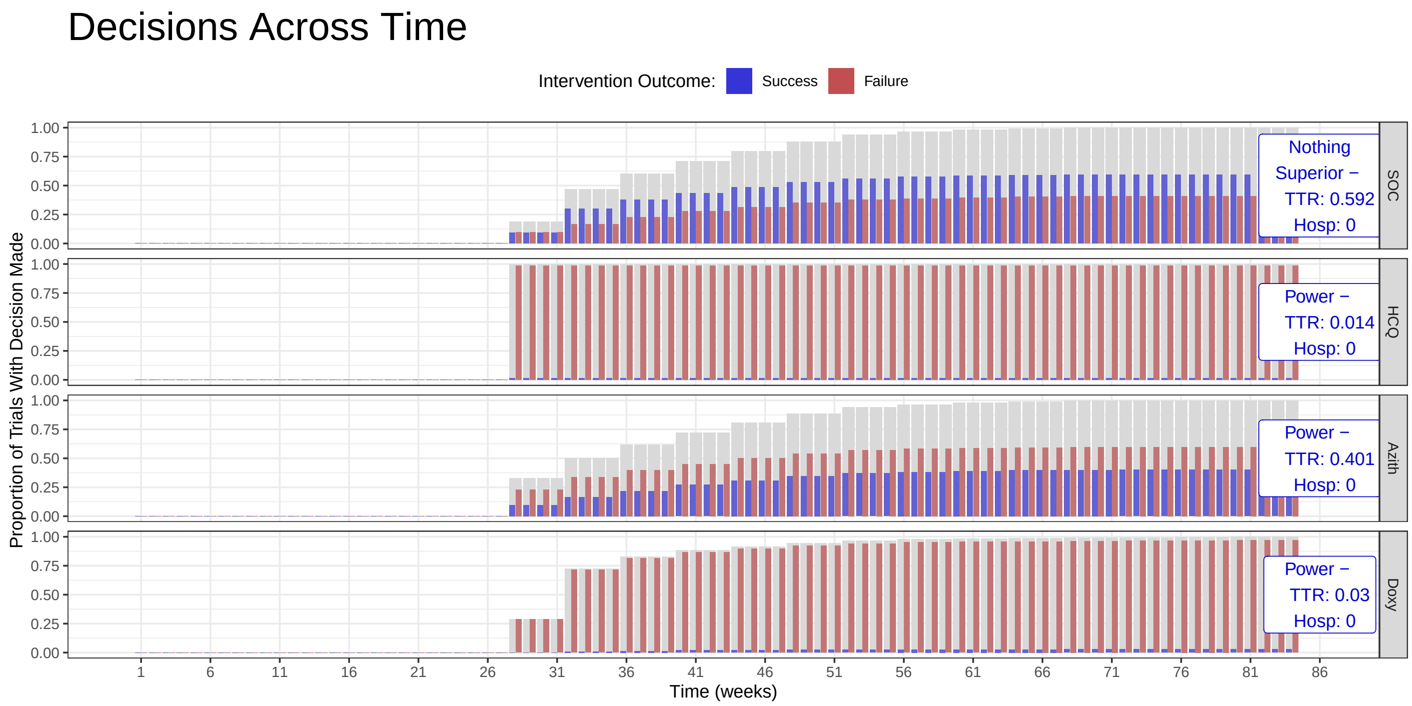

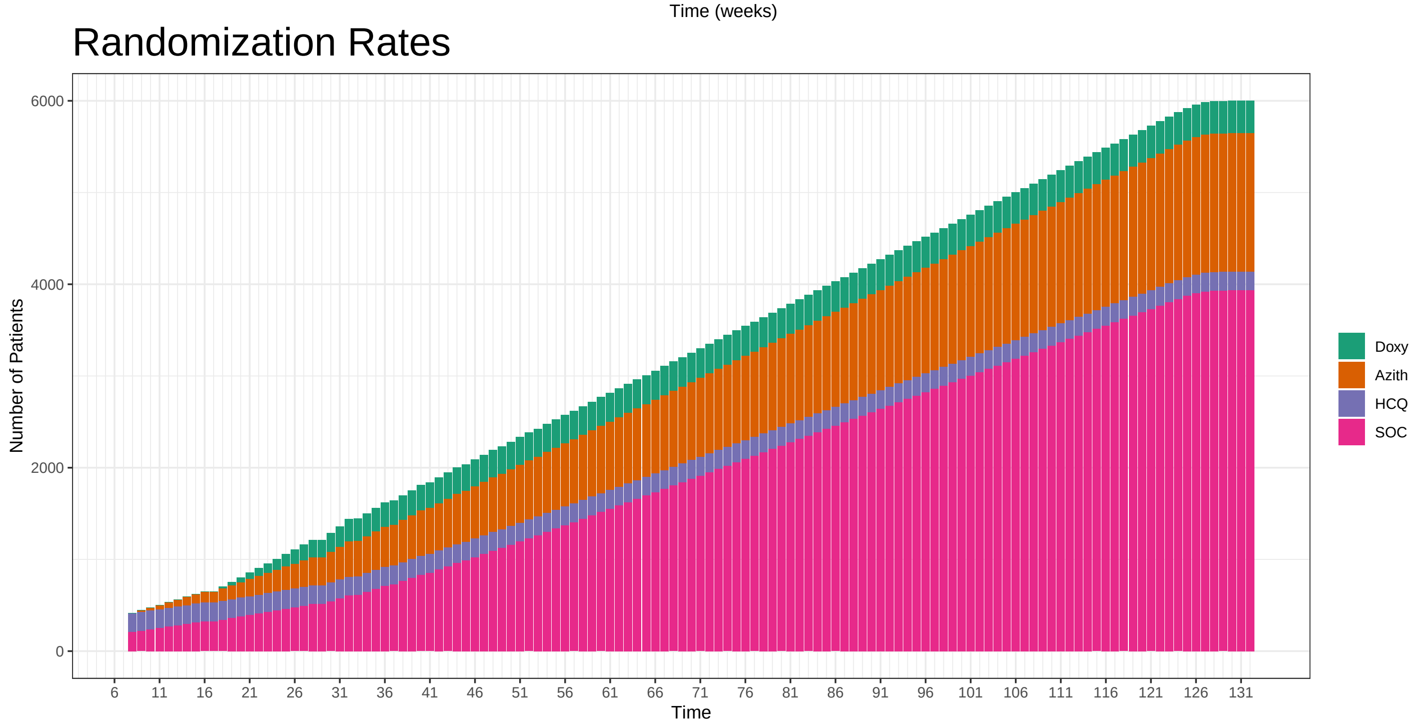

Hazard Ratios: HCQ: 1 Azith: 1.1 Doxy: 1.2

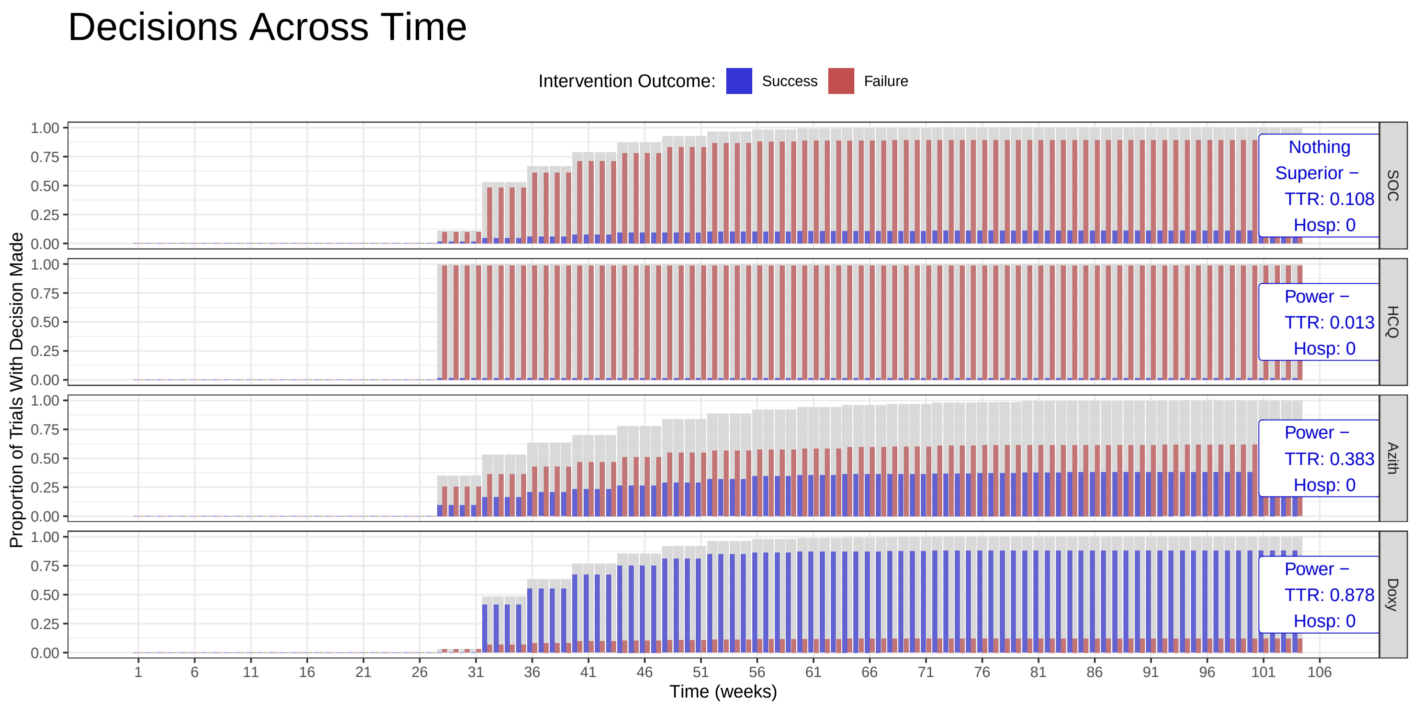

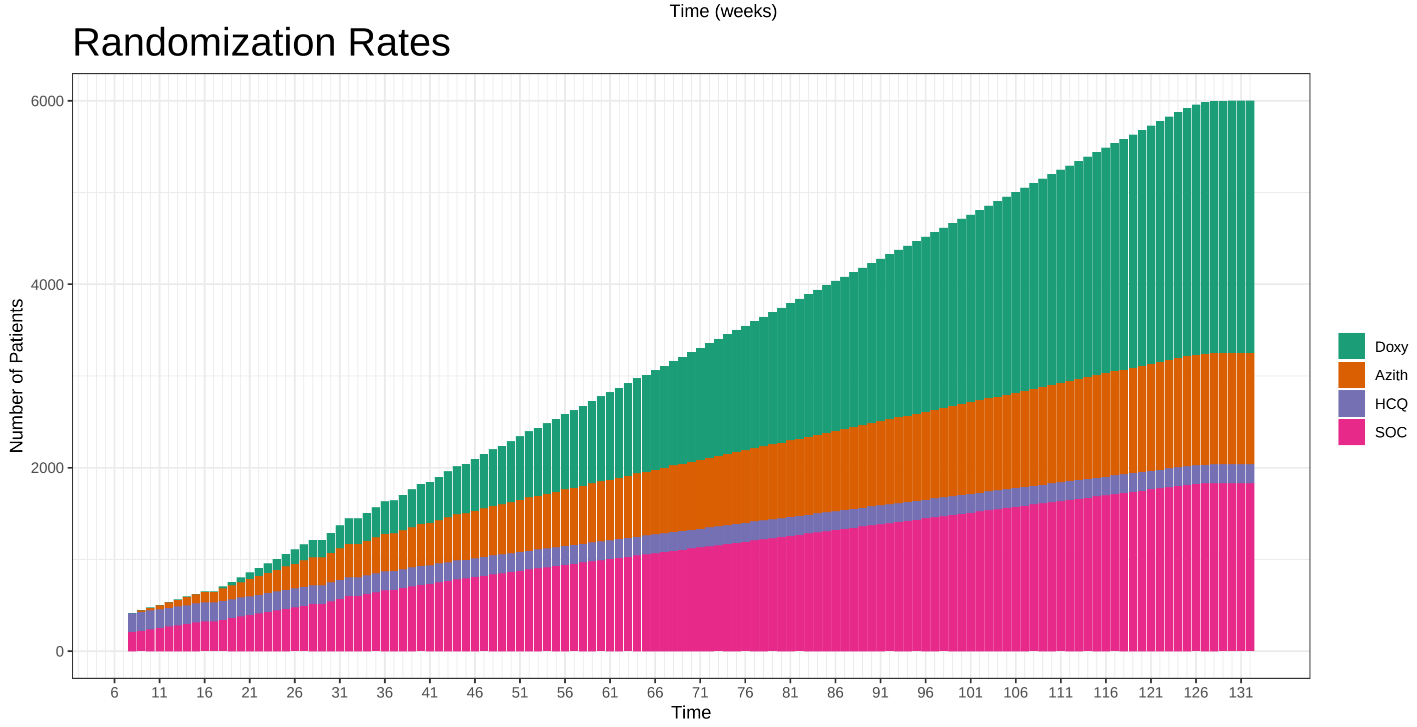

Hazard Ratios: HCQ: 1 Azith: 1.1 Doxy: 1.3

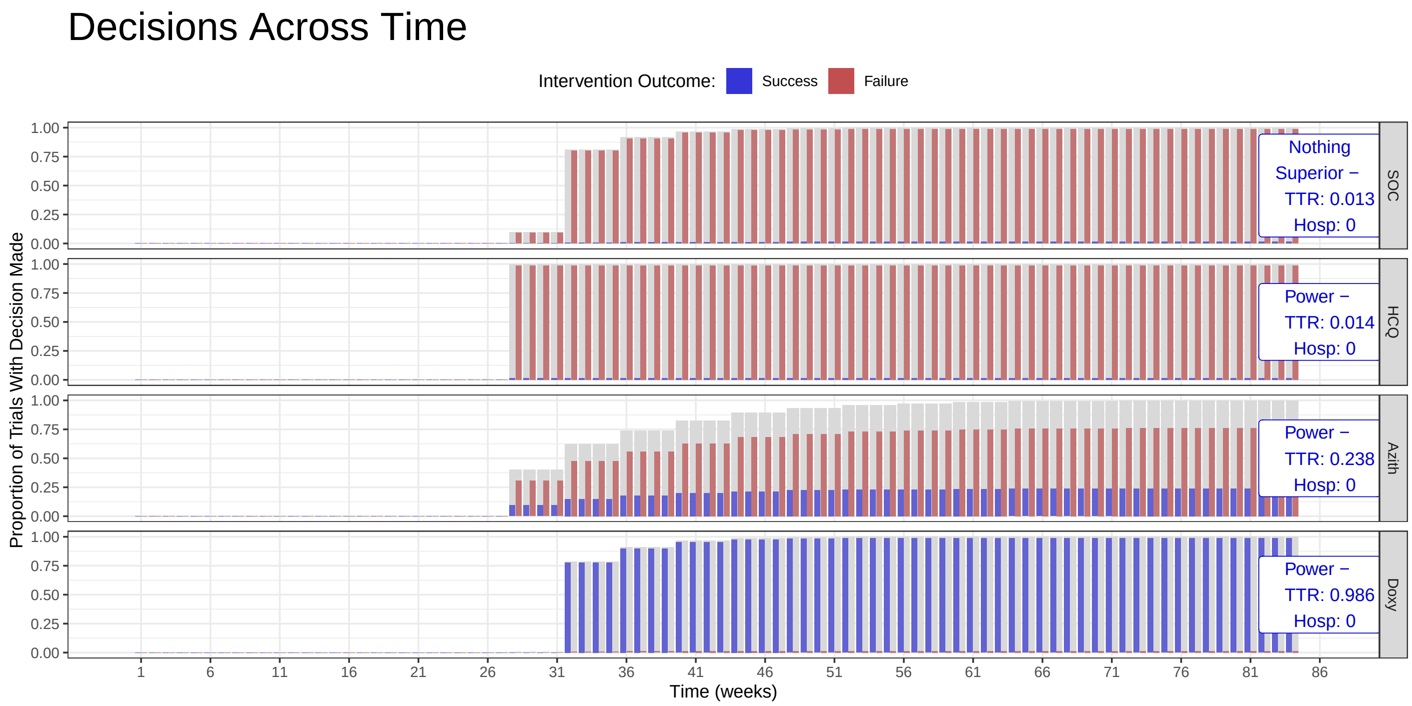

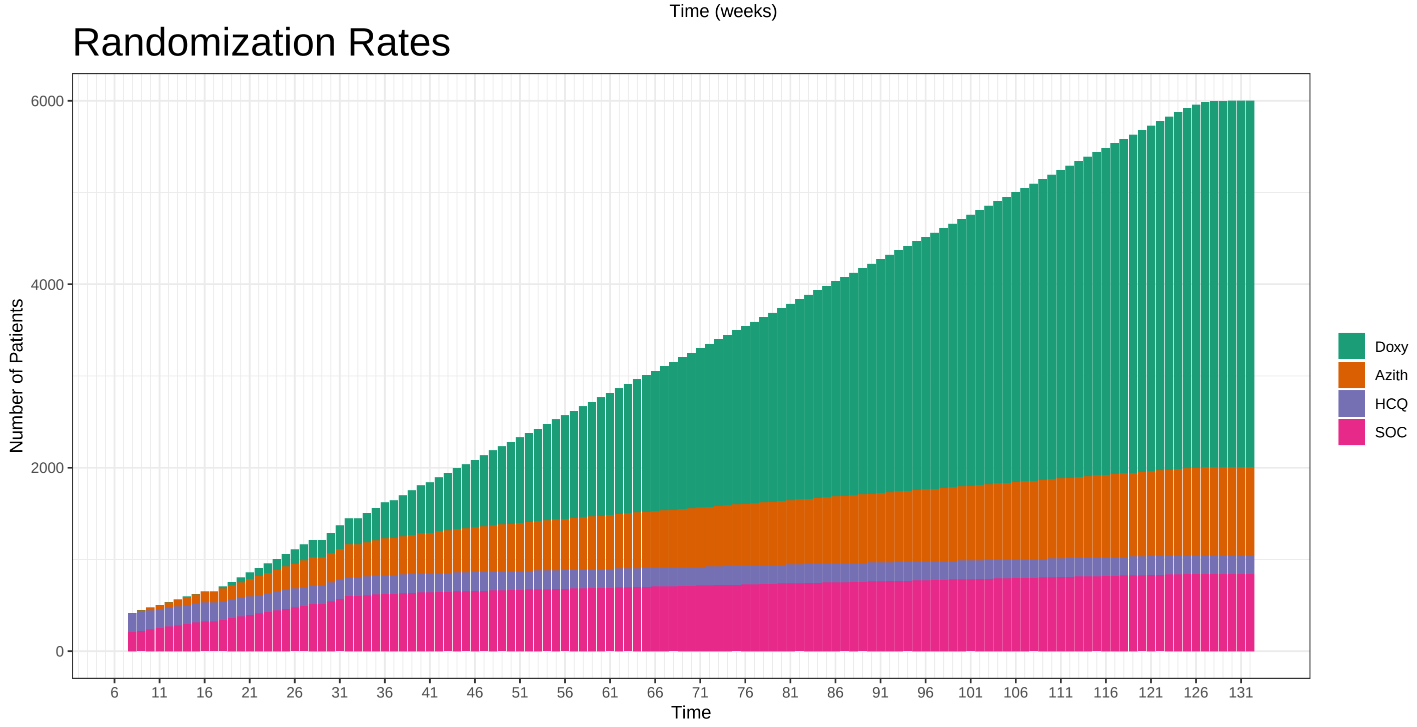

Hazard Ratios: HCQ: 1 Azith: 1.2 Doxy: 1

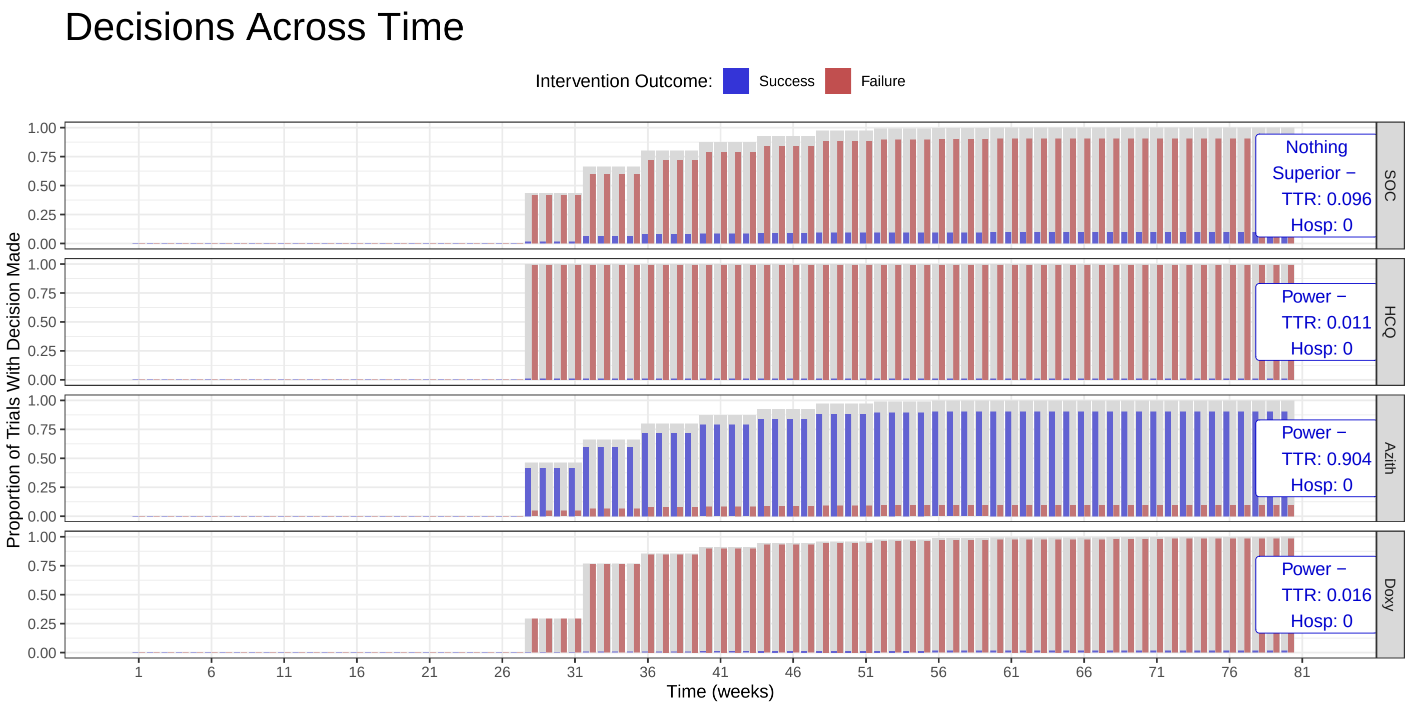

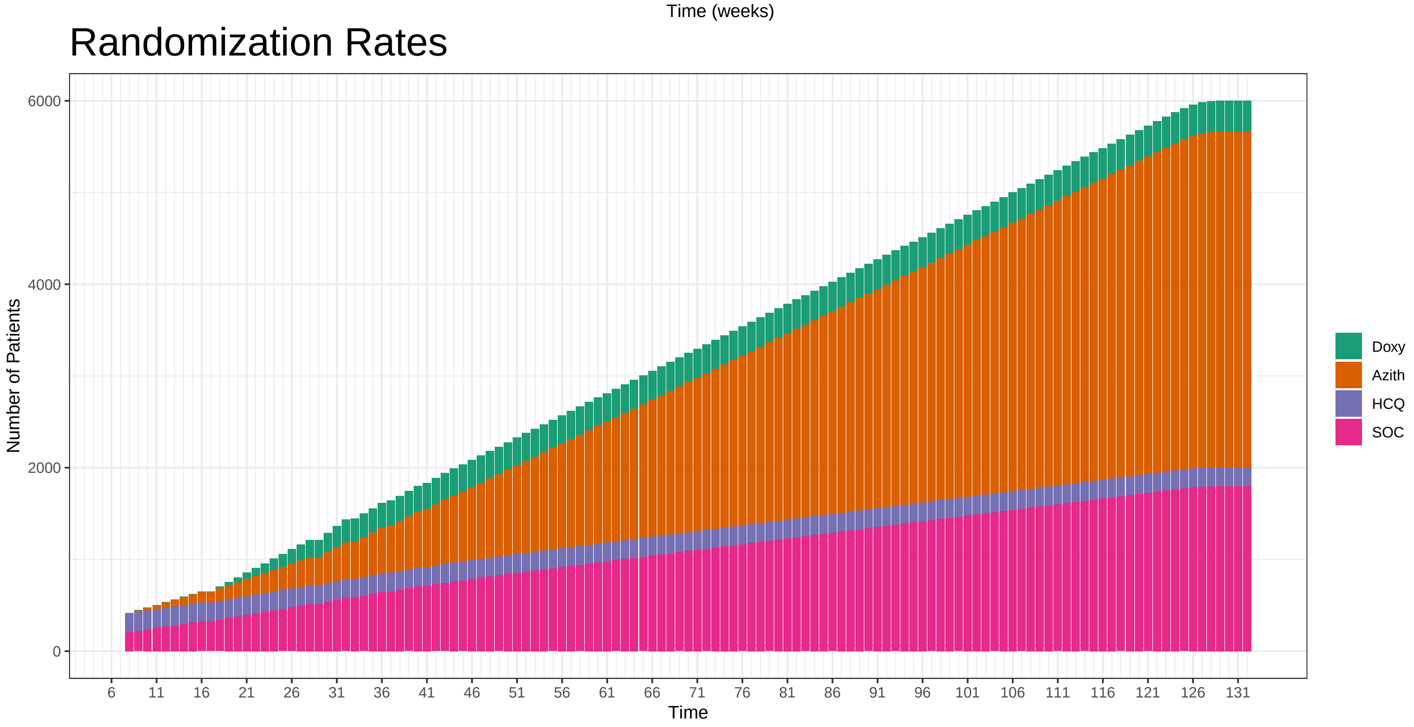

Hazard Ratios: HCQ: 1 Azith: 1.2 Doxy: 1.1

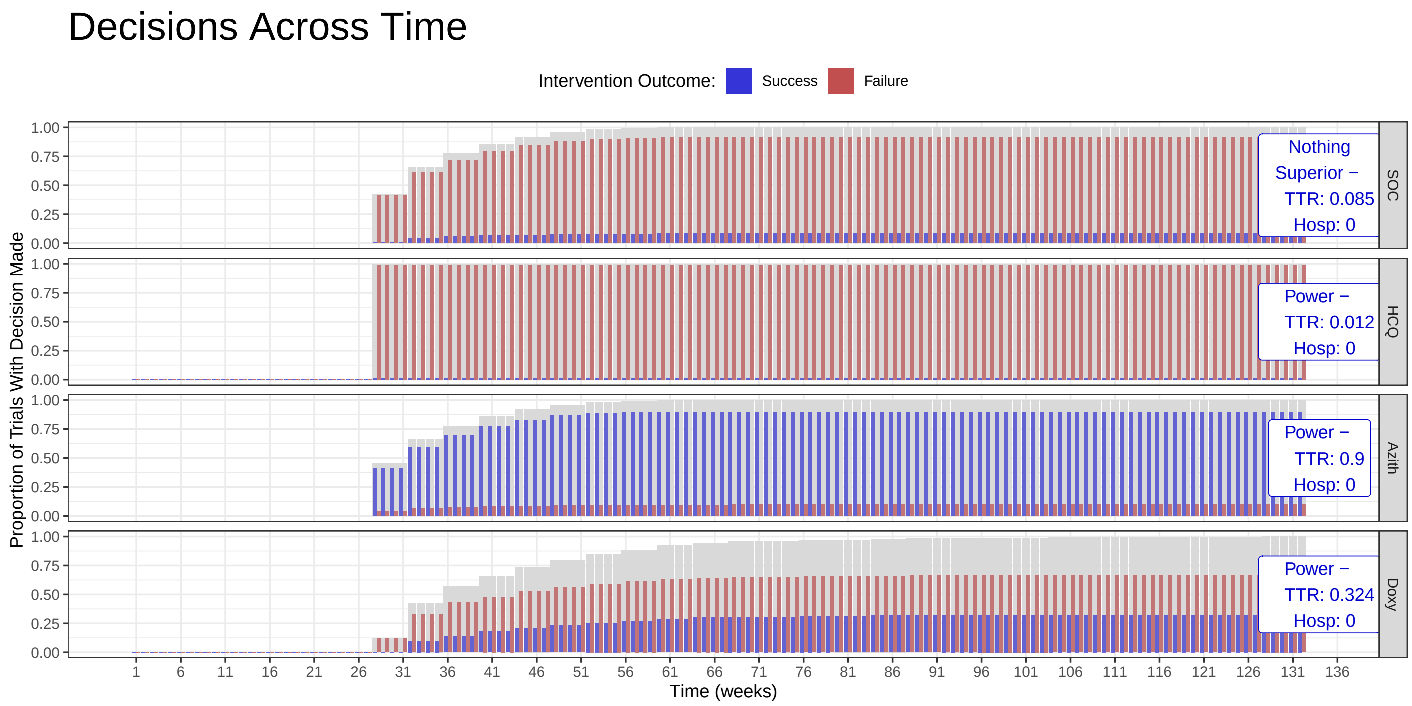

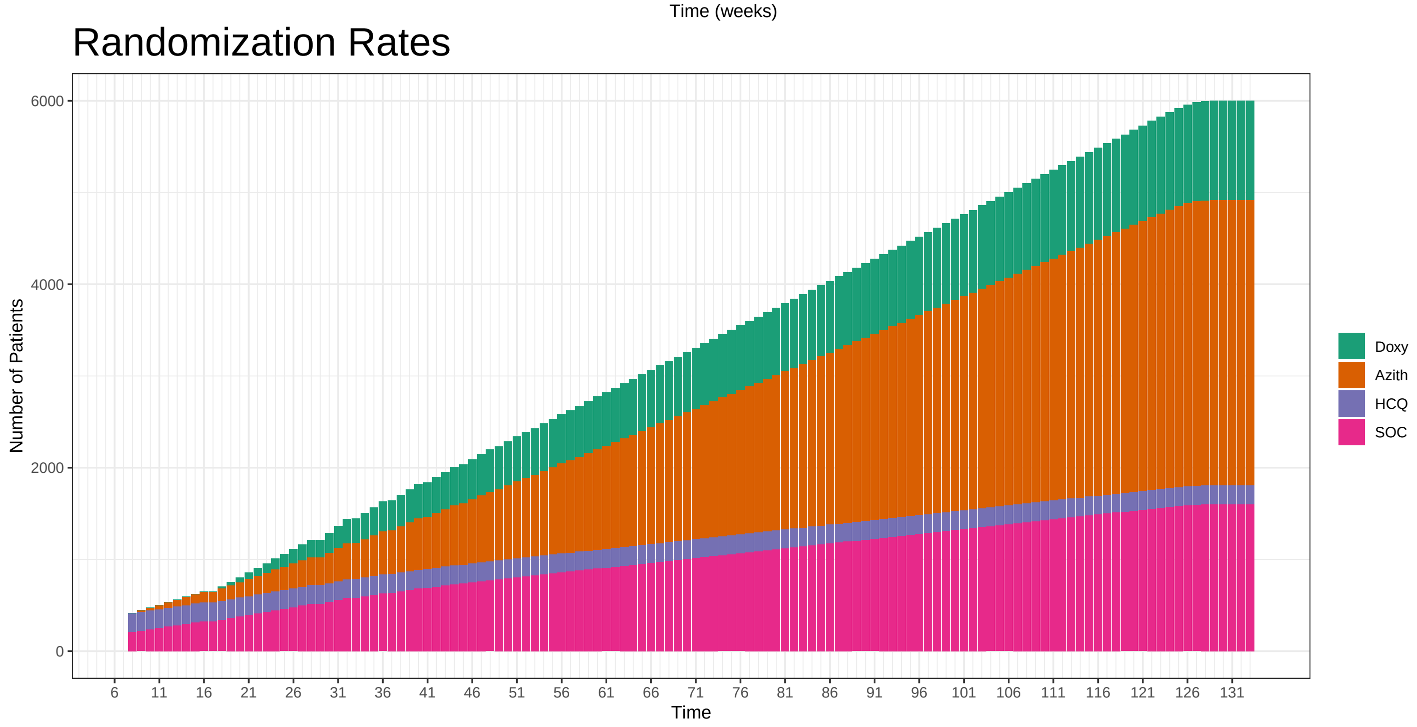

Hazard Ratios: HCQ: 1 Azith: 1.2 Doxy: 1.2

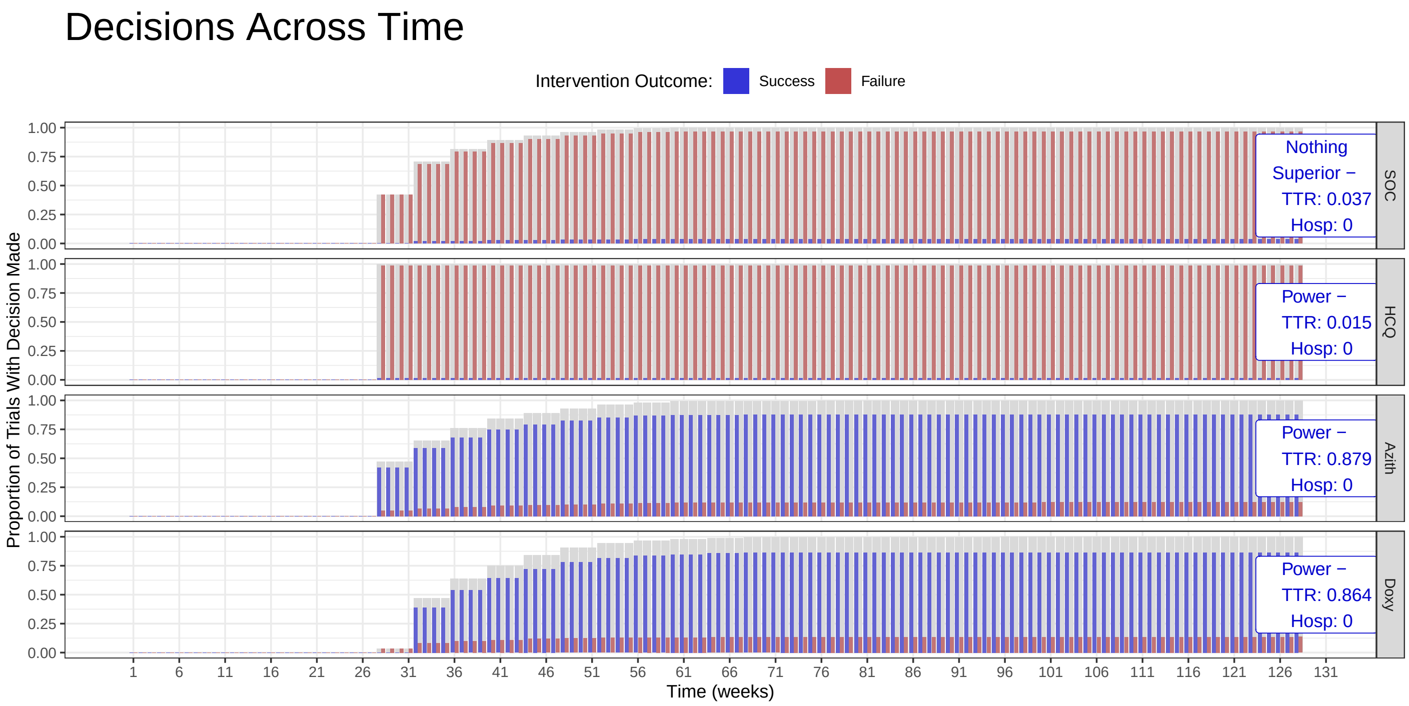

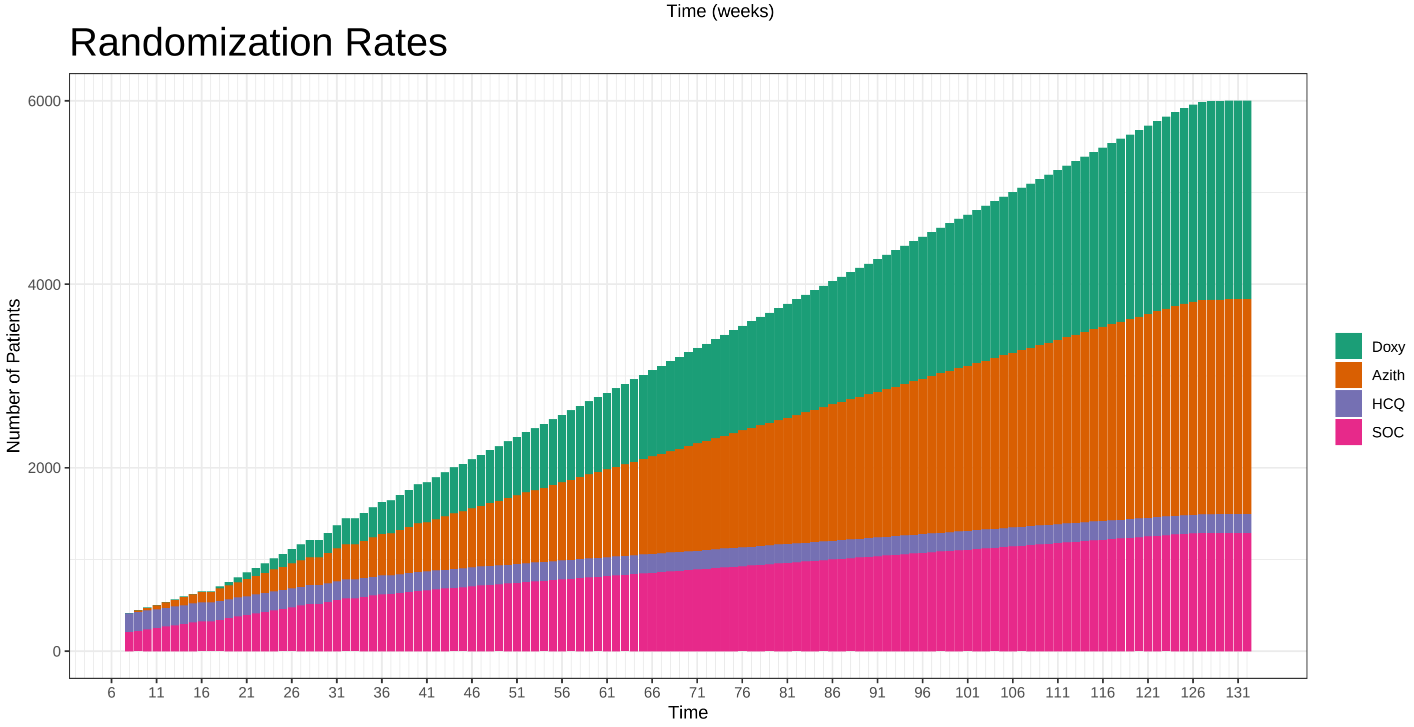

Hazard Ratios: HCQ: 1 Azith: 1.2 Doxy: 1.3

Decisions Across Time

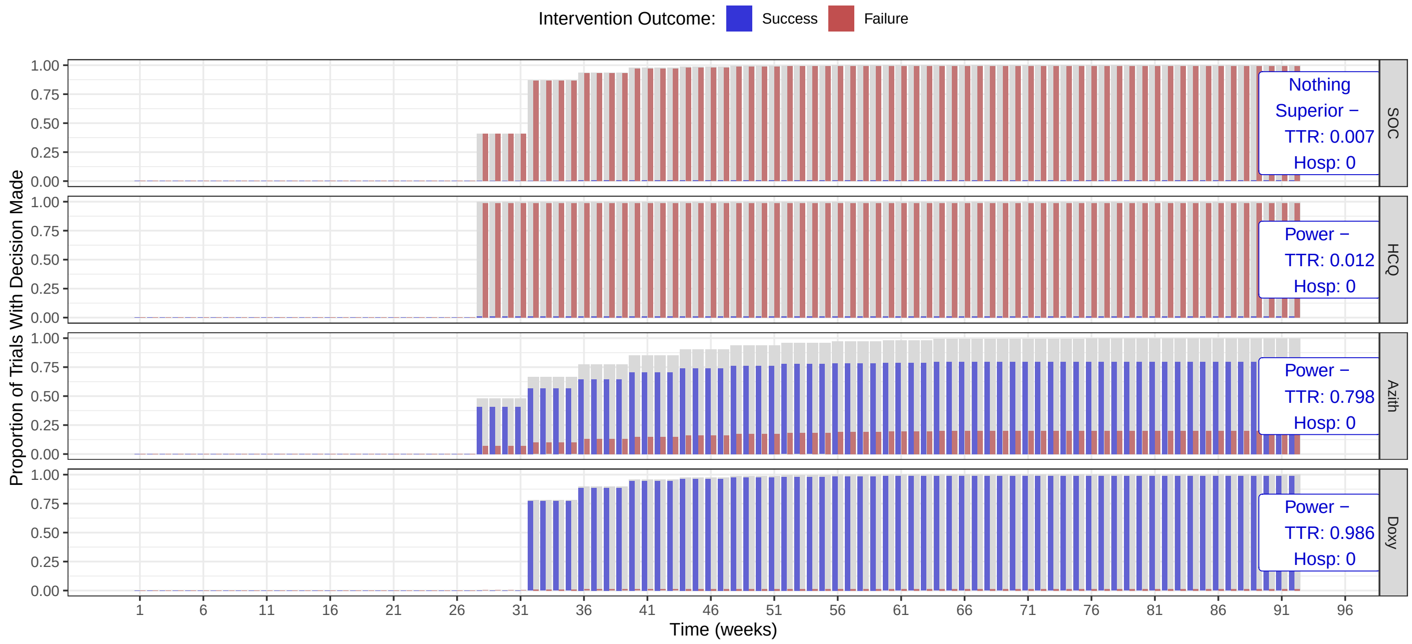

Randomization Rates

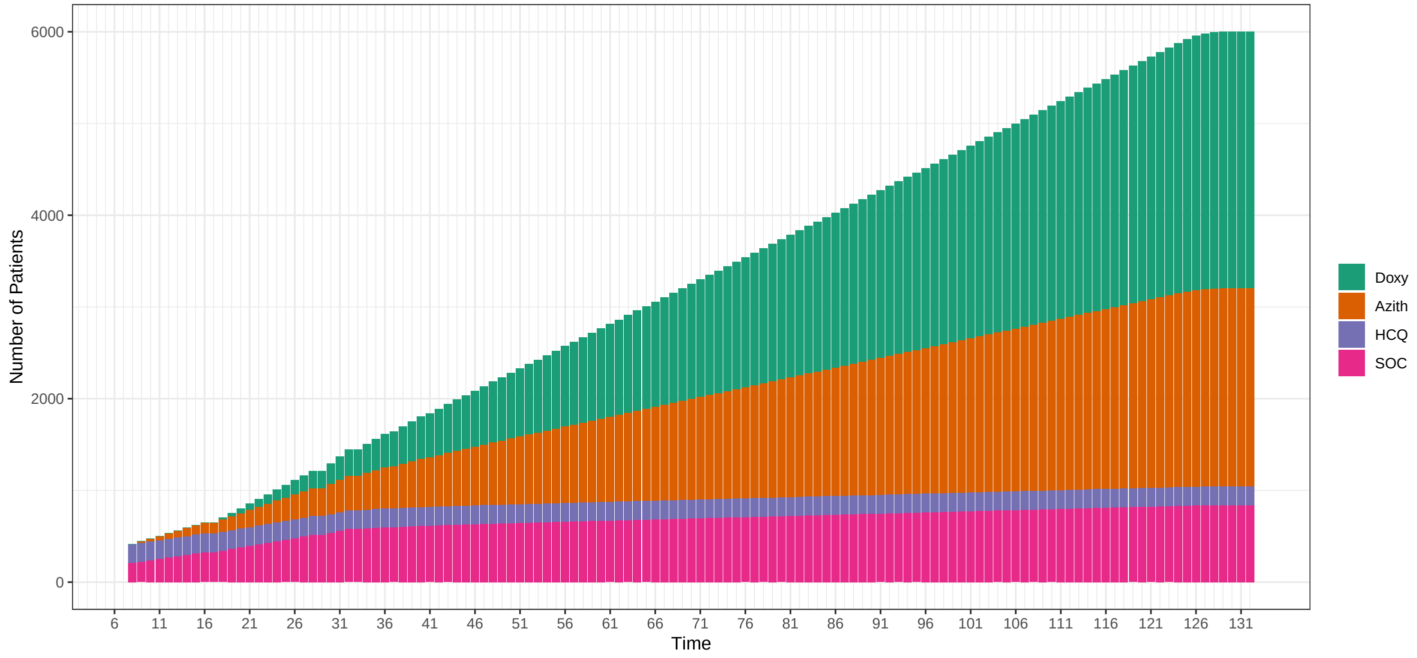

Hazard Ratios: HCQ: 1 Azith: 1.3 Doxy: 1

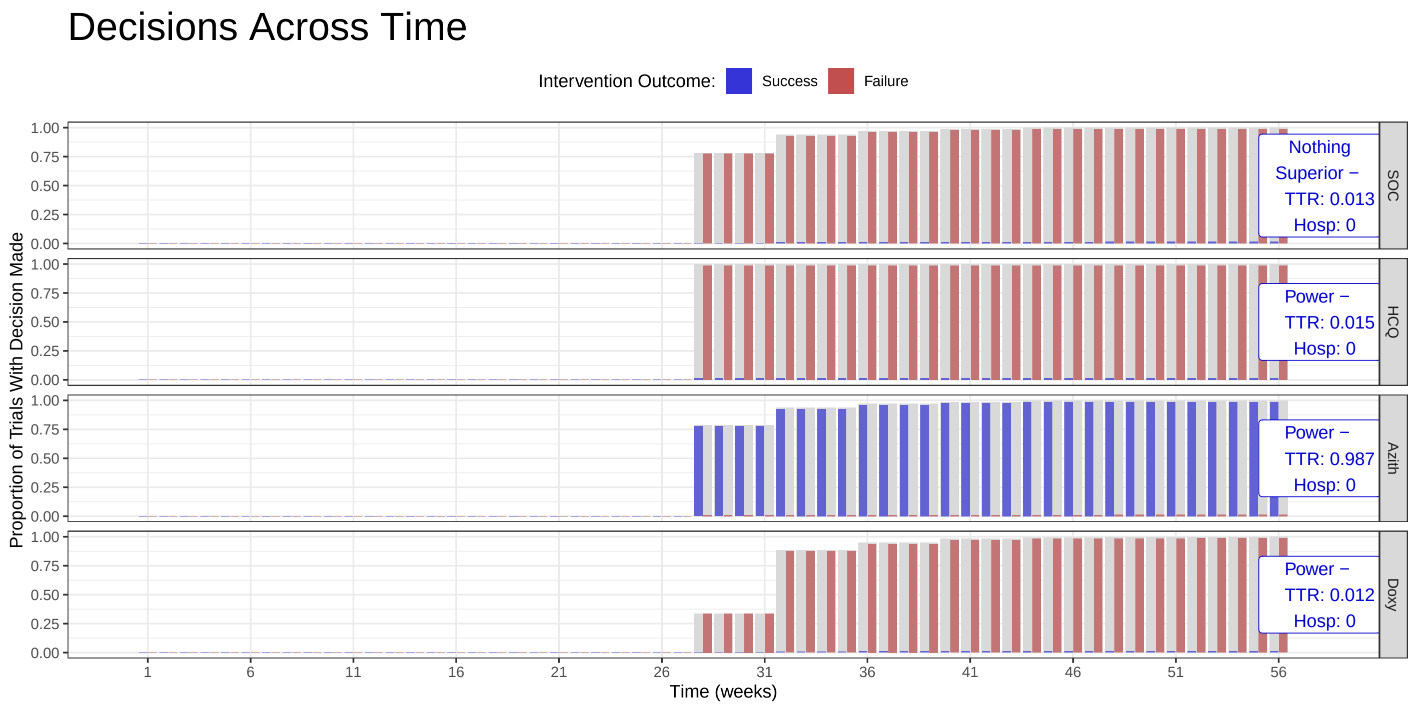

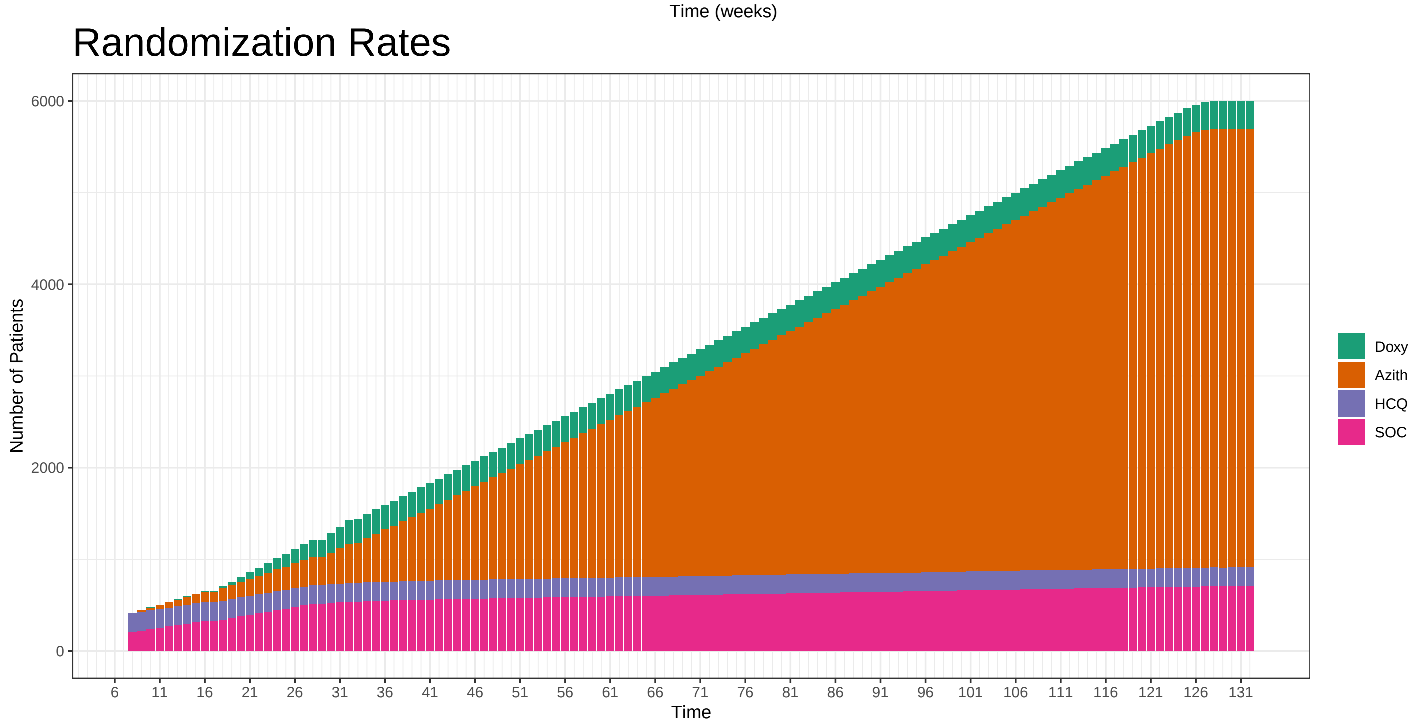

Hazard Ratios: HCQ: 1 Azith: 1.3 Doxy: 1.1

Decisions Across Time

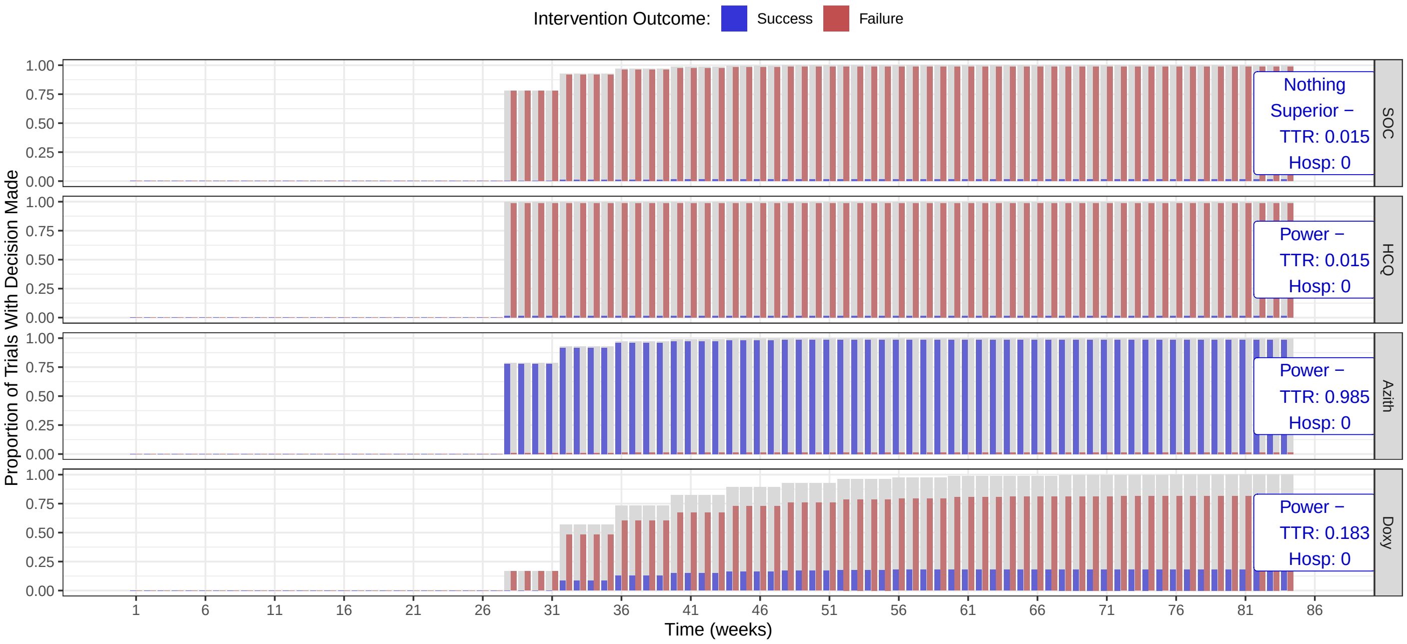

Randomization Rates

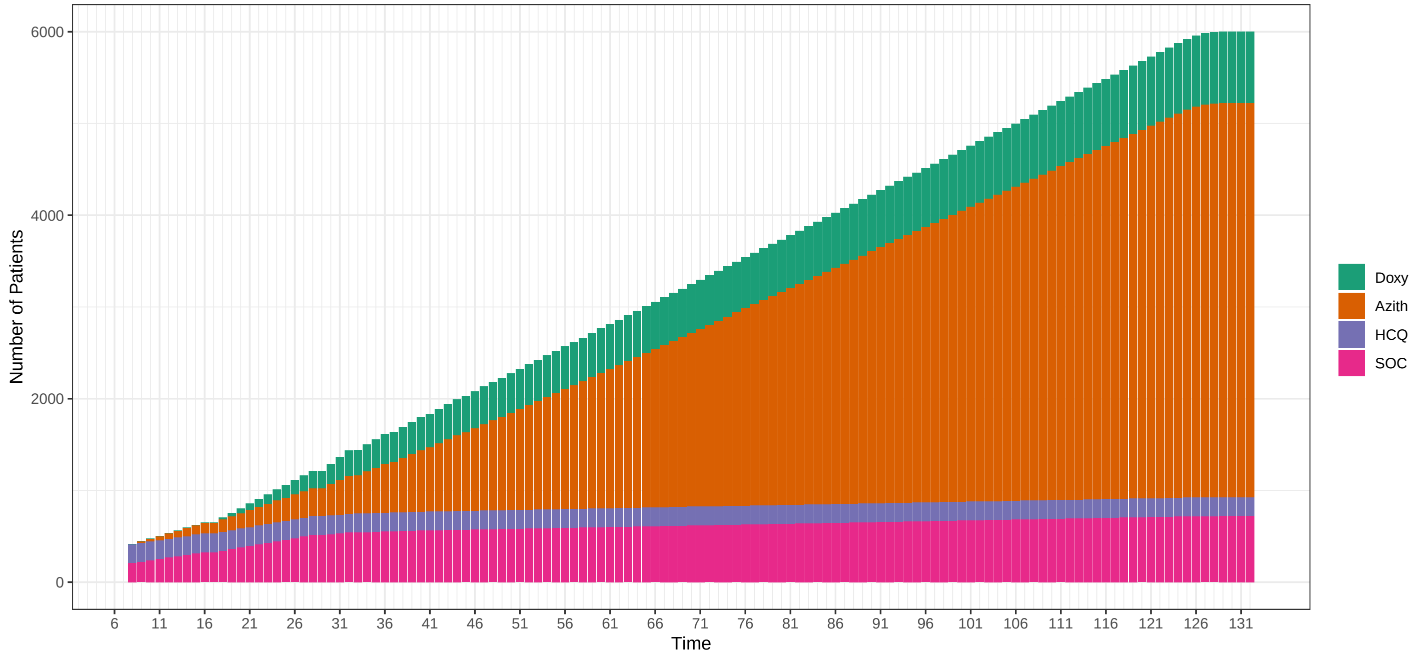

Hazard Ratios: HCQ: 1 Azith: 1.3 Doxy: 1.2

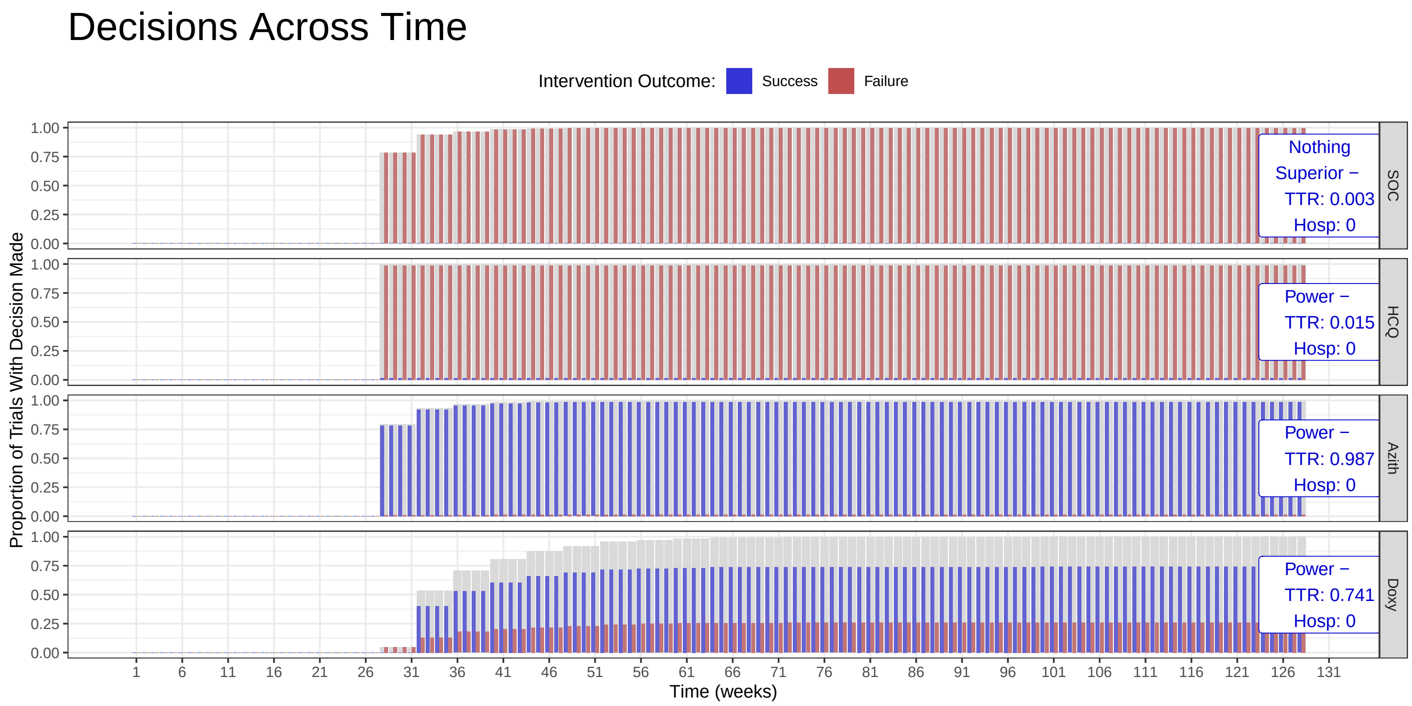

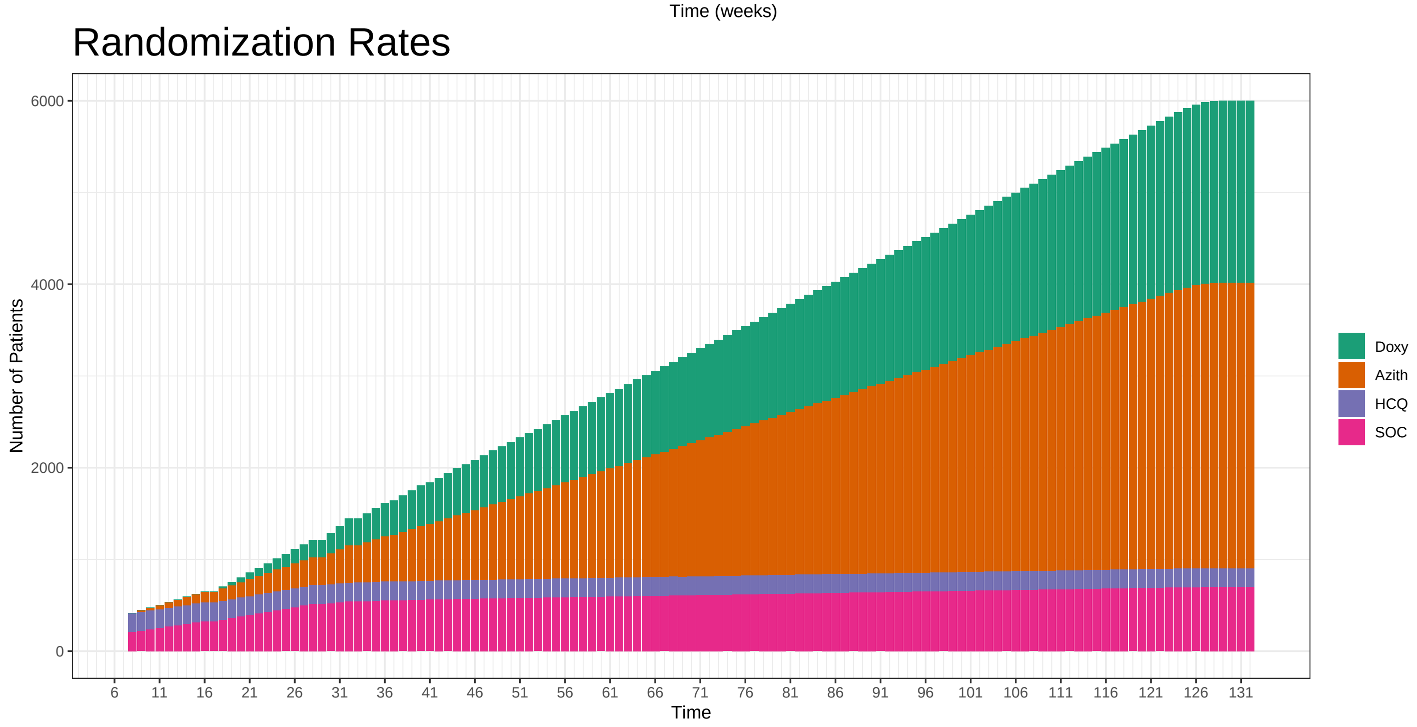

Hazard Ratios: HCQ: 1 Azith: 1.3 Doxy: 1.3

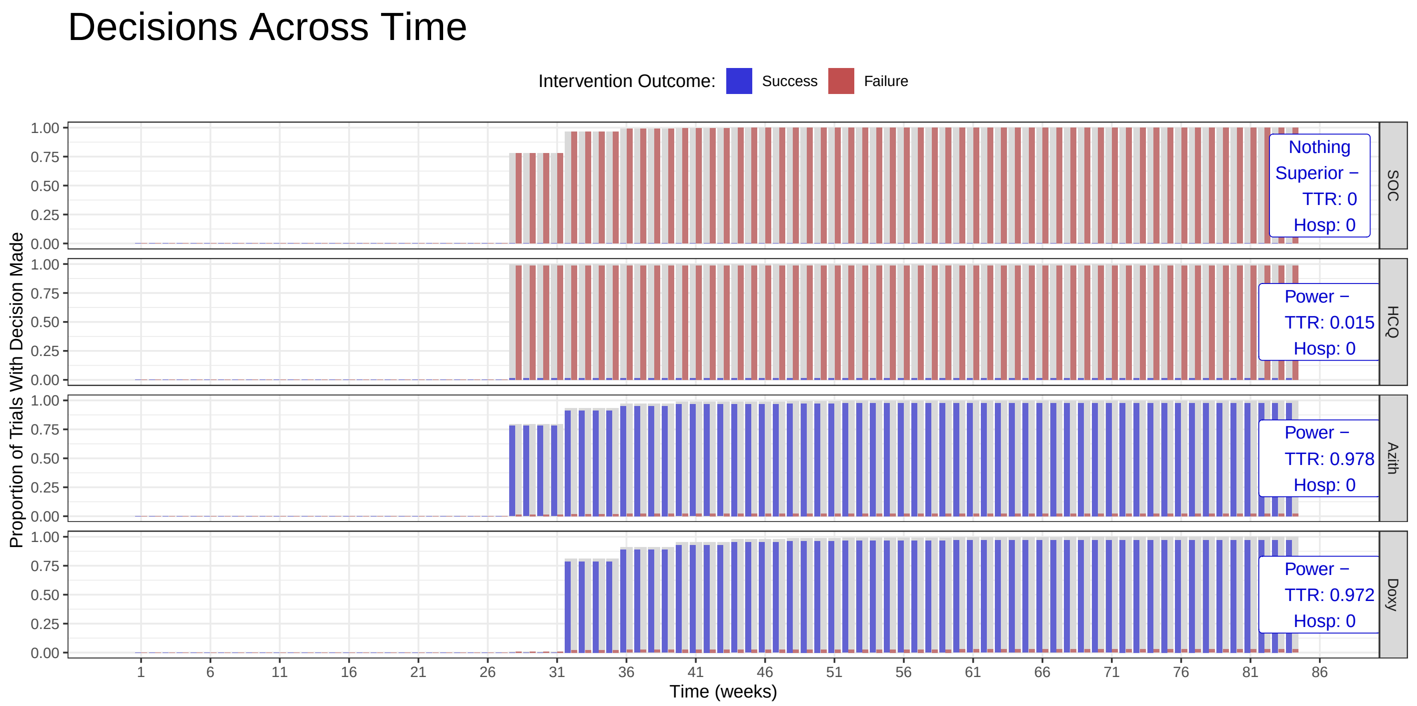

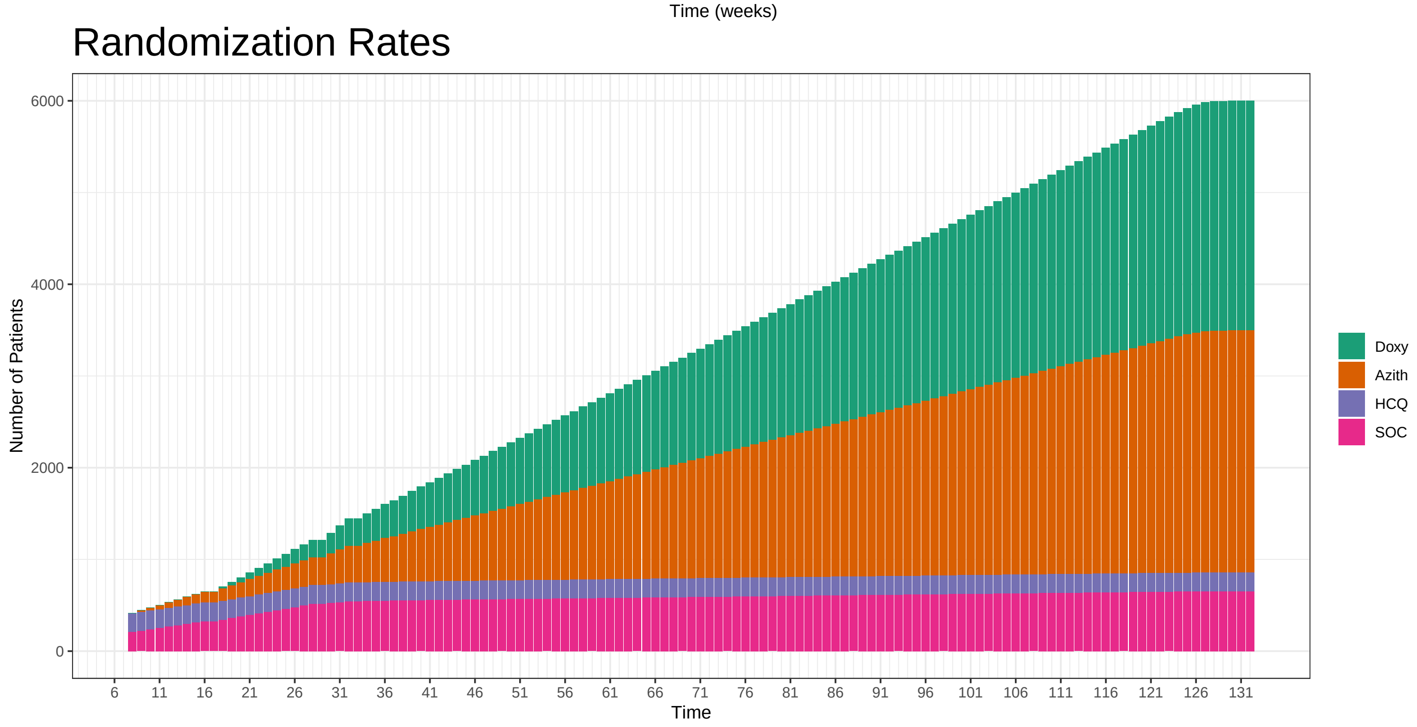

Hazard Ratios: HCQ: 1.1 Azith: 1.1 Doxy: 1.1

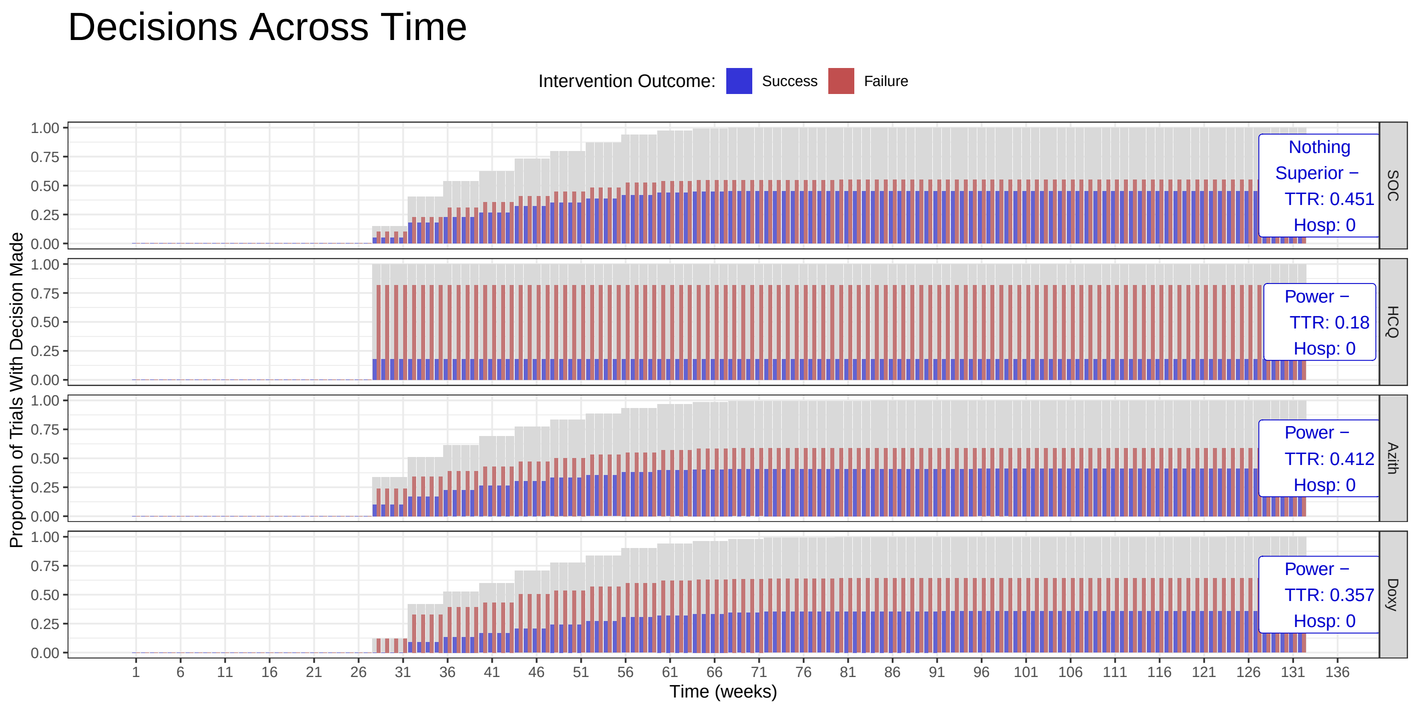

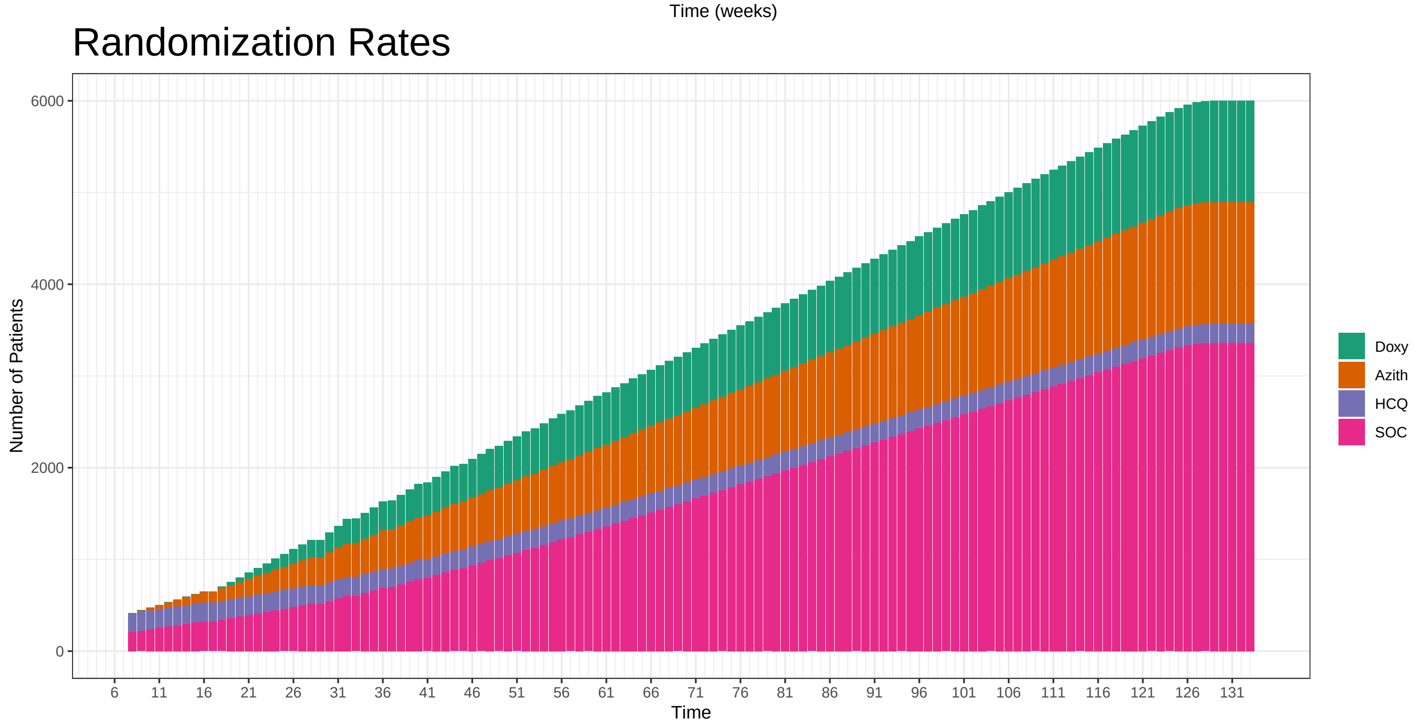

Hazard Ratios: HCQ: 1.2 Azith: 1.2 Doxy: 1.2

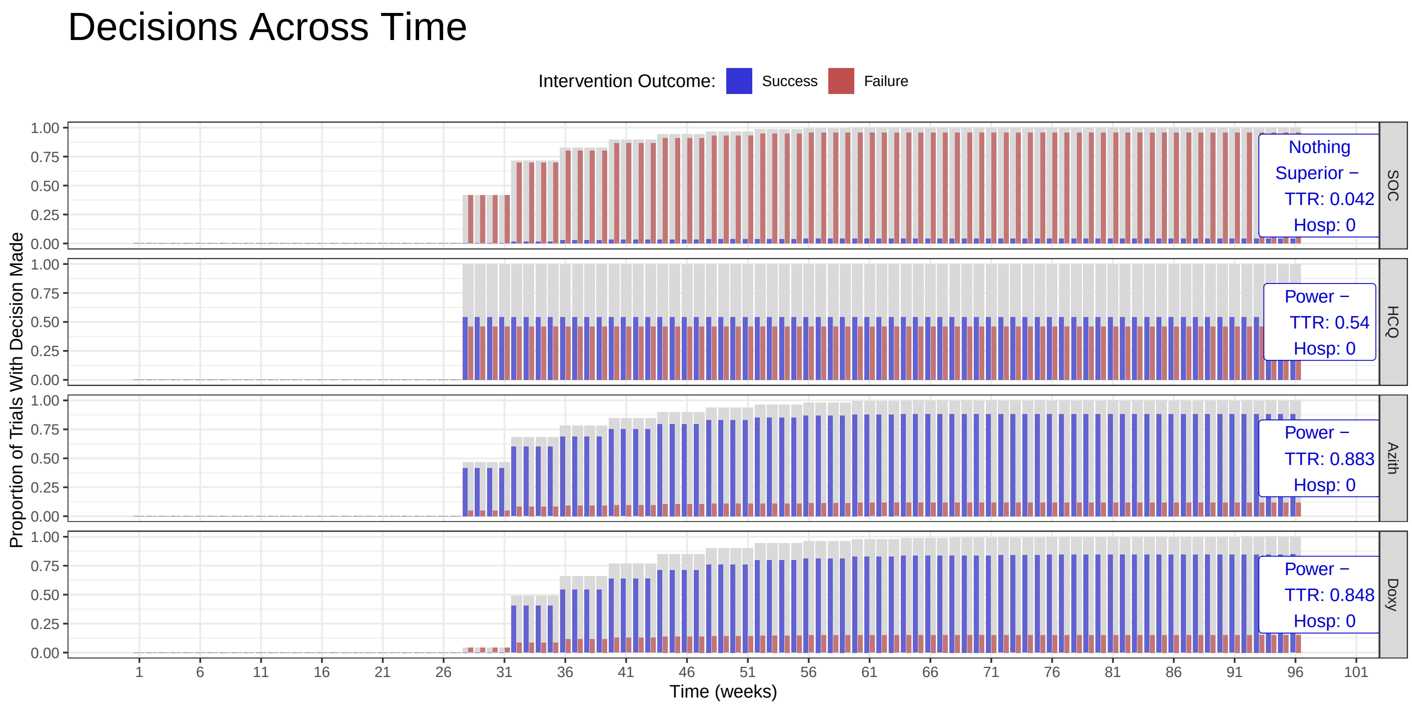

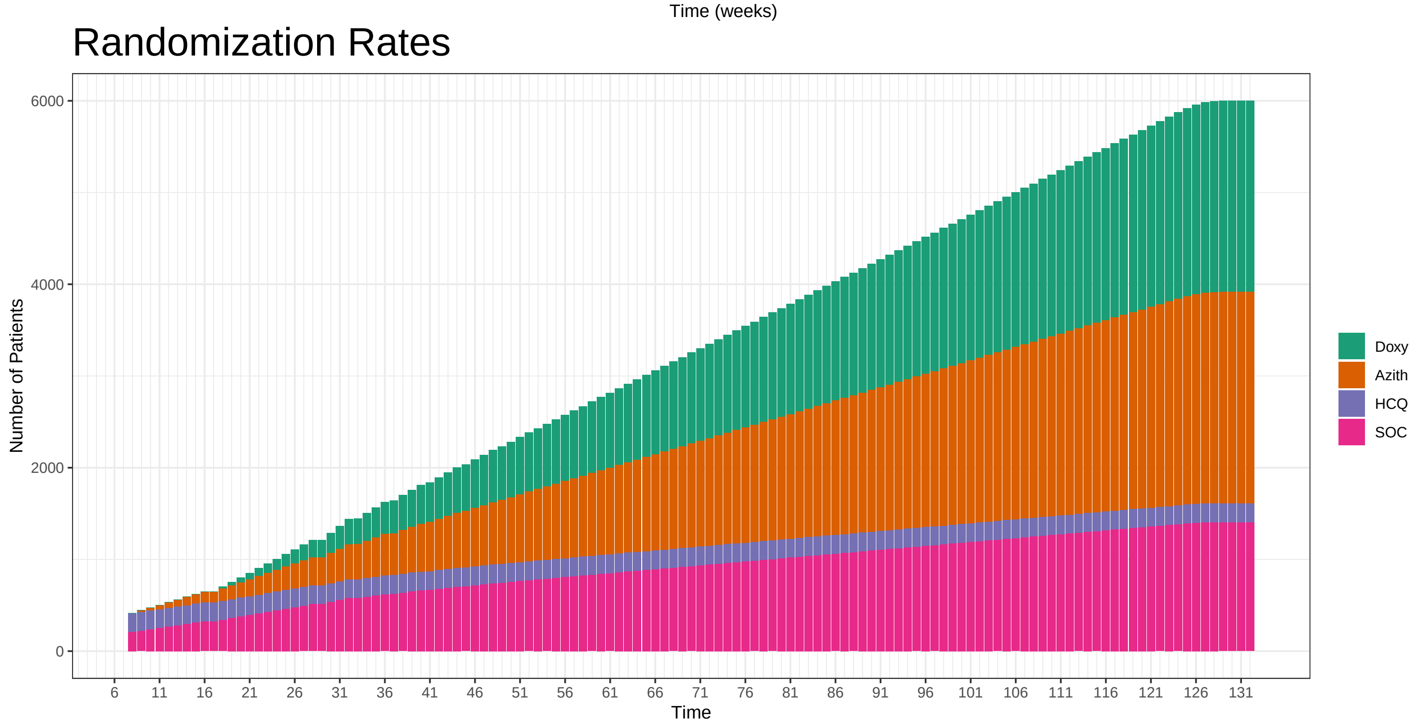

Hazard Ratios: HCQ: 1.3 Azith: 1.3 Doxy: 1.3

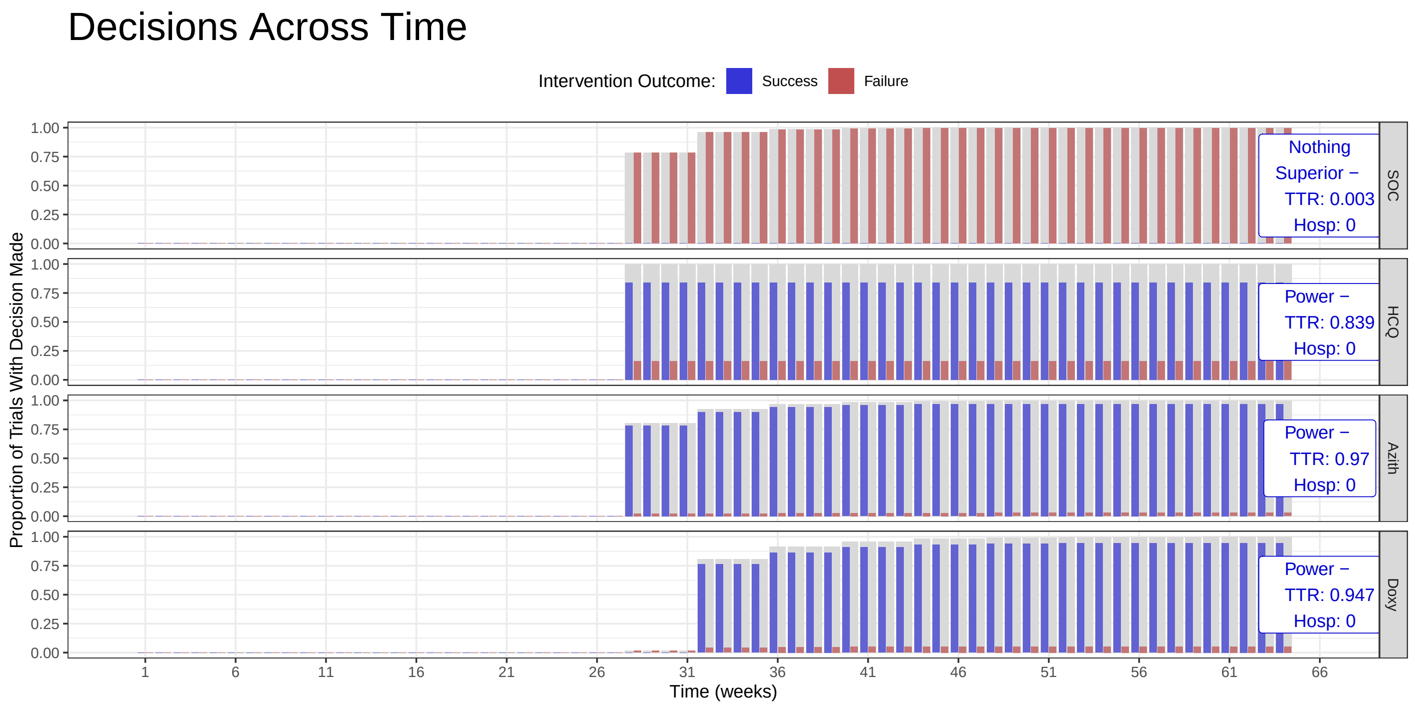

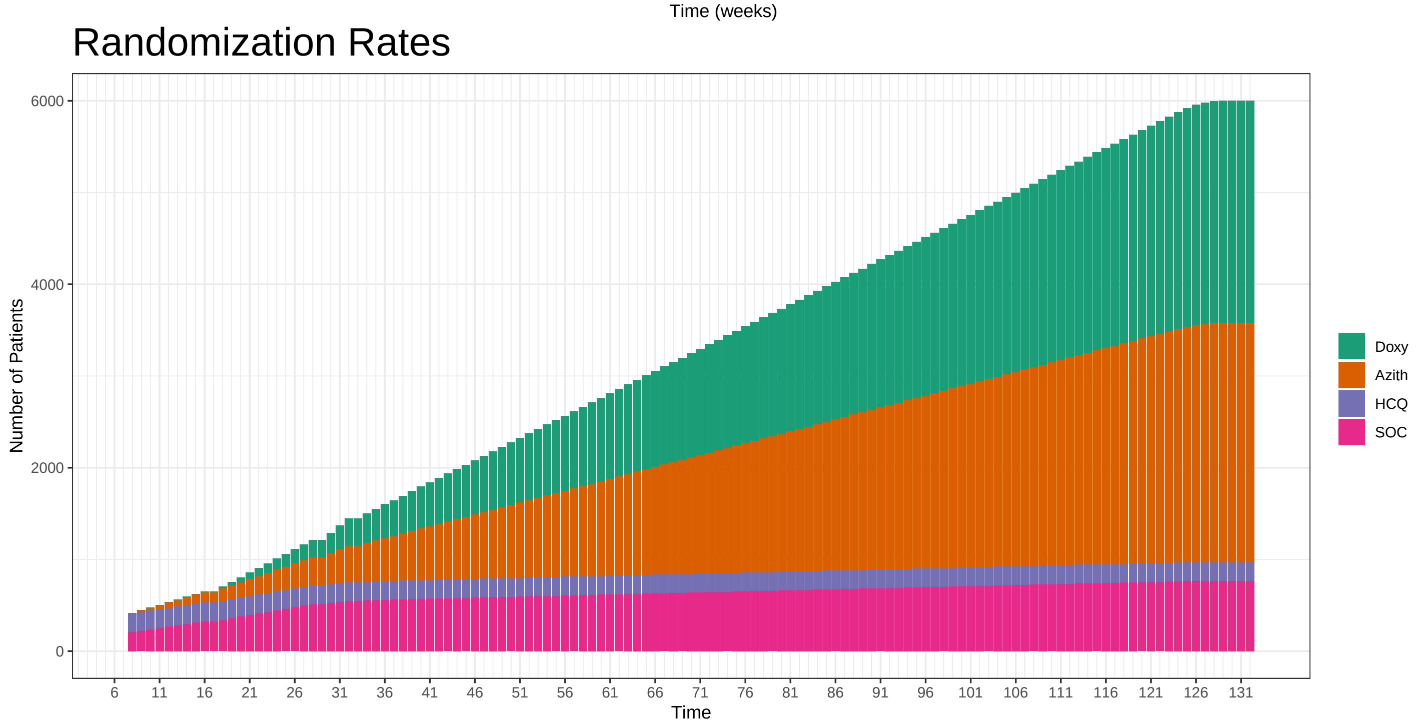

5.3 OCs Additional Scenarios: Null Effect Hospitalization

Principle OCs

Berry Consultants

10/26/2020

These operating characteristics are based on 1000 simulations per scenario across a variety of assumed treatment effects for experimental arms in the trial. The true number of patients randomized per day from April 1, 2020 to October 19, 2020. After this period is over, the simulations assume a 50 patient per week accrual rate. In absence of a definitive rule, these simulations conduct an interim every 28 days. In addition, the true day of enrollment stopping of HCQ is used as well as the days for opening enrollment to the Azithromycin and Doxycycline arms. As such, up to at least October 19, 2020 (day 199) of the trial, the enrollment rates to each arm in these simulations are expected to align closely with the true enrollment rates to the arms in the trial.

Median TTR	Hazard Ratio			Power for TTR				Hospitalization Rate Benefit (%)			Power for TTR & Hosp		
SOC	HCQ	Azith	Doxy	SOC	HCQ	Azith	Doxy	HCQ	Azith	Doxy	HCQ	Azith	Doxy
9	1.1	1.1	1.1	0.416	0.179	0.428	0.398	0	0	0	0.002	0.008	0.012
9	1.2	1.2	1.2	0.035	0.540	0.879	0.857	0	0	0	0.008	0.019	0.016
9	1.3	1.3	1.3	0.002	0.839	0.975	0.959	0	0	0	0.012	0.019	0.029

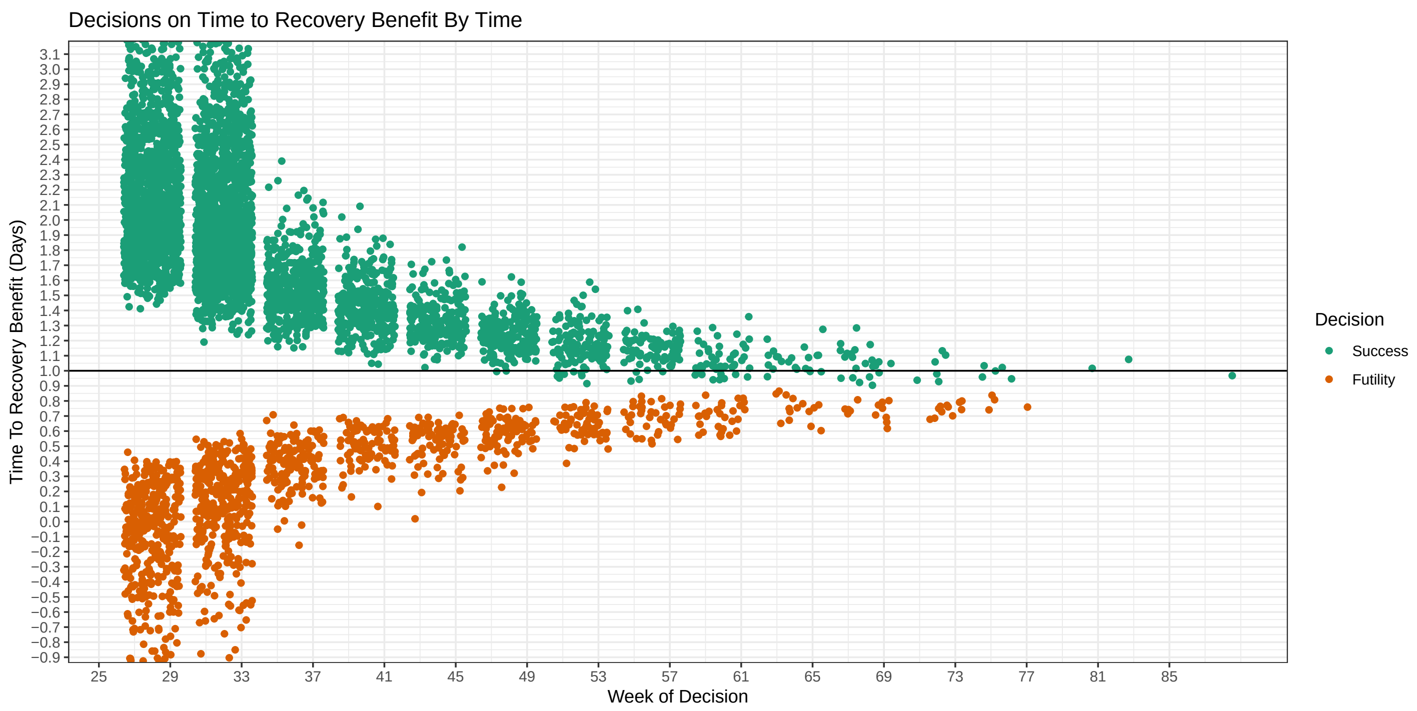

Decisions on Time to Recovery Benefit By Time
Time To Recovery Benefit (Days)
Week of Decision
Decision
Success
Futility

Hazard Ratios: HCQ: 1.1 Azith: 1.1 Doxy: 1.1

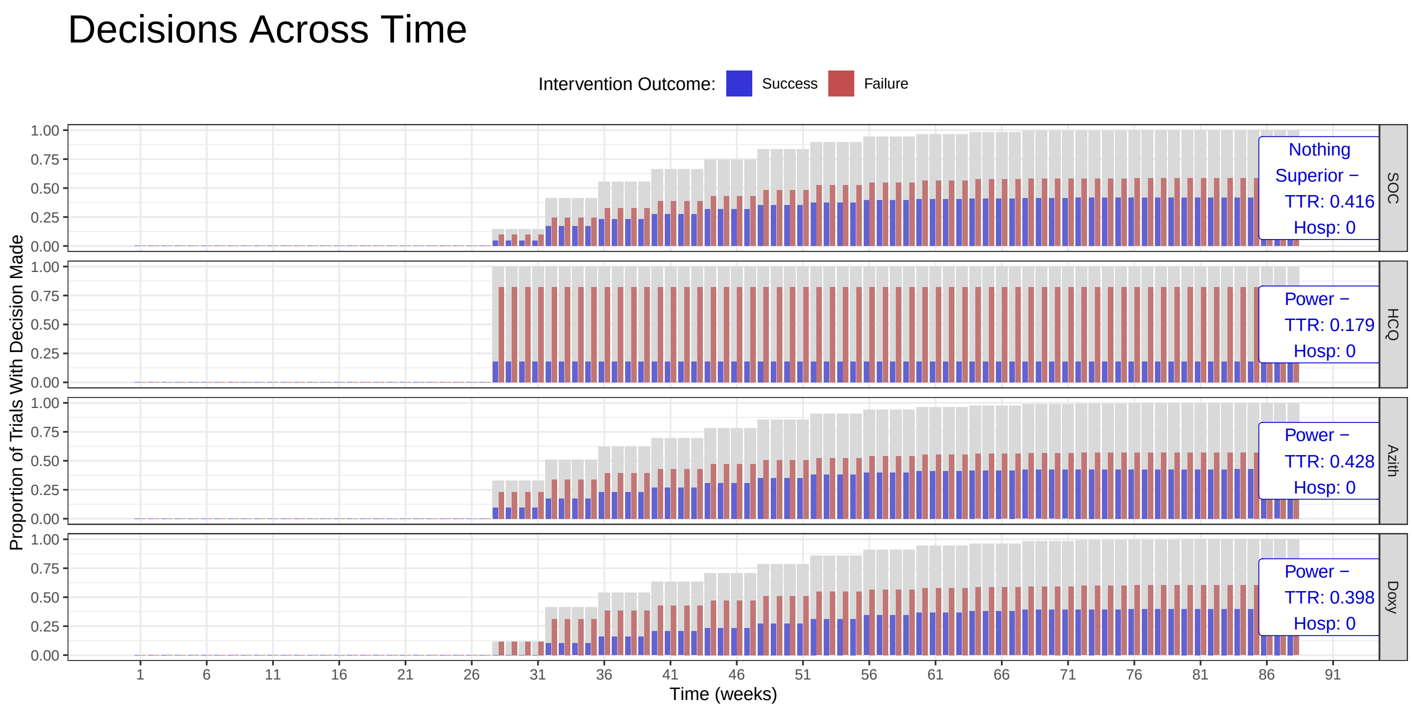

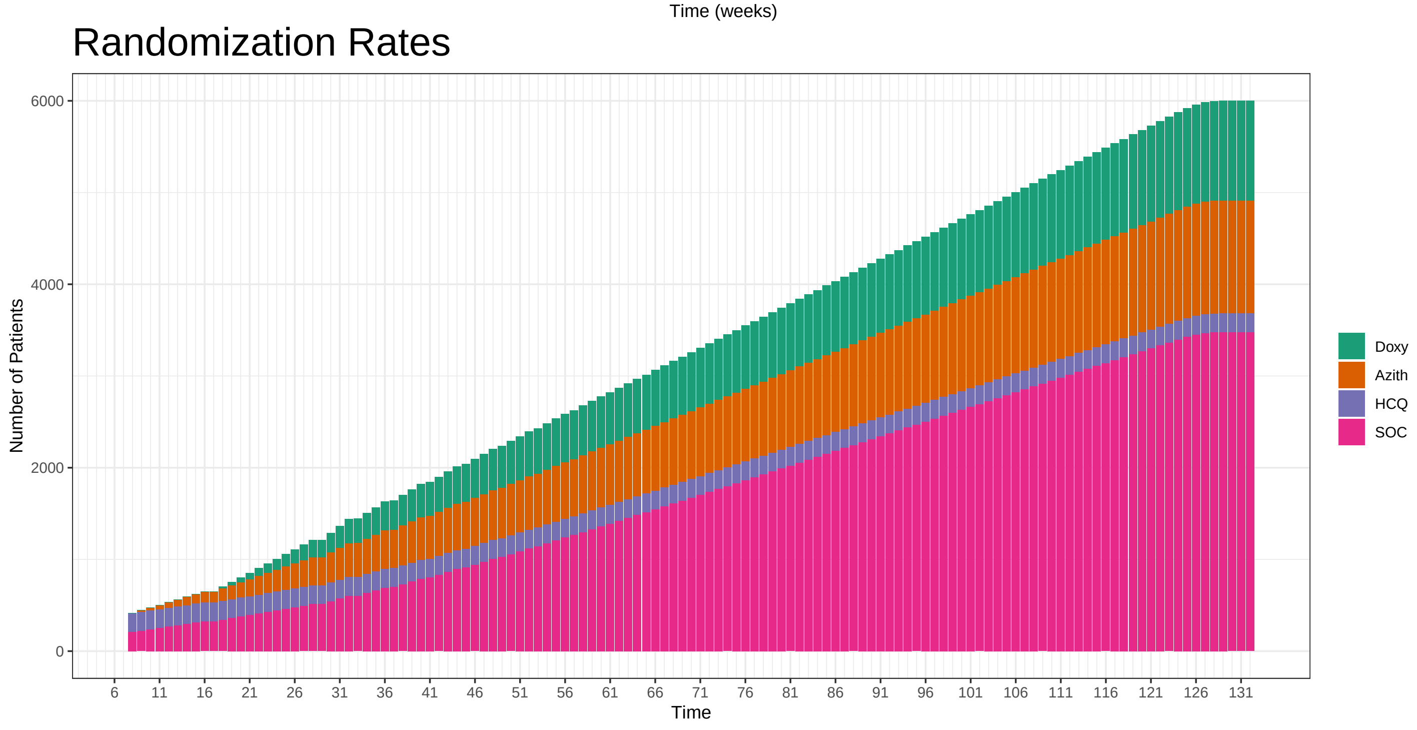

Hazard Ratios: HCQ: 1.2 Azith: 1.2 Doxy: 1.2

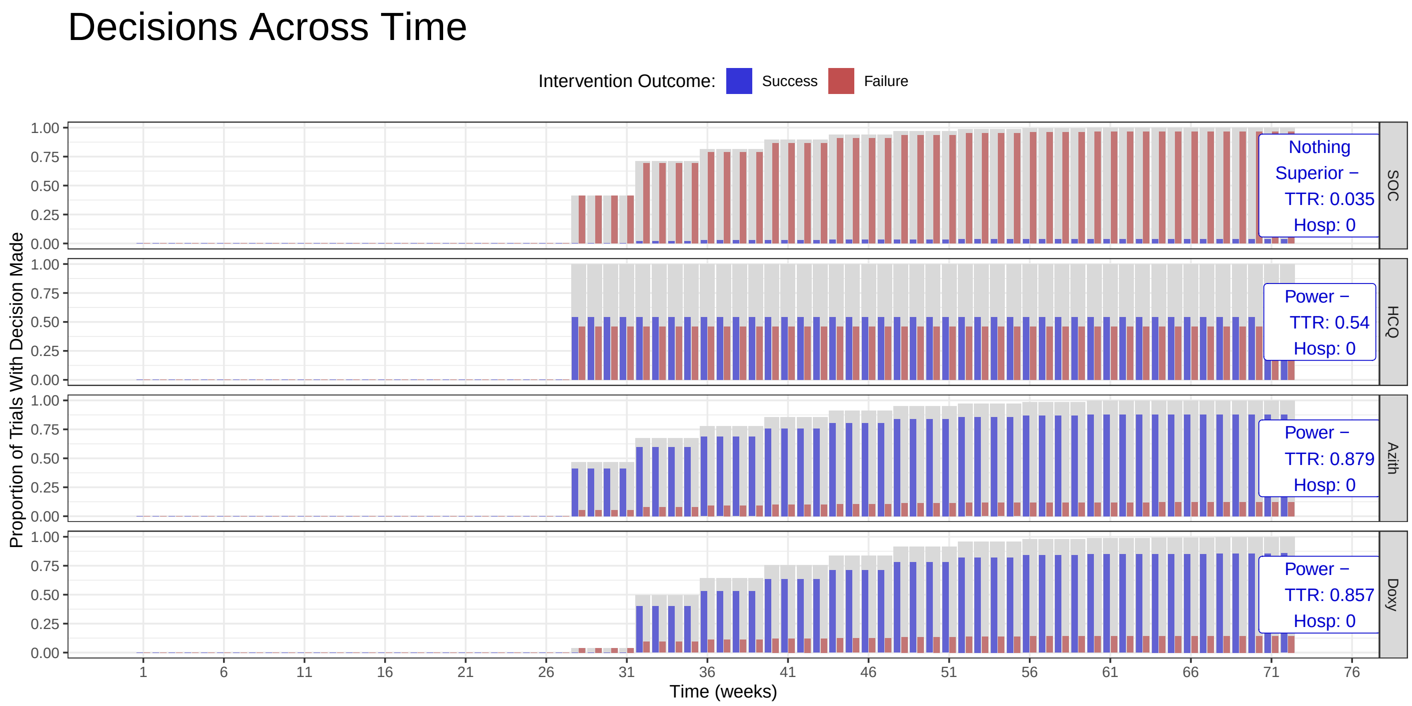

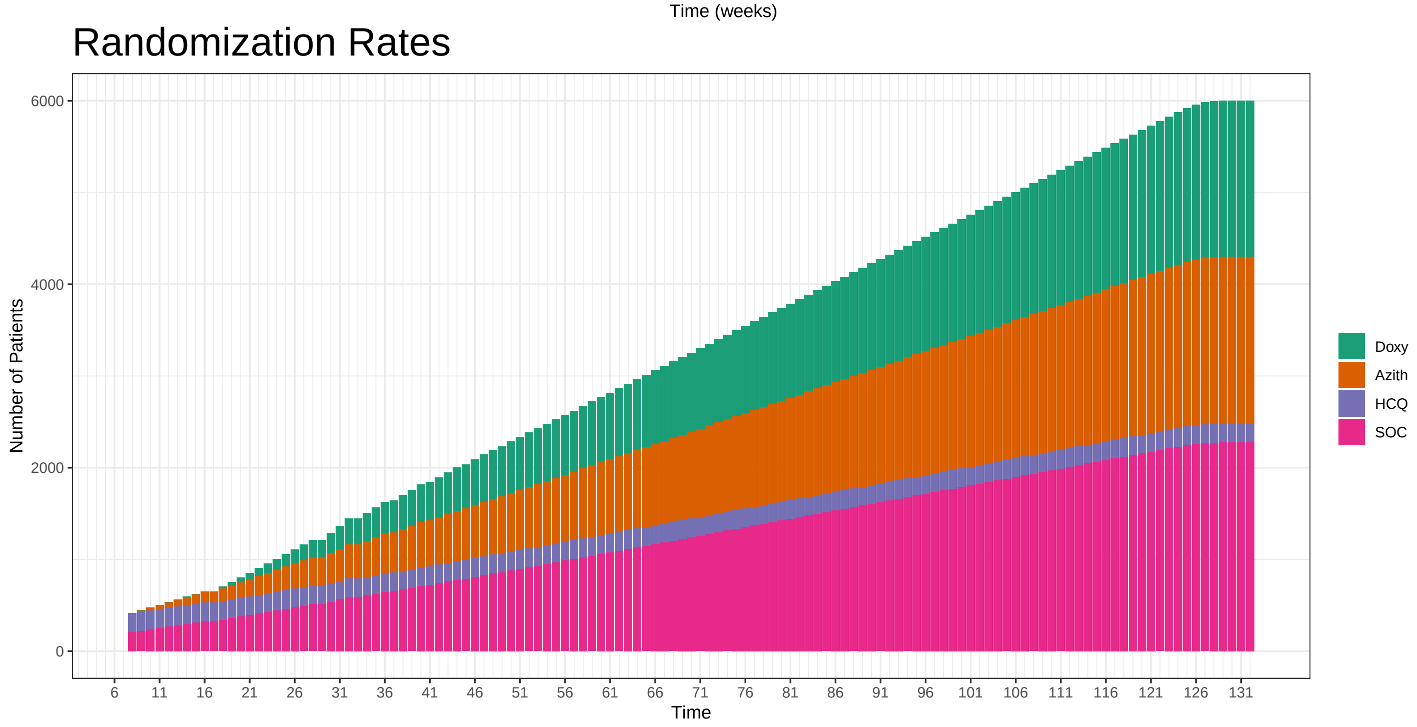

Hazard Ratios: HCQ: 1.3 Azith: 1.3 Doxy: 1.3

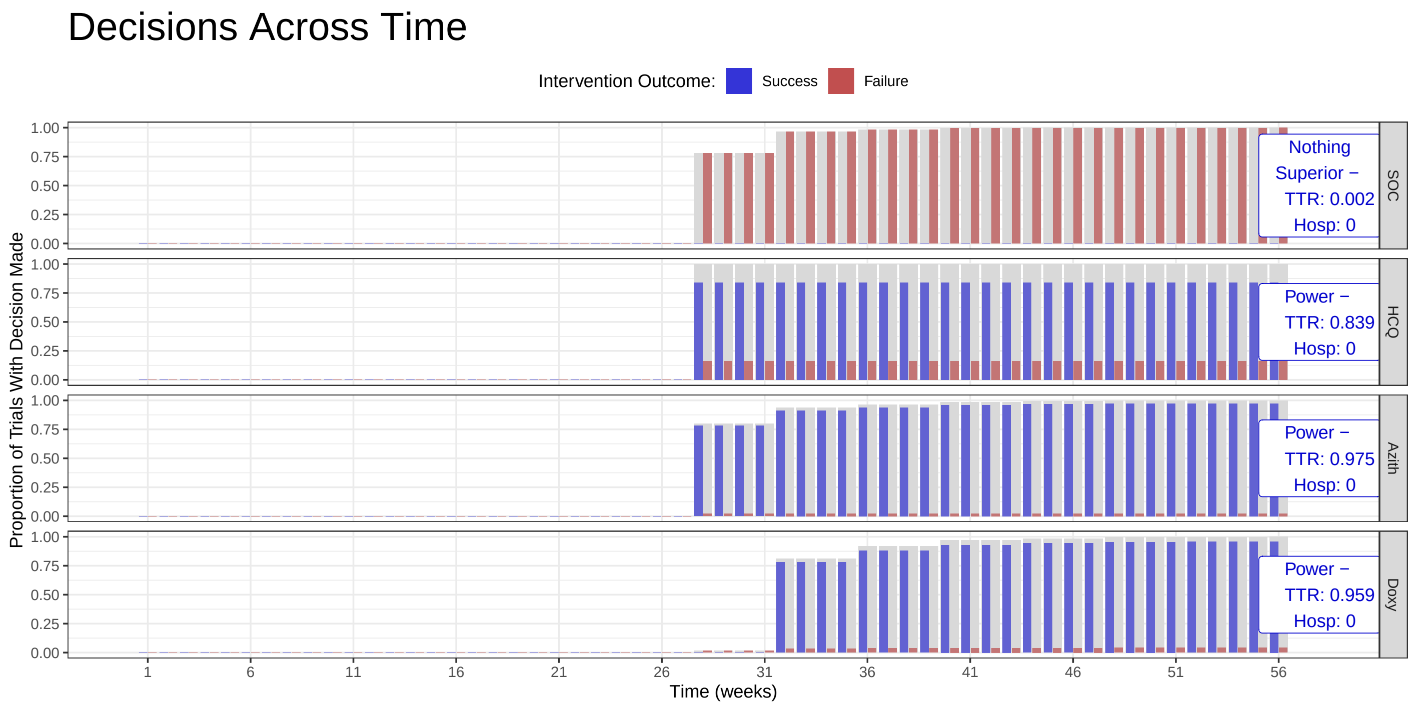

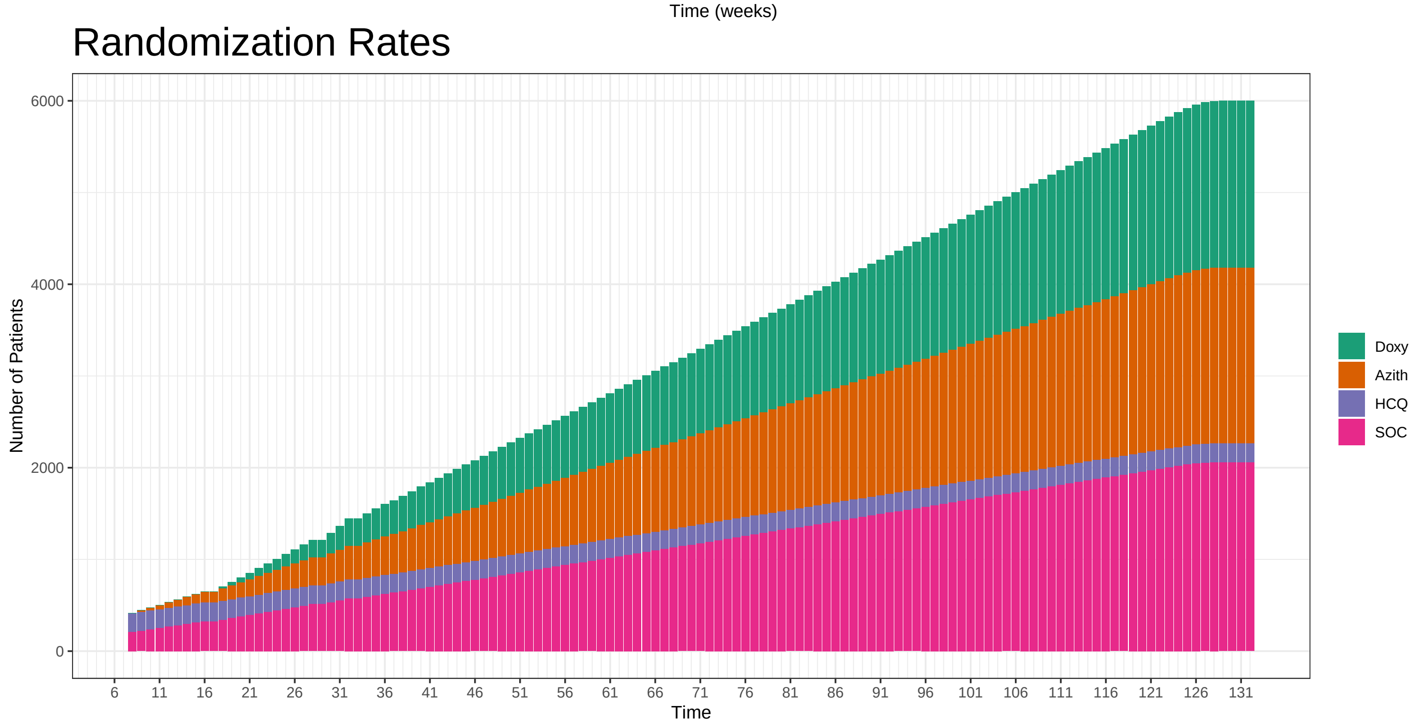

5.4 OCs Additional Scenarios: Modified Time to Recovery Rate for Usual Care Arm

Principle OCs

Berry Consultants

10/26/2020

These operating characteristics are based on 1000 simulations per scenario across a variety of assumed treatment effects for experimental arms in the trial. The true number of patients randomized per day from April 1, 2020 to October 19, 2020. After this period is over, the simulations assume a 50 patient per week accrual rate. In absence of a definitive rule, these simulations conduct an interim every 28 days. In addition, the true day of enrollment stopping of HCQ is used as well as the days for opening enrollment to the Azithromycin and Doxycycline arms. As such, up to at least October 19, 2020 (day 199) of the trial, the enrollment rates to each arm in these simulations are expected to align closely with the true enrollment rates to the arms in the trial.

Median TTR	Hazard Ratio			Power for TTR				Hospitalization Rate Benefit (%)			Power for TTR & Hosp		
SOC	HCQ	Azith	Doxy	SOC	HCQ	Azith	Doxy	HCQ	Azith	Doxy	HCQ	Azith	Doxy
7	1	1	1.0	0.979	0.009	0.012	0.010	0	0	0	0	0	0.000
7	1	1	1.1	0.768	0.009	0.019	0.221	0	0	1	0	0	0.033
7	1	1	1.2	0.317	0.011	0.010	0.683	0	0	2	0	0	0.343
7	1	1	1.3	0.059	0.007	0.007	0.941	0	0	3	0	0	0.905
11	1	1	1.0	0.940	0.017	0.036	0.030	0	0	0	0	0	0.000
11	1	1	1.1	0.467	0.015	0.038	0.522	0	0	1	0	0	0.105
11	1	1	1.2	0.057	0.015	0.014	0.943	0	0	2	0	0	0.578
11	1	1	1.3	0.003	0.015	0.013	0.997	0	0	3	0	0	0.963

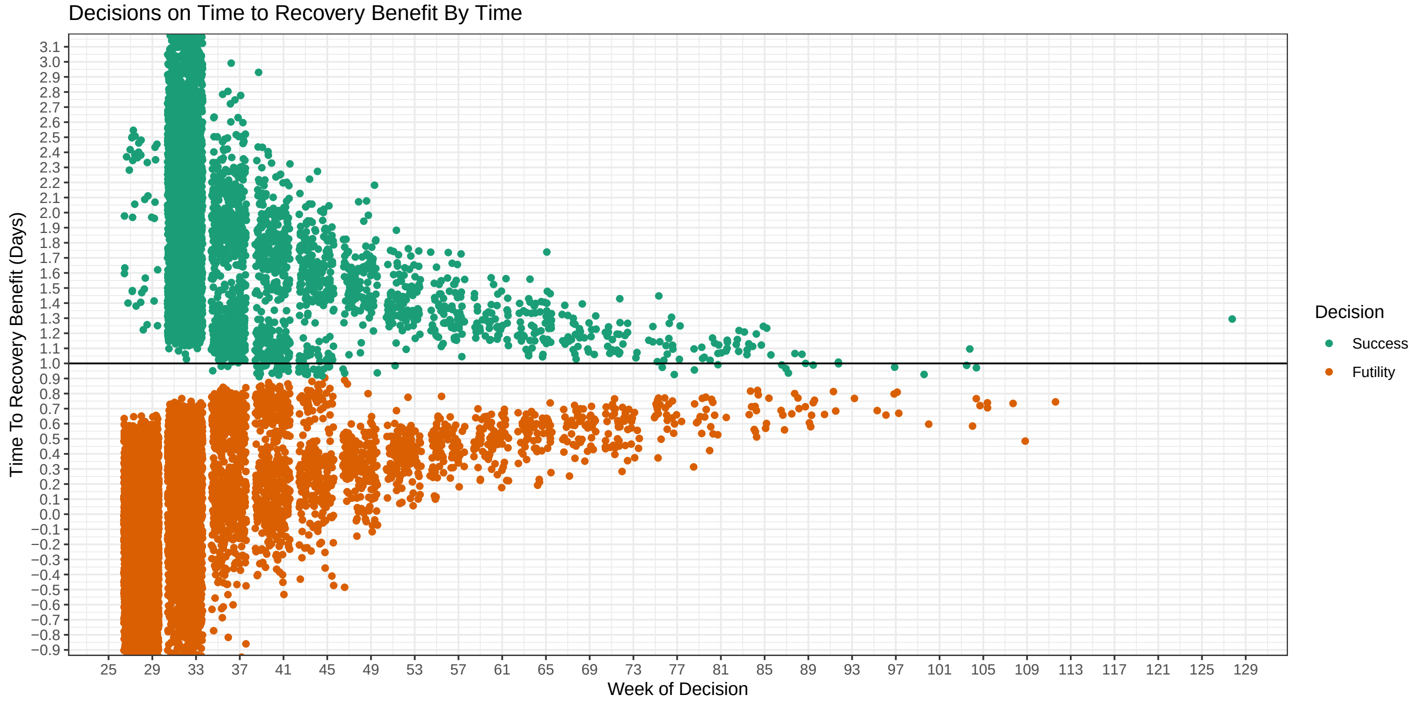

Hazard Ratios: HCQ: 1 Azith: 1 Doxy: 1

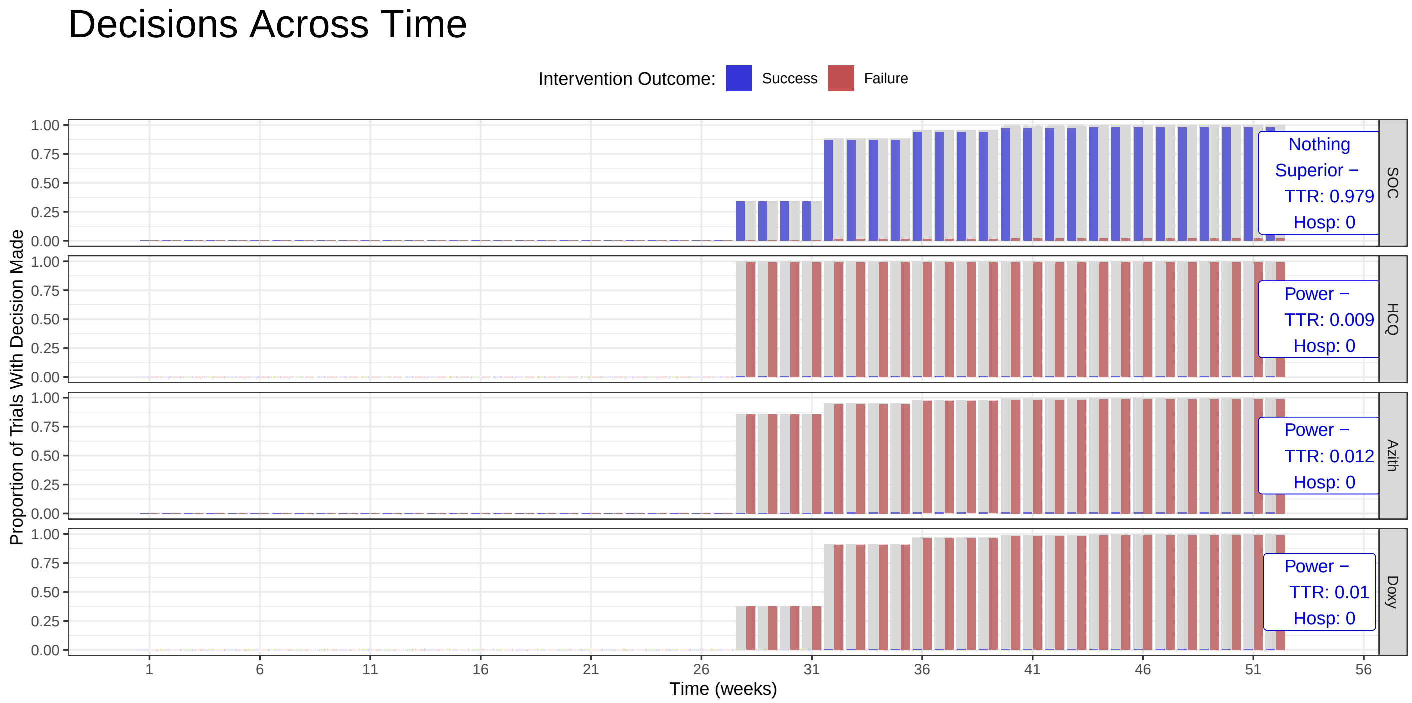

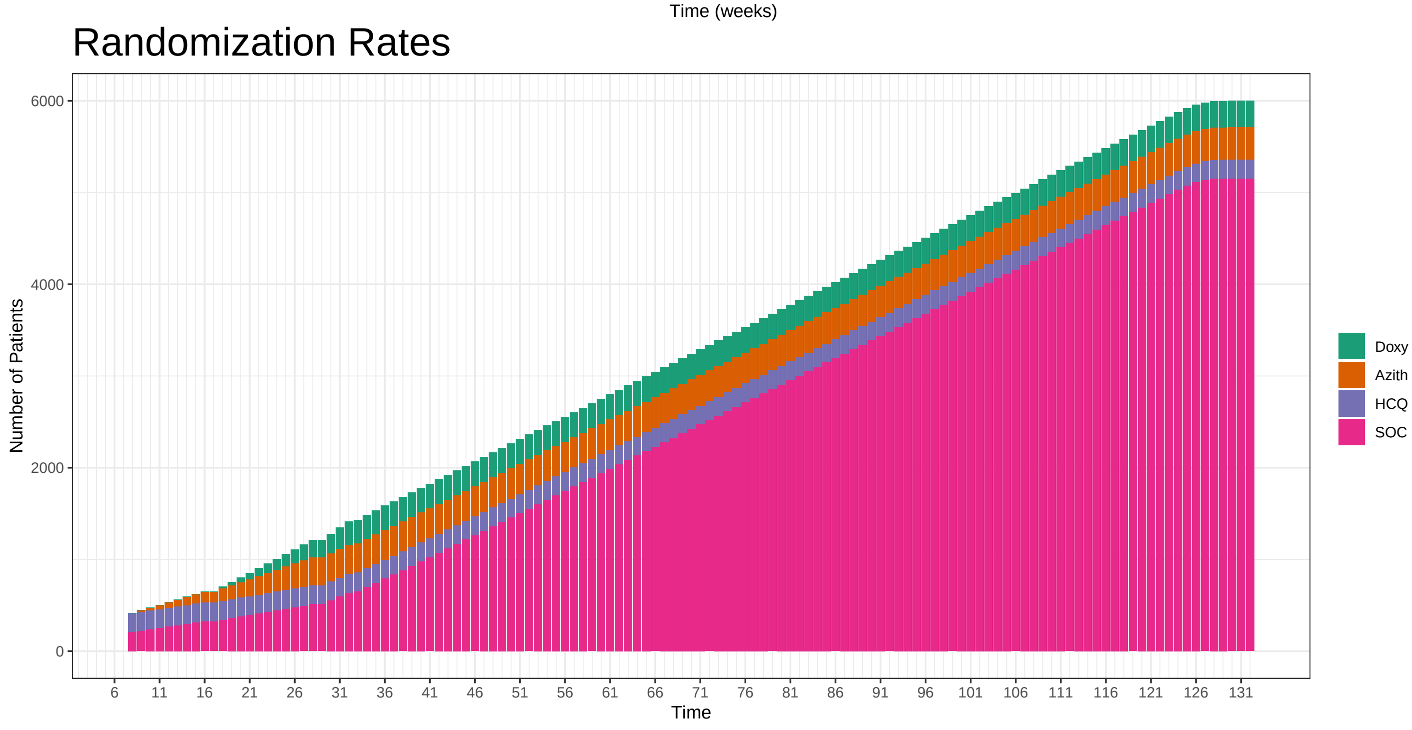

Hazard Ratios: HCQ: 1 Azith: 1 Doxy: 1.1

Decisions Across Time

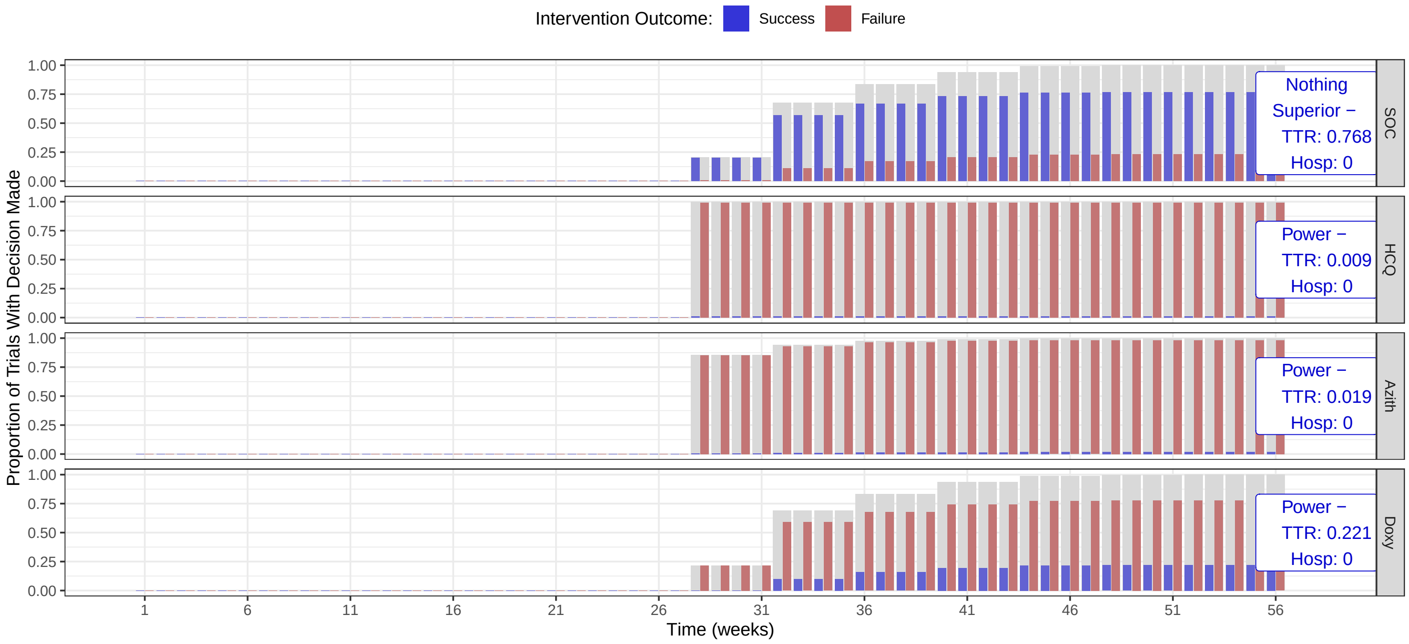

Randomization Rates

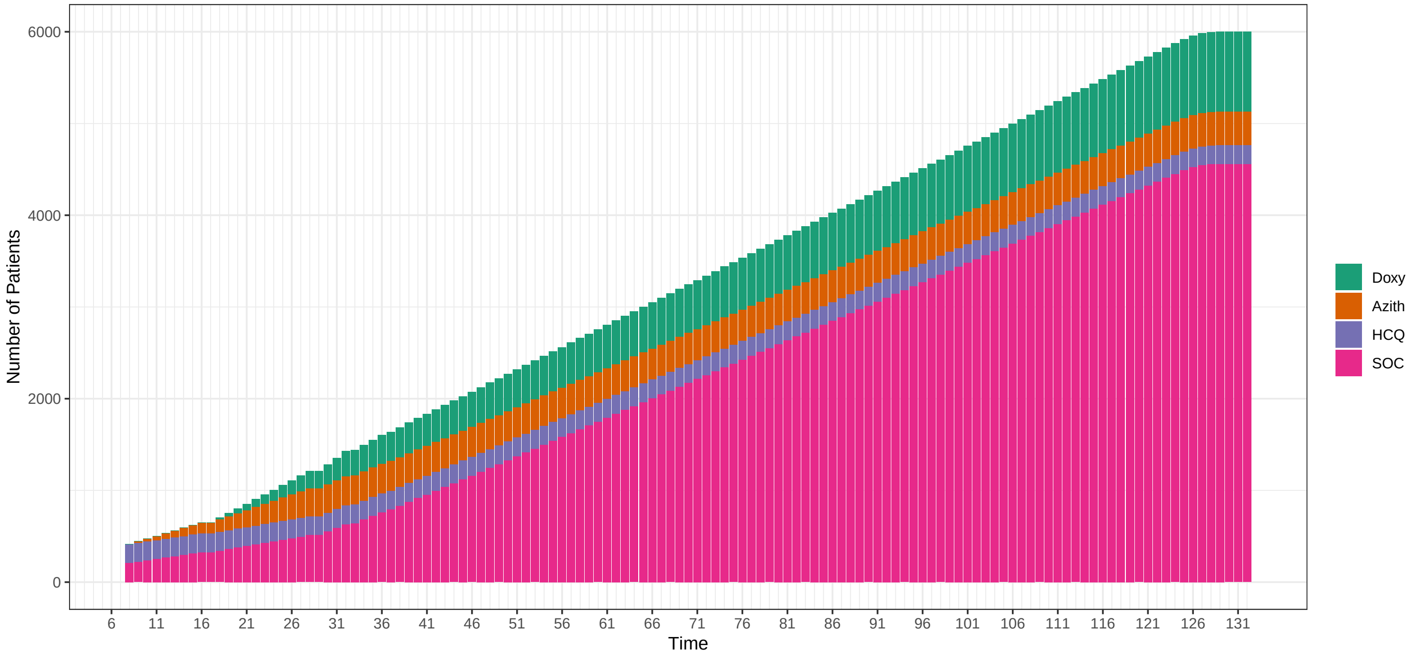

Hazard Ratios: HCQ: 1 Azith: 1 Doxy: 1.2

Decisions Across Time

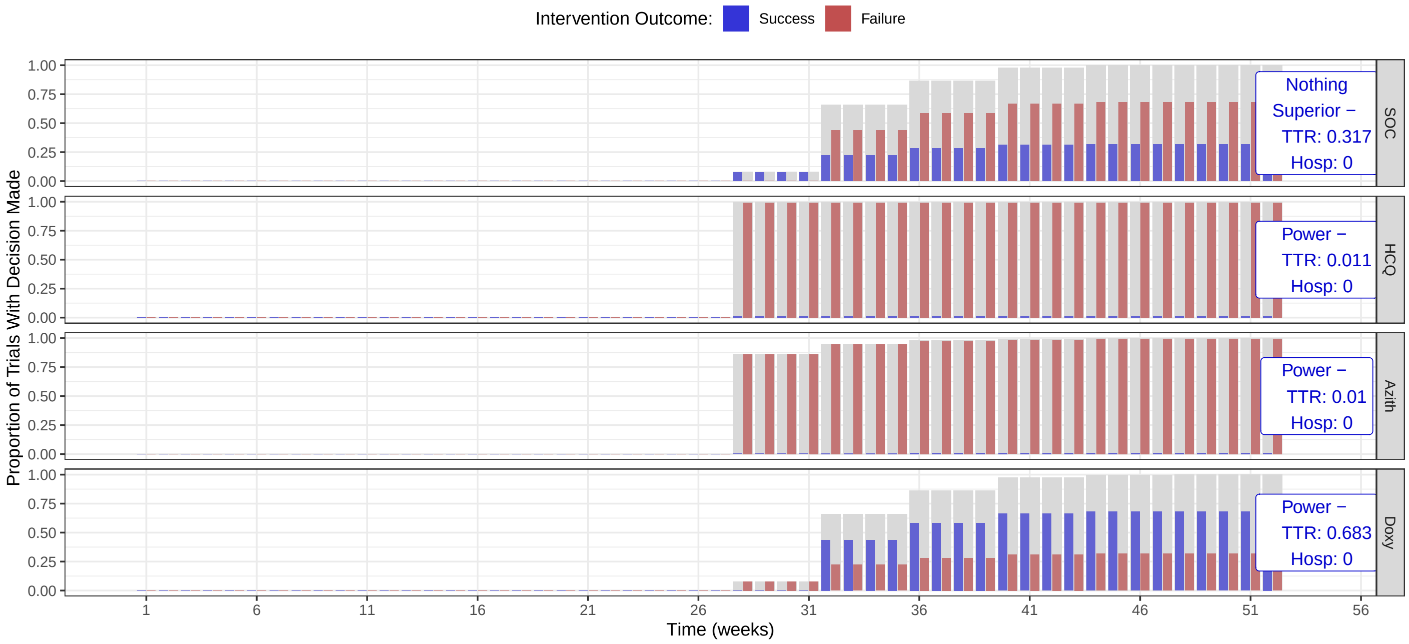

Randomization Rates

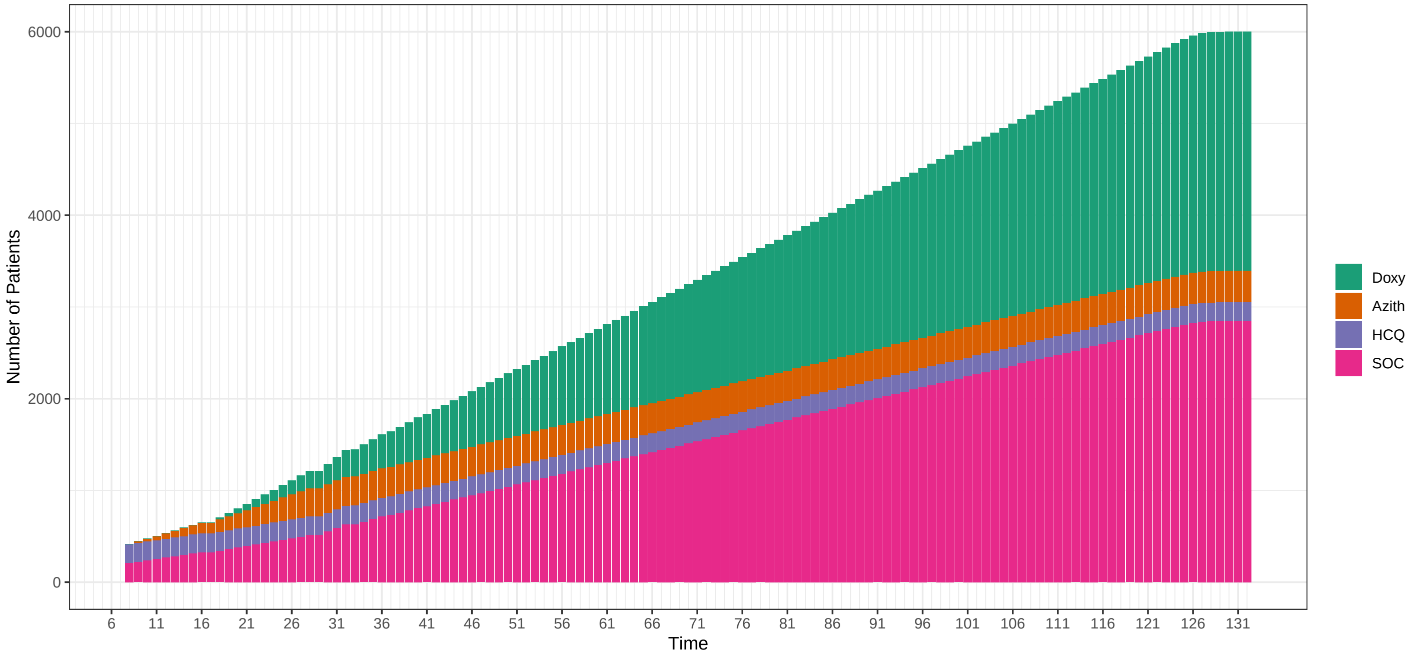

Hazard Ratios: HCQ: 1 Azith: 1 Doxy: 1.3

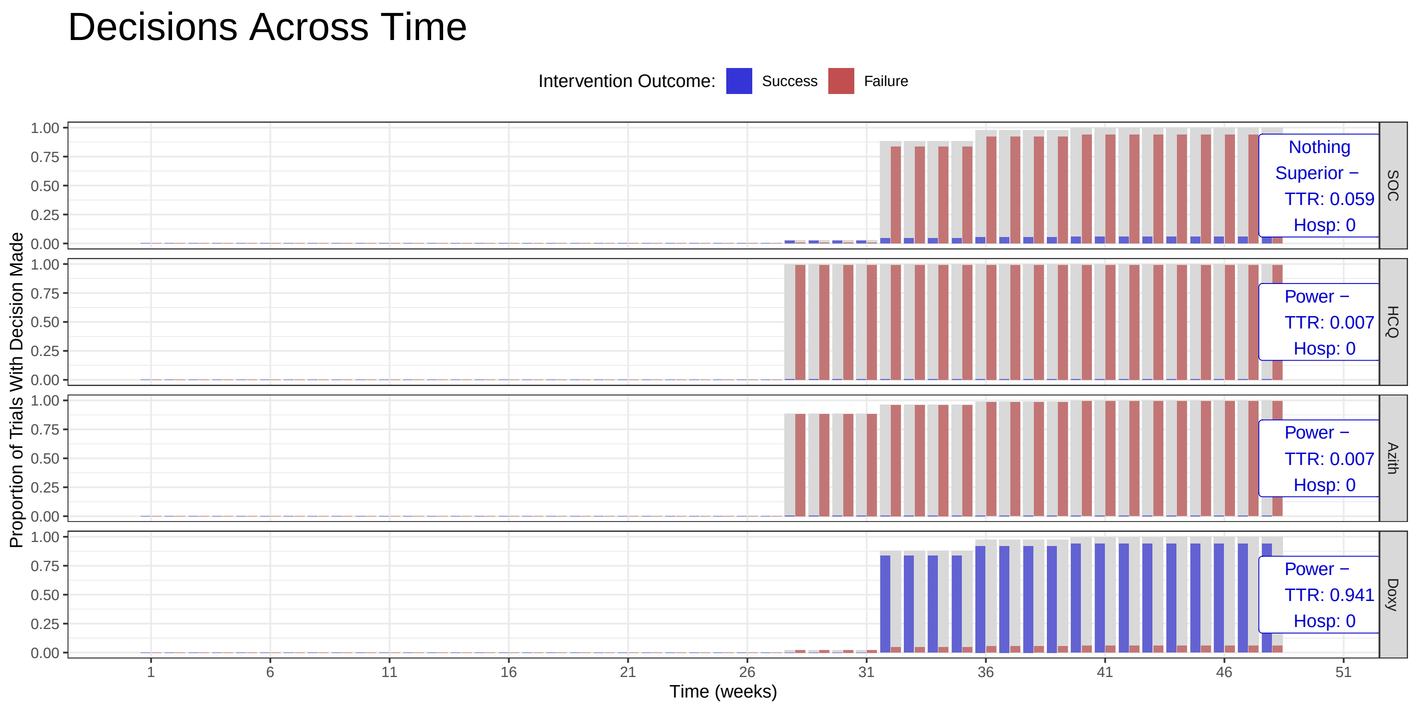

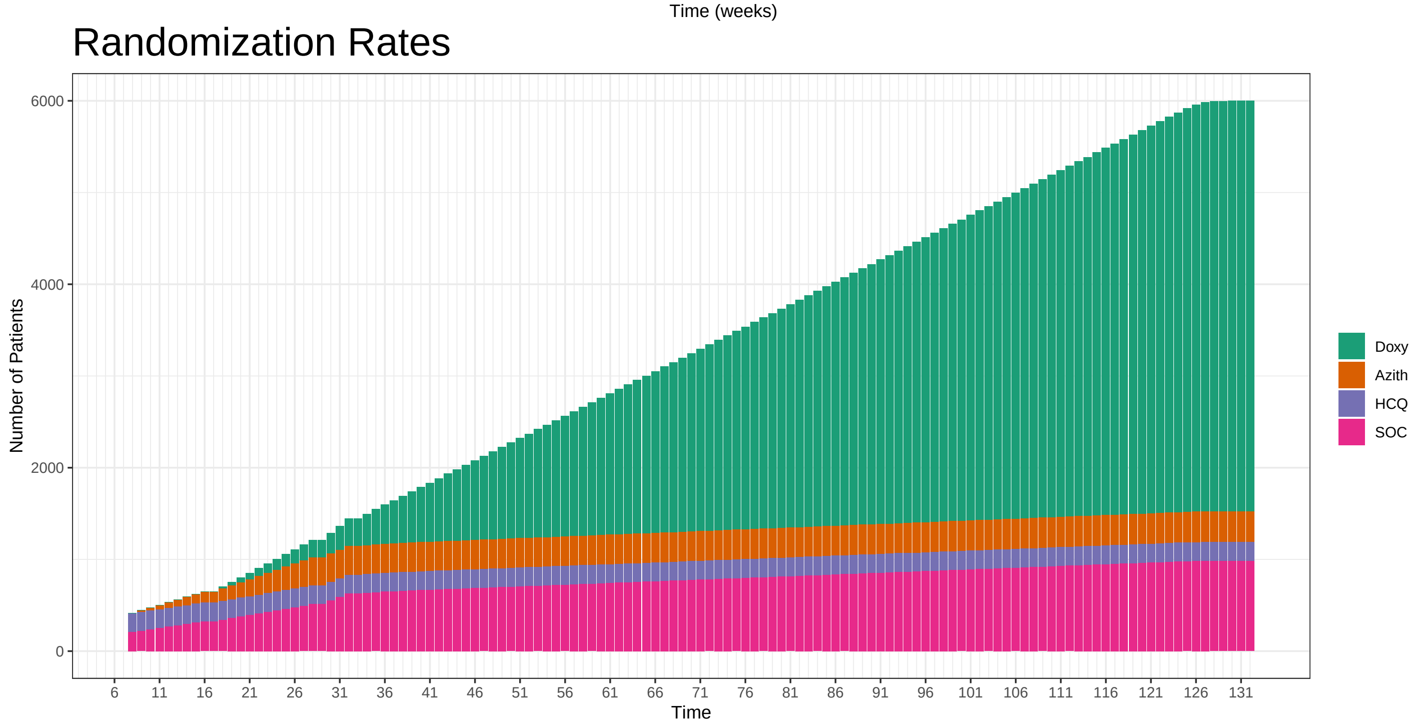

Hazard Ratios: HCQ: 1 Azith: 1 Doxy: 1

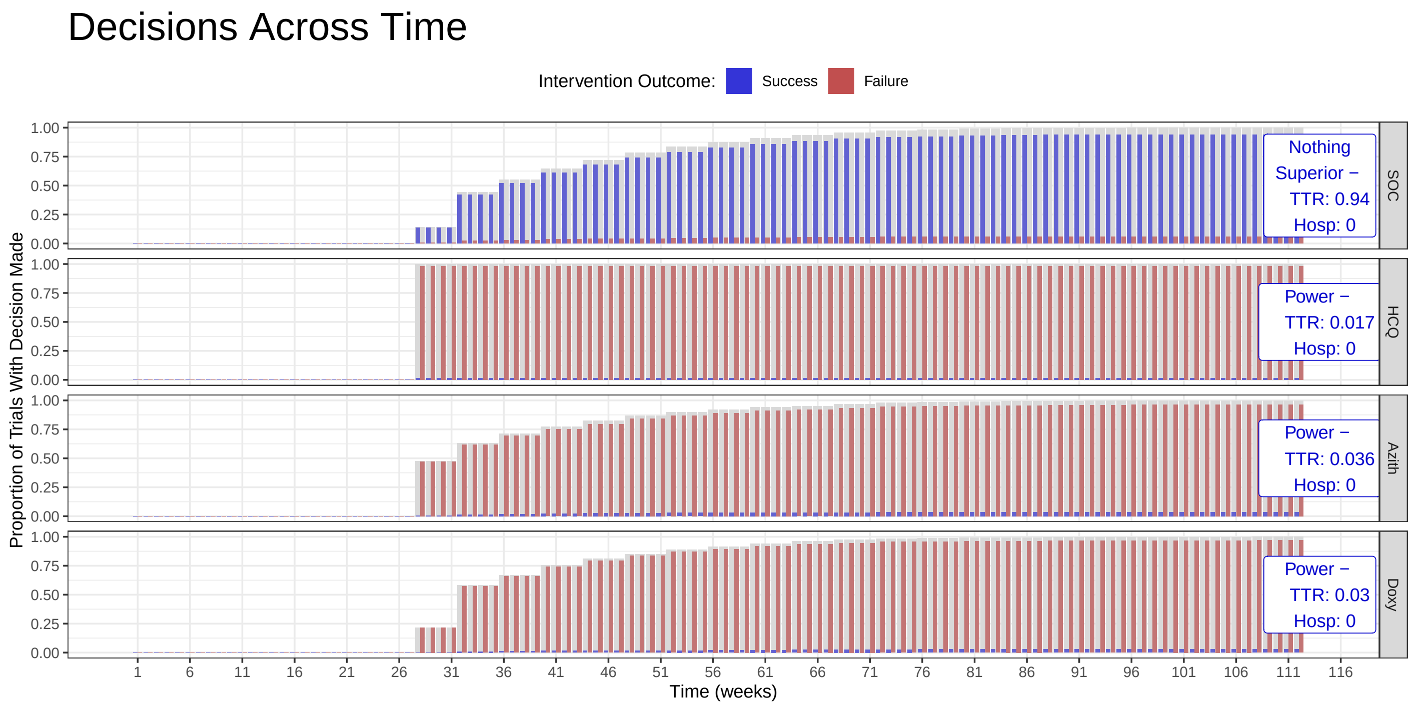

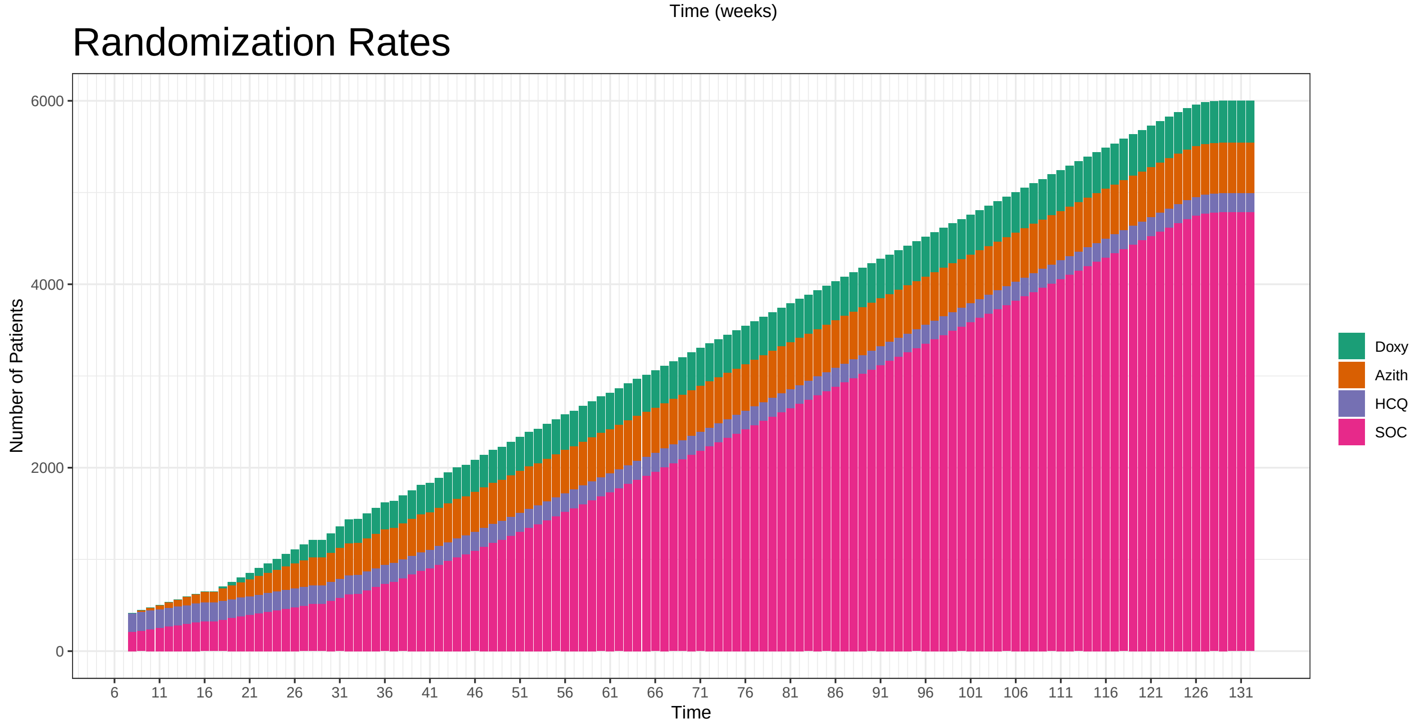

Hazard Ratios: HCQ: 1 Azith: 1 Doxy: 1.1

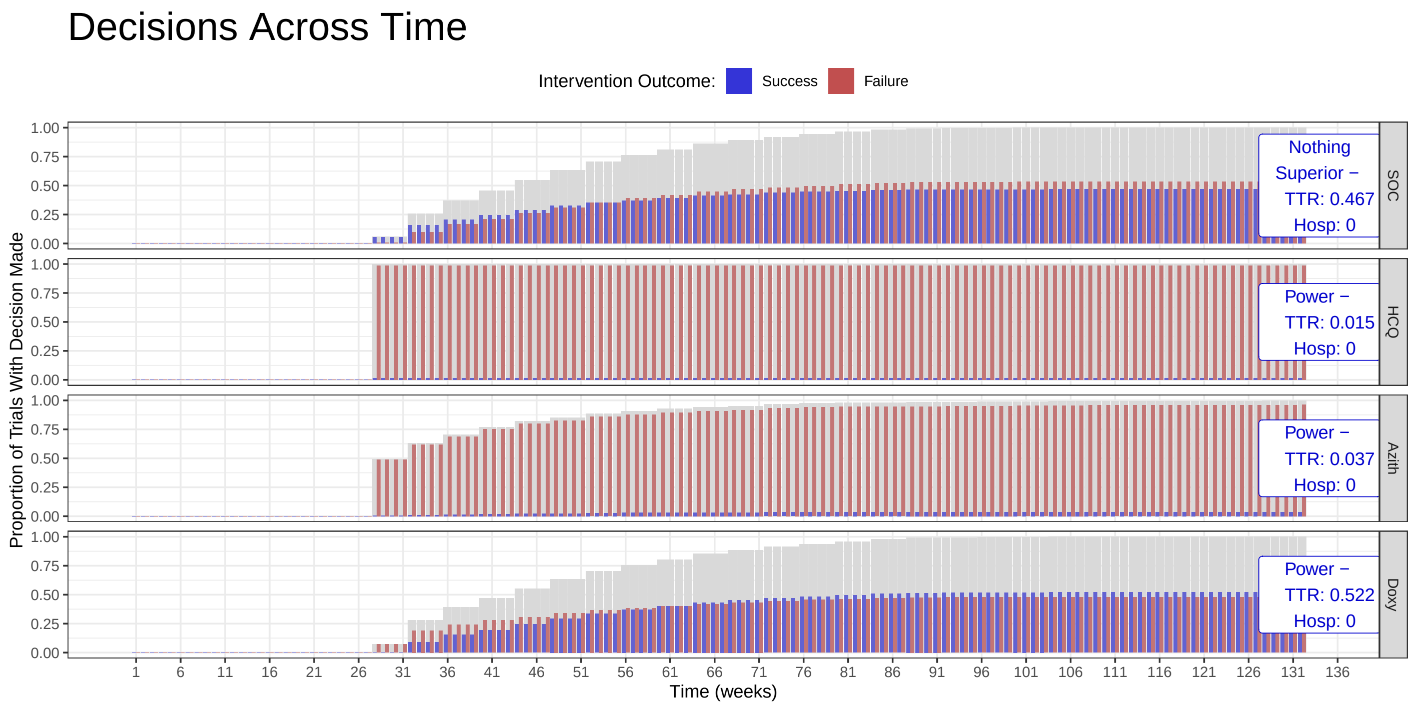

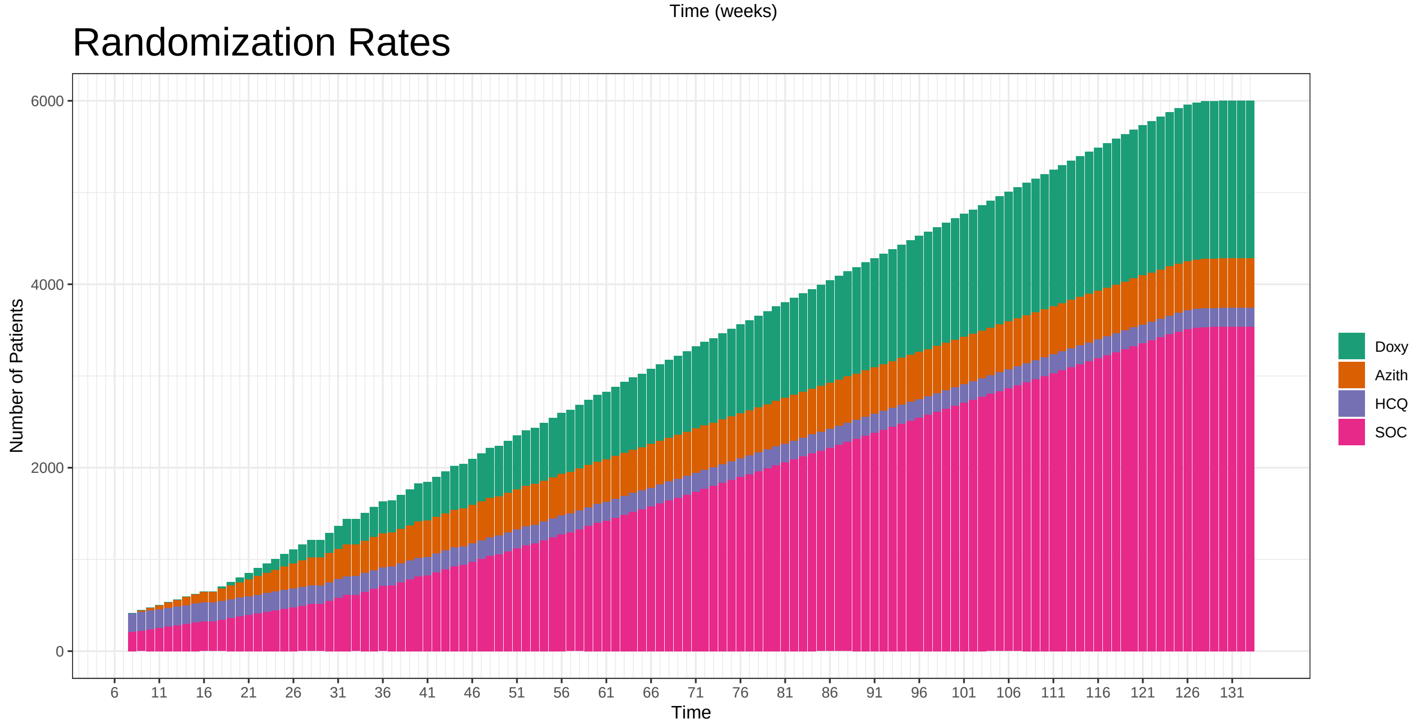

Hazard Ratios: HCQ: 1 Azith: 1 Doxy: 1.2

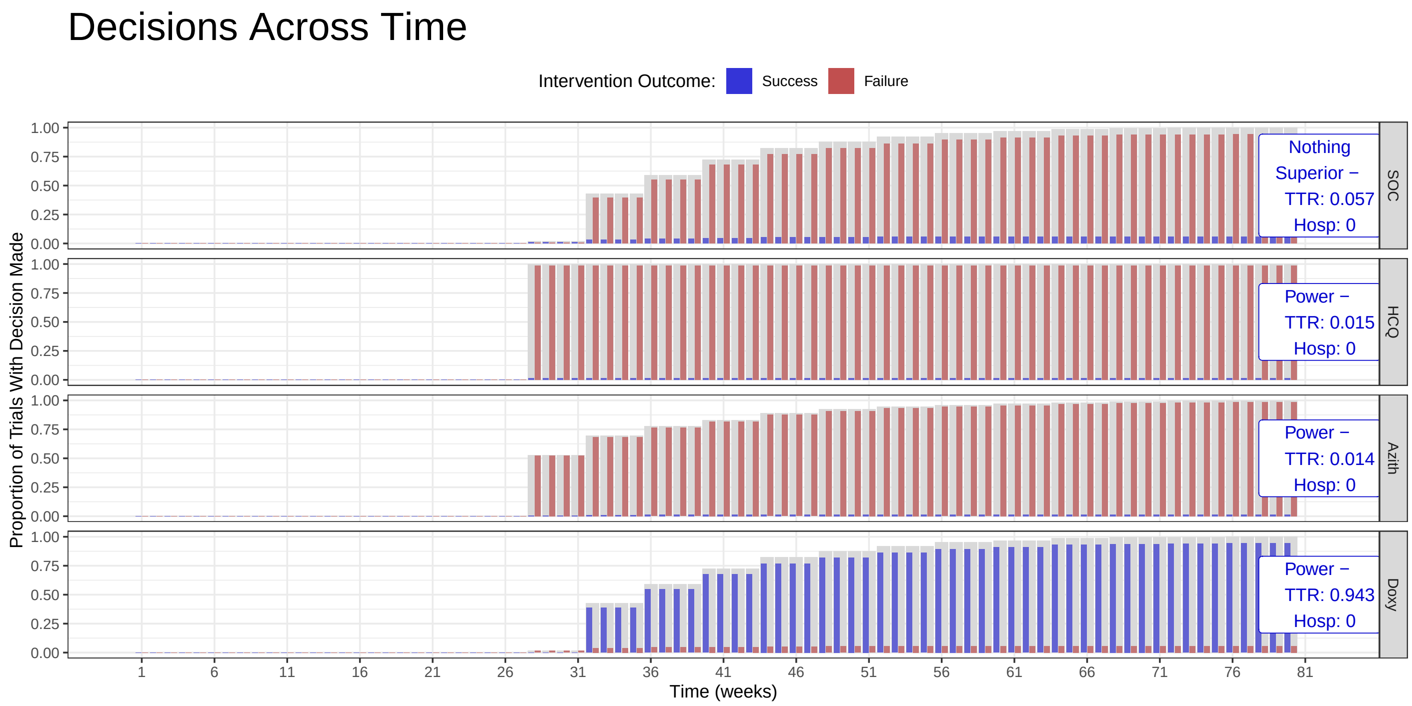

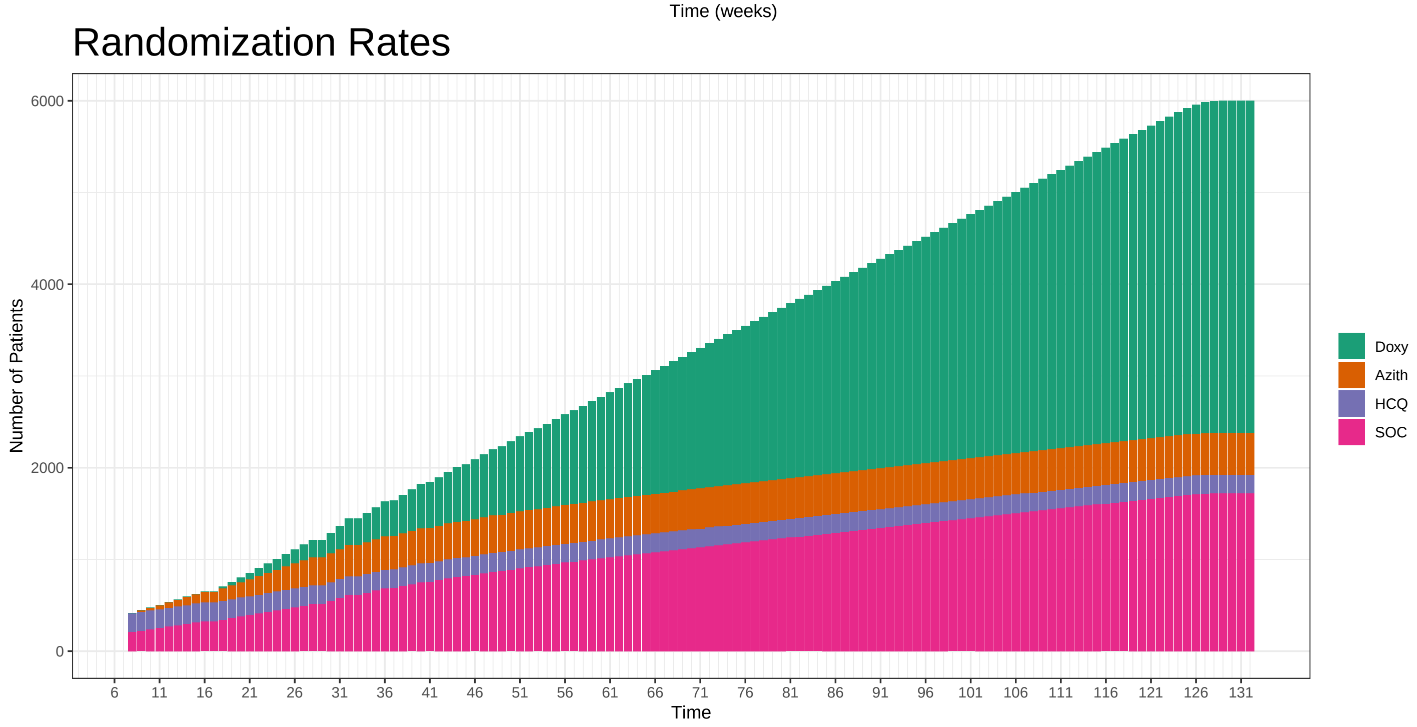

Hazard Ratios: HCQ: 1 Azith: 1 Doxy: 1.3

Decisions Across Time

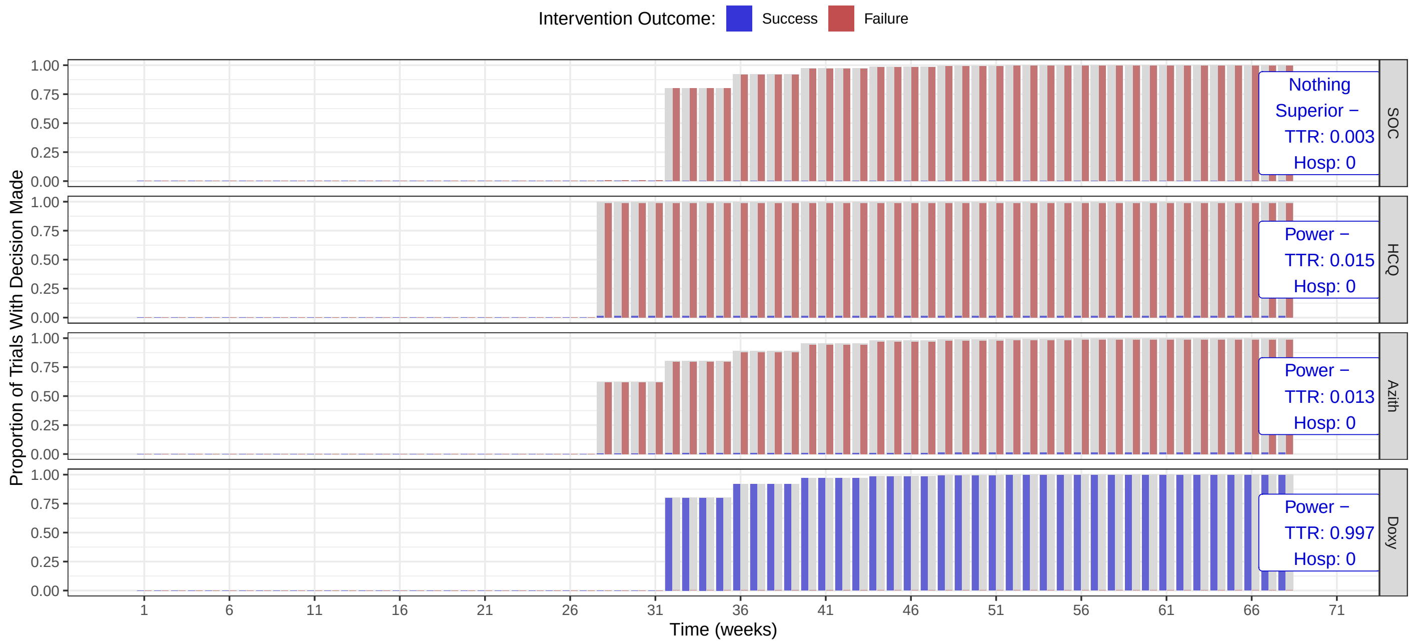

Randomization Rates

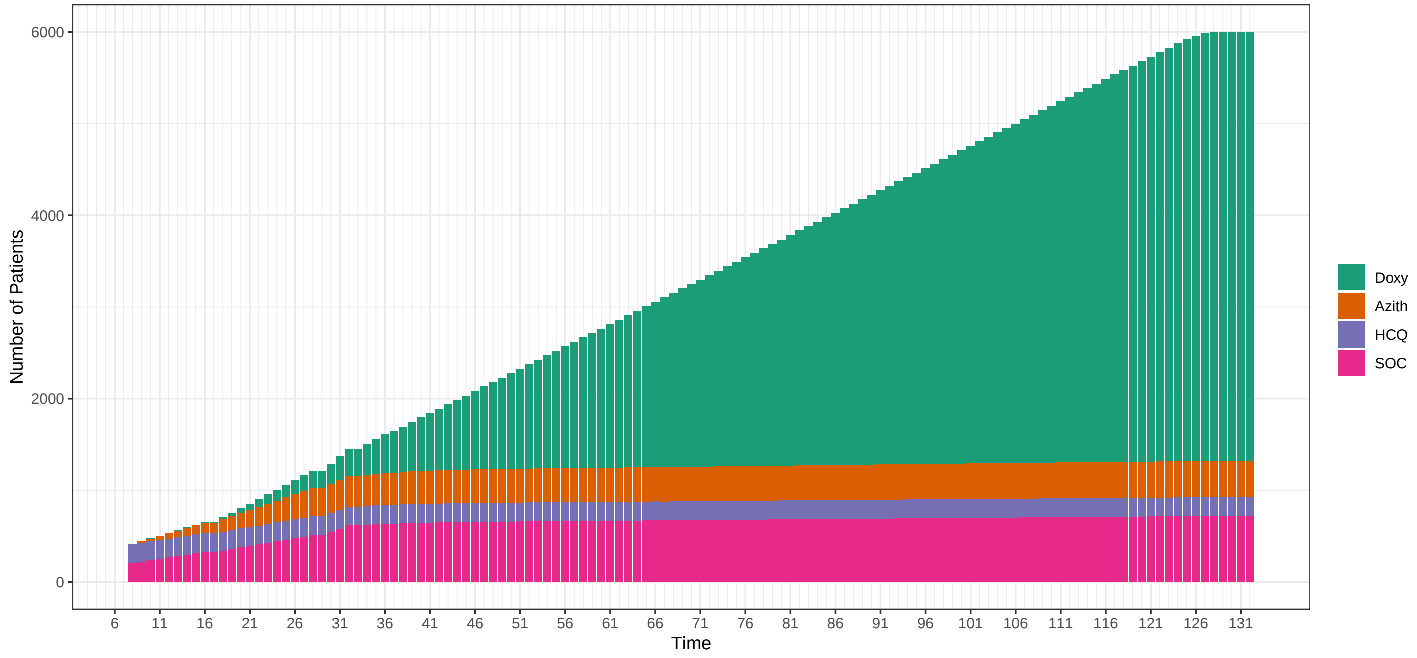

5.5 OCs Additional Scenarios: Fast Accrual

Principle OCs

Berry Consultants

10/26/2020

These operating characteristics are based on 1000 simulations per scenario across a variety of assumed treatment effects for experimental arms in the trial. The true number of patients randomized per day from April 1, 2020 to October 19, 2020. After this period is over, the simulations assume a faster than expected 100 patient per week accrual rate. In absence of a definitive rule, these simulations conduct an interim every 28 days. In addition, the true day of enrollment stopping of HCQ is used as well as the days for opening enrollment to the Azithromycin and Doxycycline arms. As such, up to at least October 19, 2020 (day 199) of the trial, the enrollment rates to each arm in these simulations are expected to align closely with the true enrollment rates to the arms in the trial.

Median TTR	Hazard Ratio			Power for TTR				Hospitalization Rate Benefit (%)			Power for TTR & Hosp		
SOC	HCQ	Azith	Doxy	SOC	HCQ	Azith	Doxy	HCQ	Azith	Doxy	HCQ	Azith	Doxy
9	1	1	1.0	0.962	0.014	0.023	0.016	0	0	0	0	0	0.000
9	1	1	1.1	0.613	0.015	0.020	0.378	0	0	1	0	0	0.067
9	1	1	1.2	0.120	0.015	0.016	0.880	0	0	2	0	0	0.588
9	1	1	1.3	0.011	0.014	0.009	0.989	0	0	3	0	0	0.975

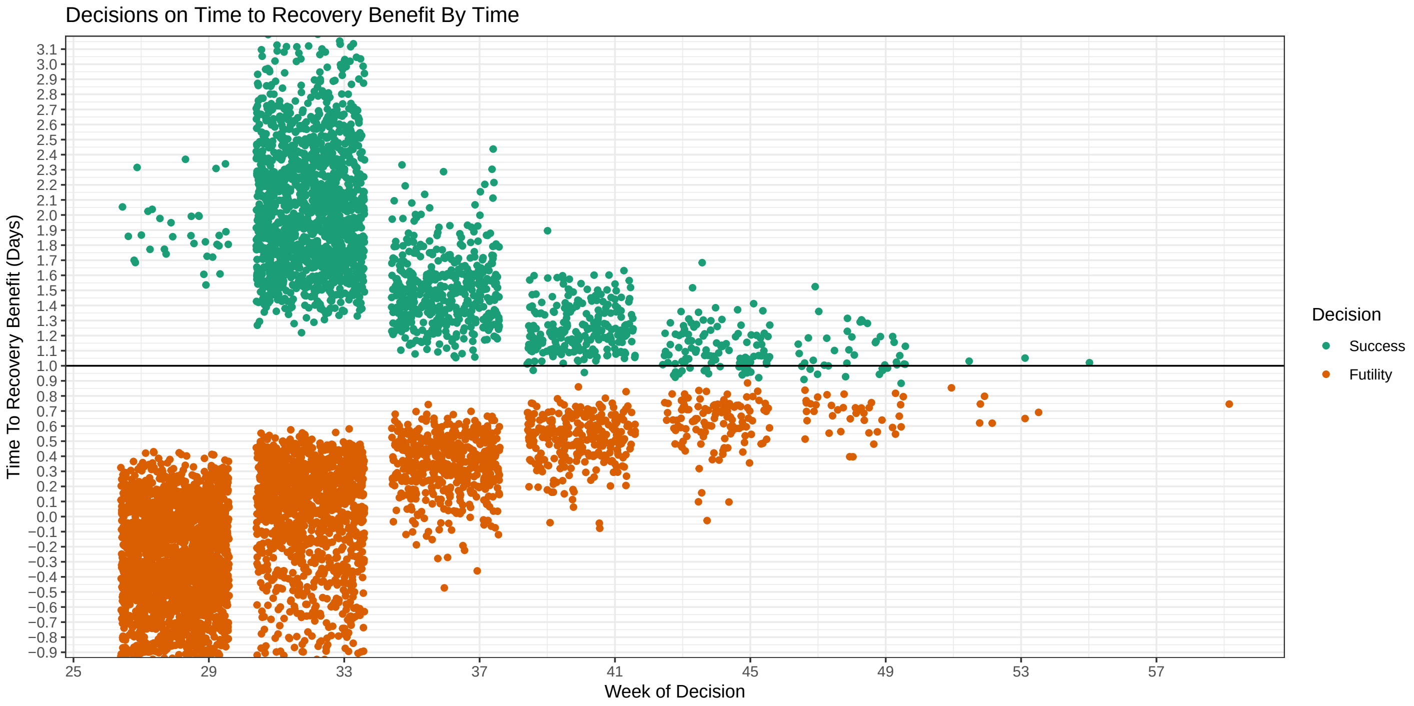

Decisions on Time to Recovery Benefit By Time
Time To Recovery Benefit (Days)
Week of Decision
25
29
33
37
41
45
49
53
57
Decision
Success
Futility

Hazard Ratios: HCQ: NA Azith: NA Doxy: NA

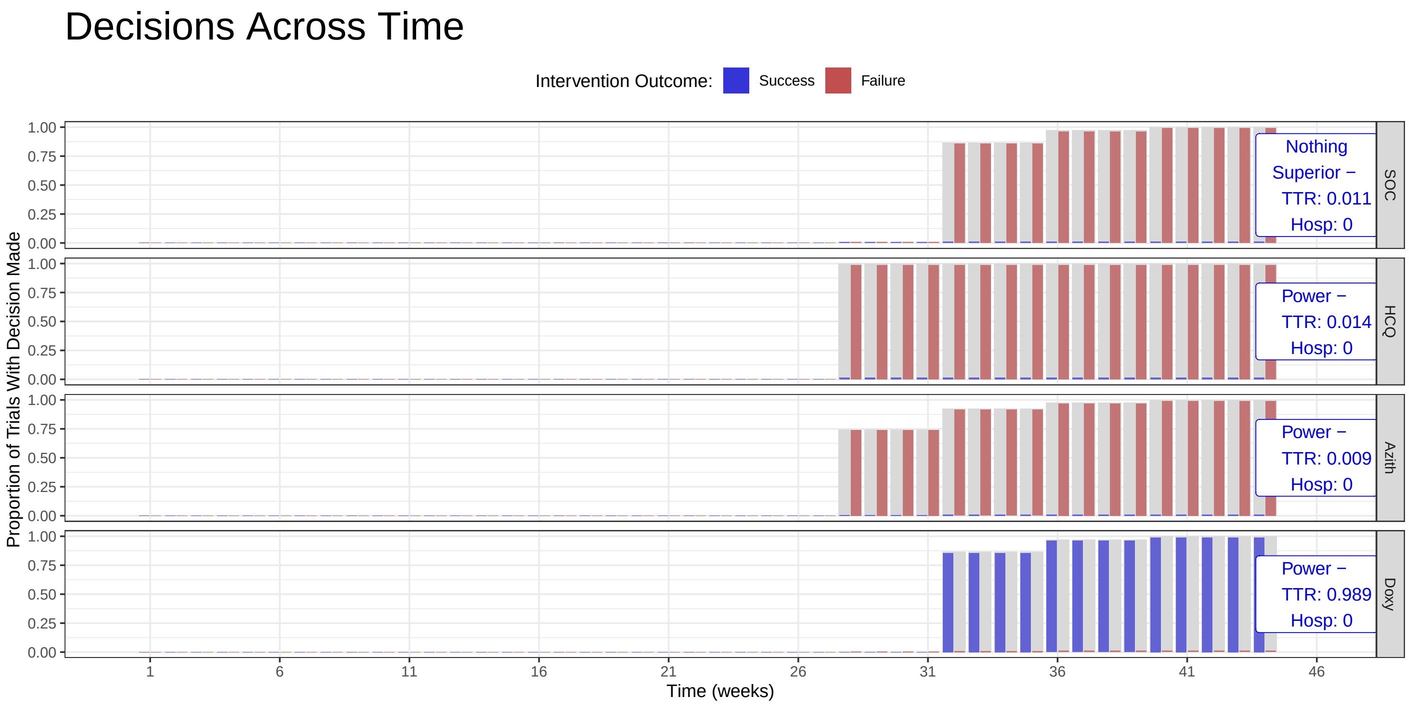

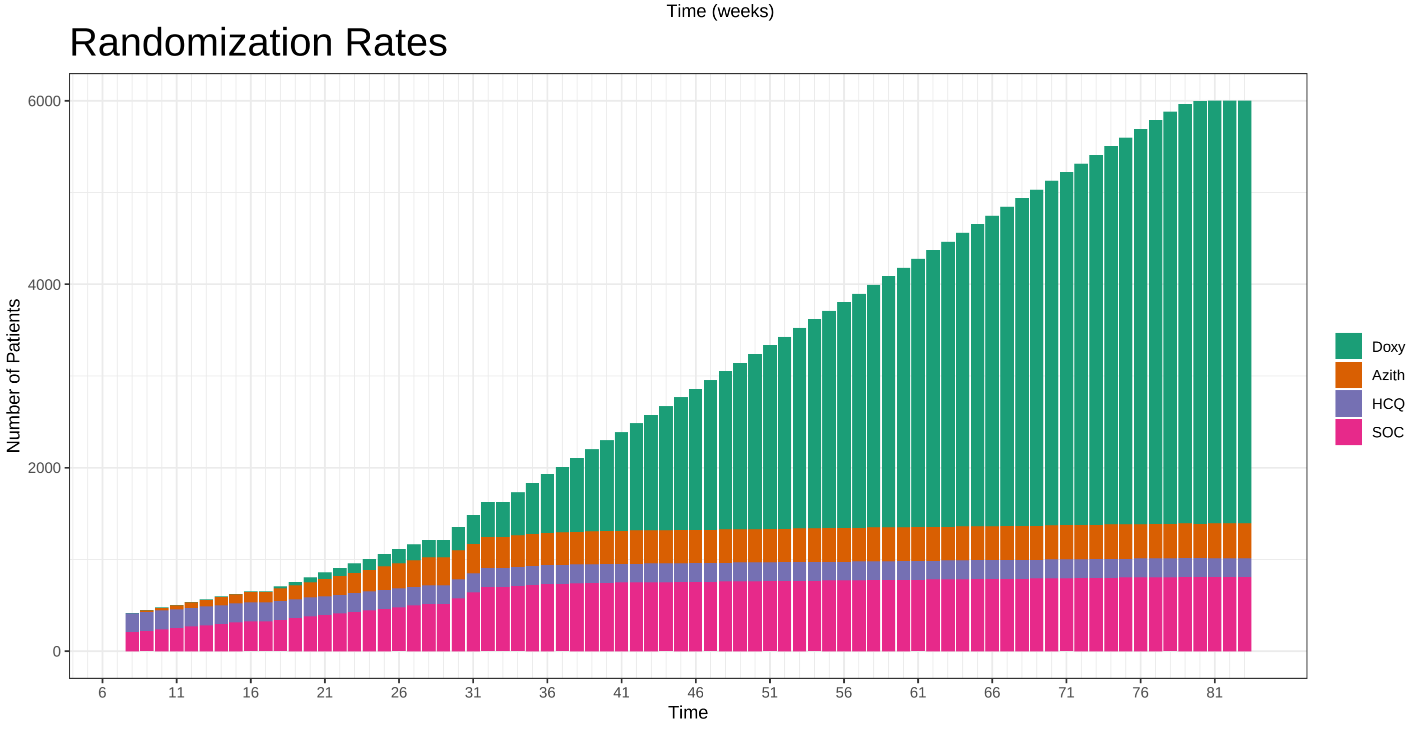

Hazard Ratios: HCQ: NA Azith: NA Doxy: NA

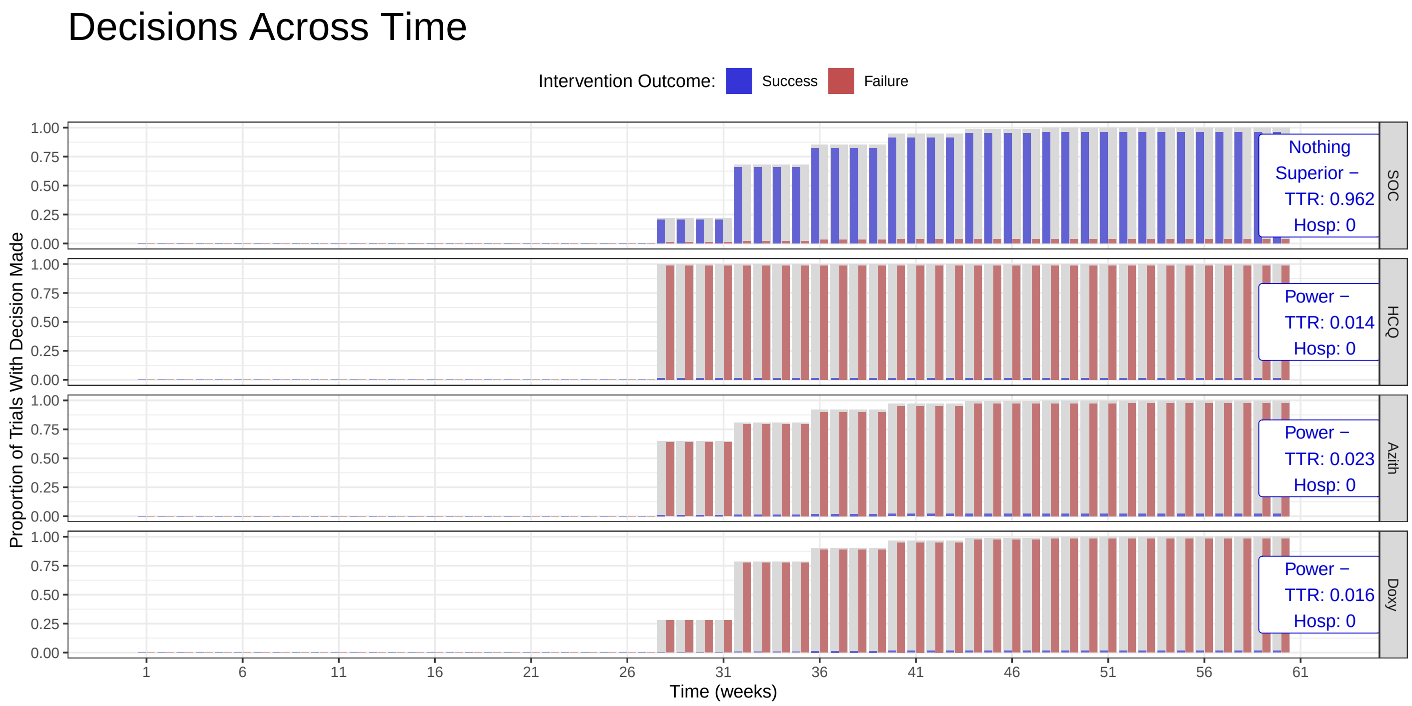

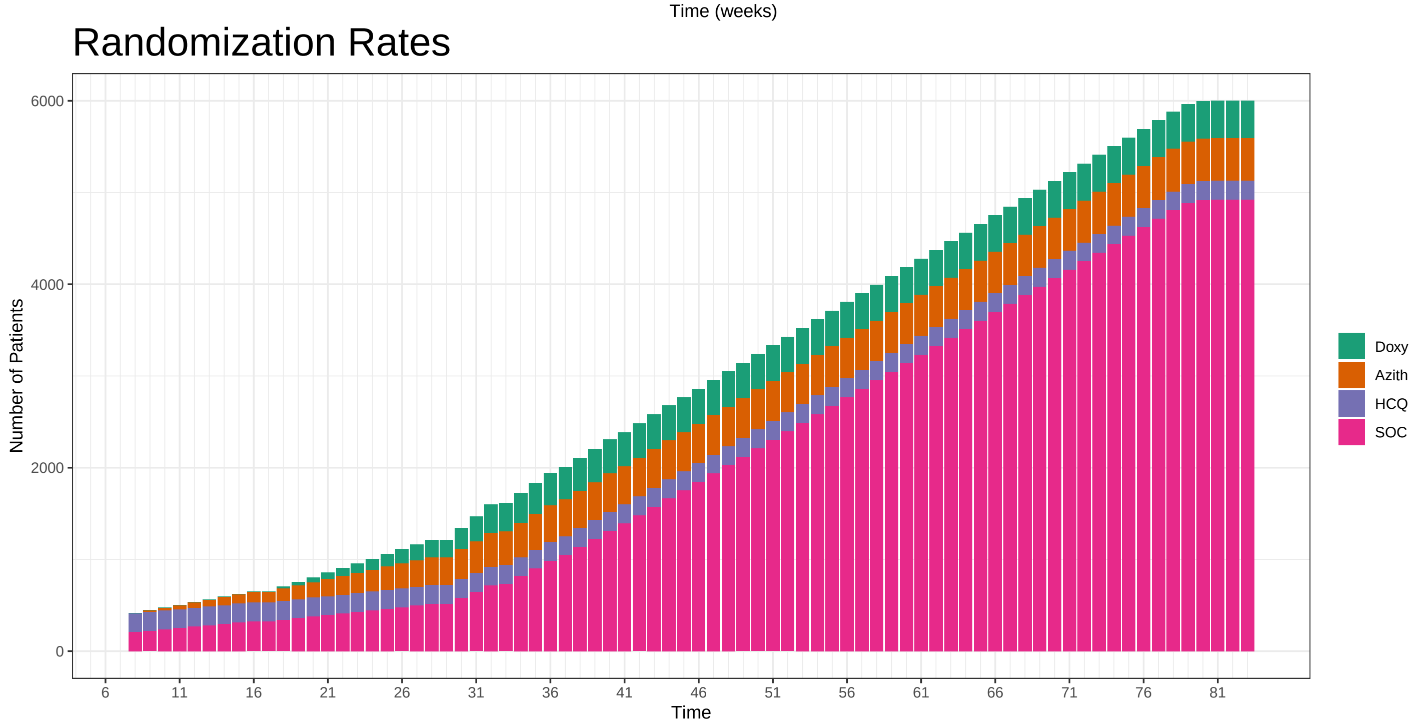

Hazard Ratios: HCQ: NA Azith: NA Doxy: NA

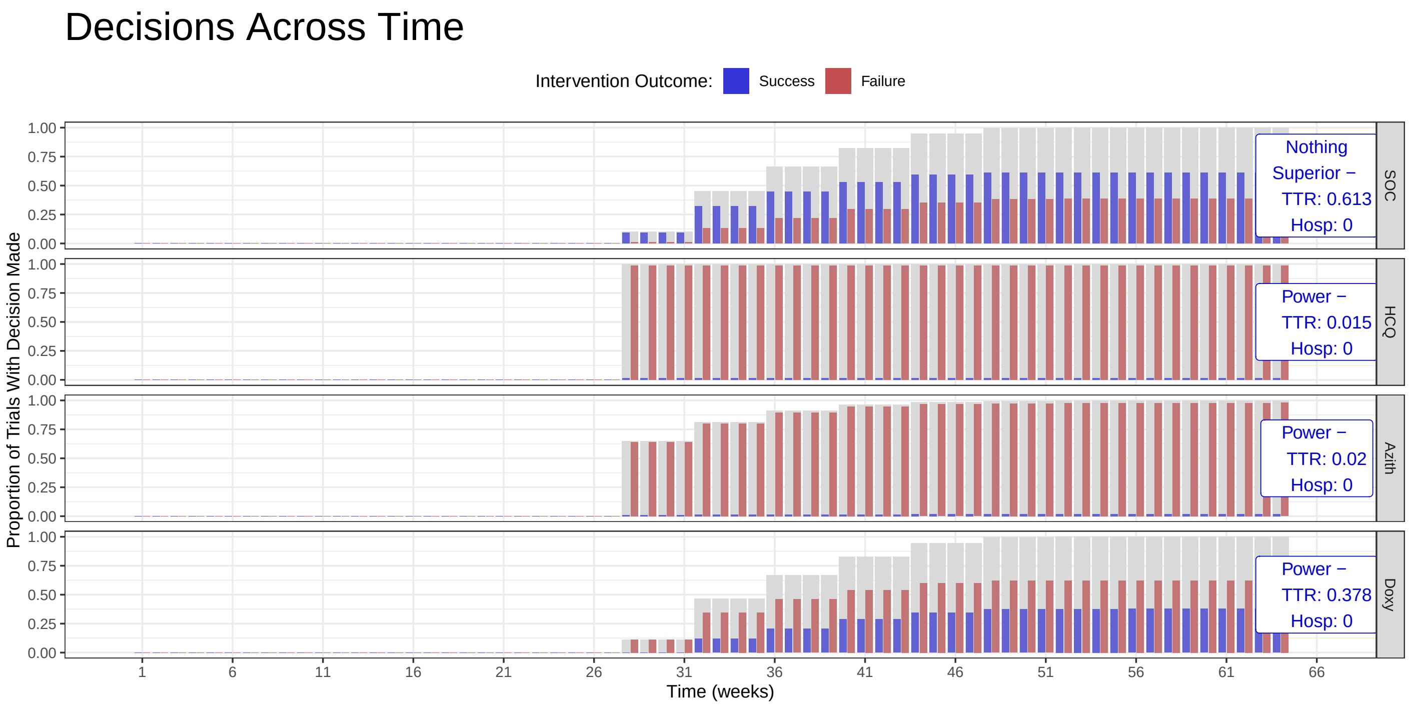

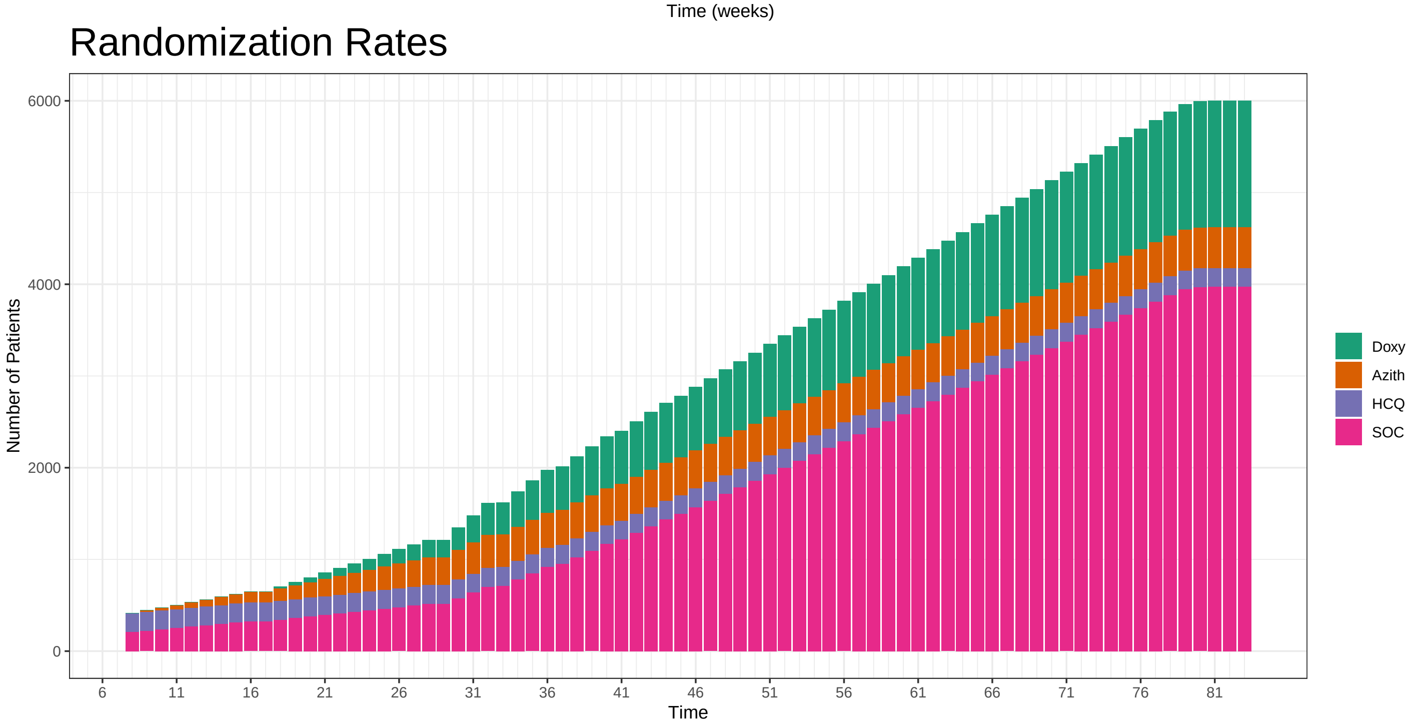

Hazard Ratios: HCQ: NA Azith: NA Doxy: NA

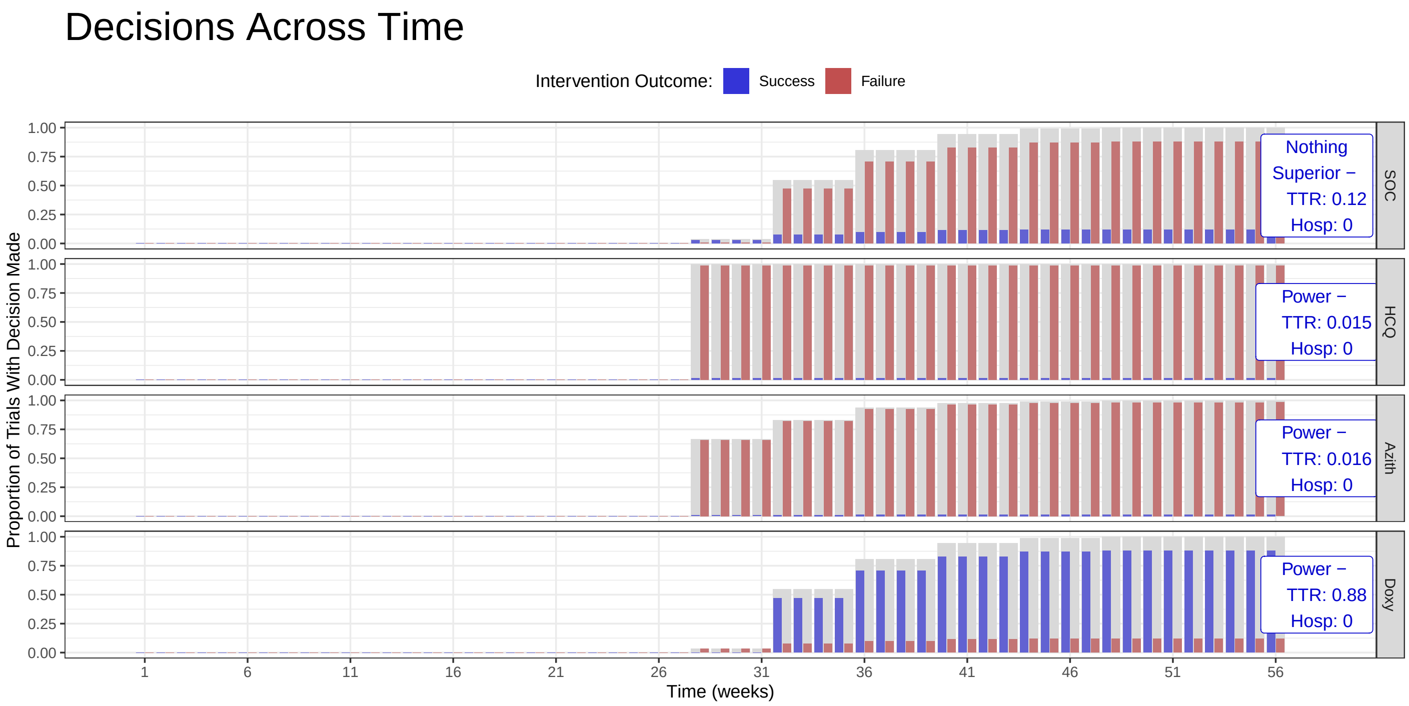

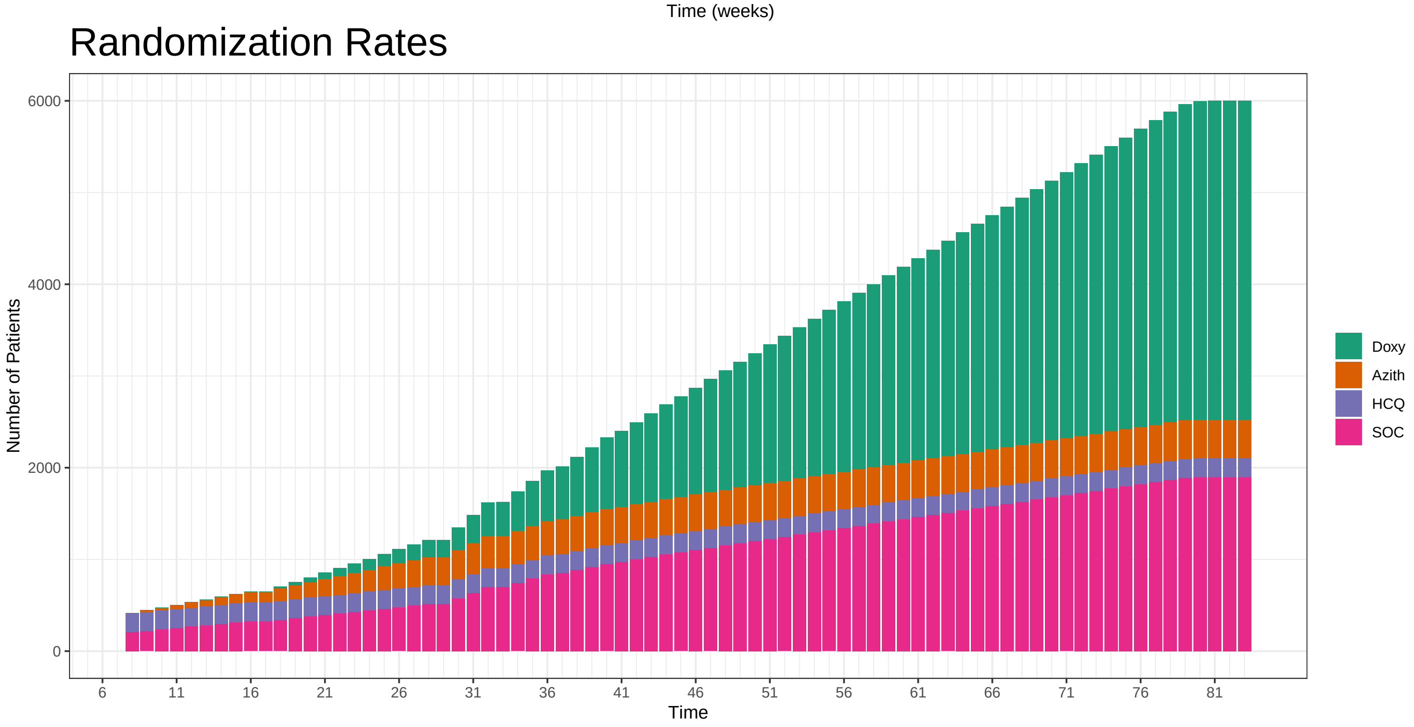

6 Simulated Example Trials

6.1 Simulated Example Trials: Three Interventions

This section explores the virtual simulations for the first three interventions versus Usual Care, with staggered entry (and HCQ suspension) as observed in the trial. We simulate virtual example trials and apply the adaptive algorithm exactly as described in the preceding sections. At each interim analysis, we perform an analysis of the data available at the time of the interim. For the purposes of the simulations, each of the trials continues to 4,000 participants in the primary analysis population, but in reality the end of the platform trial will be determined by available resources and the state of the pandemic. The calculations and statistical inferences using these data are summarized in an interim dashboard. The interim number and timing of the interim are provided in the upper left hand corner of the dashboard.

The table provides quantities calculated from the raw data as well as model estimated quantities. The columns denote the following.

Recovery Data

- Enrolled: The number of participants randomized to each arm at the time of the interim analysis
- Complete: The number of randomized participants with the opportunity to complete 28-days of follow-up
- Recovered: The number of randomized participants recovered, per primary endpoint definition (i.e. first recovery)
- Exposure Days: The total number of days of observed follow-up prior to a subjects's reported recovery across all participants
- Recoveries Per Day: The number of recoveries divided by the number of exposure days, interpreted as the number of recoveries per day per participant
- Estimated Hazard: The probability of recovery on day d given no recovery up to day d, with 95% Bayesian credible interval, averaged across all time segments
- Estimated HR: The estimated hazard ratio comparing the hazard of recovery for intervention j versus the Usual Care, with 95% Bayesian credible interval
- Estimated Median Time to Recovery: The model-based estimated median time to recovery for each treatment arm

Hospitalization Data:

- Hospitalizations: Total number of participants with hospitalization or death
- 28 Day Completers: Number of participants with opportunity to complete 28 days of follow-up
- Observed Hosp. Rate: Proportion of participants with hospitalization or death among those with the opportunity to complete 28 days of follow-up
- Est. Hosp. Rate: Model-based estimate for probability of hospitalization with corresponding 95% credible interval

Recovery Inferences:

- Pr(Superiority): The model-based estimated probability of superiority with respect to recovery endpoint for intervention j versus Usual Care
- Pr(Meaningful Effect): The model-based estimated probability that the benefit in median time to recovery for intervention j compared to Usual Care is at least 1.5 days
- Pr(Best): The model-based estimated probability that intervention j is superior to all other active interventions with respect to the recovery endpoint
- Randomization Probability: The randomization probabilities to be used for treatment allocation until the next interim analysis

Hospitalization Inferences:

- Pr(Superiority): The model-based estimated probability of superiority for intervention j versus Usual Care with respect to the hospitalization/death endpoint
- Pr(Meaningful Effect): The model-based estimated probability that the reduction in proportion of persons with hospitalization/death for intervention j relative to Usual Care 0.02 or greater (i.e., at least 2 percentage points)

The plot and table in the lower left corner of the dashboard provide a more detailed look at the observed data for the recovery endpoint. The plot is a Kaplan-Meier plot showing the proportion of subjects recovered for days 0-28. The table below the plot provides the number not yet recovered by 0, 7, 14, 21, and 28 days for each arm. Both of these summaries are showing observed data that contribute to the statistical modeling.

The two plots on the lower right show summaries of the statistical modeling of the recovery endpoint. The first plot shows the Bayesian posterior distributions of the hazard ratios corresponding to each intervention. Hazard ratios greater than one indicate faster recovery. The second plot (on far right) shows the Bayesian posterior distribution of the median time to recovery in days, with the center of each distribution shown in text above the peak of the distribution.

Below we show four virtual trials to illustrate the adaptive platform trial.

6.2 Example 1

April 1, 2020

Before Patient Enrollment Begins

Recovery Data

	Enrolled	Complete	Recovered	Exposure Days	Recoveries Per Day	Estimated Hazard	Estimated HR (95% interval)	Estimated Median Survival Time
SOC	0	0	0	0				
HCQ	0	0	0	0				
Azith	0	0	0	0				
Doxy	0	0	0	0				
Total	0	0	0	0				

Hospitalization Data

Hospitalizations	28 Day Completers	Observed Hosp. Rate	Est. Hosp. Rate (95% interval)
0	0		
0	0		
0	0		
0	0		
0	0		

Intervention Status
Enrolling
Enrolling
Unopened
Unopened

Observed Patient Recoveries

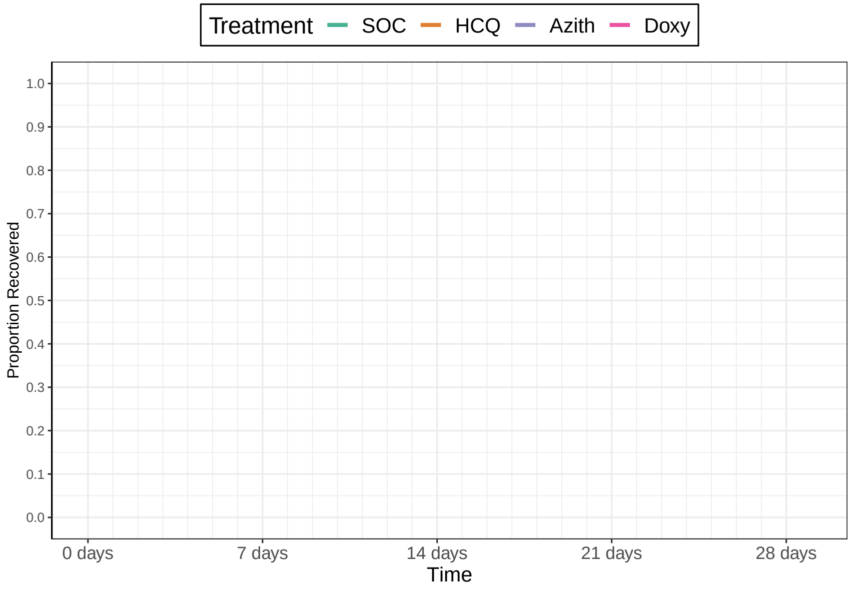

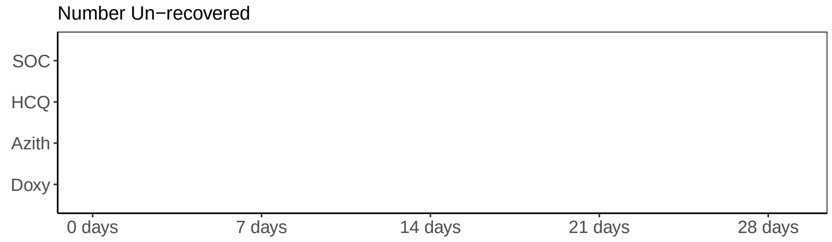

Recovery Inferences

	Pr(Superiority)	Pr(Meaningful Effect)	Pr(Best)	Randomization Probability
SOC				0.5
HCQ				0.5
Azith				0
Doxy				0

Hospitalization Inferences

Pr(Superiority)	Pr(Meaningful Effect)
0	0
0	0
0	0
0	0

Hazard Ratio Estimates

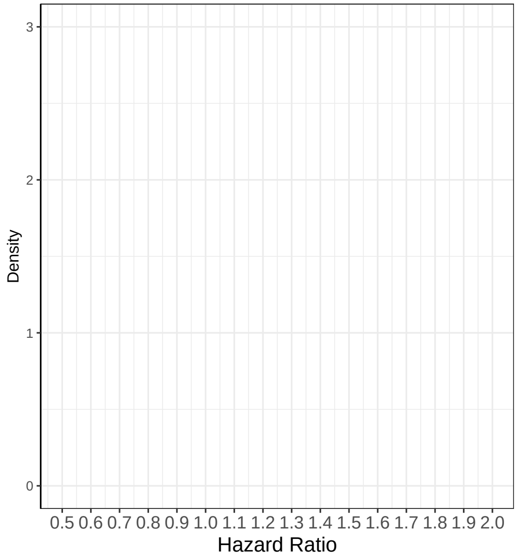

Median Time To Recovery Estimates

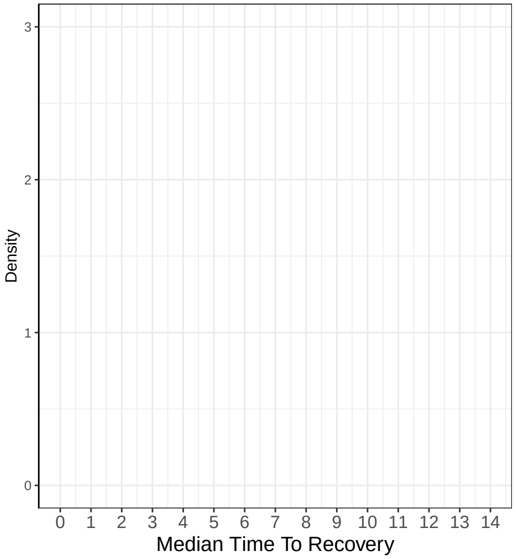

Remove Drug on May 22, 2020

Recovery Data

	Enrolled	Complete	Recovered	Exposure Days	Recoveries Per Day	Estimated Hazard	Estimated HR (95% interval)	Estimated Median Time to Recovery
SOC	207	143	134	1817	0.074			
HCQ	206	147	140	1861	0.075			
Azith	0	0	0	0				
Doxy	0	0	0	0				
Total	413	290	274	3679	0.074			

Hospitalization Data

Hospitalizations	28 Day Completers	Observed Hosp. Rate	Est. Hosp. Rate (95% interval)
2	79	0.0253	
6	78	0.0769	
0	0	NA	
0	0	NA	
8	157	0.0510	

Intervention Status
Enrolling
Removing
Unopened
Unopened

Observed Patient Recoveries

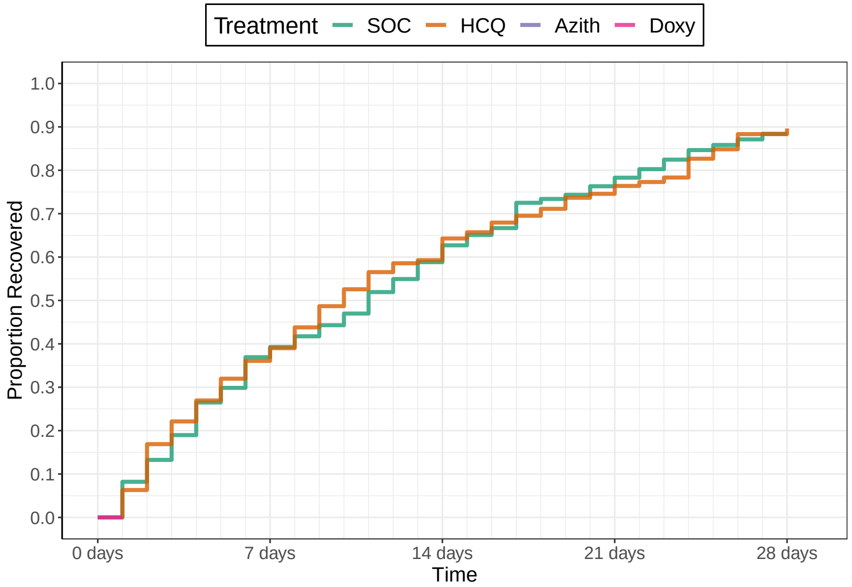

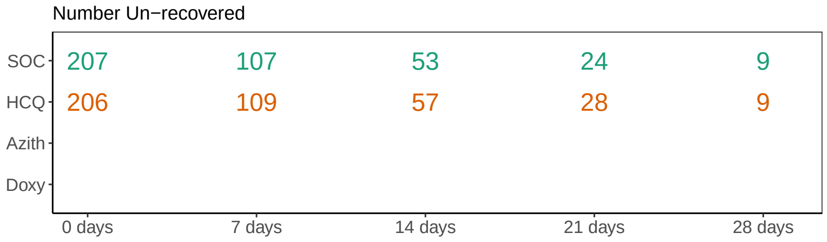

Recovery Inferences

	Pr(Superiority)	Pr(Meaningful Effect)	Pr(Best)	Randomization Probability
SOC				1
HCQ			0	0
Azith			0	0
Doxy			0	0

Hospitalization Inferences

Pr(Superiority)	Pr(Meaningful Effect)

Hazard Ratio Estimates

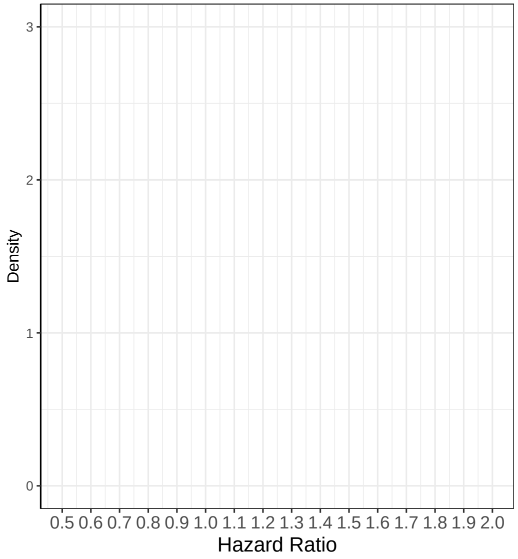

Median Time To Recovery Estimates

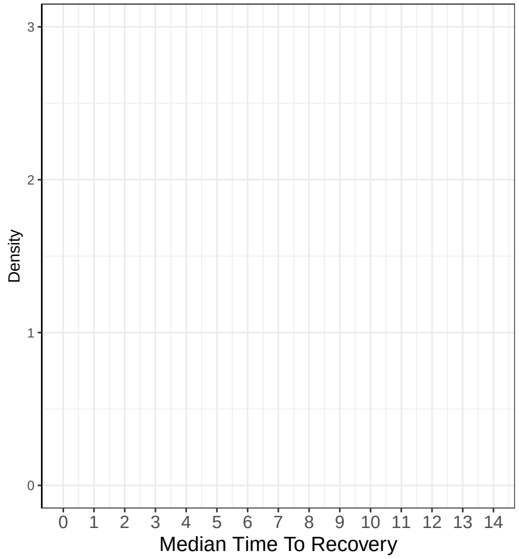

Add Drug on May 23, 2020

Recovery Data

	Enrolled	Complete	Recovered	Exposure Days	Recoveries Per Day	Estimated Hazard	Estimated HR (95% interval)	Estimated Median Time to Recovery
SOC	209	147	138	1881	0.073			
HCQ	206	151	143	1918	0.075			
Azith	0	0	0	0				
Doxy	0	0	0	0				
Total	415	298	281	3799	0.074			

Hospitalization Data

Hospitalizations	28 Day Completers	Observed Hosp. Rate	Est. Hosp. Rate (95% interval)
2	80	0.025	
6	80	0.075	
0	0	NA	
0	0	NA	
8	160	0.050	

Intervention Status
Enrolling
Paused
Introducing
Unopened

Observed Patient Recoveries

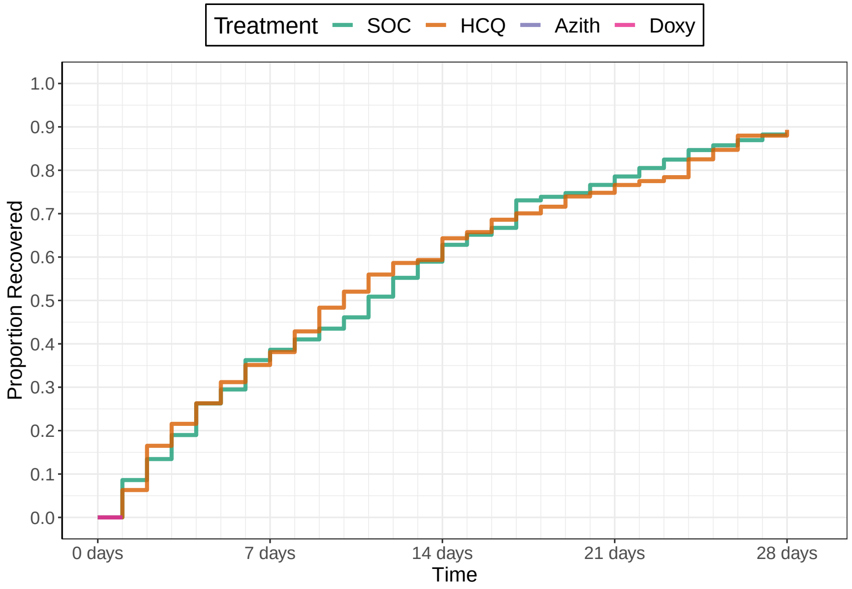

Number Un−recovered

	0 days	7 days	14 days	21 days	28 days
SOC	209	107	53	24	9
HCQ	206	109	57	28	9
Azith					
Doxy					

Recovery Inferences

	Pr(Superiority)	Pr(Meaningful Effect)	Pr(Best)	Randomization Probability
SOC				0.5
HCQ			0	0
Azith			1	0.5
Doxy			0	0

Hospitalization Inferences

Pr(Superiority)	Pr(Meaningful Effect)

Hazard Ratio Estimates

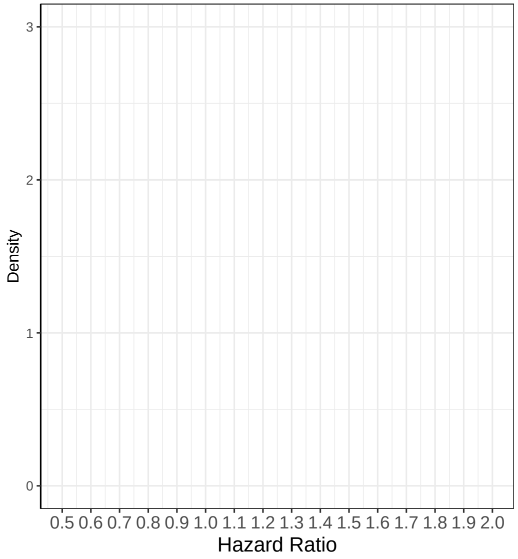

Median Time To Recovery Estimates

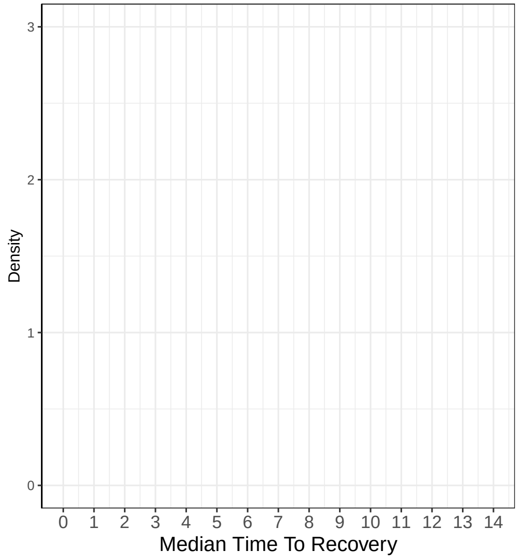

Add Drug on July 23, 2020

Recovery Data

	Enrolled	Complete	Recovered	Exposure Days	Recoveries Per Day	Estimated Hazard	Estimated HR (95% interval)	Estimated Median Time to Recovery
SOC	327	308	272	3615	0.075			
HCQ	206	206	176	2546	0.069			
Azith	118	103	94	1175	0.08			
Doxy	0	0	0	0				
Total	651	617	542	7336	0.074			

Hospitalization Data

Hospitalizations	28 Day Completers	Observed Hosp. Rate	Est. Hosp. Rate (95% interval)
3	286	0.0105	
6	206	0.0291	
8	77	0.1039	
0	0	NA	
17	569	0.0299	

Intervention Status
Enrolling
Paused
Introducing
Introducing

Observed Patient Recoveries

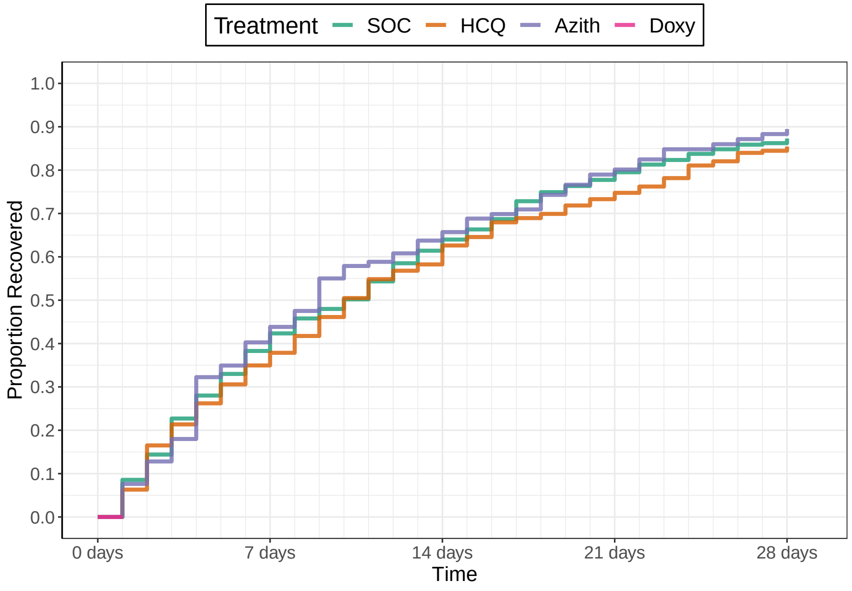

Number Un−recovered

	0 days	7 days	14 days	21 days	28 days
SOC	327	198	120	63	39
HCQ	206	134	86	55	32
Azith	118	67	37	18	10
Doxy					

Recovery Inferences

	Pr(Superiority)	Pr(Meaningful Effect)	Pr(Best)	Randomization Probability
SOC				0.33
HCQ			0	0
Azith			1	0.33
Doxy			0	0.33

Hospitalization Inferences

Pr(Superiority)	Pr(Meaningful Effect)

Hazard Ratio Estimates

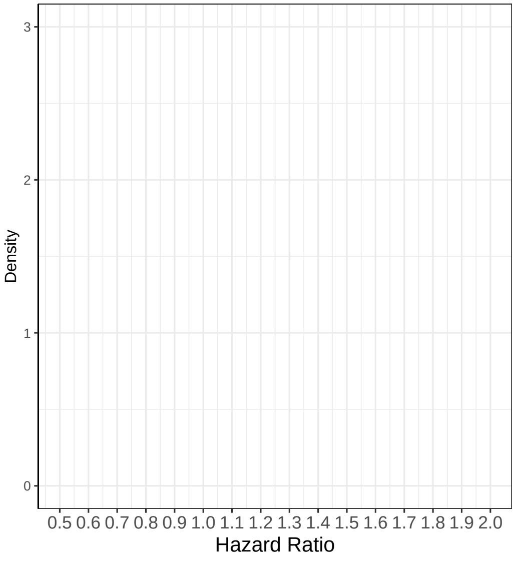

Median Time To Recovery Estimates

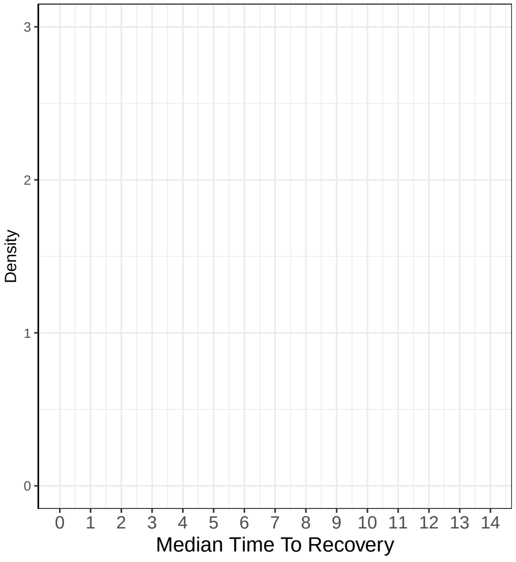

Interim 1 on October 15, 2020

Recovery Data

	Enrolled	Complete	Recovered	Exposure Days	Recoveries Per Day	Estimated Hazard	Estimated HR (95% interval)	Estimated Median Time to Recovery
SOC	496	437	388	5313	0.073	0.071	1	9.61
HCQ	206	206	176	2546	0.069	0.067	0.937 (0.795, 1.113)	10.27
Azith	313	268	252	2762	0.091	0.085	1.202 (1.03, 1.4)	8.01
Doxy	195	151	147	1353	0.109	0.097	1.368 (1.131, 1.625)	7.04
Total	1210	1062	963	11975	0.08			

Hospitalization Data

Hospitalizations	28 Day Completers	Observed Hosp. Rate	Est. Hosp. Rate (95% interval)
9	395	0.0228	0.02 (0.0095, 0.0339)
6	206	0.0291	0.0334 (0.0135, 0.061)
12	198	0.0606	0.0413 (0.0219, 0.0666)
7	74	0.0946	0.0407 (0.0179, 0.0713)
34	873	0.0389	

Intervention Status
LostOnTTR
Futility
SuccessOnTTRFutilityOnHosp Not Announced
Enrolling

Observed Patient Recoveries

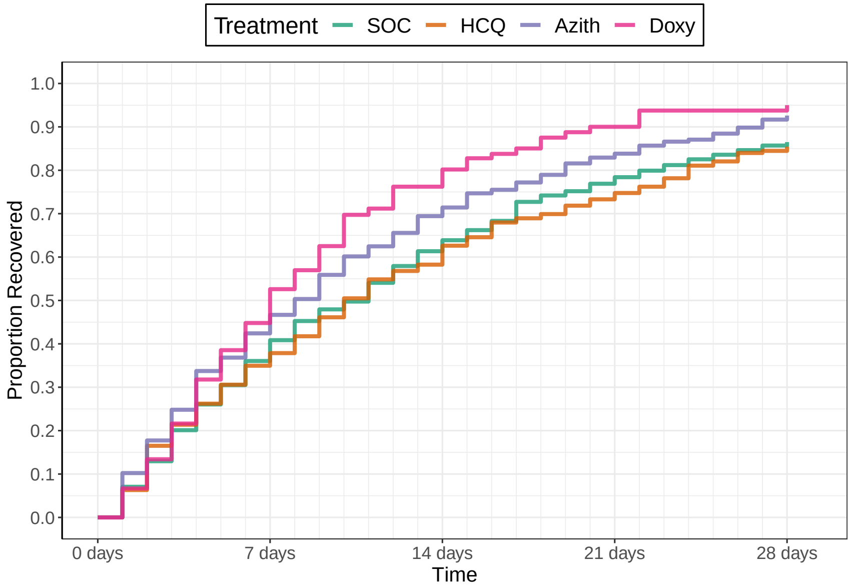

Number Un−recovered

	0 days	7 days	14 days	21 days	28 days
SOC	496	293	168	92	54
HCQ	206	134	86	55	32
Azith	313	162	77	38	18
Doxy	195	92	30	8	5

Recovery Inferences

	Pr(Superiority)	Pr(Meaningful Effect)	Pr(Best)	Randomization Probability
SOC				0.25
HCQ	0.2183	0.009	0	0
Azith	0.9903	0.5687	0.0788	0.25
Doxy	0.9993	0.9257	0.6712	0.5

Hospitalization Inferences

Pr(Superiority)	Pr(Meaningful Effect)
0.1652	0.0038
0.0455	5e−04
0.0712	0

Hazard Ratio Estimates

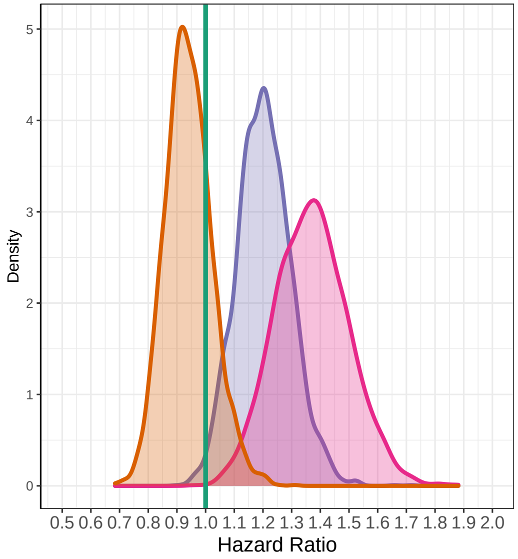

Median Time To Recovery Estimates

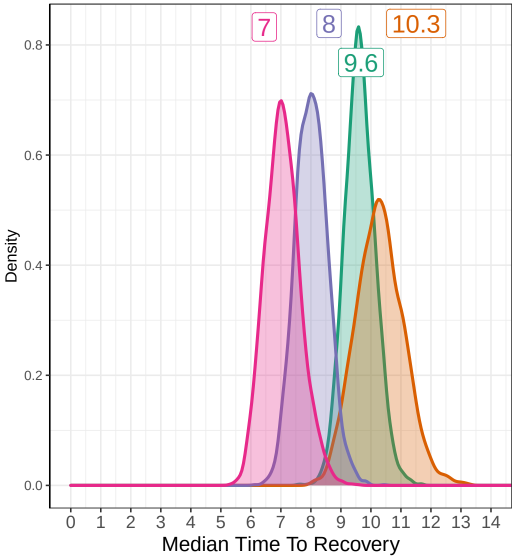

Interim 2 on November 12, 2020

Recovery Data

	Enrolled	Complete	Recovered	Exposure Days	Recoveries Per Day	Estimated Hazard	Estimated HR (95% interval)	Estimated Median Time to Recovery
SOC	550	536	472	6252	0.075	0.073	1	9.28
HCQ	206	206	176	2546	0.069	0.066	0.915 (0.768, 1.081)	10.17
Azith	377	352	327	3592	0.091	0.085	1.173 (1.022, 1.343)	7.89
Doxy	294	275	265	2468	0.107	0.098	1.348 (1.156, 1.548)	6.85
Total	1427	1369	1240	14858	0.083			

Hospitalization Data

Hospitalizations	28 Day Completers	Observed Hosp. Rate	Est. Hosp. Rate (95% interval)
14	496	0.0282	0.0272 (0.0153, 0.0422)
6	206	0.0291	0.034 (0.0137, 0.0619)
15	313	0.0479	0.0423 (0.024, 0.0654)
9	195	0.0462	0.0339 (0.0162, 0.057)
44	1210	0.0364	

Intervention Status
LostOnTTR
Dropped
SuccessOnTTRFutilityOnHosp Not Announced
SuccessOnTTRFutilityOnHosp *Announced*

Observed Patient Recoveries

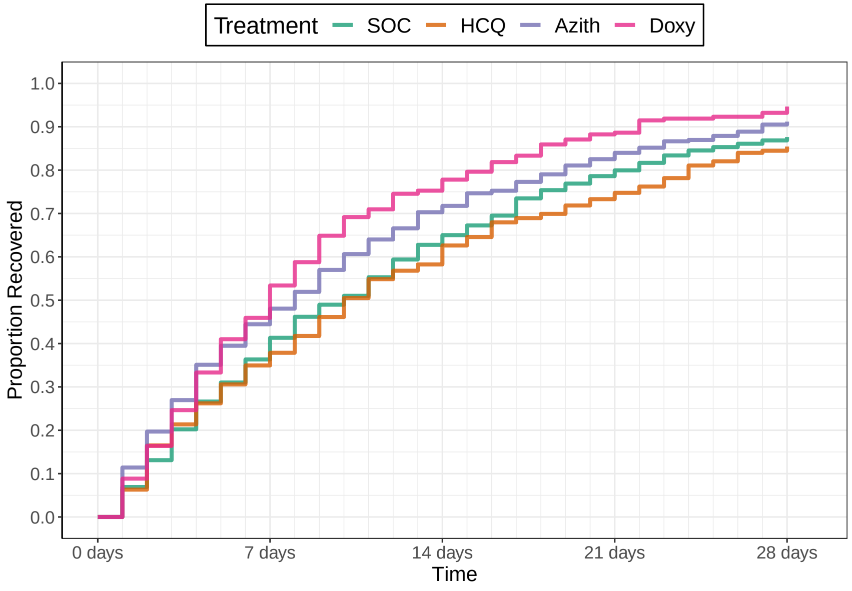

Number Un−recovered

	0 days	7 days	14 days	21 days	28 days
SOC	550	345	199	112	68
HCQ	206	134	86	55	32
Azith	377	201	102	60	28
Doxy	294	152	68	30	14

Recovery Inferences

	Pr(Superiority)	Pr(Meaningful Effect)	Pr(Best)	Randomization Probability
SOC				0.33
HCQ	0.1457	0.002	0	0
Azith	0.987	0.442	0.0415	0.33
Doxy	1	0.937	0.7085	0.33

Hospitalization Inferences

Pr(Superiority)	Pr(Meaningful Effect)
0.3375	0.02
0.1098	0.002
0.3078	0.0132

Hazard Ratio Estimates

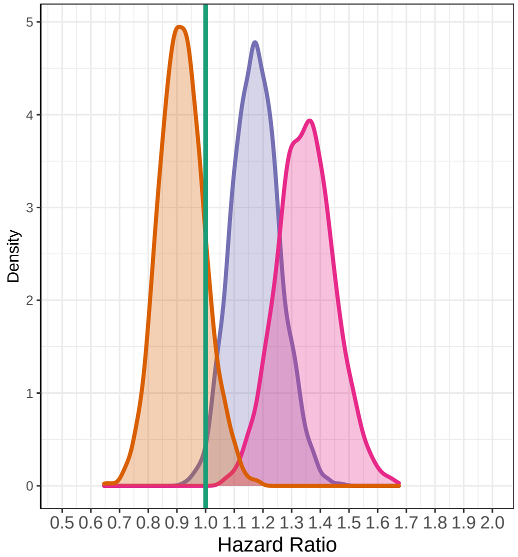

Median Time To Recovery Estimates

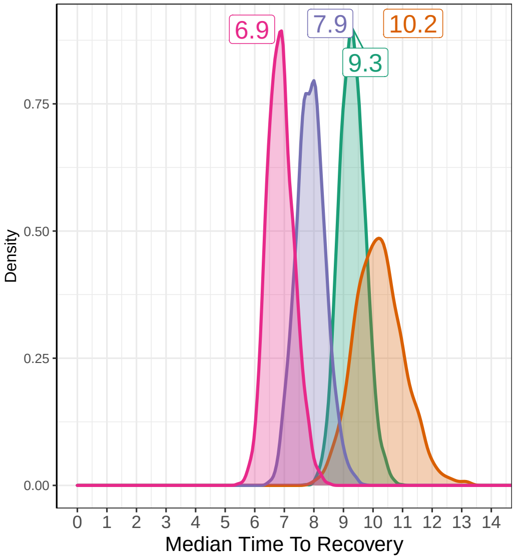

Interim 3 on September 05, 2022

Recovery Data

	Enrolled	Complete	Recovered	Exposure Days	Recoveries Per Day	Estimated Hazard	Estimated HR (95% interval)	Estimated Median Time to Recovery
SOC	2078	2078	1838	24211	0.076	0.074	1	9.53
HCQ	206	206	176	2546	0.069	0.067	0.913 (0.784, 1.056)	10.41
Azith	1918	1918	1769	19480	0.091	0.087	1.185 (1.11, 1.266)	8.08
Doxy	1798	1798	1703	16353	0.104	0.099	1.347 (1.261, 1.443)	7.13
Total	6000	6000	5486	62591	0.088			

Hospitalization Data

Hospitalizations	28 Day Completers	Observed Hosp. Rate	Est. Hosp. Rate (95% interval)
51	2078	0.0245	0.0251 (0.019, 0.0322)
6	206	0.0291	0.0334 (0.0137, 0.0612)
60	1918	0.0313	0.0318 (0.0244, 0.0399)
40	1798	0.0222	0.0227 (0.0164, 0.0299)
157	6000	0.0262	

Intervention Status
Complete
Dropped
SuccessOnTTRFutilityOnHosp Not Announced
SuccessOnTTRFutilityOnHosp *Announced*

Observed Patient Recoveries

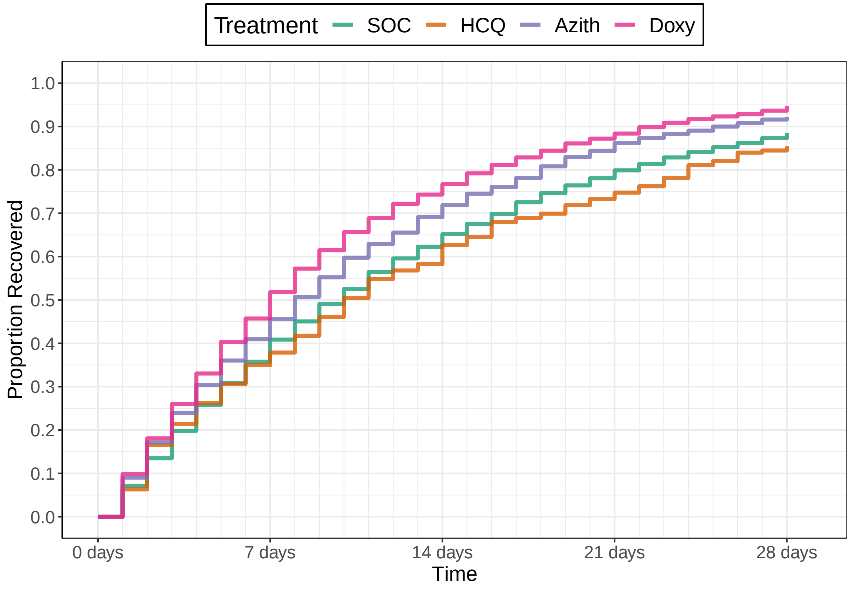

Number Un−recovered

	0 days	7 days	14 days	21 days	28 days
SOC	2078	1335	784	456	263
HCQ	206	134	86	55	32
Azith	1918	1133	593	301	161
Doxy	1798	976	462	230	114

Recovery Inferences

	Pr(Superiority)	Pr(Meaningful Effect)	Pr(Best)	Randomization Probability
SOC				
HCQ	0.1137	3e−04	0	
Azith	1	0.438	2e−04	
Doxy	1	0.9997	0.7498	

Hospitalization Inferences

Pr(Superiority)	Pr(Meaningful Effect)
0.2758	0.0012
0.095	0
0.6968	2e−04

Hazard Ratio Estimates

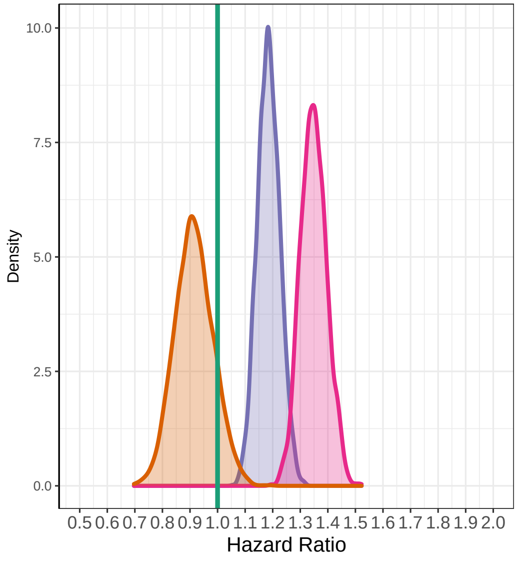

Median Time To Recovery Estimates

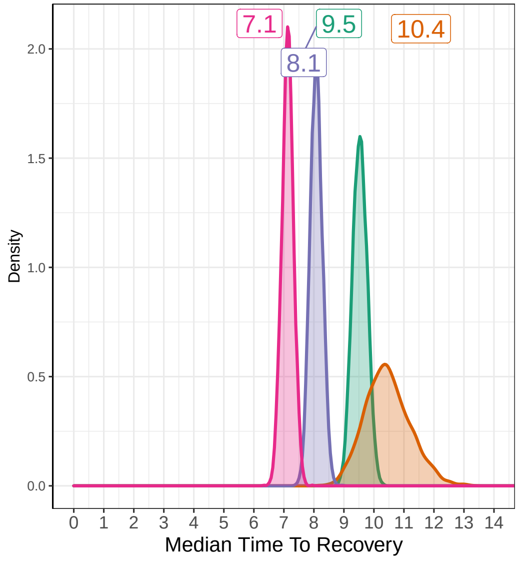

6.3 Example 2

April 1, 2020

Before Patient Enrollment Begins

Recovery Data

	Enrolled	Complete	Recovered	Exposure Days	Recoveries Per Day	Estimated Hazard	Estimated HR (95% interval)	Estimated Median Survival Time
SOC	0	0	0	0				
HCQ	0	0	0	0				
Azith	0	0	0	0				
Doxy	0	0	0	0				
Total	0	0	0	0				

Hospitalization Data

Hospitalizations	28 Day Completers	Observed Hosp. Rate	Est. Hosp. Rate (95% interval)
0	0		
0	0		
0	0		
0	0		
0	0		

Intervention Status
Enrolling
Enrolling
Unopened
Unopened

Observed Patient Recoveries

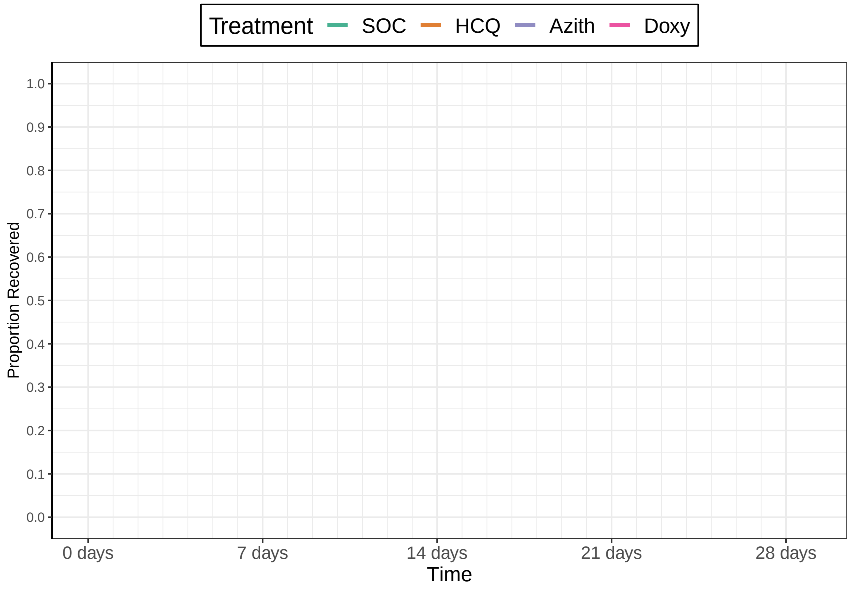

Number Un−recovered

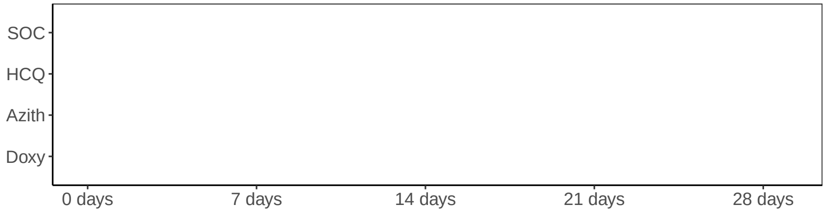

Recovery Inferences

	Pr(Superiority)	Pr(Meaningful Effect)	Pr(Best)	Randomization Probability
SOC				0.5
HCQ				0.5
Azith				0
Doxy				0

Hospitalization Inferences

Pr(Superiority)	Pr(Meaningful Effect)
0	0
0	0
0	0
0	0

Hazard Ratio Estimates

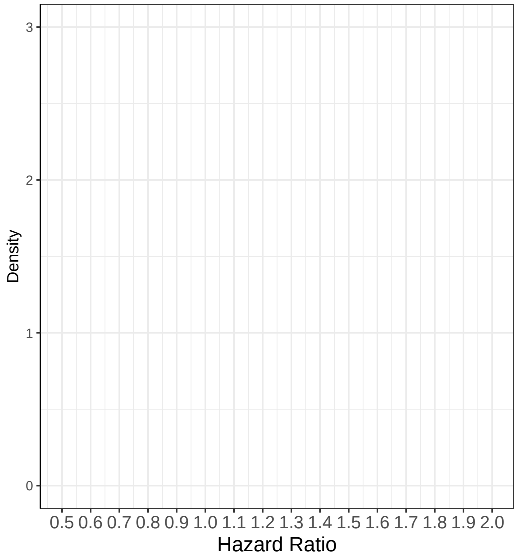

Median Time To Recovery Estimates

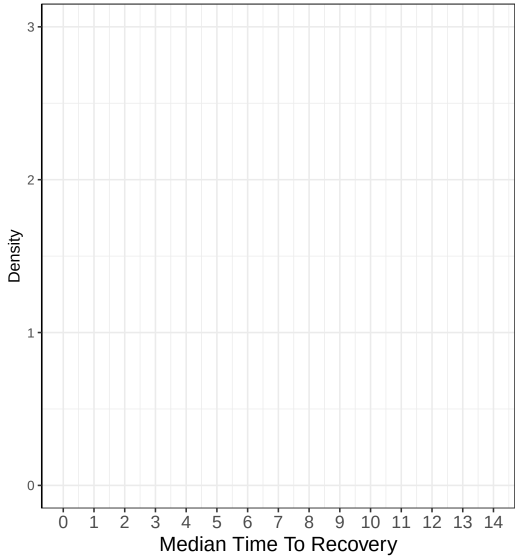

Remove Drug on May 22, 2020

Recovery Data

	Enrolled	Complete	Recovered	Exposure Days	Recoveries Per Day	Estimated Hazard	Estimated HR (95% interval)	Estimated Median Time to Recovery
SOC	206	146	136	1807	0.075			
HCQ	207	149	143	1737	0.082			
Azith	0	0	0	0				
Doxy	0	0	0	0				
Total	413	295	279	3544	0.079			

Hospitalization Data

Hospitalizations	28 Day Completers	Observed Hosp. Rate	Est. Hosp. Rate (95% interval)
1	78	0.0128	
10	79	0.1266	
0	0	NA	
0	0	NA	
11	157	0.0701	

Intervention Status
Enrolling
Removing
Unopened
Unopened

Observed Patient Recoveries

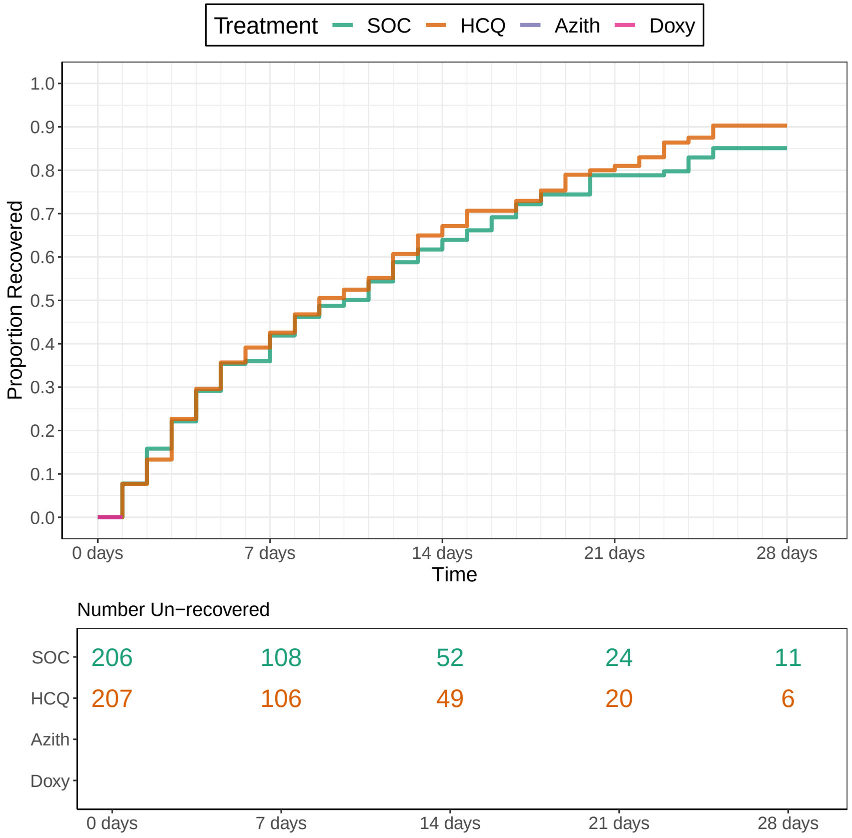

Recovery Inferences

	Pr(Superiority)	Pr(Meaningful Effect)	Pr(Best)	Randomization Probability
SOC				1
HCQ			0	0
Azith			0	0
Doxy			0	0

Hospitalization Inferences

Pr(Superiority)	Pr(Meaningful Effect)

Hazard Ratio Estimates

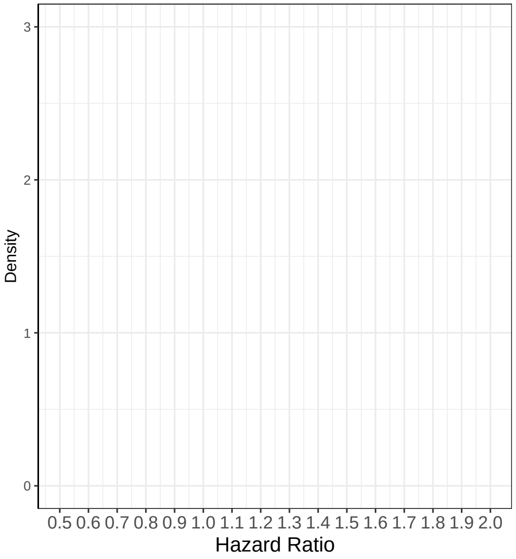

Median Time To Recovery Estimates

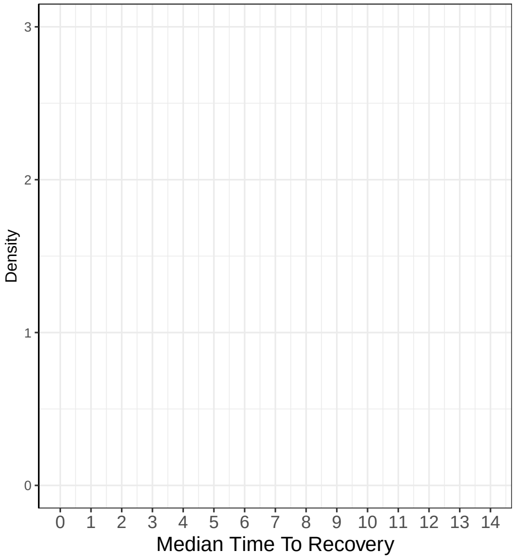

Add Drug on May 23, 2020

Recovery Data

	Enrolled	Complete	Recovered	Exposure Days	Recoveries Per Day	Estimated Hazard	Estimated HR (95% interval)	Estimated Median Time to Recovery
SOC	208	151	140	1866	0.075			
HCQ	207	154	148	1793	0.083			
Azith	0	0	0	0				
Doxy	0	0	0	0				
Total	415	305	288	3659	0.079			

Hospitalization Data

Hospitalizations	28 Day Completers	Observed Hosp. Rate	Est. Hosp. Rate (95% interval)
1	80	0.0125	
10	80	0.1250	
0	0	NA	
0	0	NA	
11	160	0.0688	

Intervention Status
Enrolling
Paused
Introducing
Unopened

Observed Patient Recoveries

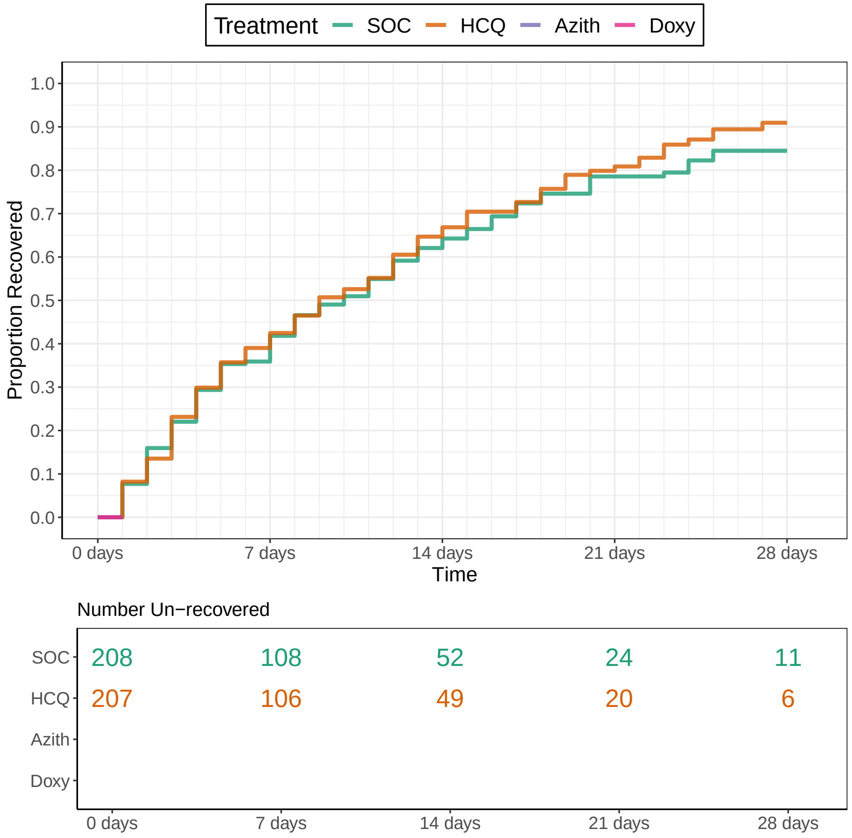

Recovery Inferences

	Pr(Superiority)	Pr(Meaningful Effect)	Pr(Best)	Randomization Probability
SOC				0.5
HCQ			0	0
Azith			1	0.5
Doxy			0	0

Hospitalization Inferences

Pr(Superiority)	Pr(Meaningful Effect)

Hazard Ratio Estimates

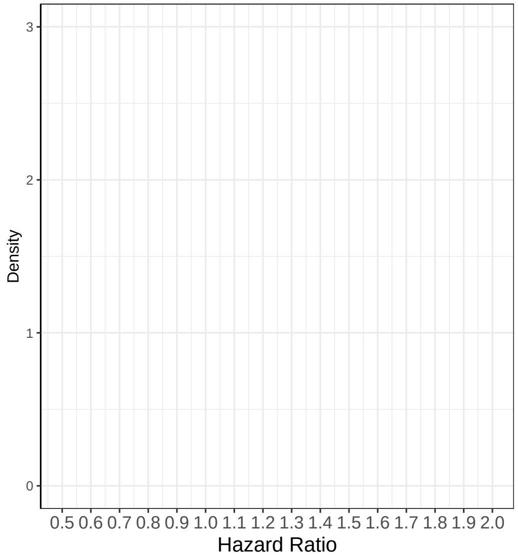

Median Time To Recovery Estimates

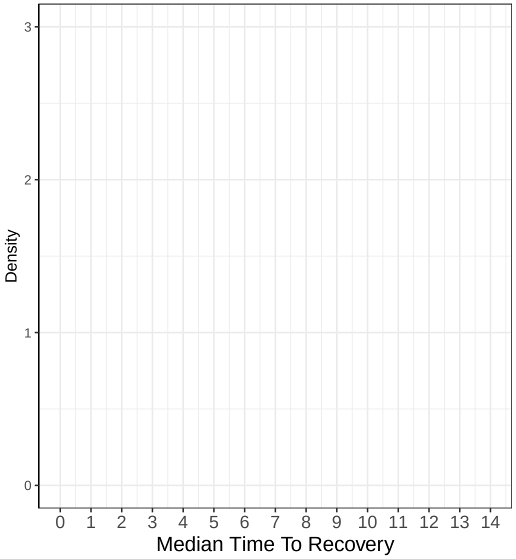

Add Drug on July 23, 2020

Recovery Data

	Enrolled	Complete	Recovered	Exposure Days	Recoveries Per Day	Estimated Hazard	Estimated HR (95% interval)	Estimated Median Time to Recovery
SOC	326	308	270	3608	0.075			
HCQ	207	207	181	2375	0.076			
Azith	118	106	97	1148	0.084			
Doxy	0	0	0	0				
Total	651	621	548	7132	0.077			

Hospitalization Data

Hospitalizations	28 Day Completers	Observed Hosp. Rate	Est. Hosp. Rate (95% interval)
7	285	0.0246	
10	207	0.0483	
4	77	0.0519	
0	0	NA	
21	569	0.0369	

Intervention Status
Enrolling
Paused
Introducing
Introducing

Observed Patient Recoveries

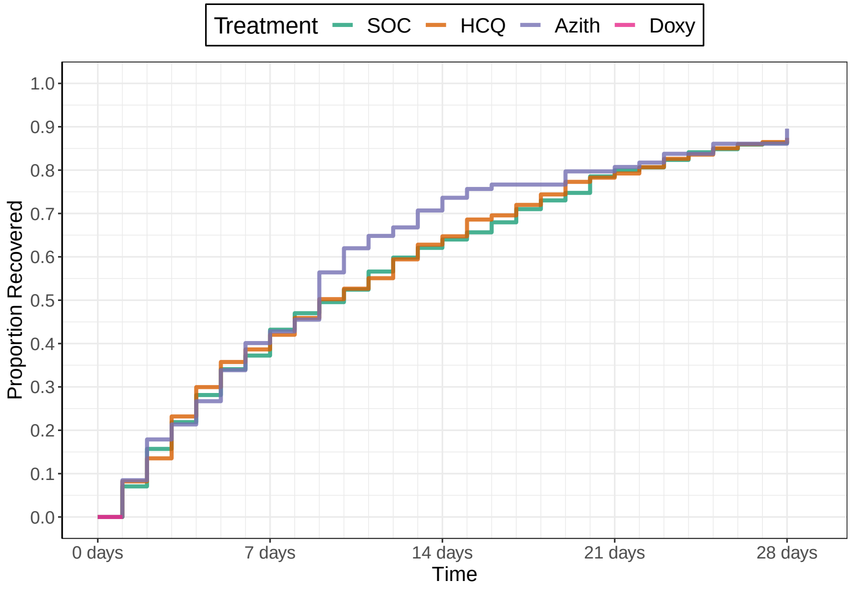

Number Un−recovered

	0 days	7 days	14 days	21 days	28 days
SOC	326	200	118	62	39
HCQ	207	127	77	45	28
Azith	118	67	30	20	12
Doxy					

Recovery Inferences

	Pr(Superiority)	Pr(Meaningful Effect)	Pr(Best)	Randomization Probability
SOC				0.33
HCQ			0	0
Azith			1	0.33
Doxy			0	0.33

Hospitalization Inferences

Pr(Superiority)	Pr(Meaningful Effect)

Hazard Ratio Estimates

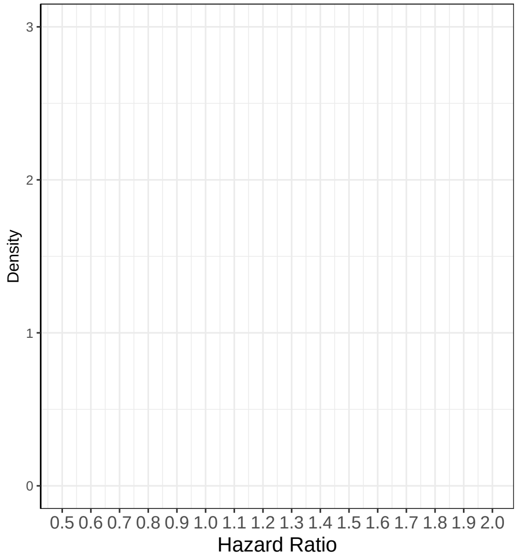

Median Time To Recovery Estimates

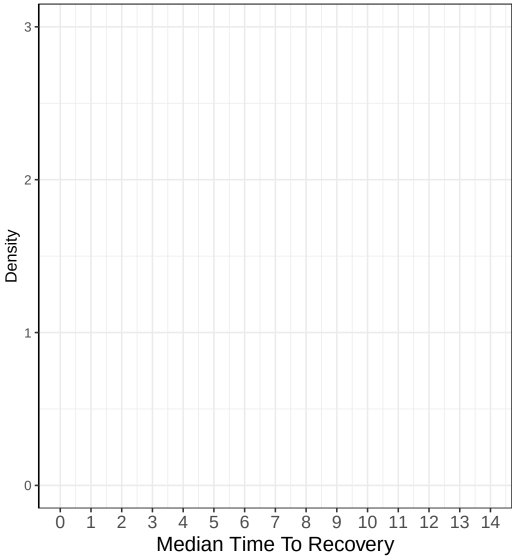

Interim 1 on October 15, 2020

Recovery Data

	Enrolled	Complete	Recovered	Exposure Days	Recoveries Per Day	Estimated Hazard	Estimated HR (95% interval)	Estimated Median Time to Recovery
SOC	515	461	415	5407	0.077	0.074	1	9.22
HCQ	207	207	181	2375	0.076	0.073	0.985 (0.835, 1.162)	9.36
Azith	309	256	234	2846	0.082	0.077	1.045 (0.907, 1.206)	8.81
Doxy	179	138	133	1309	0.102	0.092	1.246 (1.029, 1.497)	7.35
Total	1210	1062	963	11937	0.081			

Hospitalization Data

Hospitalizations	28 Day Completers	Observed Hosp. Rate	Est. Hosp. Rate (95% interval)
19	400	0.0475	0.0387 (0.0238, 0.0573)
10	207	0.0483	0.0528 (0.0274, 0.0866)
6	192	0.0312	0.0223 (0.0089, 0.0407)
12	74	0.1622	0.0713 (0.0387, 0.1129)
47	873	0.0538	

Intervention Status
Enrolling
Futility
Enrolling
Enrolling

Observed Patient Recoveries

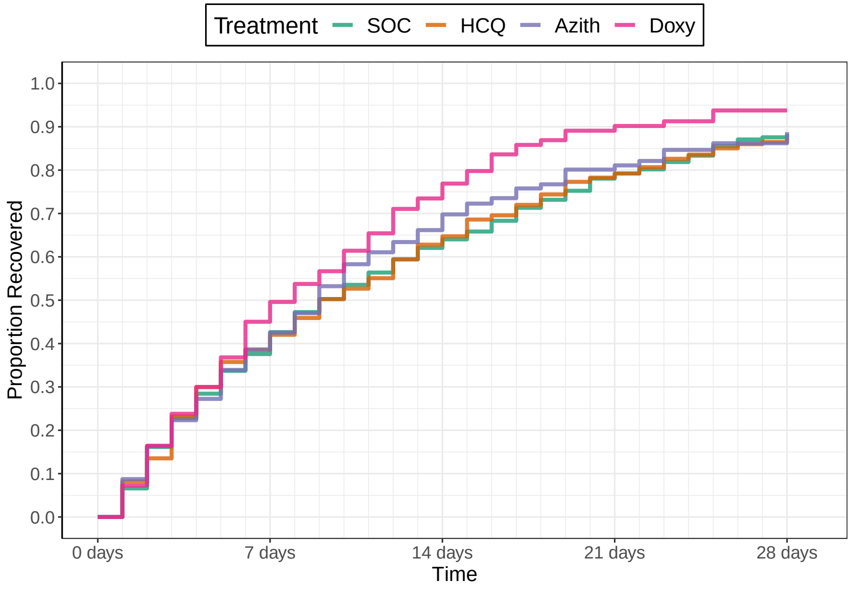

Number Un−recovered

	0 days	7 days	14 days	21 days	28 days
SOC	515	297	173	93	49
HCQ	207	127	77	45	28
Azith	309	169	84	41	27
Doxy	179	84	31	10	5

Recovery Inferences

	Pr(Superiority)	Pr(Meaningful Effect)	Pr(Best)	Randomization Probability
SOC				0.33
HCQ	0.4313	0.0207	0	0
Azith	0.7207	0.057	0.0362	0.28
Doxy	0.9853	0.6817	0.7138	0.39

Hospitalization Inferences

Pr(Superiority)	Pr(Meaningful Effect)
0.2122	0.0148
0.9192	0.3777
0.0478	0.0018

Hazard Ratio Estimates

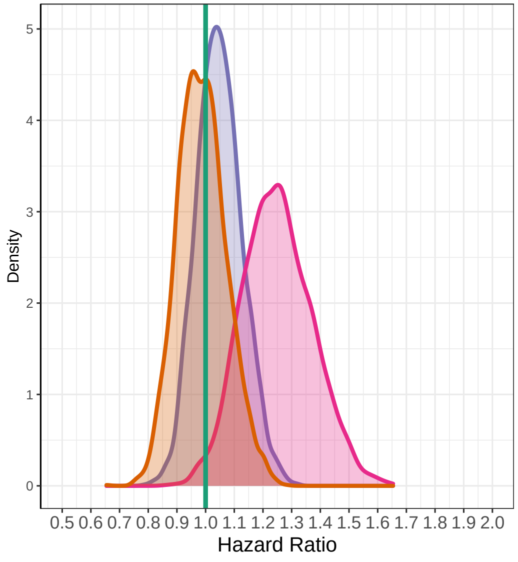

Median Time To Recovery Estimates

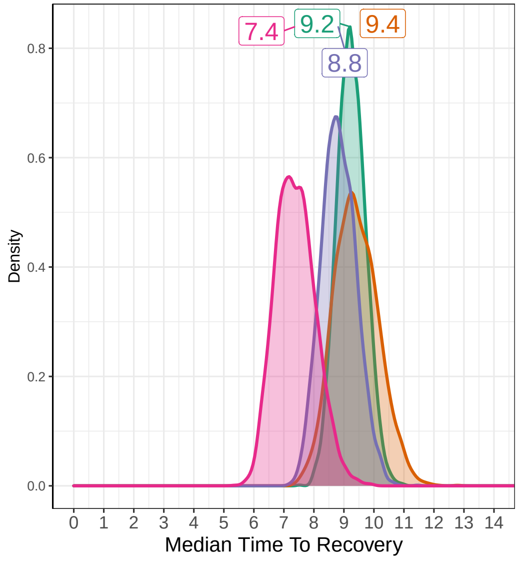

Interim 2 on November 12, 2020

Recovery Data

	Enrolled	Complete	Recovered	Exposure Days	Recoveries Per Day	Estimated Hazard	Estimated HR (95% interval)	Estimated Median Time to Recovery
SOC	593	575	512	6654	0.077	0.074	1	9.23
HCQ	207	207	181	2375	0.076	0.073	0.981 (0.83, 1.152)	9.43
Azith	375	351	321	3888	0.083	0.078	1.053 (0.923, 1.206)	8.77
Doxy	273	243	233	2182	0.107	0.098	1.323 (1.143, 1.53)	7.01
Total	1448	1376	1247	15100	0.083			

Hospitalization Data

Hospitalizations	28 Day Completers	Observed Hosp. Rate	Est. Hosp. Rate (95% interval)
22	515	0.0427	0.0387 (0.0251, 0.0548)
10	207	0.0483	0.0526 (0.0266, 0.0875)
7	309	0.0227	0.0212 (0.0092, 0.0381)
14	179	0.0782	0.054 (0.0308, 0.0841)
53	1210	0.0438	

Intervention Status
LostOnTTR
Dropped
InferiorToDoxy
SuccessOnTTRFutilityOnHosp *Announced*

Observed Patient Recoveries

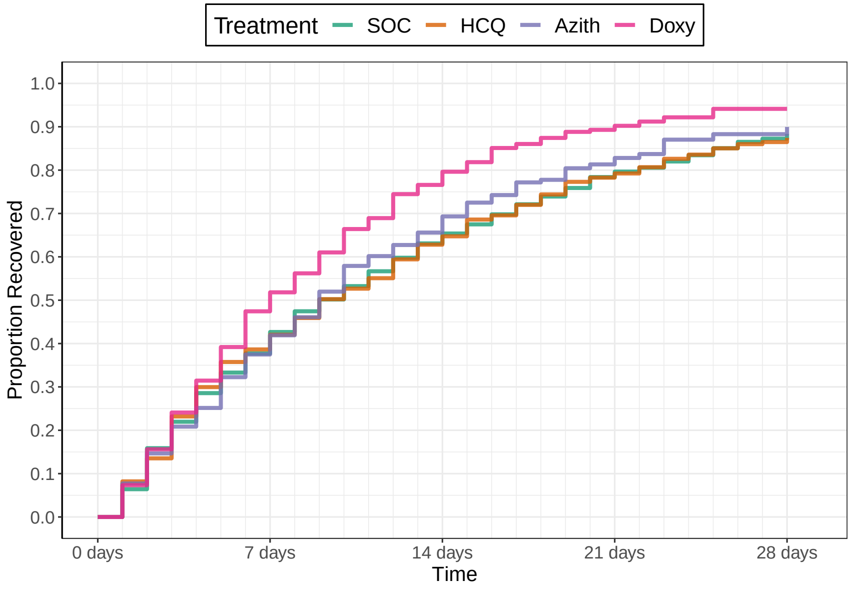

Number Un−recovered

	0 days	7 days	14 days	21 days	28 days
SOC	593	365	210	120	69
HCQ	207	127	77	45	28
Azith	375	226	120	63	35
Doxy	273	132	54	23	12

Recovery Inferences

	Pr(Superiority)	Pr(Meaningful Effect)	Pr(Best)	Randomization Probability
SOC				0.5
HCQ	0.4057	0.0127	0	0
Azith	0.772	0.0437	0	0
Doxy	0.9997	0.9037	0.75	0.5

Hospitalization Inferences

Pr(Superiority)	Pr(Meaningful Effect)
0.2158	0.0158
0.9422	0.4105
0.1645	0.0045

Hazard Ratio Estimates

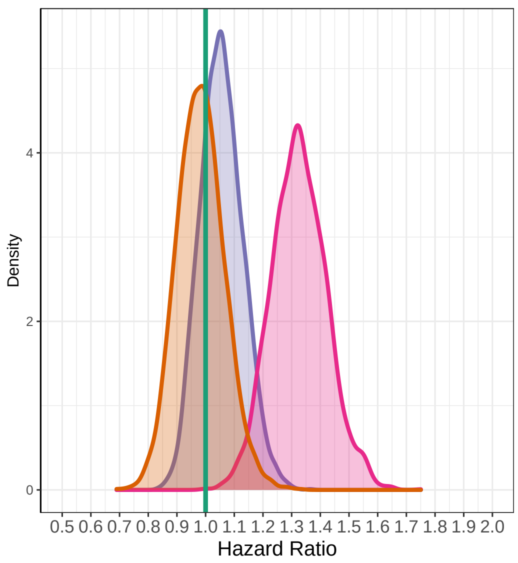

Median Time To Recovery Estimates

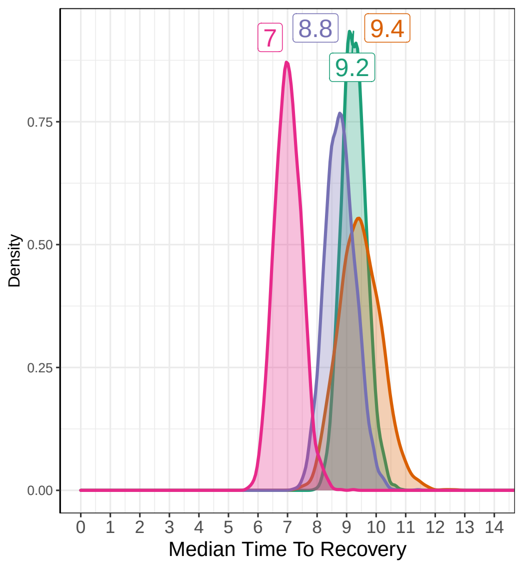

Interim 3 on August 30, 2022

Recovery Data

	Enrolled	Complete	Recovered	Exposure Days	Recoveries Per Day	Estimated Hazard	Estimated HR (95% interval)	Estimated Median Time to Recovery
SOC	2851	2851	2499	33636	0.074	0.072	1	9.69
HCQ	207	207	181	2375	0.076	0.073	1.021 (0.876, 1.173)	9.5
Azith	375	375	339	4072	0.083	0.08	1.11 (0.991, 1.238)	8.75
Doxy	2567	2567	2417	23633	0.102	0.097	1.354 (1.282, 1.431)	7.16
Total	6000	6000	5436	63716	0.085			

Hospitalization Data

Hospitalizations	28 Day Completers	Observed Hosp. Rate	Est. Hosp. Rate (95% interval)
83	2851	0.0291	0.0294 (0.0235, 0.0362)
10	207	0.0483	0.053 (0.0272, 0.0879)
7	375	0.0187	0.0212 (0.0093, 0.0381)
62	2567	0.0242	0.0246 (0.0188, 0.0306)
162	6000	0.0270	

Intervention Status
Complete
Dropped
Dropped
SuccessOnTTRFutilityOnHosp *Announced*

Observed Patient Recoveries

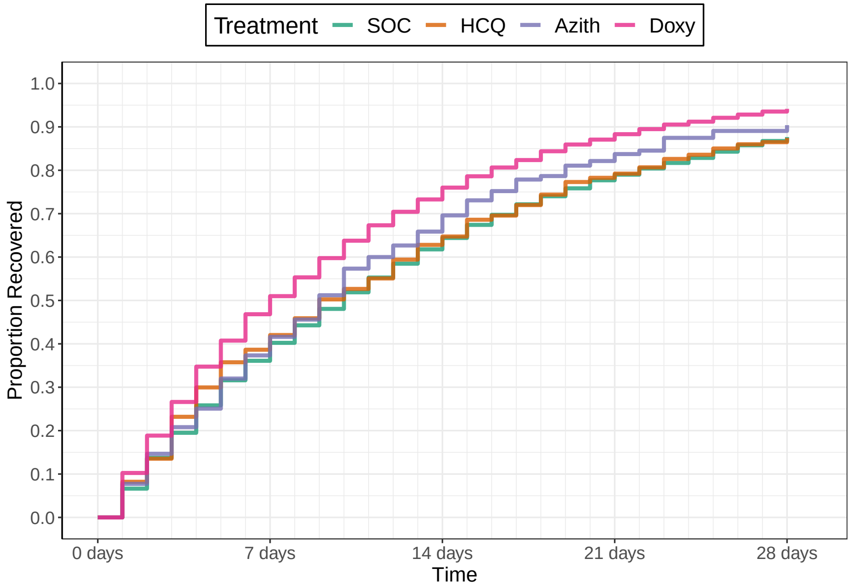

Number Un−recovered

	0 days	7 days	14 days	21 days	28 days
SOC	2851	1822	1090	636	378
HCQ	207	127	77	45	28
Azith	375	235	128	67	41
Doxy	2567	1365	686	332	166

Recovery Inferences

	Pr(Superiority)	Pr(Meaningful Effect)	Pr(Best)	Randomization Probability
SOC				
HCQ	0.6043	0.021	0	
Azith	0.966	0.1283	0	
Doxy	1	1	0.75	

Hospitalization Inferences

Pr(Superiority)	Pr(Meaningful Effect)
0.0495	0
0.8518	0.056
0.866	2e−04

Hazard Ratio Estimates

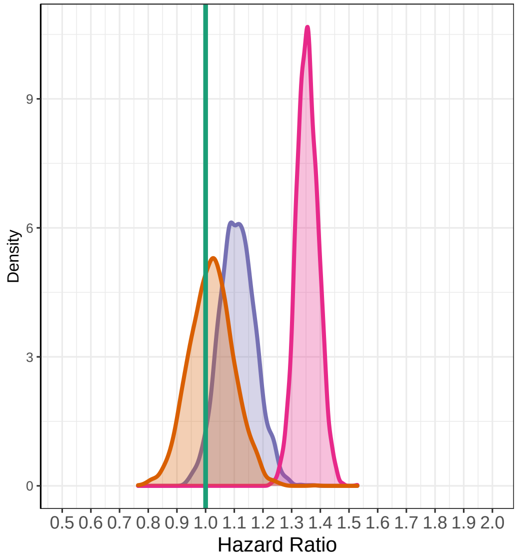

Median Time To Recovery Estimates

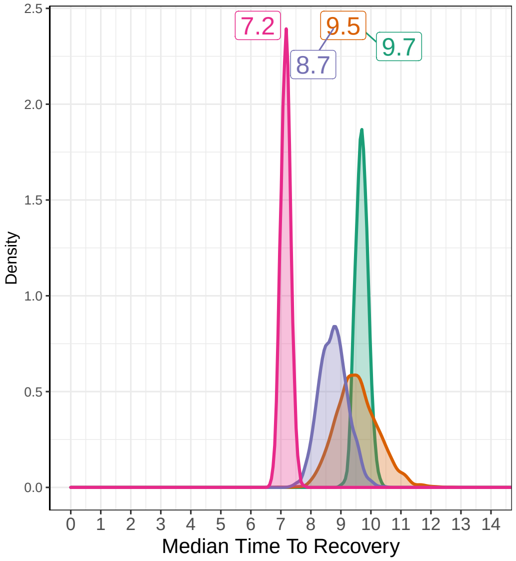

6.4 Example 3

April 1, 2020

Before Patient Enrollment Begins

Recovery Data

	Enrolled	Complete	Recovered	Exposure Days	Recoveries Per Day	Estimated Hazard	Estimated HR (95% interval)	Estimated Median Survival Time
SOC	0	0	0	0				
HCQ	0	0	0	0				
Azith	0	0	0	0				
Doxy	0	0	0	0				
Total	0	0	0	0				

Hospitalization Data

Hospitalizations	28 Day Completers	Observed Hosp. Rate	Est. Hosp. Rate (95% interval)
0	0		
0	0		
0	0		
0	0		
0	0		

Intervention Status
Enrolling
Enrolling
Unopened
Unopened

Observed Patient Recoveries

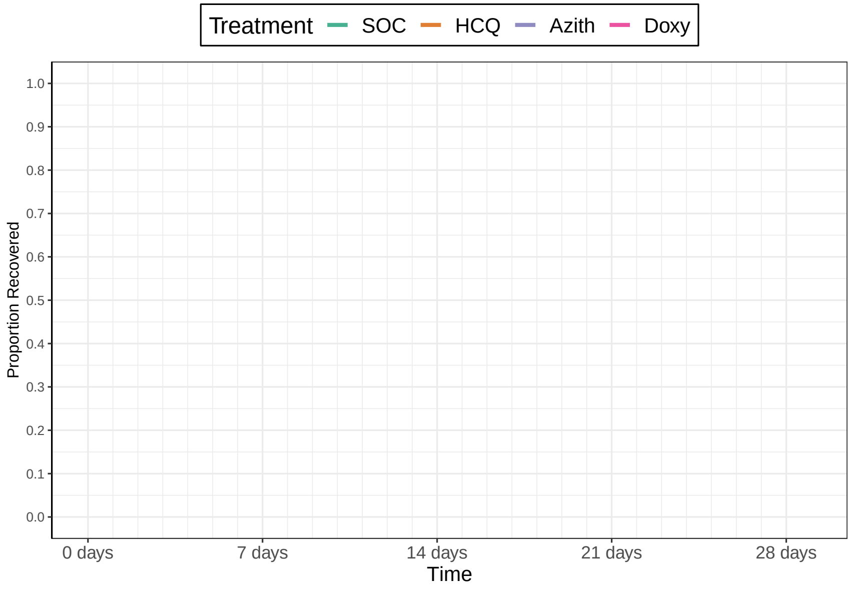

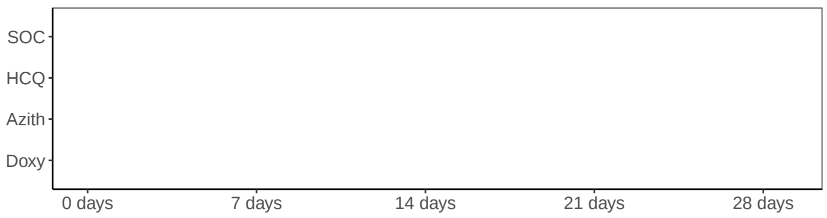

Recovery Inferences

	Pr(Superiority)	Pr(Meaningful Effect)	Pr(Best)	Randomization Probability
SOC				0.5
HCQ				0.5
Azith				0
Doxy				0

Hospitalization Inferences

Pr(Superiority)	Pr(Meaningful Effect)
0	0
0	0
0	0
0	0

Hazard Ratio Estimates

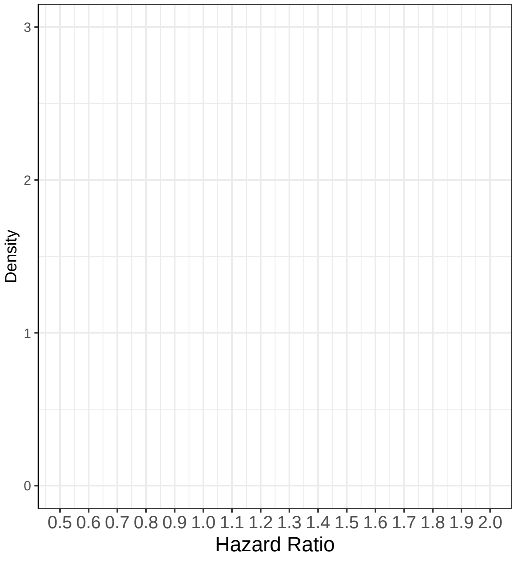

Median Time To Recovery Estimates

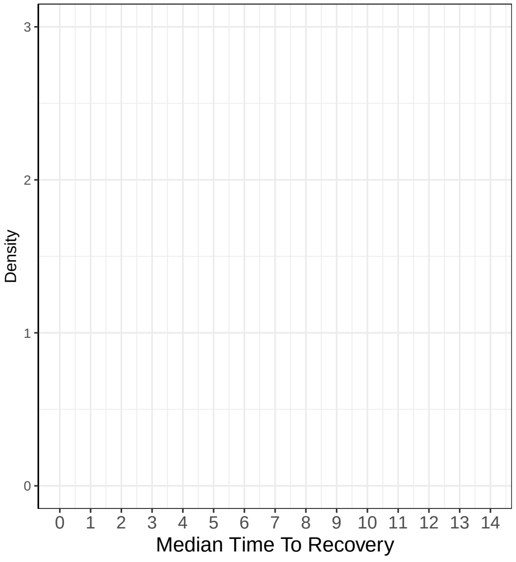

Remove Drug on May 22, 2020

Recovery Data

	Enrolled	Complete	Recovered	Exposure Days	Recoveries Per Day	Estimated Hazard	Estimated HR (95% interval)	Estimated Median Time to Recovery
SOC	206	150	141	1753	0.08			
HCQ	207	156	147	1684	0.087			
Azith	0	0	0	0				
Doxy	0	0	0	0				
Total	413	306	288	3437	0.084			

Hospitalization Data

Hospitalizations	28 Day Completers	Observed Hosp. Rate	Est. Hosp. Rate (95% interval)
5	78	0.0641	
7	79	0.0886	
0	0	NA	
0	0	NA	
12	157	0.0764	

Intervention Status
Enrolling
Removing
Unopened
Unopened

Observed Patient Recoveries

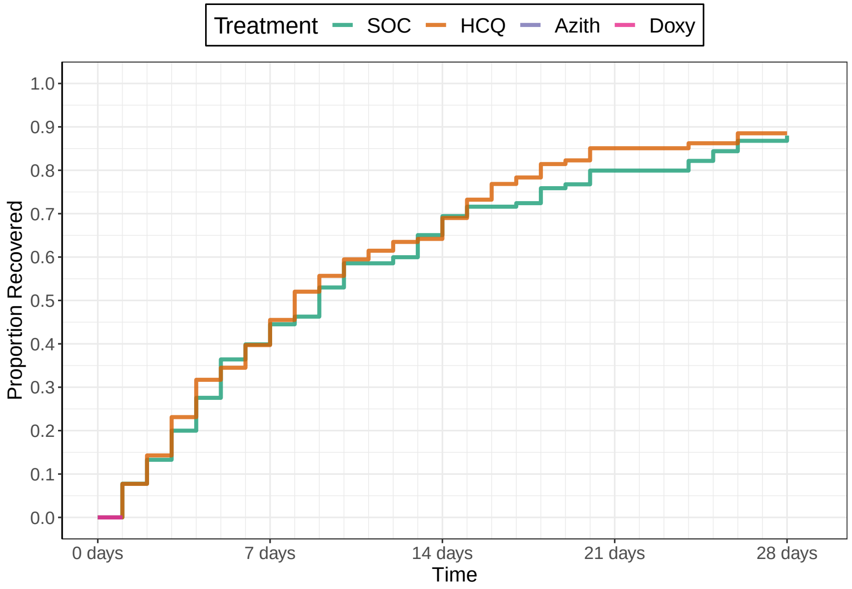

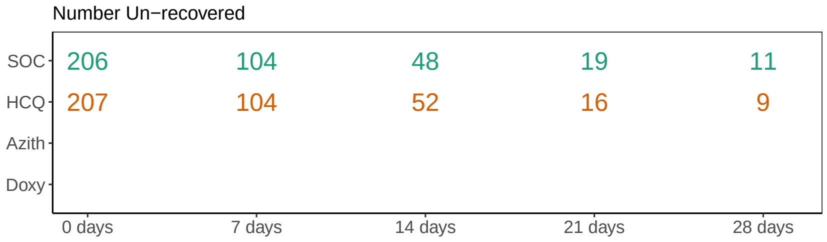

Recovery Inferences

	Pr(Superiority)	Pr(Meaningful Effect)	Pr(Best)	Randomization Probability
SOC				1
HCQ			0	0
Azith			0	0
Doxy			0	0

Hospitalization Inferences

Pr(Superiority)	Pr(Meaningful Effect)

Hazard Ratio Estimates

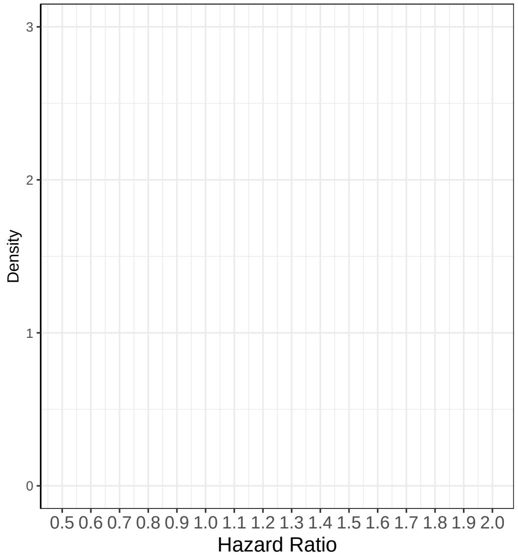

Median Time To Recovery Estimates

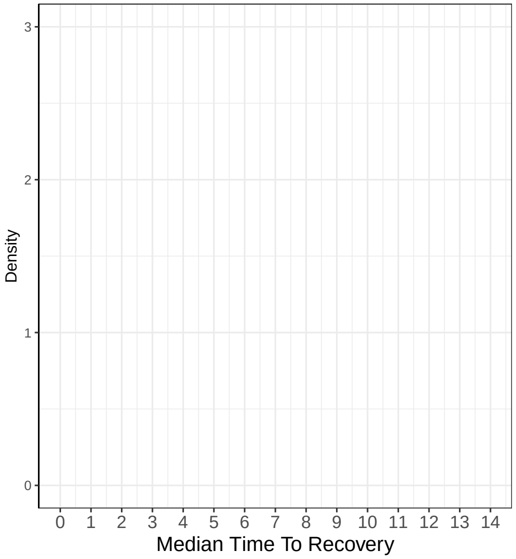

Add Drug on May 23, 2020

Recovery Data

	Enrolled	Complete	Recovered	Exposure Days	Recoveries Per Day	Estimated Hazard	Estimated HR (95% interval)	Estimated Median Time to Recovery
SOC	208	155	145	1808	0.08			
HCQ	207	166	157	1728	0.091			
Azith	0	0	0	0				
Doxy	0	0	0	0				
Total	415	321	302	3536	0.085			

Hospitalization Data

Hospitalizations	28 Day Completers	Observed Hosp. Rate	Est. Hosp. Rate (95% interval)
5	80	0.0625	
7	80	0.0875	
0	0	NA	
0	0	NA	
12	160	0.0750	

Intervention Status
Enrolling
Paused
Introducing
Unopened

Observed Patient Recoveries

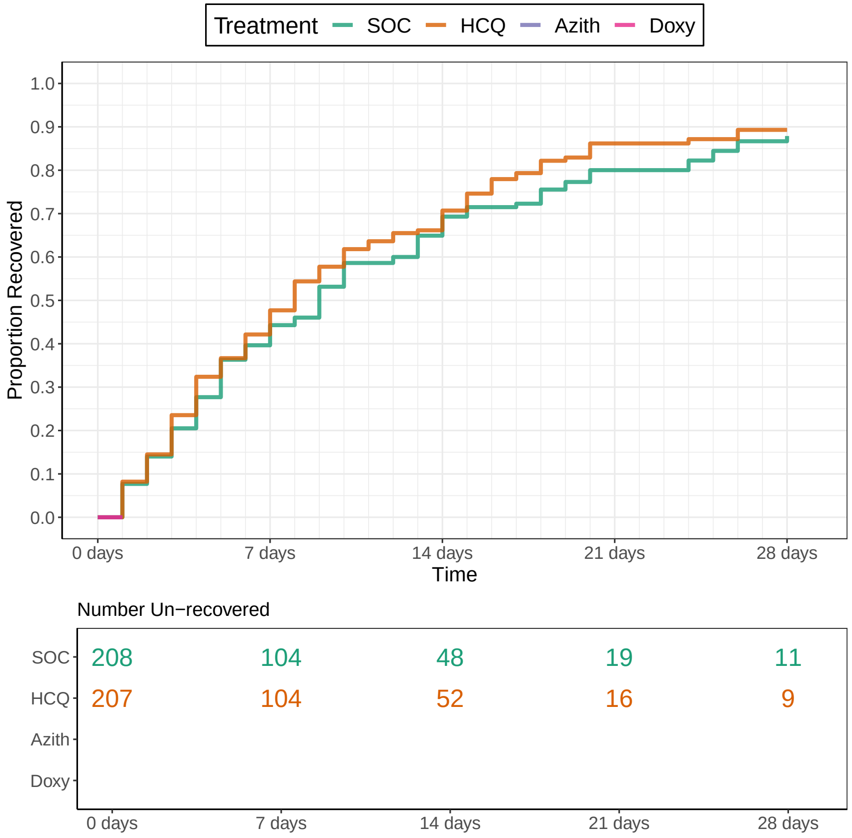

Recovery Inferences

	Pr(Superiority)	Pr(Meaningful Effect)	Pr(Best)	Randomization Probability
SOC				0.5
HCQ			0	0
Azith			1	0.5
Doxy			0	0

Hospitalization Inferences

Pr(Superiority)	Pr(Meaningful Effect)

Hazard Ratio Estimates

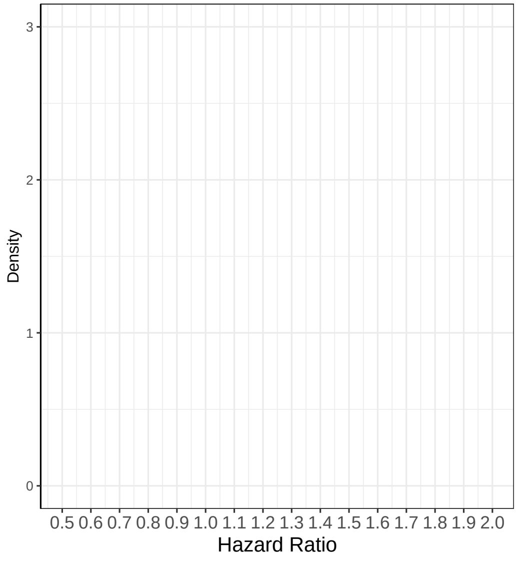

Median Time To Recovery Estimates

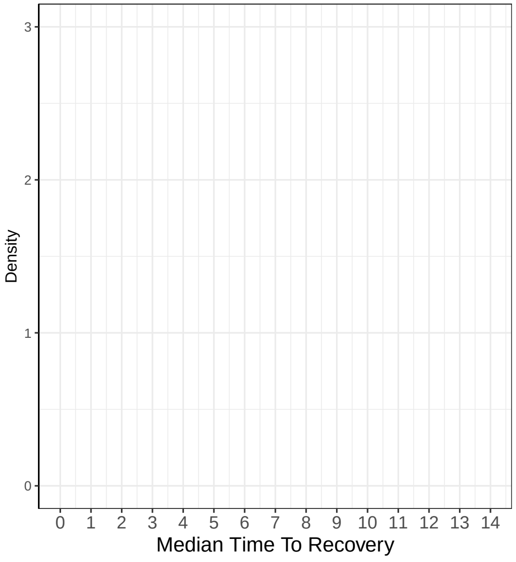

Add Drug on July 23, 2020

Recovery Data

	Enrolled	Complete	Recovered	Exposure Days	Recoveries Per Day	Estimated Hazard	Estimated HR (95% interval)	Estimated Median Time to Recovery
SOC	326	305	275	3462	0.079			
HCQ	207	207	188	2127	0.088			
Azith	118	105	97	1041	0.093			
Doxy	0	0	0	0				
Total	651	617	560	6630	0.084			

Hospitalization Data

Hospitalizations	28 Day Completers	Observed Hosp. Rate	Est. Hosp. Rate (95% interval)
6	285	0.0211	
7	207	0.0338	
3	77	0.0390	
0	0	NA	
16	569	0.0281	

Intervention Status
Enrolling
Paused
Introducing
Introducing

Observed Patient Recoveries

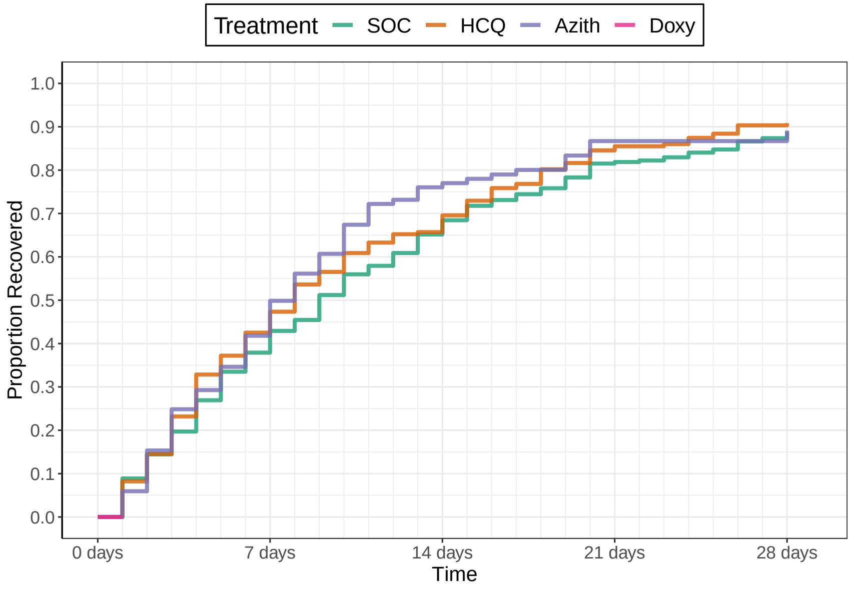

Number Un−recovered

	0 days	7 days	14 days	21 days	28 days
SOC	326	198	106	52	34
HCQ	207	119	71	32	20
Azith	118	65	25	12	11
Doxy					

Recovery Inferences

	Pr(Superiority)	Pr(Meaningful Effect)	Pr(Best)	Randomization Probability
SOC				0.33
HCQ			0	0
Azith			1	0.33
Doxy			0	0.33

Hospitalization Inferences

Pr(Superiority)	Pr(Meaningful Effect)

Hazard Ratio Estimates

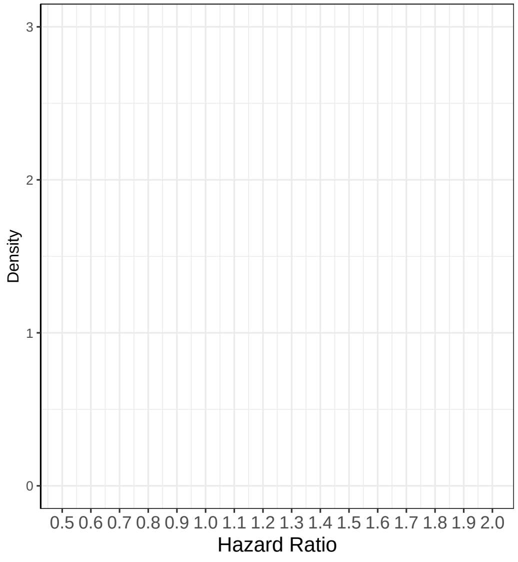

Median Time To Recovery Estimates

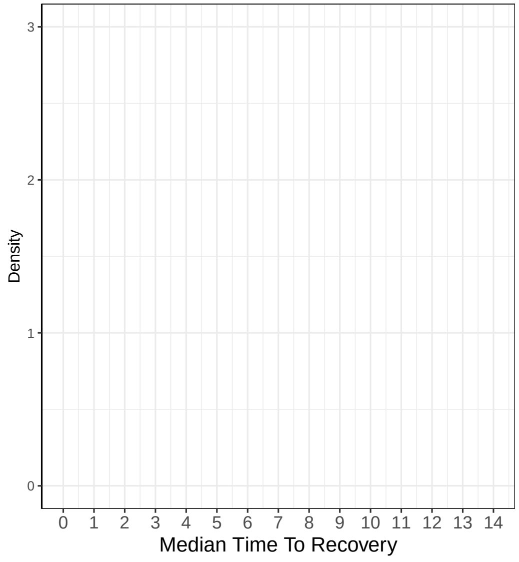

Interim 1 on October 15, 2020

Recovery Data	Enrolled	Complete	Recovered	Exposure Days	Recoveries Per Day	Estimated Hazard	Estimated HR (95% interval)	Estimated Median Time to Recovery
SOC	519	477	433	5488	0.079	0.073	1	8.96
HCQ	207	207	188	2127	0.088	0.08	1.098 (0.937, 1.295)	8.18
Azith	317	262	242	2746	0.088	0.079	1.083 (0.928, 1.255)	8.28
Doxy	167	118	108	1301	0.083	0.075	1.019 (0.831, 1.239)	8.79
Total	1210	1064	971	11662	0.083			

Hospitalization Data

Hospitalizations	28 Day Completers	Observed Hosp. Rate	Est. Hosp. Rate (95% interval)
14	419	0.0334	0.0287 (0.0162, 0.0445)
7	207	0.0338	0.038 (0.0165, 0.0671)
9	185	0.0486	0.0314 (0.0155, 0.0526)
6	62	0.0968	0.0409 (0.017, 0.0745)
36	873	0.0412	

Intervention Status
Enrolling
Futility
Enrolling
Enrolling

Observed Patient Recoveries

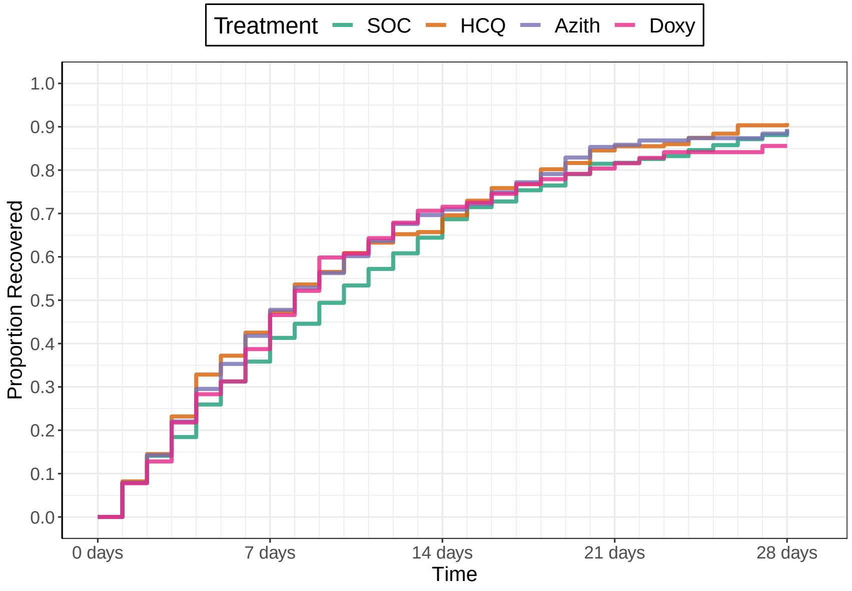

Number Un−recovered

	0 days	7 days	14 days	21 days	28 days
SOC	519	317	167	84	50
HCQ	207	119	71	32	20
Azith	317	165	72	30	22
Doxy	167	78	32	16	10

Recovery Inferences

	Pr(Superiority)	Pr(Meaningful Effect)	Pr(Best)	Randomization Probability
SOC				0.33
HCQ	0.8727	0.1377	0	0
Azith	0.8553	0.0917	0.525	0.4
Doxy	0.5723	0.0587	0.225	0.27

Hospitalization Inferences

Pr(Superiority)	Pr(Meaningful Effect)
0.2815	0.0148
0.4105	0.0232
0.2388	0.0127

Hazard Ratio Estimates

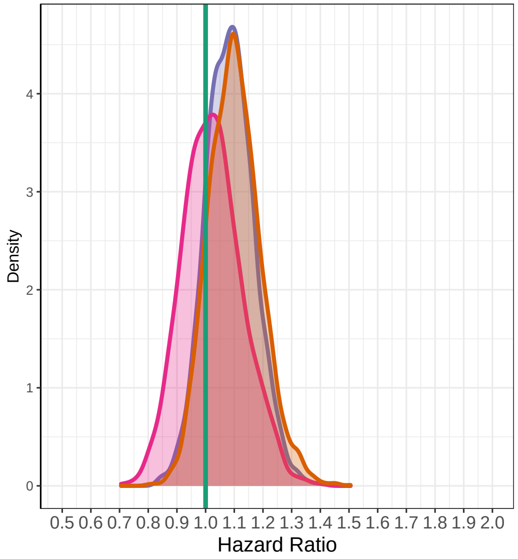

Median Time To Recovery Estimates

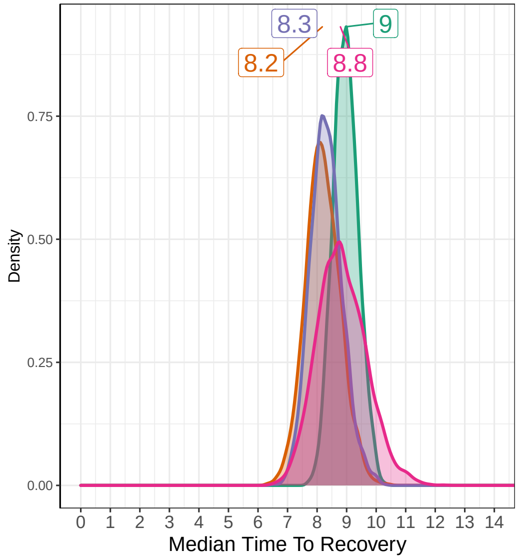

Interim 2 on November 12, 2020

Recovery Data

	Enrolled	Complete	Recovered	Exposure Days	Recoveries Per Day	Estimated Hazard	Estimated HR (95% interval)	Estimated Median Time to Recovery
SOC	605	574	522	6588	0.079	0.076	1	9.04
HCQ	207	207	188	2127	0.088	0.083	1.094 (0.932, 1.284)	8.3
Azith	417	392	362	3924	0.092	0.086	1.139 (1.001, 1.305)	7.98
Doxy	227	204	187	2176	0.086	0.08	1.065 (0.911, 1.25)	8.5
Total	1456	1377	1259	14815	0.085			

Hospitalization Data

Hospitalizations	28 Day Completers	Observed Hosp. Rate	Est. Hosp. Rate (95% interval)
18	519	0.0347	0.0311 (0.0189, 0.0462)
7	207	0.0338	0.0382 (0.0162, 0.0686)
12	317	0.0379	0.0309 (0.0164, 0.0497)
9	167	0.0539	0.0436 (0.0213, 0.0734)
46	1210	0.0380	

Intervention Status
Enrolling
Dropped
Enrolling
Enrolling

Observed Patient Recoveries

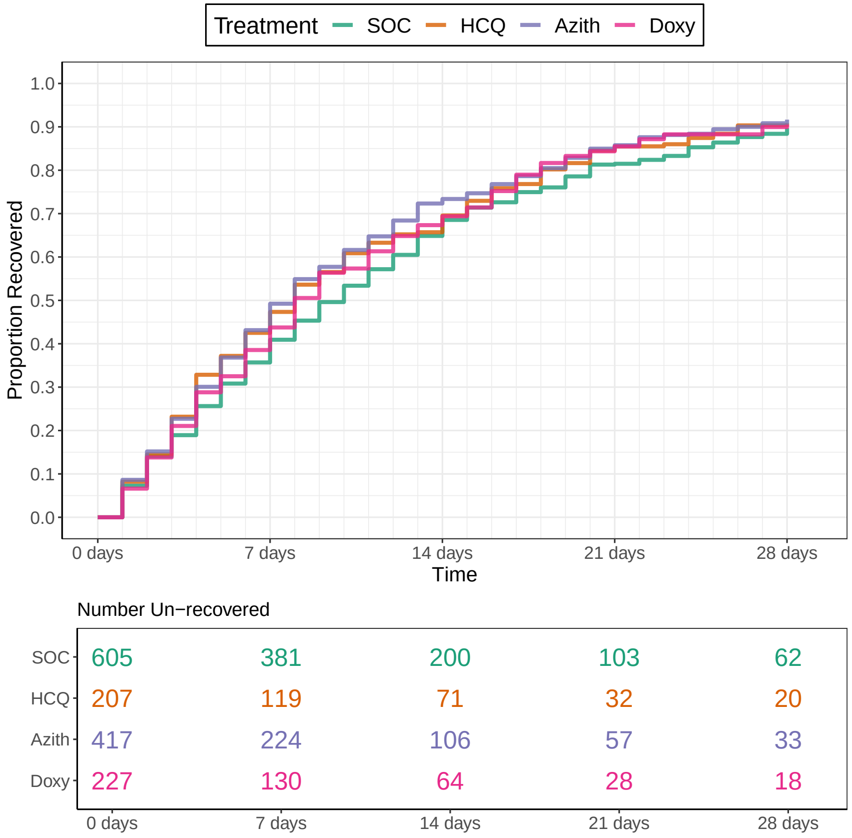

Recovery Inferences

	Pr(Superiority)	Pr(Meaningful Effect)	Pr(Best)	Randomization Probability
SOC				0.33
HCQ	0.8647	0.133	0	0
Azith	0.9757	0.22	0.5913	0.37
Doxy	0.7733	0.077	0.1588	0.29

Hospitalization Inferences

Pr(Superiority)	Pr(Meaningful Effect)
0.3355	0.018
0.5128	0.029
0.205	0.0102

Hazard Ratio Estimates

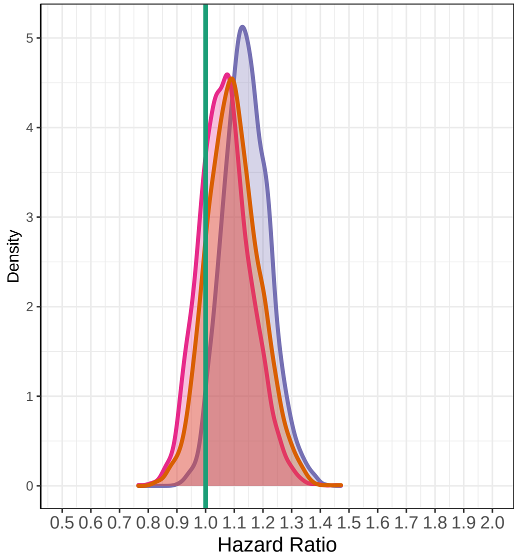

Median Time To Recovery Estimates

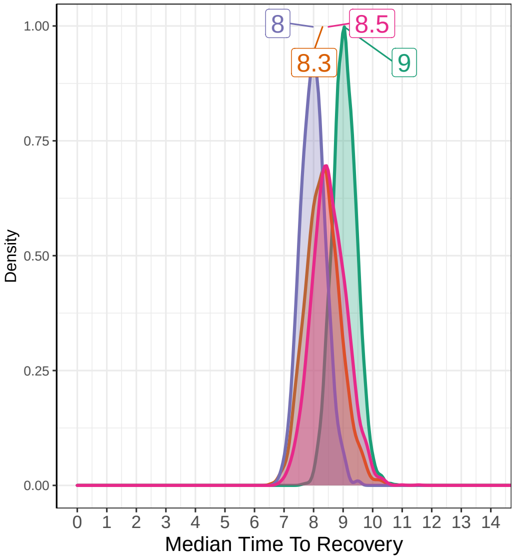

Interim 3 on December 10, 2020

Recovery Data

	Enrolled	Complete	Recovered	Exposure Days	Recoveries Per Day	Estimated Hazard	Estimated HR (95% interval)	Estimated Median Time to Recovery
SOC	668	636	573	7328	0.078	0.075	1	9.14
HCQ	207	207	188	2127	0.088	0.083	1.107 (0.945, 1.296)	8.29
Azith	472	453	419	4606	0.091	0.086	1.136 (1.01, 1.286)	8.07
Doxy	289	264	245	2946	0.083	0.079	1.042 (0.904, 1.204)	8.76
Total	1636	1560	1425	17008	0.084			

Hospitalization Data

Hospitalizations	28 Day Completers	Observed Hosp. Rate	Est. Hosp. Rate (95% interval)
22	605	0.0364	0.0344 (0.0222, 0.0488)
7	207	0.0338	0.0388 (0.0169, 0.0687)
15	417	0.0360	0.0337 (0.0194, 0.0518)
9	227	0.0396	0.0341 (0.0168, 0.0574)
53	1456	0.0364	

Intervention Status
Enrolling
Dropped
Enrolling
Futility

Observed Patient Recoveries

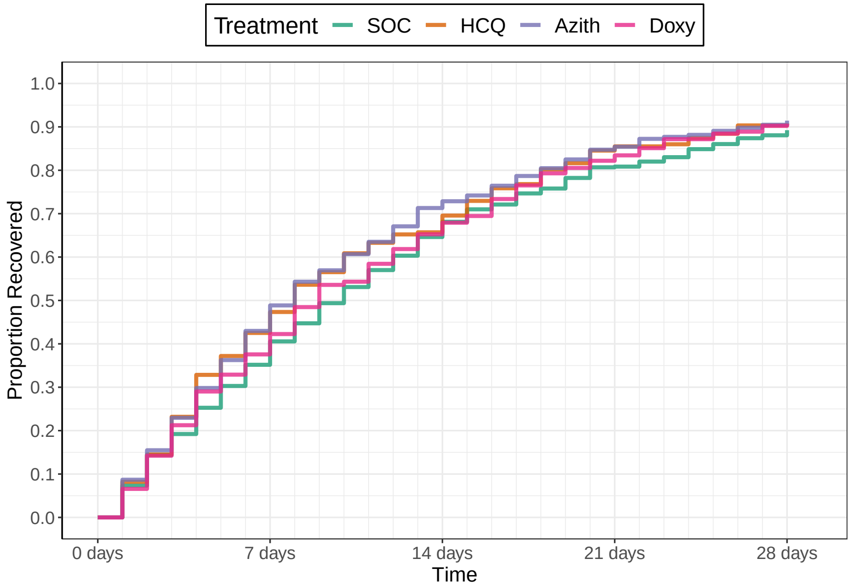

Number Un−recovered

	0 days	7 days	14 days	21 days	28 days
SOC	668	421	222	118	71
HCQ	207	119	71	32	20
Azith	472	262	129	68	40
Doxy	289	173	91	43	21

Recovery Inferences

	Pr(Superiority)	Pr(Meaningful Effect)	Pr(Best)	Randomization Probability
SOC				0.5
HCQ	0.8873	0.153	0	0
Azith	0.9803	0.2037	0.75	0.5
Doxy	0.7243	0.034	0	0

Hospitalization Inferences

Pr(Superiority)	Pr(Meaningful Effect)
0.4158	0.04
0.5435	0.0325
0.532	0.0452

Hazard Ratio Estimates

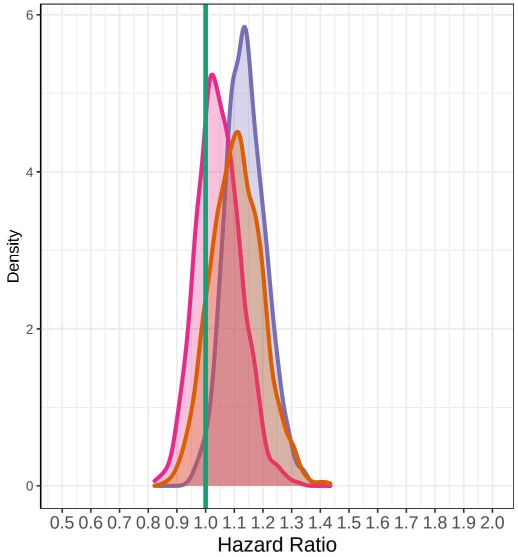

Median Time To Recovery Estimates

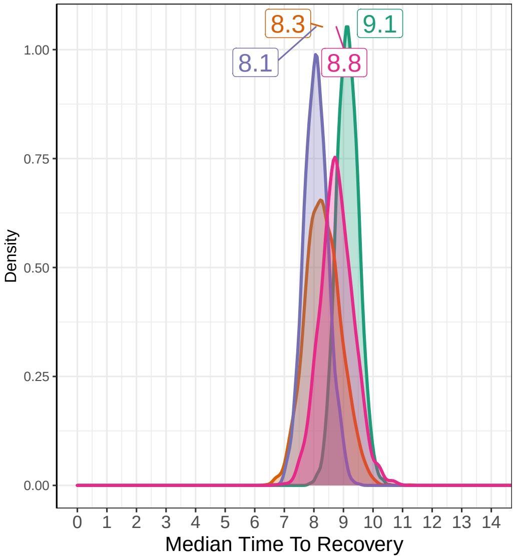

Interim 4 on January 07, 2021

Recovery Data

	Enrolled	Complete	Recovered	Exposure Days	Recoveries Per Day	Estimated Hazard	Estimated HR (95% interval)	Estimated Median Time to Recovery
SOC	770	724	650	8446	0.077	0.074	1	9.27
HCQ	207	207	188	2127	0.088	0.083	1.123 (0.964, 1.31)	8.27
Azith	583	540	497	5606	0.089	0.083	1.131 (1.008, 1.266)	8.23
Doxy	289	289	263	3158	0.083	0.079	1.069 (0.932, 1.219)	8.67
Total	1849	1760	1598	19337	0.083			

Hospitalization Data

Hospitalizations	28 Day Completers	Observed Hosp. Rate	Est. Hosp. Rate (95% interval)
25	668	0.0374	0.0337 (0.0221, 0.047)
7	207	0.0338	0.0384 (0.0173, 0.0679)
17	472	0.0360	0.0306 (0.0184, 0.046)
9	289	0.0311	0.0344 (0.0162, 0.0593)
58	1636	0.0355	

Intervention Status
Enrolling
Dropped
Enrolling
Dropped

Observed Patient Recoveries

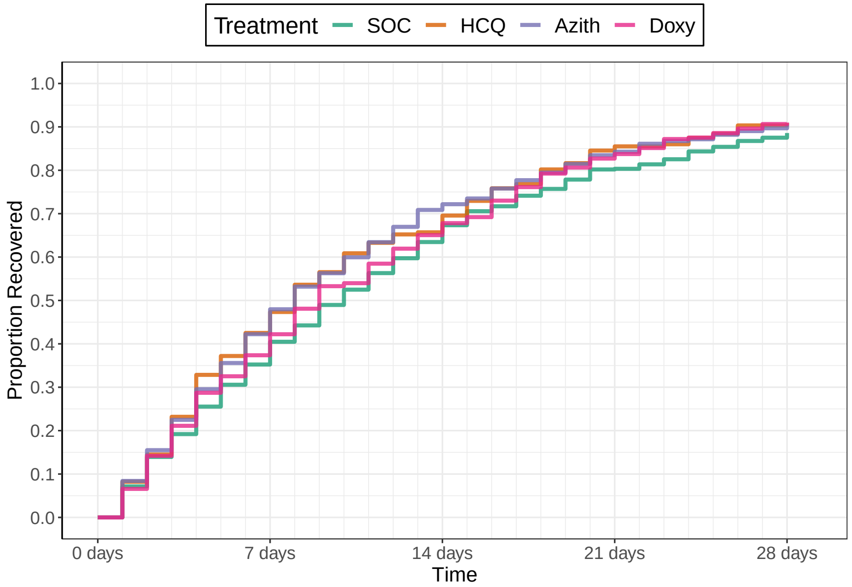

Number Un−recovered

	0 days	7 days	14 days	21 days	28 days
SOC	770	482	264	135	81
HCQ	207	119	71	32	20
Azith	583	322	155	82	48
Doxy	289	181	101	50	27

Recovery Inferences

	Pr(Superiority)	Pr(Meaningful Effect)	Pr(Best)	Randomization Probability
SOC				0.5
HCQ	0.9317	0.218	0	0
Azith	0.982	0.1753	0.75	0.5
Doxy	0.8383	0.0533	0	0

Hospitalization Inferences

Pr(Superiority)	Pr(Meaningful Effect)
0.4008	0.032
0.6305	0.0352
0.51	0.0415

Hazard Ratio Estimates

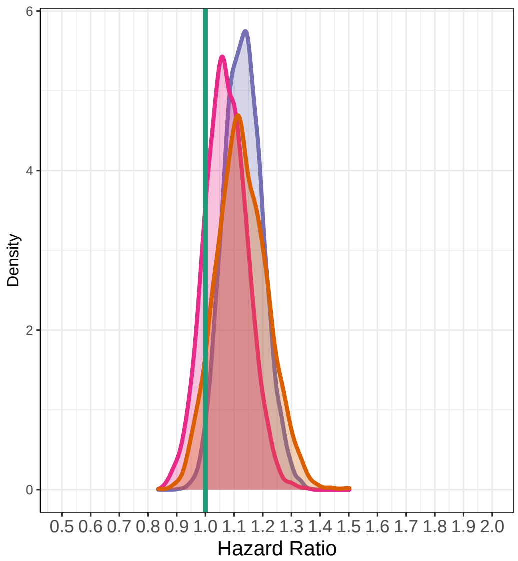

Median Time To Recovery Estimates

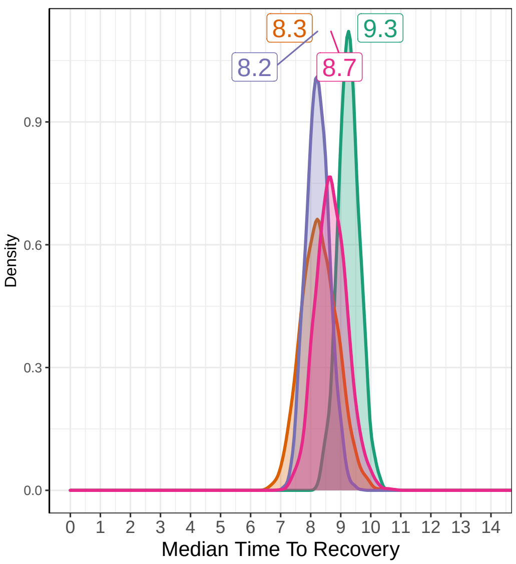

Interim 5 on February 04, 2021

Recovery Data

	Enrolled	Complete	Recovered	Exposure Days	Recoveries Per Day	Estimated Hazard	Estimated HR (95% interval)	Estimated Median Time to Recovery
SOC	881	832	747	9689	0.077	0.074	1	9.31
HCQ	207	207	188	2127	0.088	0.084	1.124 (0.958, 1.325)	8.31
Azith	682	643	590	6725	0.088	0.083	1.122 (1.005, 1.246)	8.34
Doxy	289	289	263	3158	0.083	0.079	1.069 (0.932, 1.219)	8.73
Total	2059	1971	1788	21700	0.082			

Hospitalization Data

Hospitalizations	28 Day Completers	Observed Hosp. Rate	Est. Hosp. Rate (95% interval)
28	770	0.0364	0.033 (0.0223, 0.0459)
7	207	0.0338	0.0383 (0.0167, 0.0678)
20	583	0.0343	0.0306 (0.019, 0.0447)
9	289	0.0311	0.0346 (0.017, 0.0578)
64	1849	0.0346	

Intervention Status
Enrolling
Dropped
Enrolling
Dropped

Observed Patient Recoveries

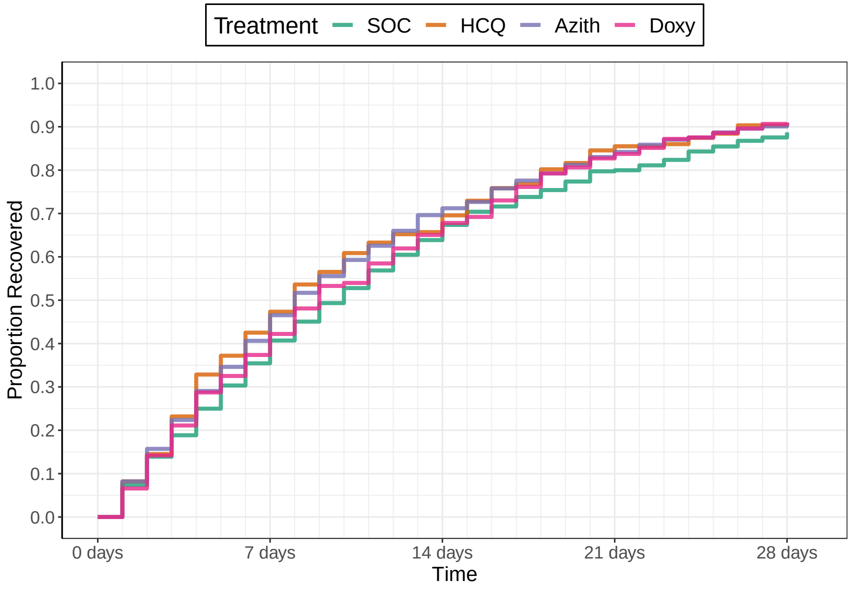

Number Un−recovered

	0 days	7 days	14 days	21 days	28 days
SOC	881	552	299	163	95
HCQ	207	119	71	32	20
Azith	682	392	192	102	57
Doxy	289	181	101	50	27

Recovery Inferences

	Pr(Superiority)	Pr(Meaningful Effect)	Pr(Best)	Randomization Probability
SOC				0.5
HCQ	0.928	0.2117	0	0
Azith	0.9803	0.125	0.75	0.5
Doxy	0.826	0.054	0	0

Hospitalization Inferences

Pr(Superiority)	Pr(Meaningful Effect)
0.38	0.023
0.607	0.0235
0.4732	0.025

Hazard Ratio Estimates

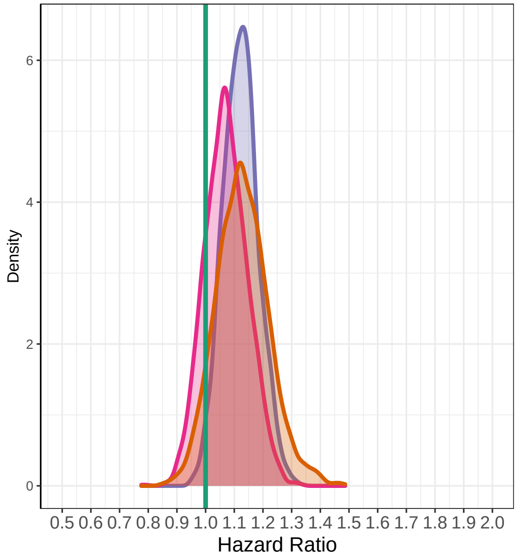

Median Time To Recovery Estimates

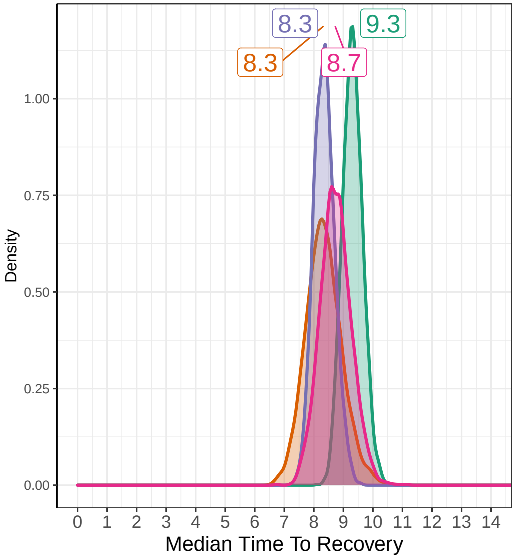

Interim 6 on March 04, 2021

Recovery Data

	Enrolled	Complete	Recovered	Exposure Days	Recoveries Per Day	Estimated Hazard	Estimated HR (95% interval)	Estimated Median Time to Recovery
SOC	1010	955	861	11070	0.078	0.075	1	9.3
HCQ	207	207	188	2127	0.088	0.084	1.113 (0.957, 1.299)	8.38
Azith	782	737	677	7695	0.088	0.084	1.115 (1.019, 1.232)	8.38
Doxy	289	289	263	3158	0.083	0.08	1.062 (0.926, 1.22)	8.78
Total	2288	2188	1989	24050	0.083			

Hospitalization Data

Hospitalizations	28 Day Completers	Observed Hosp. Rate	Est. Hosp. Rate (95% interval)
31	881	0.0352	0.0314 (0.0215, 0.0431)
7	207	0.0338	0.0384 (0.0174, 0.0679)
27	682	0.0396	0.0356 (0.0239, 0.0497)
9	289	0.0311	0.0341 (0.0164, 0.0578)
74	2059	0.0359	

Intervention Status
Enrolling
Dropped
Enrolling
Dropped

Observed Patient Recoveries

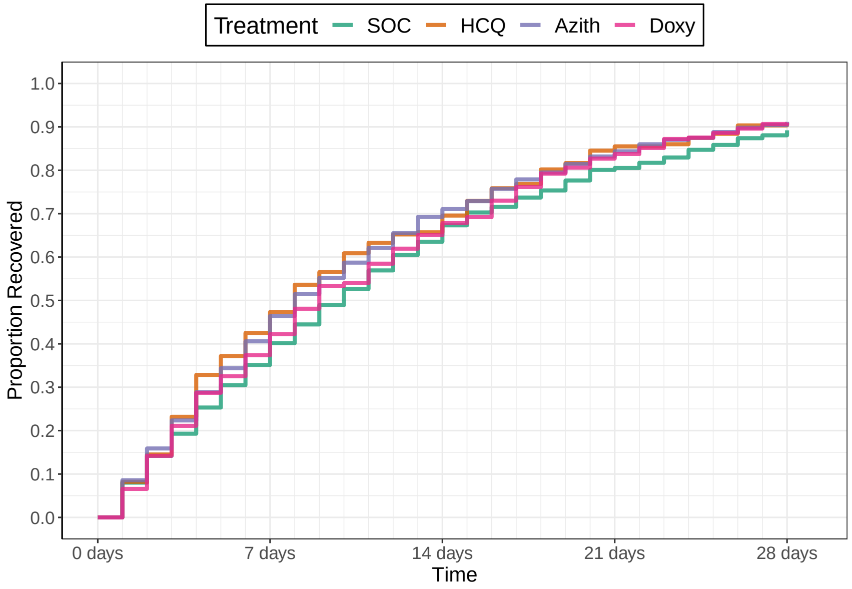

Number Un−recovered

	0 days	7 days	14 days	21 days	28 days
SOC	1010	635	347	181	104
HCQ	207	119	71	32	20
Azith	782	448	221	114	65
Doxy	289	181	101	50	27

Recovery Inferences

	Pr(Superiority)	Pr(Meaningful Effect)	Pr(Best)	Randomization Probability
SOC				0.5
HCQ	0.9197	0.1733	0	0
Azith	0.9887	0.077	0.75	0.5
Doxy	0.8143	0.0377	0	0

Hospitalization Inferences

Pr(Superiority)	Pr(Meaningful Effect)
0.331	0.0162
0.3137	0.0028
0.4255	0.0168

Hazard Ratio Estimates

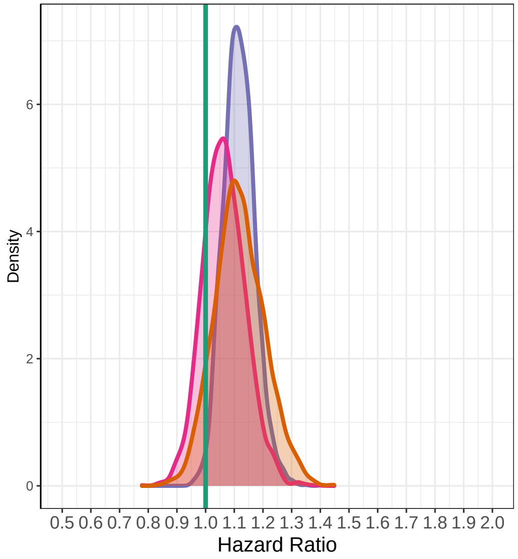

Median Time To Recovery Estimates

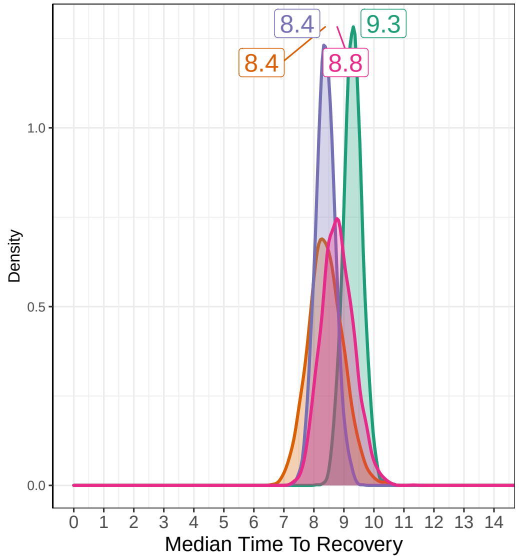

Interim 7 on April 01, 2021

Recovery Data

	Enrolled	Complete	Recovered	Exposure Days	Recoveries Per Day	Estimated Hazard	Estimated HR (95% interval)	Estimated Median Time to Recovery
SOC	1130	1085	975	12506	0.078	0.076	1	9.29
HCQ	207	207	188	2127	0.088	0.085	1.114 (0.96, 1.287)	8.38
Azith	884	848	784	8906	0.088	0.085	1.115 (1.015, 1.217)	8.37
Doxy	289	289	263	3158	0.083	0.081	1.059 (0.924, 1.206)	8.8
Total	2510	2429	2210	26697	0.083			

Hospitalization Data

Hospitalizations	28 Day Completers	Observed Hosp. Rate	Est. Hosp. Rate (95% interval)
33	1010	0.0327	0.03 (0.0209, 0.0405)
7	207	0.0338	0.0382 (0.0164, 0.0674)
31	782	0.0396	0.0361 (0.0249, 0.0494)
9	289	0.0311	0.0342 (0.0163, 0.0577)
80	2288	0.0350	

Intervention Status
Enrolling
Dropped
Enrolling
Dropped

Observed Patient Recoveries

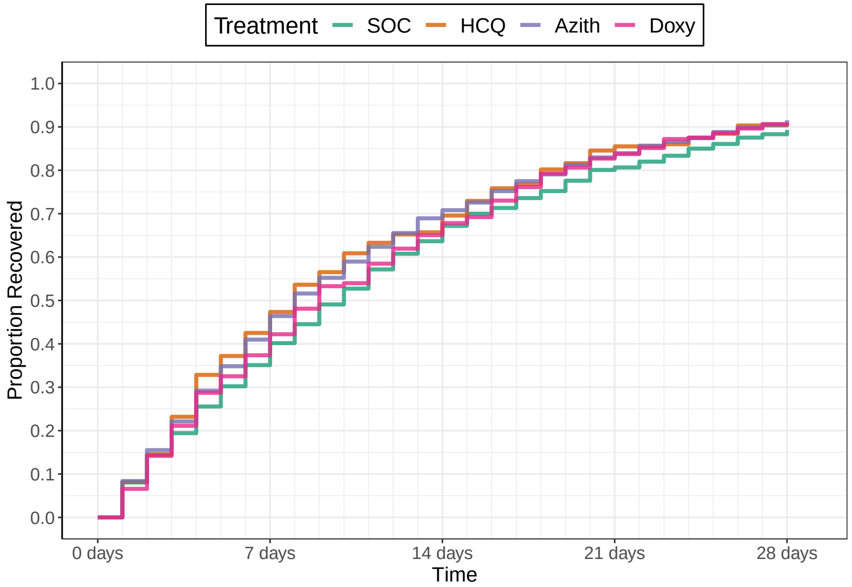

Number Un−recovered

	0 days	7 days	14 days	21 days	28 days
SOC	1130	717	390	209	120
HCQ	207	119	71	32	20
Azith	884	511	265	139	73
Doxy	289	181	101	50	27

Recovery Inferences

	Pr(Superiority)	Pr(Meaningful Effect)	Pr(Best)	Randomization Probability
SOC				0.5
HCQ	0.927	0.1657	0	0
Azith	0.9893	0.0693	0.75	0.5
Doxy	0.803	0.036	0	0

Hospitalization Inferences

Pr(Superiority)	Pr(Meaningful Effect)
0.3045	0.0082
0.2268	8e−04
0.3902	0.0092

Hazard Ratio Estimates

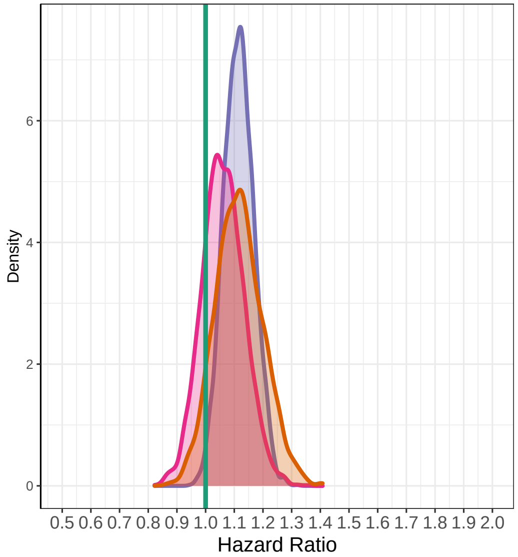

Median Time To Recovery Estimates

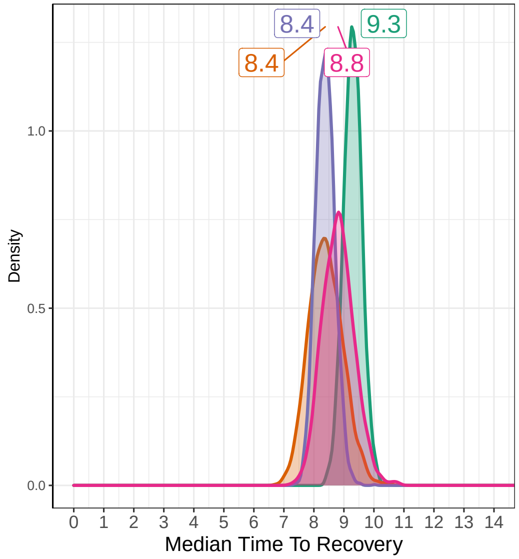

Interim 8 on April 29, 2021

Recovery Data

	Enrolled	Complete	Recovered	Exposure Days	Recoveries Per Day	Estimated Hazard	Estimated HR (95% interval)	Estimated Median Time to Recovery
SOC	1228	1194	1073	13572	0.079	0.076	1	9.12
HCQ	207	207	188	2127	0.088	0.084	1.1 (0.944, 1.283)	8.33
Azith	996	954	876	9989	0.088	0.084	1.097 (1.008, 1.198)	8.35
Doxy	289	289	263	3158	0.083	0.08	1.049 (0.921, 1.187)	8.72
Total	2720	2644	2400	28845	0.083			

Hospitalization Data

Hospitalizations	28 Day Completers	Observed Hosp. Rate	Est. Hosp. Rate (95% interval)
34	1130	0.0301	0.0285 (0.0199, 0.0385)
7	207	0.0338	0.0382 (0.0166, 0.0675)
35	884	0.0396	0.0361 (0.0254, 0.0481)
9	289	0.0311	0.0344 (0.0167, 0.0579)
85	2510	0.0339	

Intervention Status
Enrolling
Dropped
Futility
Dropped

Observed Patient Recoveries

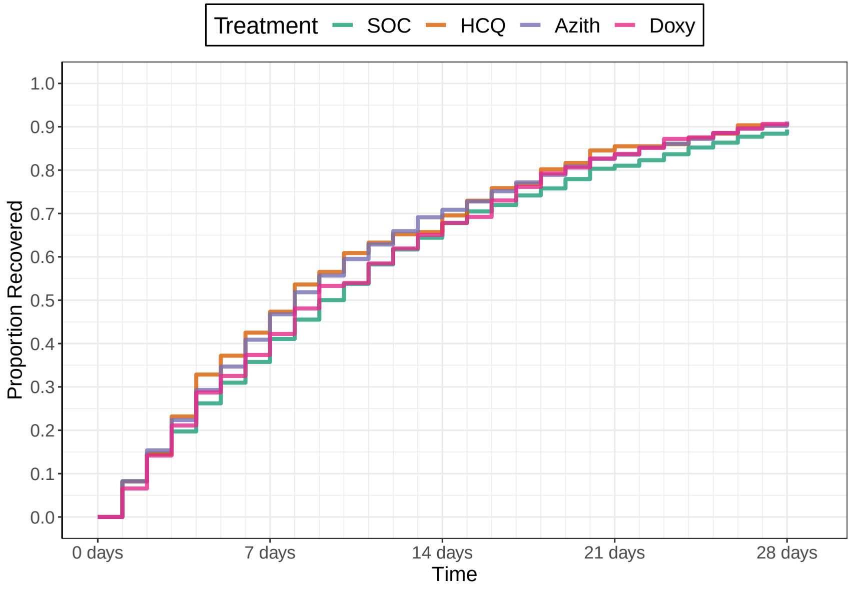

Number Un−recovered

	0 days	7 days	14 days	21 days	28 days
SOC	1228	777	421	230	133
HCQ	207	119	71	32	20
Azith	996	573	288	159	87
Doxy	289	181	101	50	27

Recovery Inferences

	Pr(Superiority)	Pr(Meaningful Effect)	Pr(Best)	Randomization Probability
SOC				1
HCQ	0.8877	0.1253	0	0
Azith	0.9853	0.027	0	0
Doxy	0.759	0.019	0	0

Hospitalization Inferences

Pr(Superiority)	Pr(Meaningful Effect)
0.254	0.0068
0.1568	0
0.3295	0.006

Hazard Ratio Estimates

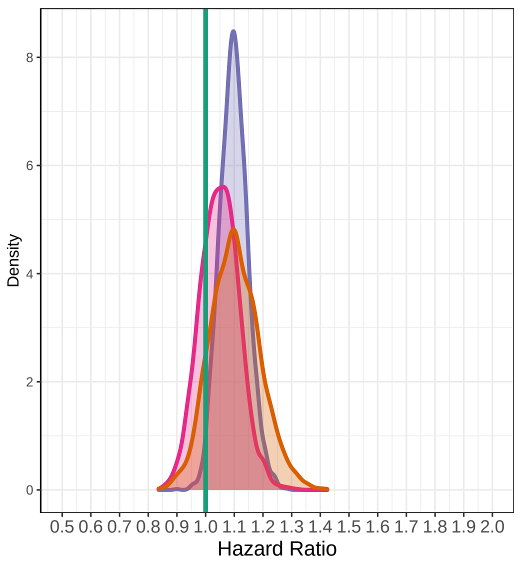

Median Time To Recovery Estimates

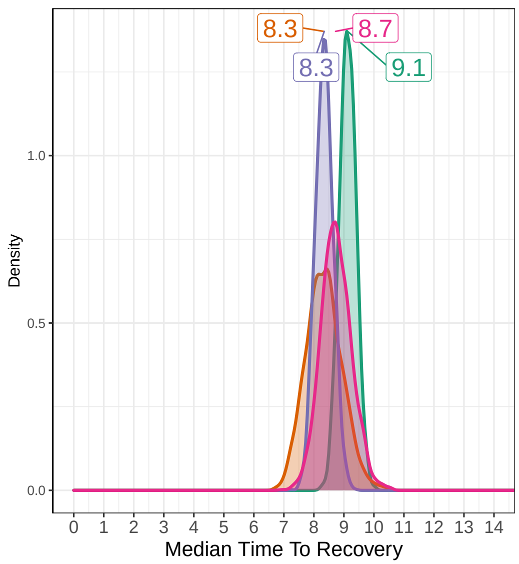

Interim 9 on August 26, 2022

Recovery Data

	Enrolled	Complete	Recovered	Exposure Days	Recoveries Per Day	Estimated Hazard	Estimated HR (95% interval)	Estimated Median Time to Recovery
SOC	4508	4508	3979	52829	0.075	0.073	1	9.64
HCQ	207	207	188	2127	0.088	0.085	1.154 (0.995, 1.322)	8.39
Azith	996	996	908	10382	0.087	0.084	1.153 (1.071, 1.235)	8.4
Doxy	289	289	263	3158	0.083	0.081	1.098 (0.97, 1.243)	8.79
Total	6000	6000	5338	68497	0.078			

Hospitalization Data

	Hospitalizations	28 Day Completers	Observed Hosp. Rate	Est. Hosp. Rate (95% interval)
SOC	138	4508	0.0306	0.0308 (0.026, 0.0361)
HCQ	7	207	0.0338	0.0387 (0.017, 0.0686)
Azith	35	996	0.0351	0.0361 (0.0253, 0.0486)
Doxy	9	289	0.0311	0.0344 (0.0167, 0.0584)
Total	189	6000	0.0315	

	Intervention Status
SOC	Complete
HCQ	Dropped
Azith	Dropped
Doxy	Dropped
Total	

Observed Patient Recoveries

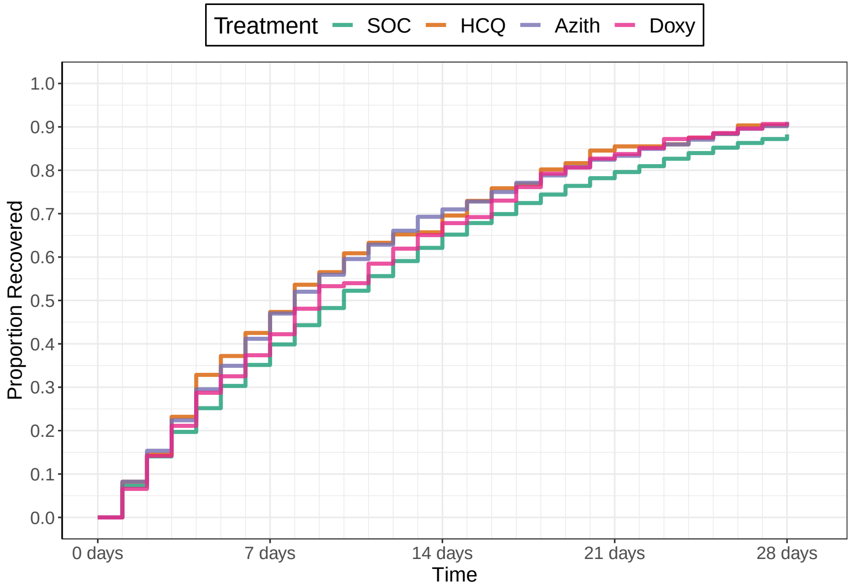

Number Un−recovered

	0 days	7 days	14 days	21 days	28 days
SOC	4508	2924	1708	984	577
HCQ	207	119	71	32	20
Azith	996	586	306	175	98
Doxy	289	181	101	50	27

Recovery Inferences

	Pr(Superiority)	Pr(Meaningful Effect)	Pr(Best)	Randomization Probability
SOC				
HCQ	0.9697	0.3407	0	
Azith	1	0.1867	0	
Doxy	0.928	0.1067	0	

Hospitalization Inferences

	Pr(Superiority)	Pr(Meaningful Effect)
SOC		
HCQ	0.3088	0.005
Azith	0.2218	0
Doxy	0.4058	0.0032

Hazard Ratio Estimates

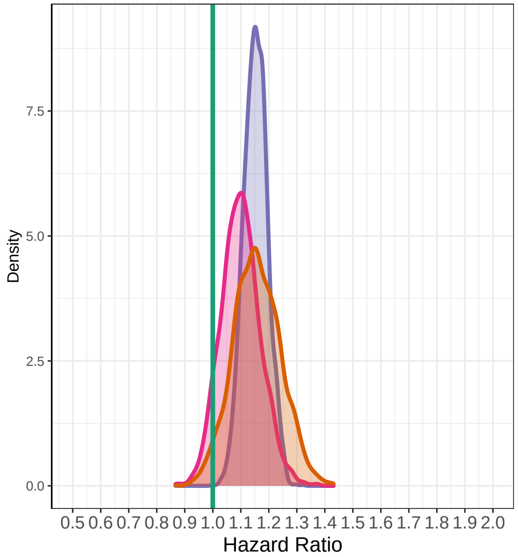

Median Time To Recovery Estimates

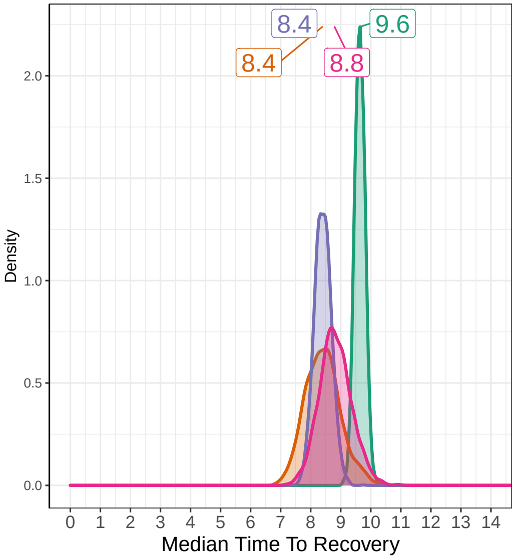

6.5 Example 4

April 1, 2020

Before Patient Enrollment Begins

Recovery Data

	Enrolled	Complete	Recovered	Exposure Days	Recoveries Per Day	Estimated Hazard	Estimated HR (95% interval)	Estimated Median Survival Time
SOC	0	0	0	0				
HCQ	0	0	0	0				
Azith	0	0	0	0				
Doxy	0	0	0	0				
Total	0	0	0	0				

Hospitalization Data

Hospitalizations	28 Day Completers	Observed Hosp. Rate	Est. Hosp. Rate (95% interval)
0	0		
0	0		
0	0		
0	0		
0	0		

Intervention Status
Enrolling
Enrolling
Unopened
Unopened

Observed Patient Recoveries

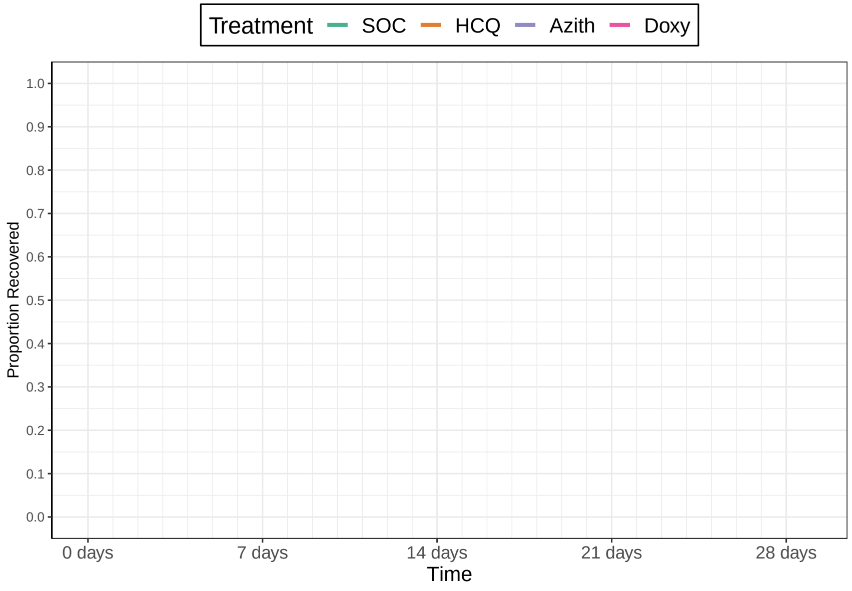

Number Un−recovered

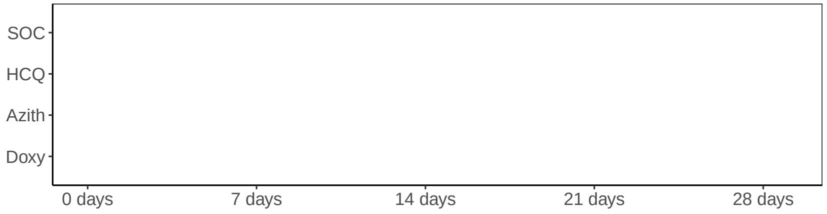

Recovery Inferences

	Pr(Superiority)	Pr(Meaningful Effect)	Pr(Best)	Randomization Probability
SOC				0.5
HCQ				0.5
Azith				0
Doxy				0

Hospitalization Inferences

Pr(Superiority)	Pr(Meaningful Effect)
0	0
0	0
0	0
0	0

Hazard Ratio Estimates

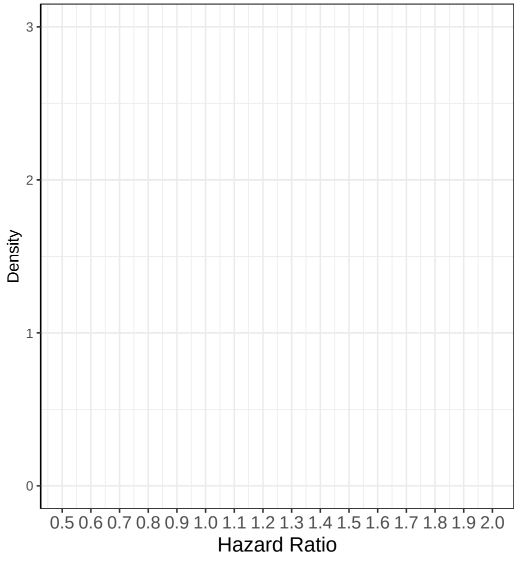

Median Time To Recovery Estimates

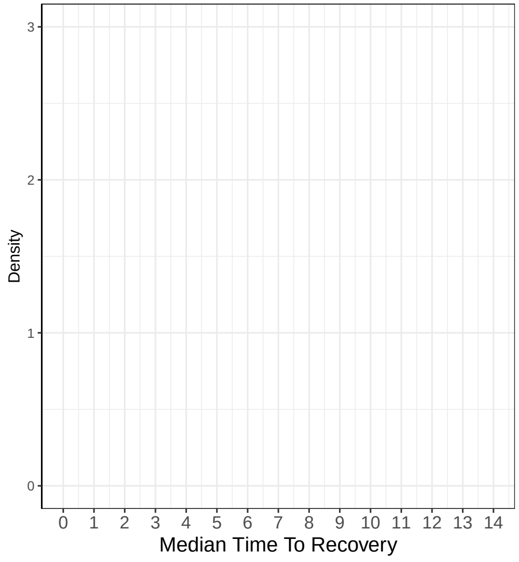

Remove Drug on May 22, 2020

Recovery Data

	Enrolled	Complete	Recovered	Exposure Days	Recoveries Per Day	Estimated Hazard	Estimated HR (95% interval)	Estimated Median Time to Recovery
SOC	206	152	146	1728	0.084			
HCQ	207	143	134	1821	0.074			
Azith	0	0	0	0				
Doxy	0	0	0	0				
Total	413	295	280	3549	0.079			

Hospitalization Data

Hospitalizations	28 Day Completers	Observed Hosp. Rate	Est. Hosp. Rate (95% interval)
4	79	0.0506	
5	78	0.0641	
0	0	NA	
0	0	NA	
9	157	0.0573	

Intervention Status
Enrolling
Removing
Unopened
Unopened

Observed Patient Recoveries

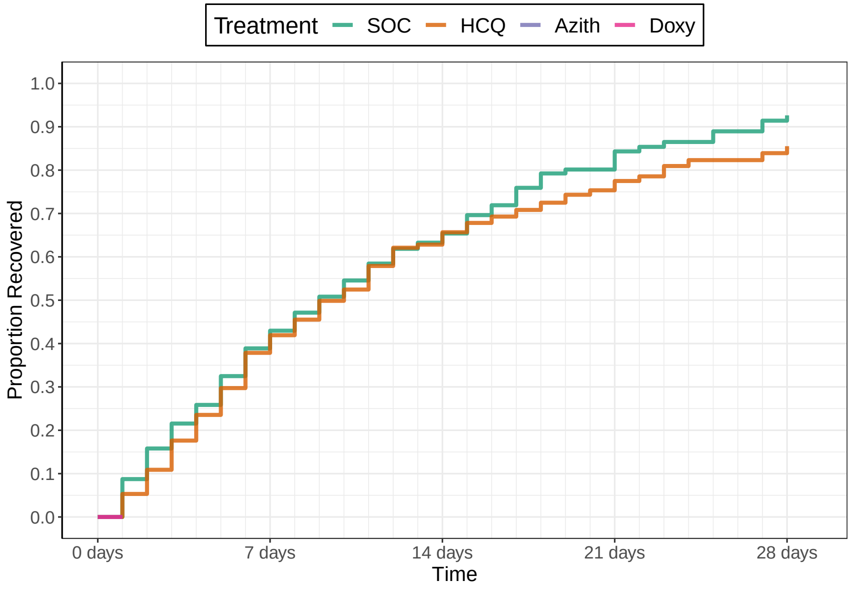

Number Un−recovered

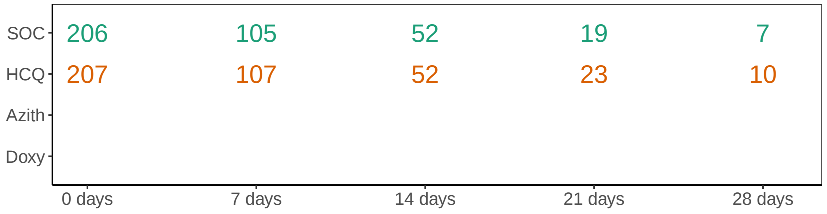

Recovery Inferences

	Pr(Superiority)	Pr(Meaningful Effect)	Pr(Best)	Randomization Probability
SOC				1
HCQ			0	0
Azith			0	0
Doxy			0	0

Hospitalization Inferences

Pr(Superiority)	Pr(Meaningful Effect)

Hazard Ratio Estimates

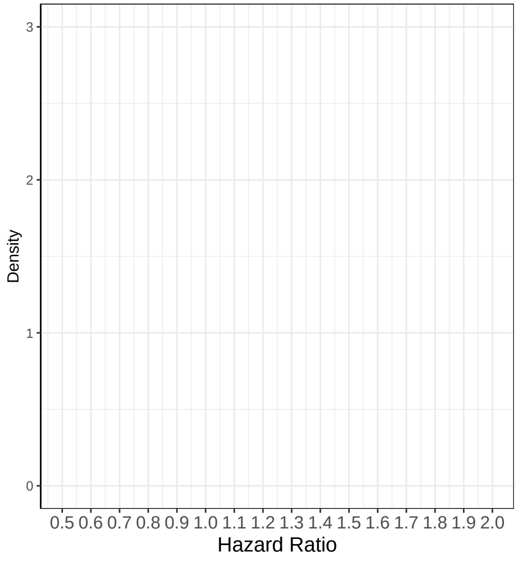

Median Time To Recovery Estimates

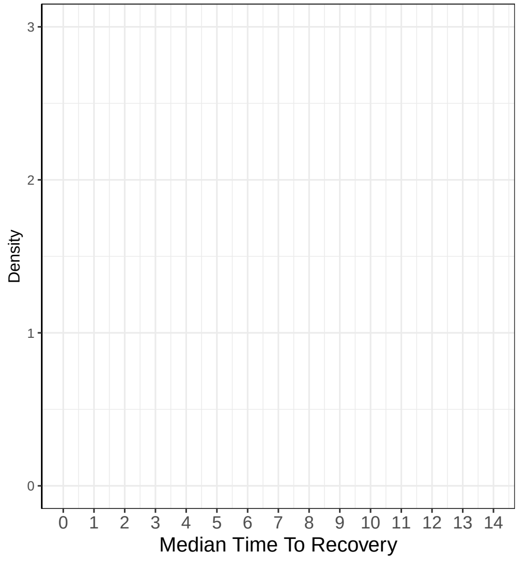

Add Drug on May 23, 2020

Recovery Data

	Enrolled	Complete	Recovered	Exposure Days	Recoveries Per Day	Estimated Hazard	Estimated HR (95% interval)	Estimated Median Time to Recovery
SOC	208	155	149	1782	0.084			
HCQ	207	147	138	1884	0.073			
Azith	0	0	0	0				
Doxy	0	0	0	0				
Total	415	302	287	3666	0.078			

Hospitalization Data

Hospitalizations	28 Day Completers	Observed Hosp. Rate	Est. Hosp. Rate (95% interval)
4	80	0.0500	
5	80	0.0625	
0	0	NA	
0	0	NA	
9	160	0.0562	

Intervention Status
Enrolling
Paused
Introducing
Unopened

Observed Patient Recoveries

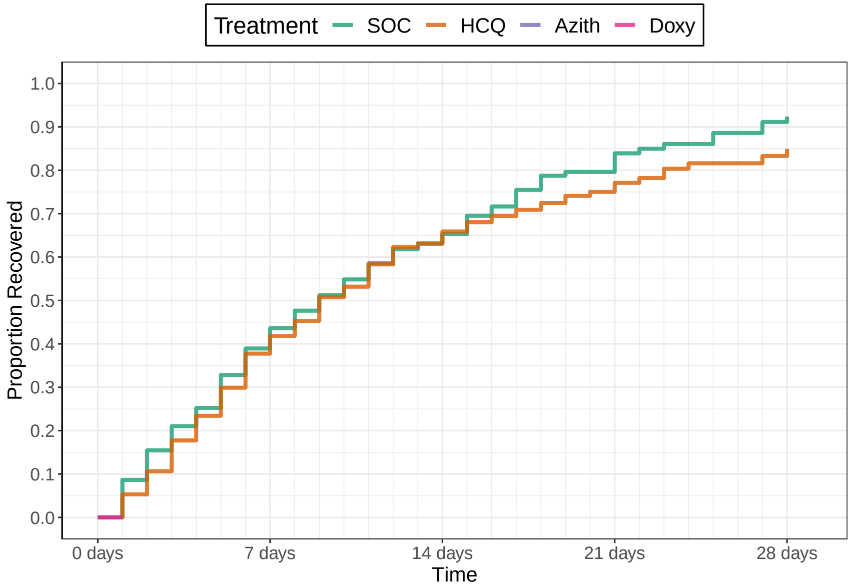

Number Un−recovered

	0 days	7 days	14 days	21 days	28 days
SOC	208	105	52	19	7
HCQ	207	107	52	24	10
Azith					
Doxy					

Recovery Inferences

	Pr(Superiority)	Pr(Meaningful Effect)	Pr(Best)	Randomization Probability
SOC				0.5
HCQ			0	0
Azith			1	0.5
Doxy			0	0

Hospitalization Inferences

Pr(Superiority)	Pr(Meaningful Effect)

Hazard Ratio Estimates

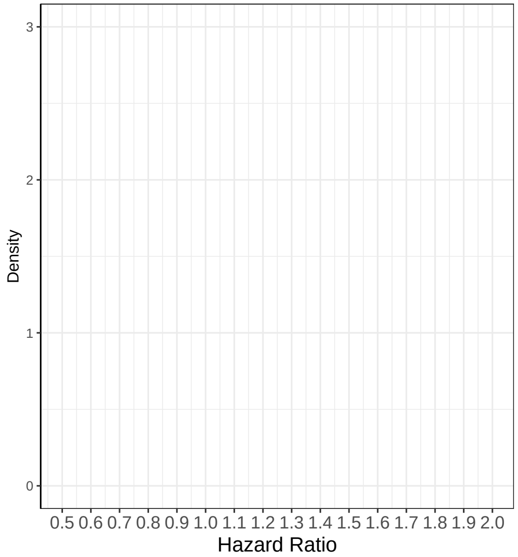

Median Time To Recovery Estimates

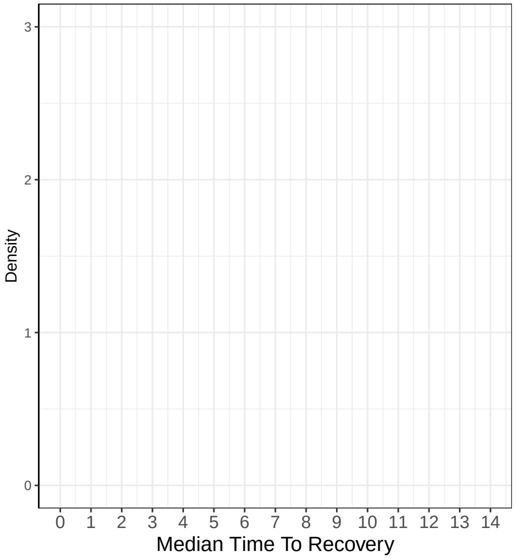

Add Drug on July 23, 2020

Recovery Data

	Enrolled	Complete	Recovered	Exposure Days	Recoveries Per Day	Estimated Hazard	Estimated HR (95% interval)	Estimated Median Time to Recovery
SOC	326	310	286	3499	0.082			
HCQ	207	207	179	2453	0.073			
Azith	118	104	100	1078	0.093			
Doxy	0	0	0	0				
Total	651	621	565	7030	0.08			

Hospitalization Data

Hospitalizations	28 Day Completers	Observed Hosp. Rate	Est. Hosp. Rate (95% interval)
7	285	0.0246	
5	207	0.0242	
4	77	0.0519	
0	0	NA	
16	569	0.0281	

Intervention Status
Enrolling
Paused
Introducing
Introducing

Observed Patient Recoveries

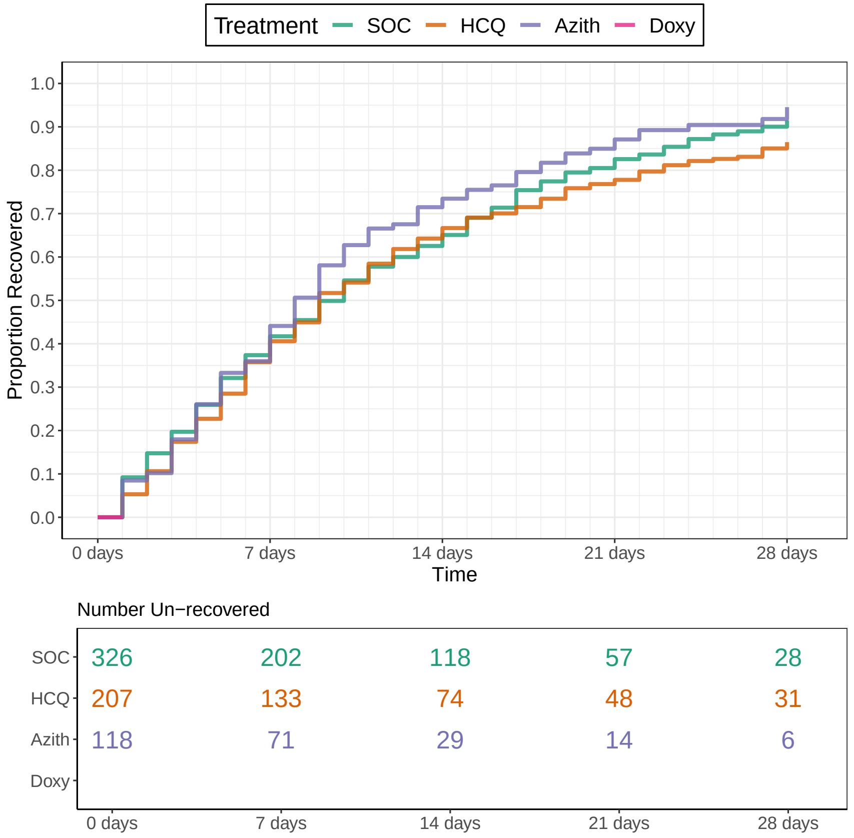

Recovery Inferences

	Pr(Superiority)	Pr(Meaningful Effect)	Pr(Best)	Randomization Probability
SOC				0.33
HCQ			0	0
Azith			1	0.33
Doxy			0	0.33

Hospitalization Inferences

Pr(Superiority)	Pr(Meaningful Effect)

Hazard Ratio Estimates

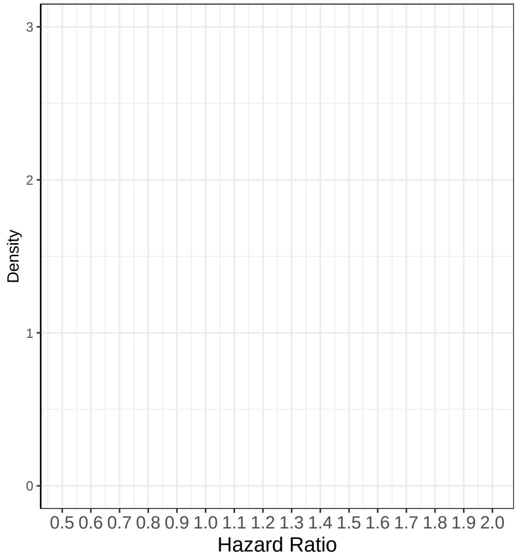

Median Time To Recovery Estimates

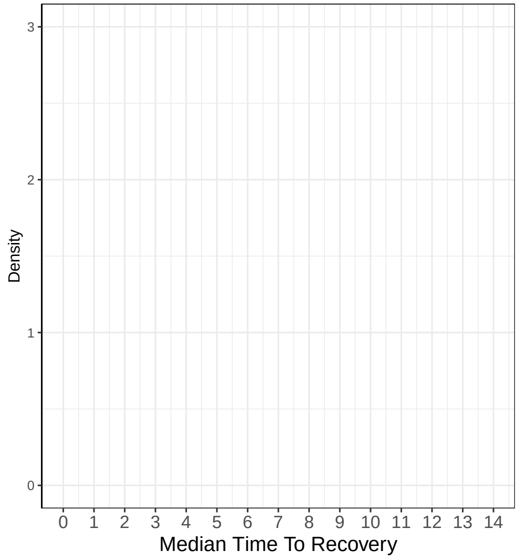

Interim 1 on October 15, 2020

Recovery Data

	Enrolled	Complete	Recovered	Exposure Days	Recoveries Per Day	Estimated Hazard	Estimated HR (95% interval)	Estimated Median Time to Recovery
SOC	482	440	405	4826	0.084	0.082	1	8.69
HCQ	207	207	179	2453	0.073	0.072	0.875 (0.742, 1.03)	9.88
Azith	312	270	260	2615	0.099	0.095	1.158 (1.004, 1.349)	7.53
Doxy	209	151	146	1571	0.093	0.088	1.08 (0.901, 1.302)	8.05
Total	1210	1068	990	11465	0.086			

Hospitalization Data

Hospitalizations	28 Day Completers	Observed Hosp. Rate	Est. Hosp. Rate (95% interval)
10	387	0.0258	0.0228 (0.0115, 0.0382)
5	207	0.0242	0.0286 (0.0108, 0.0541)
8	200	0.0400	0.0288 (0.0132, 0.05)
2	79	0.0253	0.0141 (0.003, 0.0346)
25	873	0.0286	

Intervention Status
Enrolling
Futility
Enrolling
Enrolling

Observed Patient Recoveries

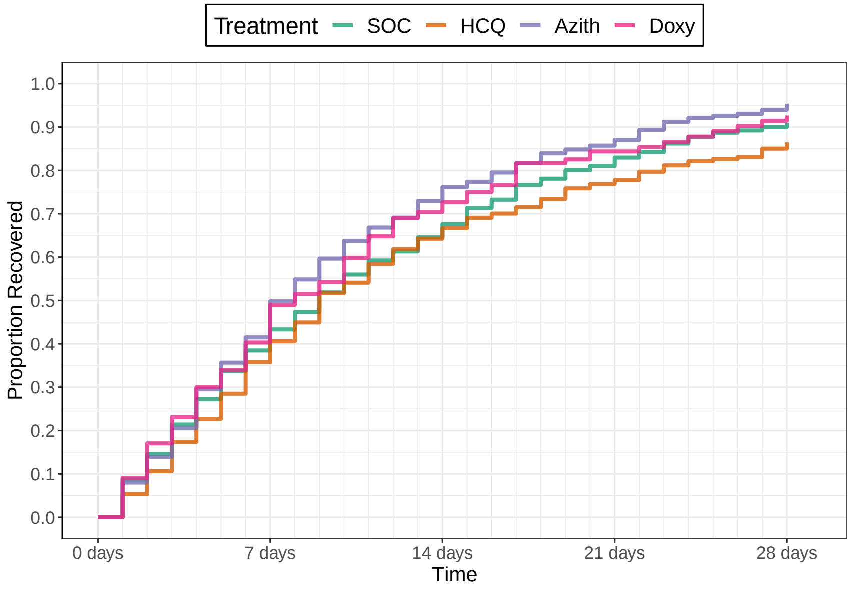

Number Un−recovered

	0 days	7 days	14 days	21 days	28 days
SOC	482	279	152	78	40
HCQ	207	133	74	48	31
Azith	312	162	68	32	13
Doxy	209	103	40	16	7

Recovery Inferences

	Pr(Superiority)	Pr(Meaningful Effect)	Pr(Best)	Randomization Probability
SOC				0.33
HCQ	0.0513	0	0	0
Azith	0.9793	0.28	0.567	0.37
Doxy	0.8027	0.116	0.183	0.3

Hospitalization Inferences

Pr(Superiority)	Pr(Meaningful Effect)
0.3352	0.0142
0.3022	0.0122
0.806	0.1338

Hazard Ratio Estimates

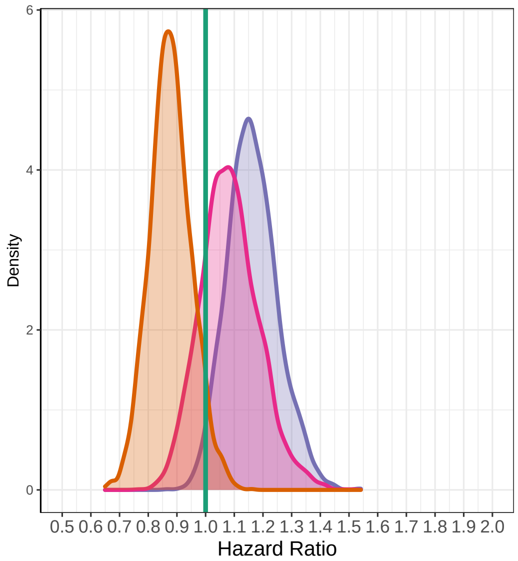

Median Time To Recovery Estimates

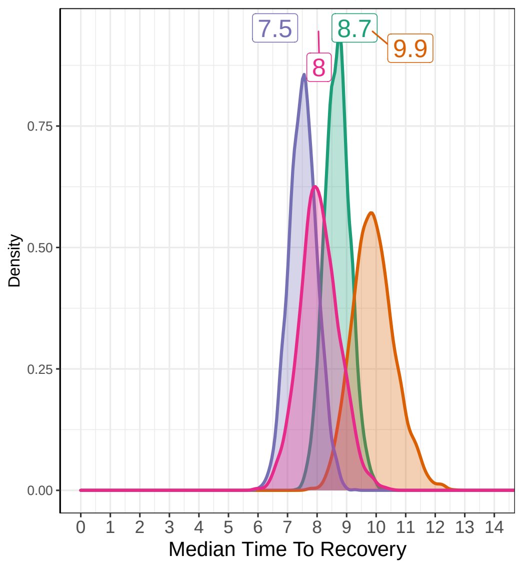

Interim 2 on November 12, 2020

Recovery Data

	Enrolled	Complete	Recovered	Exposure Days	Recoveries Per Day	Estimated Hazard	Estimated HR (95% interval)	Estimated Median Time to Recovery
SOC	573	535	488	5941	0.082	0.079	1	8.83
HCQ	207	207	179	2453	0.073	0.071	0.891 (0.756, 1.049)	9.85
Azith	405	373	354	3728	0.095	0.09	1.132 (0.989, 1.296)	7.85
Doxy	263	246	231	2421	0.095	0.09	1.134 (0.977, 1.32)	7.83
Total	1448	1361	1252	14543	0.086			

Hospitalization Data

Hospitalizations	28 Day Completers	Observed Hosp. Rate	Est. Hosp. Rate (95% interval)
15	482	0.0311	0.0279 (0.0162, 0.0424)
5	207	0.0242	0.0285 (0.0109, 0.054)
10	312	0.0321	0.0274 (0.014, 0.0455)
4	209	0.0191	0.019 (0.0062, 0.0387)
34	1210	0.0281	

Intervention Status
Enrolling
Dropped
Enrolling
Enrolling

Observed Patient Recoveries

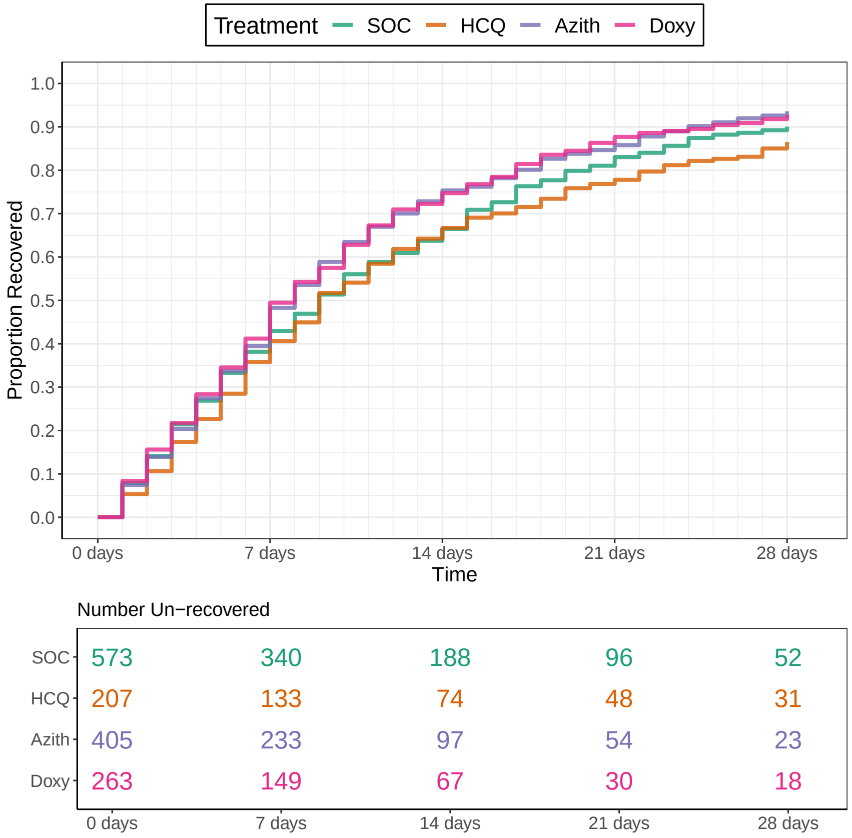

Recovery Inferences

	Pr(Superiority)	Pr(Meaningful Effect)	Pr(Best)	Randomization Probability
SOC				0.33
HCQ	0.0867	0	0	0
Azith	0.966	0.1797	0.3665	0.34
Doxy	0.944	0.1967	0.3835	0.33

Hospitalization Inferences

Pr(Superiority)	Pr(Meaningful Effect)
0.5152	0.042
0.5262	0.033
0.8028	0.1468

Hazard Ratio Estimates

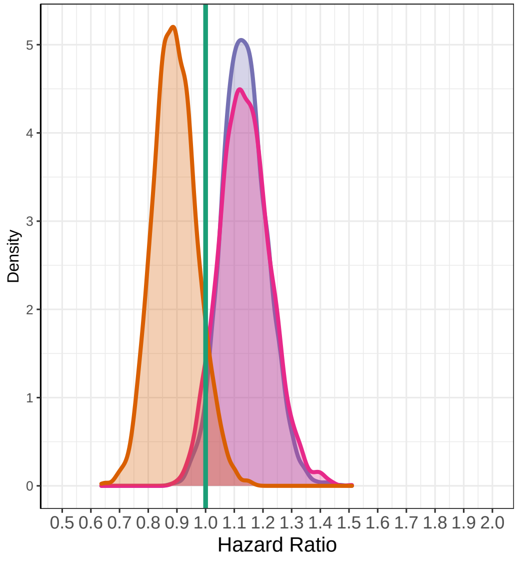

Median Time To Recovery Estimates

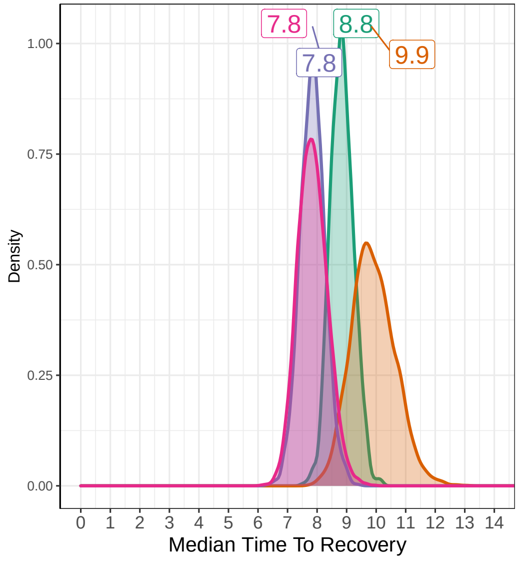

Interim 3 on December 10, 2020

Recovery Data

	Enrolled	Complete	Recovered	Exposure Days	Recoveries Per Day	Estimated Hazard	Estimated HR (95% interval)	Estimated Median Time to Recovery
SOC	638	615	559	6714	0.083	0.08	1	8.77
HCQ	207	207	179	2453	0.073	0.071	0.884 (0.749, 1.036)	9.85
Azith	470	452	422	4524	0.093	0.089	1.107 (0.983, 1.254)	7.99
Doxy	334	307	290	3140	0.092	0.088	1.093 (0.954, 1.259)	8.07
Total	1649	1581	1450	16831	0.086			

Hospitalization Data

Hospitalizations	28 Day Completers	Observed Hosp. Rate	Est. Hosp. Rate (95% interval)
18	573	0.0314	0.0295 (0.0181, 0.044)
5	207	0.0242	0.0288 (0.0108, 0.0568)
11	405	0.0272	0.0252 (0.0134, 0.0417)
7	263	0.0266	0.0241 (0.0103, 0.0436)
41	1448	0.0283	

Intervention Status
Enrolling
Dropped
Enrolling
Enrolling

Observed Patient Recoveries

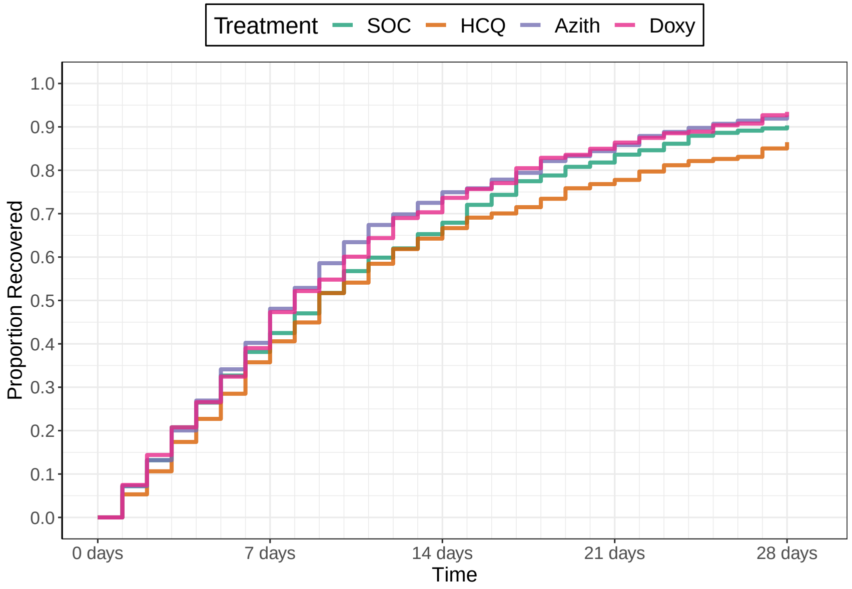

Number Un−recovered

	0 days	7 days	14 days	21 days	28 days
SOC	638	384	211	110	60
HCQ	207	133	74	48	31
Azith	470	274	124	67	33
Doxy	334	191	89	42	19

Recovery Inferences

	Pr(Superiority)	Pr(Meaningful Effect)	Pr(Best)	Randomization Probability
SOC				0.33
HCQ	0.064	0	0	0
Azith	0.9527	0.0717	0.4225	0.34
Doxy	0.9047	0.0707	0.3275	0.32

Hospitalization Inferences

Pr(Superiority)	Pr(Meaningful Effect)
0.5562	0.06
0.6805	0.0505
0.7072	0.0848

Hazard Ratio Estimates

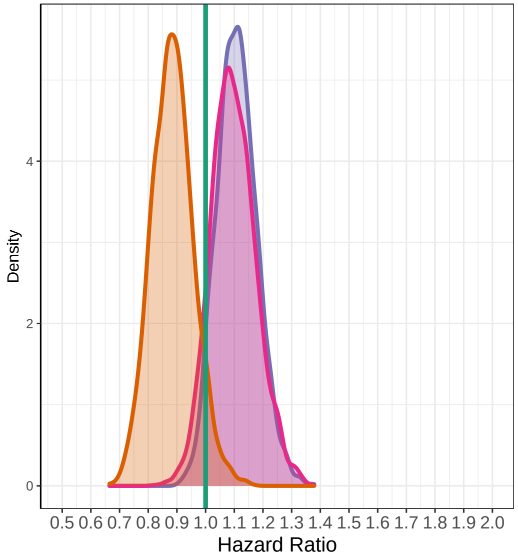

Median Time To Recovery Estimates

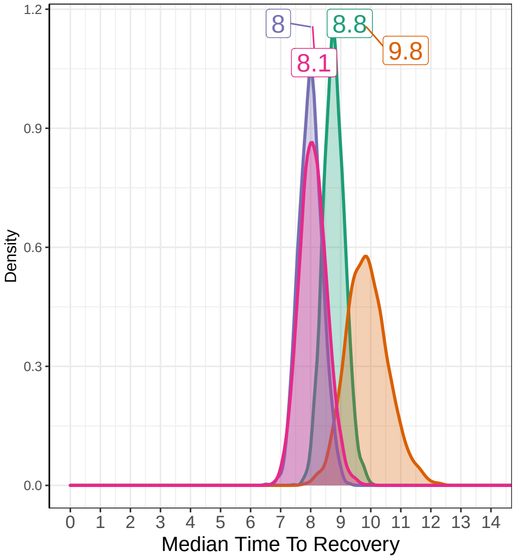

Interim 4 on January 07, 2021

Recovery Data

	Enrolled	Complete	Recovered	Exposure Days	Recoveries Per Day	Estimated Hazard	Estimated HR (95% interval)	Estimated Median Time to Recovery
SOC	709	684	620	7506	0.083	0.08	1	8.89
HCQ	207	207	179	2453	0.073	0.071	0.886 (0.759, 1.029)	9.92
Azith	553	516	484	5173	0.094	0.09	1.118 (1, 1.254)	8.04
Doxy	388	367	347	3635	0.095	0.091	1.138 (1.003, 1.285)	7.92
Total	1857	1774	1630	18768	0.087			

Hospitalization Data

Hospitalizations	28 Day Completers	Observed Hosp. Rate	Est. Hosp. Rate (95% interval)
18	638	0.0282	0.0267 (0.0163, 0.0397)
5	207	0.0242	0.0289 (0.0106, 0.0564)
12	470	0.0255	0.0235 (0.0125, 0.0373)
10	334	0.0299	0.0283 (0.0143, 0.0466)
45	1649	0.0273	

Intervention Status
Enrolling
Dropped
Enrolling
Enrolling

Observed Patient Recoveries

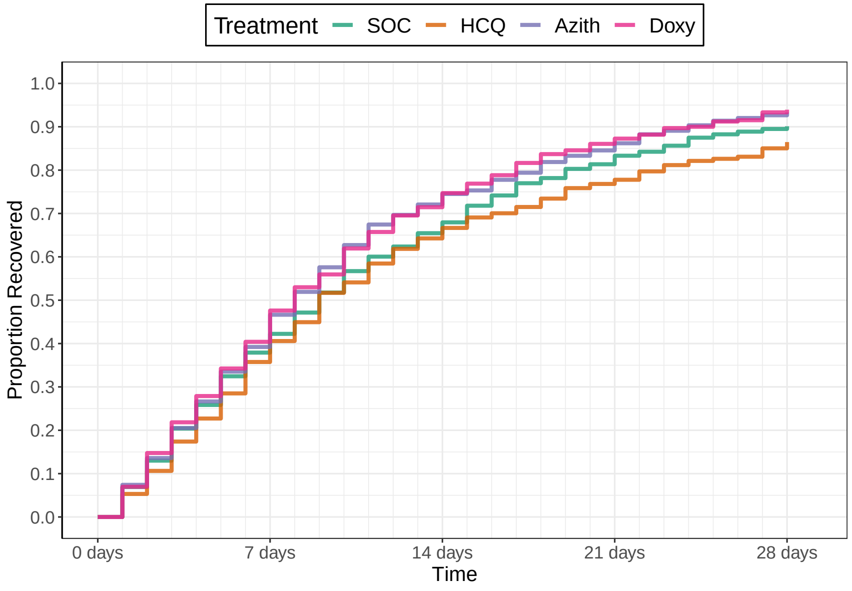

Number Un−recovered

	0 days	7 days	14 days	21 days	28 days
SOC	709	431	235	123	68
HCQ	207	133	74	48	31
Azith	553	319	138	75	35
Doxy	388	223	105	46	22

Recovery Inferences

	Pr(Superiority)	Pr(Meaningful Effect)	Pr(Best)	Randomization Probability
SOC				0.33
HCQ	0.0633	0	0	0
Azith	0.9743	0.0653	0.2918	0.33
Doxy	0.978	0.128	0.4582	0.33

Hospitalization Inferences

Pr(Superiority)	Pr(Meaningful Effect)
0.4572	0.0332
0.643	0.0288
0.461	0.0102

Hazard Ratio Estimates

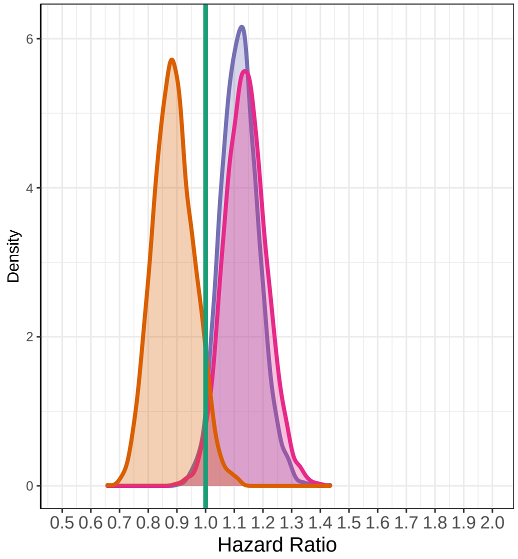

Median Time To Recovery Estimates

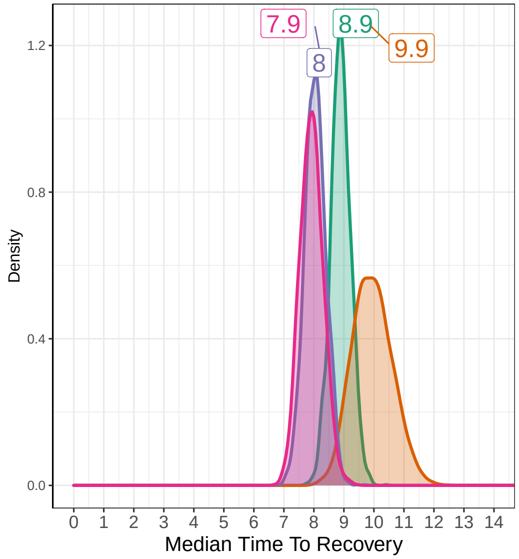

Interim 5 on February 04, 2021

Recovery Data

	Enrolled	Complete	Recovered	Exposure Days	Recoveries Per Day	Estimated Hazard	Estimated HR (95% interval)	Estimated Median Time to Recovery
SOC	770	742	672	8161	0.082	0.081	1	8.97
HCQ	207	207	179	2453	0.073	0.072	0.892 (0.762, 1.037)	9.94
Azith	631	597	561	6070	0.092	0.09	1.113 (0.996, 1.241)	8.14
Doxy	445	426	403	4196	0.096	0.093	1.154 (1.018, 1.302)	7.89
Total	2053	1972	1815	20881	0.087			

Hospitalization Data

Hospitalizations	28 Day Completers	Observed Hosp. Rate	Est. Hosp. Rate (95% interval)
18	709	0.0254	0.0245 (0.0146, 0.0368)
5	207	0.0242	0.0289 (0.0106, 0.0552)
14	553	0.0253	0.0238 (0.0135, 0.0369)
11	388	0.0284	0.0269 (0.0138, 0.0433)
48	1857	0.0258	

Intervention Status
Enrolling
Dropped
Enrolling
Enrolling

Observed Patient Recoveries

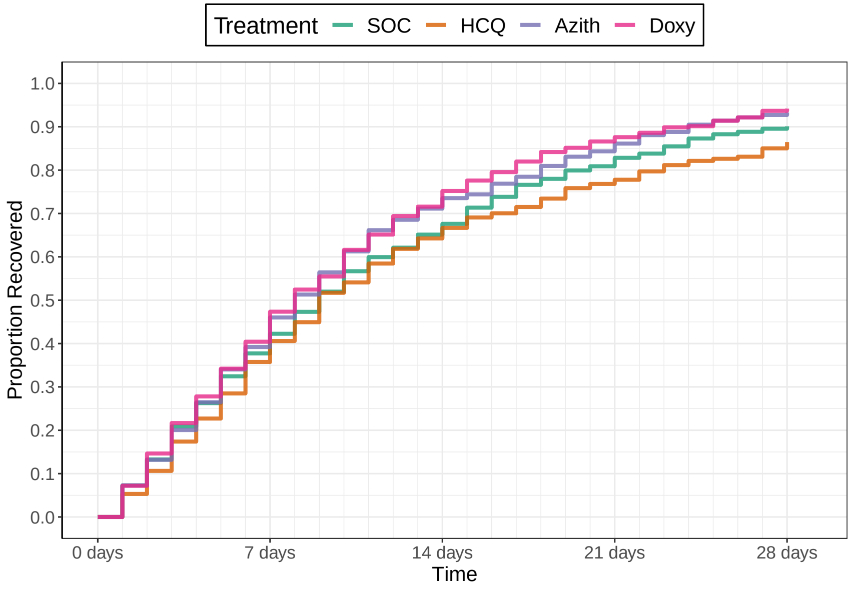

Number Un−recovered

	0 days	7 days	14 days	21 days	28 days
SOC	770	469	253	138	74
HCQ	207	133	74	48	31
Azith	631	374	167	88	39
Doxy	445	258	118	54	25

Recovery Inferences

	Pr(Superiority)	Pr(Meaningful Effect)	Pr(Best)	Randomization Probability
SOC				0.33
HCQ	0.069	0	0	0
Azith	0.9693	0.0623	0.2185	0.33
Doxy	0.9857	0.1877	0.5315	0.34

Hospitalization Inferences

Pr(Superiority)	Pr(Meaningful Effect)
0.3985	0.0142
0.5432	0.0082
0.4128	0.0068

Hazard Ratio Estimates

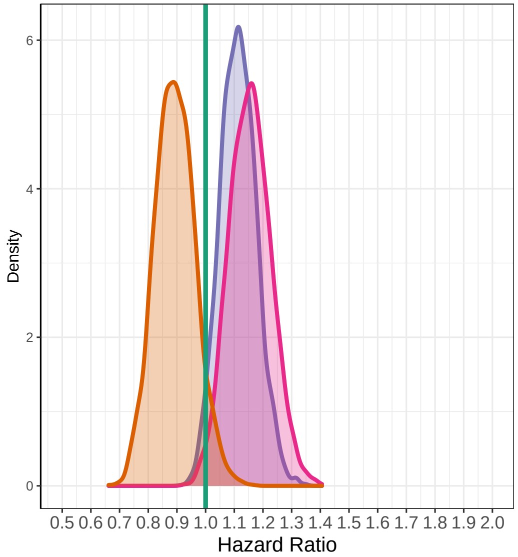

Median Time To Recovery Estimates

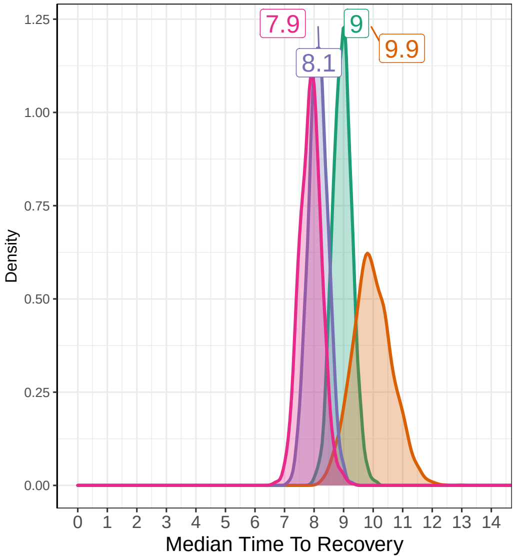

Interim 6 on March 04, 2021

Recovery Data

	Enrolled	Complete	Recovered	Exposure Days	Recoveries Per Day	Estimated Hazard	Estimated HR (95% interval)	Estimated Median Time to Recovery
SOC	847	807	733	8965	0.082	0.08	1	9.02
HCQ	207	207	179	2453	0.073	0.072	0.895 (0.764, 1.043)	9.97
Azith	695	673	629	6919	0.091	0.089	1.102 (0.995, 1.216)	8.26
Doxy	506	482	458	4790	0.096	0.093	1.156 (1.037, 1.296)	7.91
Total	2255	2169	1999	23127	0.086			

Hospitalization Data

Hospitalizations	28 Day Completers	Observed Hosp. Rate	Est. Hosp. Rate (95% interval)
19	770	0.0247	0.0236 (0.0145, 0.0346)
5	207	0.0242	0.0287 (0.0106, 0.0557)
16	631	0.0254	0.0245 (0.0145, 0.0368)
14	445	0.0315	0.0296 (0.0168, 0.0463)
54	2053	0.0263	

Intervention Status
LostOnTTR
Dropped
Futility
SuccessOnTTRFutilityOnHosp Not Announced

Observed Patient Recoveries

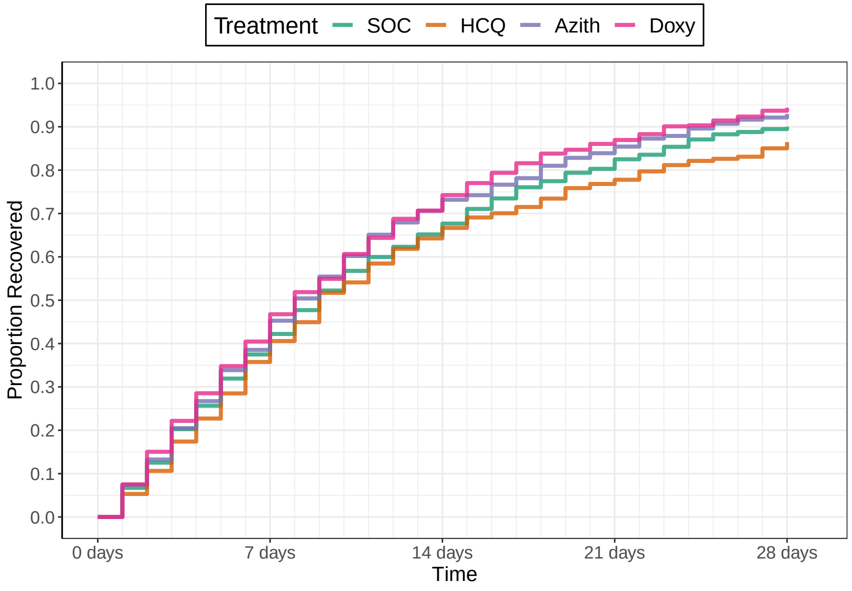

Number Un−recovered

	0 days	7 days	14 days	21 days	28 days
SOC	847	516	279	152	78
HCQ	207	133	74	48	31
Azith	695	420	196	105	49
Doxy	506	293	139	62	28

Recovery Inferences

	Pr(Superiority)	Pr(Meaningful Effect)	Pr(Best)	Randomization Probability
SOC				0.5
HCQ	0.0757	0	0	0
Azith	0.9667	0.0317	0	0
Doxy	0.9933	0.1847	0.75	0.5

Hospitalization Inferences

Pr(Superiority)	Pr(Meaningful Effect)
0.369	0.0078
0.464	0.0028
0.2502	0.0022

Hazard Ratio Estimates

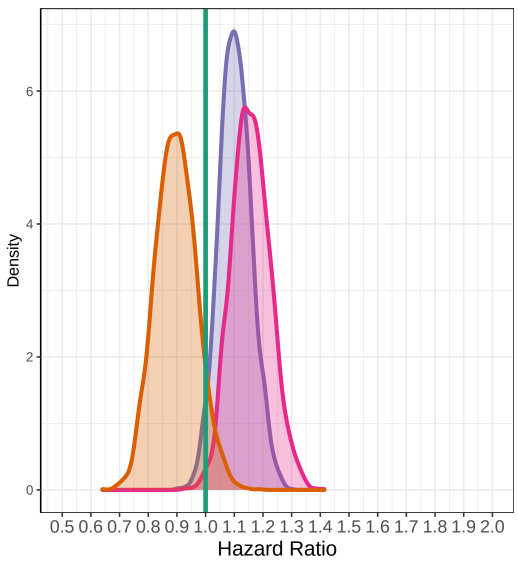

Median Time To Recovery Estimates

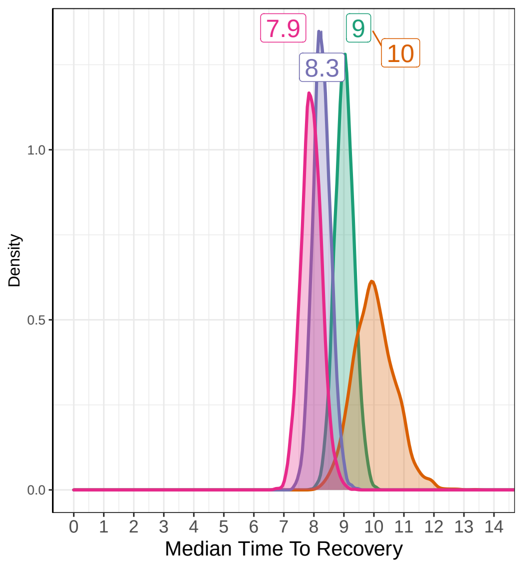

Interim 7 on August 27, 2022

Recovery Data

	Enrolled	Complete	Recovered	Exposure Days	Recoveries Per Day	Estimated Hazard	Estimated HR (95% interval)	Estimated Median Time to Recovery
SOC	2688	2688	2373	30880	0.077	0.074	1	9.39
HCQ	207	207	179	2453	0.073	0.07	0.948 (0.82, 1.088)	9.86
Azith	695	695	646	7084	0.091	0.087	1.17 (1.074, 1.275)	8.1
Doxy	2410	2410	2264	22450	0.101	0.096	1.293 (1.22, 1.368)	7.37
Total	6000	6000	5462	62867	0.087			

Hospitalization Data

Hospitalizations	28 Day Completers	Observed Hosp. Rate	Est. Hosp. Rate (95% interval)
88	2688	0.0327	0.0331 (0.0266, 0.0403)
5	207	0.0242	0.0284 (0.0105, 0.0557)
16	695	0.0230	0.0243 (0.0143, 0.037)
68	2410	0.0282	0.0286 (0.0224, 0.0355)
177	6000	0.0295	

Intervention Status
Complete
Dropped
Dropped
SuccessOnTTRFutilityOnHosp *Announced*

Observed Patient Recoveries

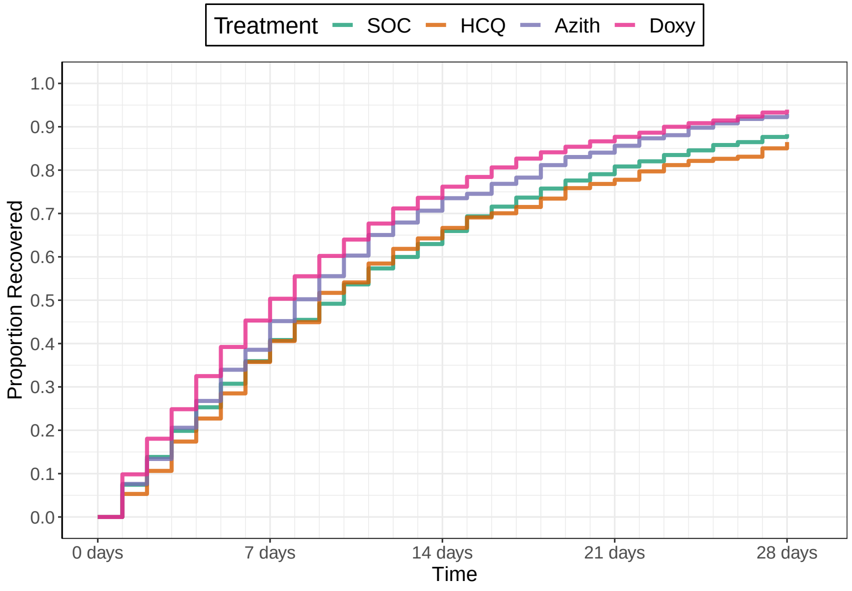

Number Un−recovered

	0 days	7 days	14 days	21 days	28 days
SOC	2688	1722	996	563	332
HCQ	207	133	74	48	31
Azith	695	427	204	111	54
Doxy	2410	1318	636	322	162

Recovery Inferences

	Pr(Superiority)	Pr(Meaningful Effect)	Pr(Best)	Randomization Probability
SOC				
HCQ	0.2353	3e−04	0	
Azith	1	0.271	0	
Doxy	1	0.9847	0.75	

Hospitalization Inferences

Pr(Superiority)	Pr(Meaningful Effect)
0.6968	0.0802
0.9035	0.0392
0.8315	8e−04

Hazard Ratio Estimates

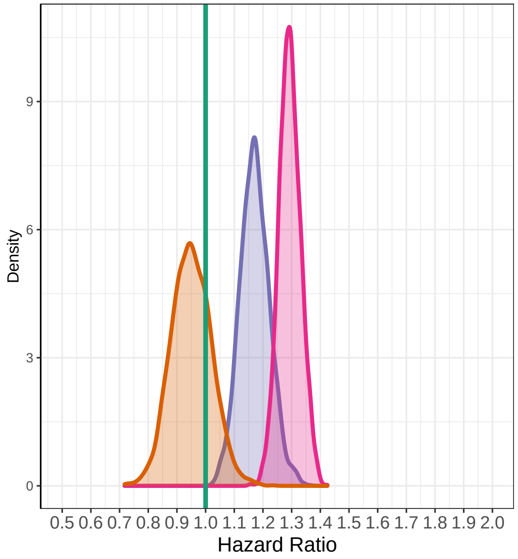

Median Time To Recovery Estimates

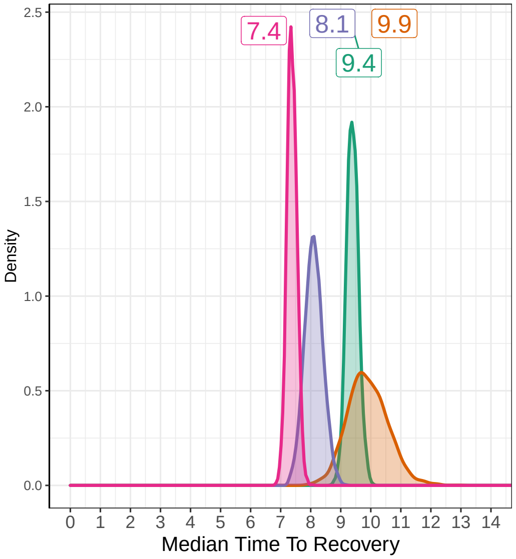

6.6 Computational Details

In the simulations, relevant covariates (age, comorbidity, vaccination status) are regarded as nuisance variables, and hence are not factored into the simulations. For computational efficiency, the primary analysis for the hospitalization endpoint was simulated using beta-binomial posterior distributions; however logistic regression will be used during execution to account for the covariates (as specified in Section 3.2). Operating characteristics are based on a minimum of 1,000 simulations per scenario. Each Bayesian MCMC fit includes at least 4,000 posterior samples with a burn-in of at least 1,000 samples. In trial execution, at least 10,000 MCMC samples will be taken from the Bayesian posterior distribution after an appropriate burn-in. The R software package was used to summarize the simulation output and to create graphics and tables for this report. This document was typeset with LaTeX.

Figure S1 Estimated mean and 95% confidence interval of daily rating of feeling well over 28 days by treatment arm (concurrent randomisation and eligible population)

(a) SARS-CoV-2 positive participants

(b) Participants irrespective of SARS-CoV-2 status

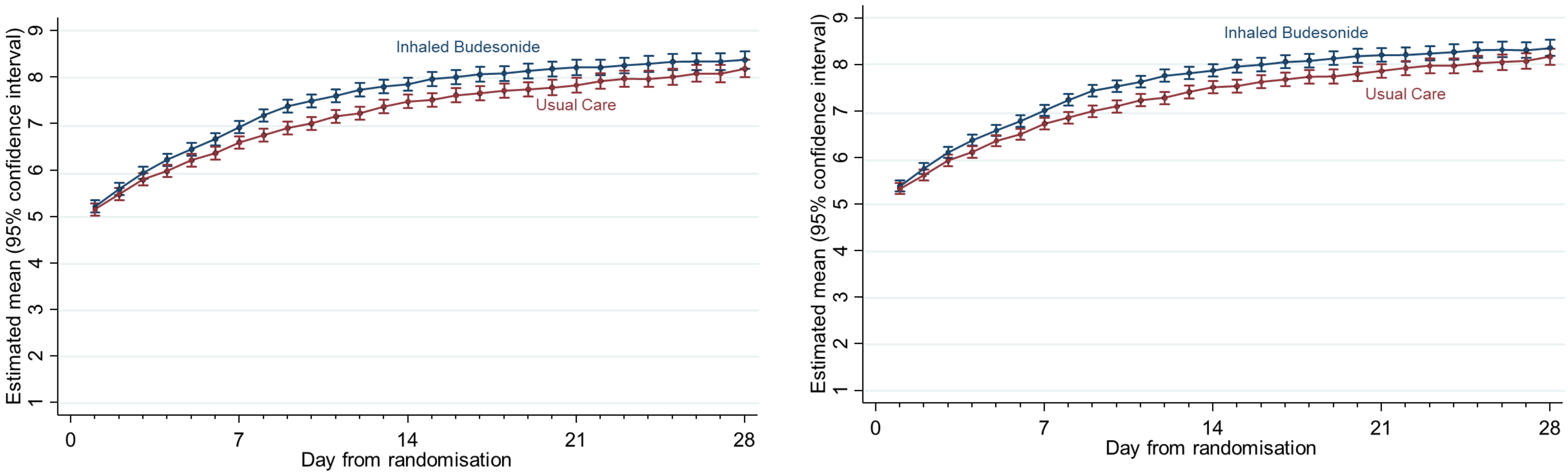

Figure S2 Summary and results of the time to sustained recovery (concurrent randomisation and eligible population)

(a) SARS-CoV-2 positive participants

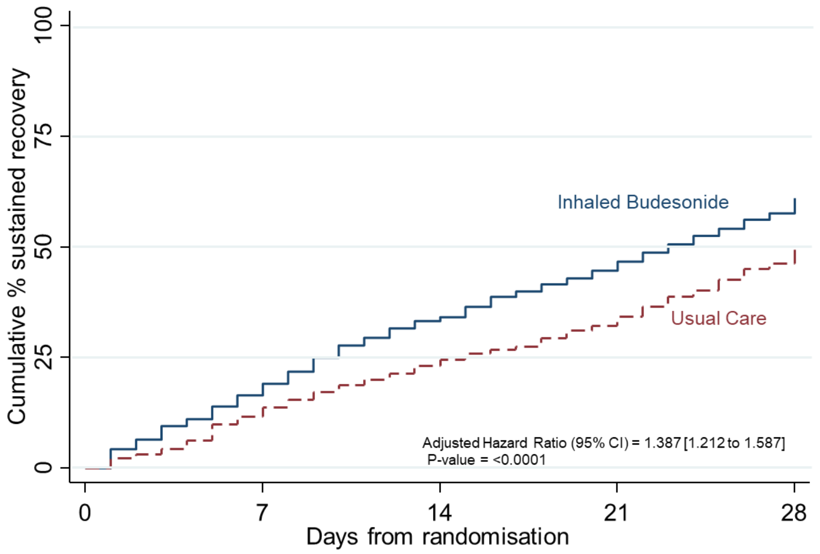

Cumulative number not yet recovered (recovered)

Inhaled Budesonide	787 (0)	644 (149)	499 (263)	409 (356)	312 (462)
Usual Care	799 (0)	696 (109)	598 (192)	516 (267)	404 (390)

(b) Irrespective of SARS-CoV-2 status

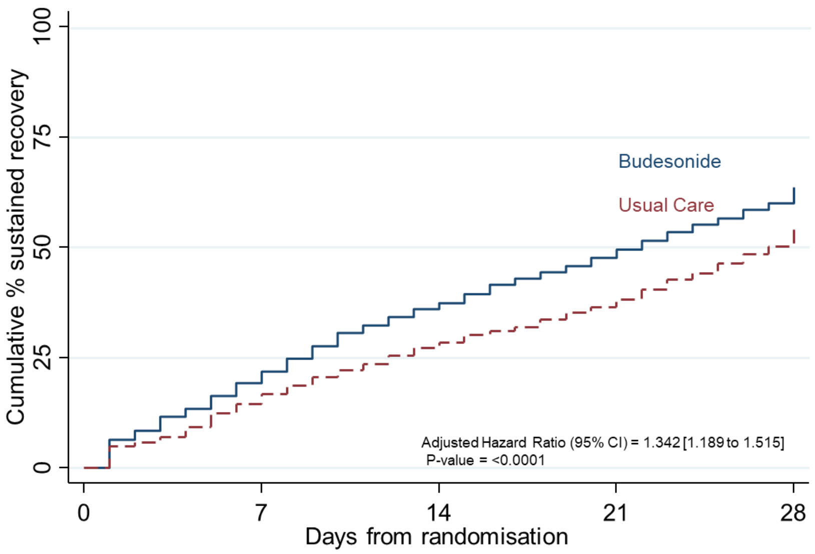

Cumulative number not yet recovered (recovered)

Inhaled Budesonide	989 (0)	783 (214)	602 (362)	487 (475)	369 (606)
Usual Care	986 (0)	831 (165)	696 (278)	596 (370)	462 (517)

Figure S3 Secondary Time-to-Event Outcomes (the concurrent randomisation and eligible SARS-CoV-2 positive population)

a) Time to alleviation of all symptoms, and each symptom

Time to Alleviation of Symptom[1]	Inhaled Budesonide	Usual Care	Adjusted HR (95% CI)	P-value
All symptoms	630/701	666/732	1·07 (0·96, 1·19)	0·25
Fever	260/278	263/282	1·13 (0·95, 1·35)	0·16
Cough	404/441	440/468	1·04 (0·91, 1·19)	0·55
Shortness of breath	293/328	291/330	1·14 (0·97, 1·34)	0·12
Muscle ache	415/444	445/473	1·16 (1·02, 1·33)	0·027
Nausea-vomiting	177/193	183/204	1·29 (1·05, 1·59)	0·017
Diarrhoea	176/188	180/199	1·28 (1·04, 1·58)	0·021
Generally unwell	566/608	607/648	1·08 (0·96, 1·21)	0·19

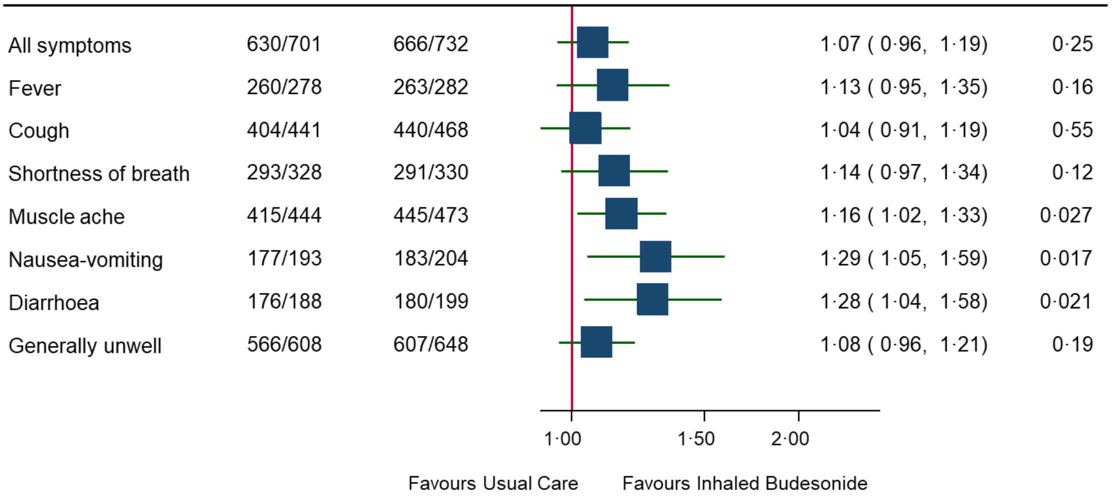

[1]Time to alleviation of symptoms is defined as the time from randomisation to all symptoms or each symptom being rated as mild or none

(b) Time to sustained alleviation of all symptoms, each symptom

Time to Sustained Alleviation[1]	Inhaled Budesonide	Usual Care	Adjusted HR (95% CI)	P-value
All symptoms	579/701	597/731	1·13 (1·01, 1·27)	0·037
Fever	254/278	256/281	1·12 (0·94, 1·33)	0·22
Cough	388/441	414/467	1·12 (0·97, 1·29)	0·11
Shortness of breath	267/328	256/330	1·13 (0·95, 1·34)	0·17
Muscle ache	387/444	407/472	1·27 (1·10, 1·46)	0·001
Nausea-vomiting	175/193	181/204	1·37 (1·11, 1·69)	0·003
Diarrhoea	172/188	173/199	1·30 (1·05, 1·61)	0·014
Generally unwell	530/608	560/647	1·13 (1·00, 1·27)	0·042

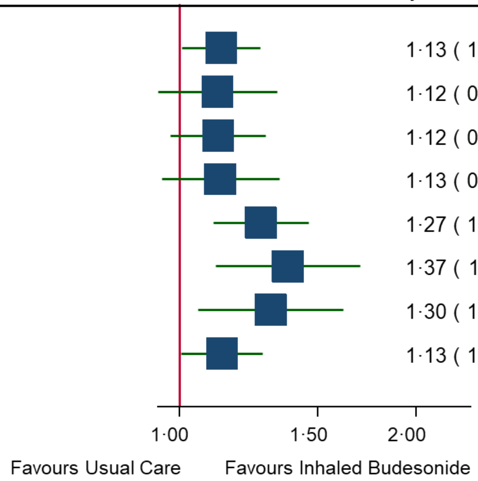

[1]Time to sustained alleviation of symptoms is defined as time to alleviation of symptoms with no subsequent symptom being rated as mild or none

(c) Time to initial reduction of severity of all symptoms, and each symptom

Time to Initial Reduction of Severity of Symptom[1]	Inhaled Budesonide	Usual Care	Adjusted HR (95% CI)	P-value
All symptoms	662/786	650/797	1·19 (1·07, 1·32)	0·0019
Fever	382/401	402/424	1·14 (0·99, 1·32)	0·061
Cough	605/656	618/683	1·13 (1·01, 1·26)	0·035
Shortness of breath	358/409	351/415	1·17 (1·01, 1·35)	0·041
Muscle ache	553/583	580/624	1·24 (1·10, 1·39)	<0·0001
Nausea-vomiting	238/256	252/272	1·17 (0·98, 1·40)	0·089
Diarrhoea	198/213	195/215	1·13 (0·92, 1·38)	0·24
Generally unwell	709/761	712/772	1·12 (1·01, 1·25)	0·027

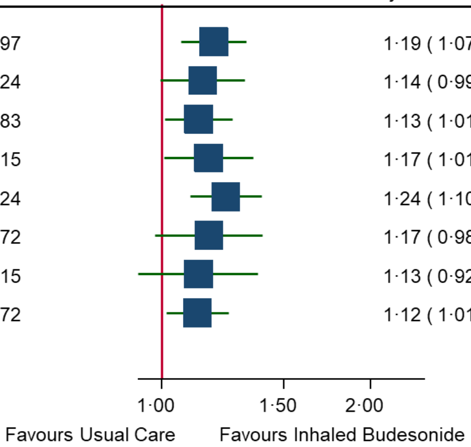

[1]Time to initial reduction of severity of symptoms is defined as time to reduction in severity of each individual symptoms to at least one grade lower

Figure S4 Secondary Time-to-Event Outcomes (the concurrent randomisation and eligible population, irrespective of SARS-CoV-2 status)

a) Time to alleviations of all symptoms, and each symptom

Time to Alleviation of Symptom[1]	Inhaled Budesonide	Usual Care	Adjusted HR (95% CI)	P-value
All symptoms	795/875	811/889	1·08 (0·97, 1·19)	0·14
Fever	320/343	313/333	1·14 (0·97, 1·34)	0·12
Cough	510/553	532/566	1·04 (0·92, 1·18)	0·54
Shortness of breath	350/390	341/385	1·15 (0·99, 1·34)	0·071
Muscle ache	493/530	542/574	1·14 (1·00, 1·29)	0·046
Nausea-vomiting	198/219	204/227	1·26 (1·03, 1·55)	0·024
Diarrhoea	208/225	215/236	1·22 (1·00, 1·49)	0·047
Generally unwell	680/730	716/760	1·06 (0·95, 1·18)	0·30

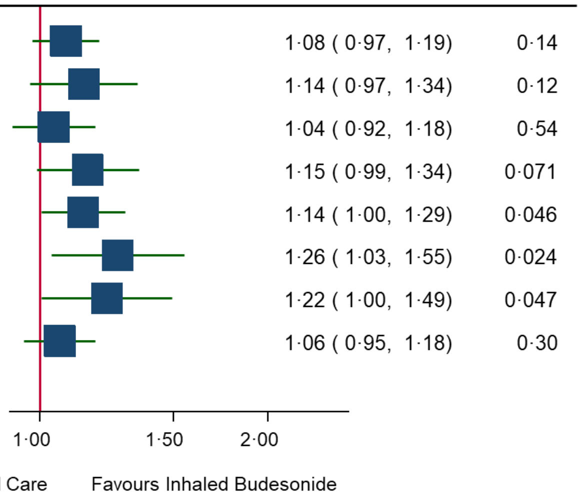

[1]Time to alleviation of symptoms is defined as the time from randomisation to all symptoms or each symptom being rated as mild or none

(b) Time to sustained alleviation of all symptoms, and each symptom

Time to Sustained Alleviation[1]	Inhaled Budesonide	Usual Care	Adjusted HR (95% CI)	P-value
All symptoms	727/874	730/887	1·12 (1·01, 1·24)	0·038
Fever	314/343	303/332	1·14 (0·97, 1·35)	0·11
Cough	486/553	500/565	1·10 (0·97, 1·25)	0·14
Shortness of breath	316/390	299/385	1·17 (1·00, 1·38)	0·055
Muscle ache	460/529	497/573	1·23 (1·08, 1·41)	0·002
Nausea-vomiting	196/219	202/227	1·33 (1·09, 1·63)	0·006
Diarrhoea	203/224	206/236	1·28 (1·05, 1·57)	0·015
Generally unwell	635/729	655/759	1·13 (1·01, 1·26)	0·039

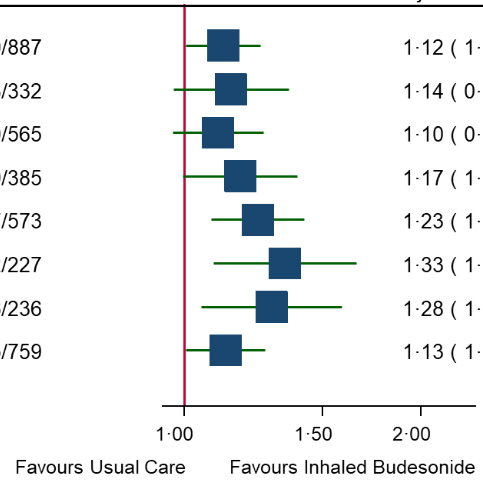

[1]Time to sustained alleviation of symptoms is defined as time to alleviation of symptoms with no subsequent symptom being rated as mild or none

(c) Time to initial reduction of severity of all symptoms, and each symptom

Time to Initial Reduction of Severity of Symptom[1]	Inhaled Budesonide	Usual Care	Adjusted HR (95% CI)	P-value
All symptoms	840/985	820/985	1.14 (1.03, 1.26)	0.010
Fever	487/511	511/535	1.11 (0.98, 1.27)	0.10
Cough	755/818	771/846	1.09 (0.98, 1.21)	0.10
Shortness of breath	452/515	445/516	1.09 (0.95, 1.25)	0.23
Muscle ache	679/717	700/751	1.22 (1.09, 1.36)	<0.0001
Nausea-vomiting	283/306	292/312	1.14 (0.96, 1.35)	0.14
Diarrhoea	242/262	235/255	1.09 (0.90, 1.31)	0.37
Generally unwell	874/934	872/939	1.11 (1.01, 1.23)	0.031

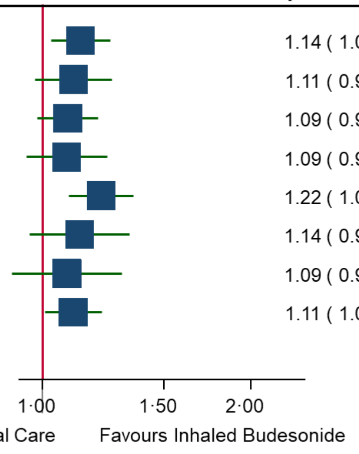

[1]Time to initial reduction of severity of symptoms is defined as time to reduction in severity of each individual symptoms to at least one grade lower

Table S1 Baseline characteristics for participants irrespective of SARS-CoV-2 status by treatment group population

	Inhaled Budesonide (N=1047)	Usual Care* (N=1959)	Overall (N=3006)
**Age category, n(%)**			
50-64	431 (41)	1037 (53)	1468 (49)
65 and over	616 (59)	922 (47)	1538 (51)
**Sex, n(%)**			
Female	542 (52)	1064 (54)	1606 (53)
Male	505 (48)	895 (46)	1400 (47)
**Ethnicity†, n(%)**			
White	966 (92)	1722 (88)	2688 (89)
Mixed background	12 (1)	29 (2)	41 (1)
South Asian	55 (5)	89 (5)	144 (5)
Black	6 (<1)	8 (<1)	14 (1)
Other	8 (<1)	22 (1)	30 (1)
Missing, n(%)	0 (<1)	89 (5)	89 (3)
**Index of multiple deprivation quintile, n(%)**			
(Most Deprived) 1	181 (17)	343 (18)	524 (17)
2	195 (19)	325 (17)	521 (17)
3	202 (19)	399 (20)	601 (20)
4	227 (22)	427 (22)	654 (22)
(Least Deprived) 5	237 (23)	464 (24)	701 (23)
Missing, n(%)	4 (<1)	1 (<1)	5 (<1)
**Duration of illness prior to randomisation, median(IQR)**	6·0 (4·0 to 9·0)	6·0 (4·0 to 9·0)	6·0 (4·0 to 9·0)
**Smoking status, n(%)**			
Current smoker	69 (7)	194 (9.9%)	263 (9)
Former smoker	427 (41)	755 (38.5%)	1182 (39)
Never smoker	542 (52)	971 (49.6%)	1513 (50)
Missing, n(%)	9 (<1)	39 (2.0%)	48 (2)
**Swab result, n(%)**			
Negative	135 (13)	557 (28.4%)	692 (23)
Positive	833 (80)	1126 (57.5%)	1959 (65)
No result	9 (1)	9 (0.5%)	18 (1)
Missing, n(%)	70 (7)	267 (13.6%)	337 (11)
**Received vaccination, n(%)**	121 (12)	116 (5.9%)	237 (8)
**Vaccine doses received, n(%)**			
One dose	113 (11)	106 (5)	219 (7)
Two doses	8 (1)	10 (1)	18 (1)
**Comorbidity, n(%)**	850 (81)	1655 (85)	2505 (8)
**Number of comorbidities, median (IQR)**	1 (1 to 2)	1 (1 to 2)	1 (1 to 2)
**Comorbidities**			
**Asthma, COPD or lung disease, n(%)**	89 (9)	465 (24)	554 (18)
**Diabetes, n(%)**	208 (20)	412 (21)	620 (21)
**Heart problems‡, n(%)**	176 (17)	289 (15)	465 (16)
**High blood pressure required medication, n(%)**	476 (46)	848 (43)	1324 (44)
**Liver disease, n(%)**	23 (2)	54 (3)	77 (3)
**Stroke or other neurological problem, n(%)**	72 (7)	113 (6)	185 (6)
**Taking angiotensin-converting enzyme inhibitor§, n(%)**	249 (24)	417 (21)	666 (22)
Missing, n(%)	4 (<1)	9 (1)	13 (<1)
**Fever, n(%)**			

	Inhaled Budesonide (N=1047)	Usual Care* (N=1959)	Overall (N=3006)
No problem	512 (49)	868 (44)	1380 (46)
Mild problem	315 (30)	677 (35)	992 (33)
Moderate problem	193 (18)	354 (18)	547 (18)
Major problem	27 (3)	60 (3)	87 (3)
**Cough, n(%)**			
No problem	182 (17)	320 (16)	502 (17)
Mild problem	460 (44)	815 (42)	1275 (42)
Moderate problem	328 (31)	699 (36)	1027 (34)
Major problem	77 (7)	125 (6)	202 (7)
**Shortness of breath, n(%)**			
No problem	509 (49)	793 (41)	1302 (43)
Mild problem	372 (36)	775 (40)	1147 (38)
Moderate problem	142 (14)	347 (18)	489 (16)
Major problem	24 (2)	44 (2)	68 (2)
**Muscle ache, n(%)**			
No problem	288 (28)	537 (27)	825 (27)
Mild problem	346 (33)	730 (37)	1076 (36)
Moderate problem	297 (28)	519 (27)	816 (27)
Major problem	116 (11)	173 (9)	289 (10)
**Nausea/vomiting, n(%)**			
No problem	733 (70)	1404 (72)	2137 (71)
Mild problem	202 (19)	411 (21)	613 (20)
Moderate problem	88 (8)	113 (6)	201 (7)
Major problem	24 (2)	31 (2)	55 (2)
**Feeling generally unwell, n(%)**			
No problem	59 (6)	97 (5)	156 (5)
Mild problem	384 (37)	675 (35)	1059 (35)
Moderate problem	448 (43)	737 (38)	1185 (39)
Major problem	156 (15)	224 (11)	380 (13)
Missing, n(%)	0 (0)	226 (12)	226 (8)
**Diarrhea, n(%)**			
No problem	778 (74)	1293 (66)	2071 (69)
Mild problem	174 (17)	302 (15)	476 (16)
Moderate problem	74 (7)	104 (5)	178 (6)
Major problem	21 (2)	34 (2)	55 (2)
Missing, n(%)	0 (0)	226 (12)	226 (8)
**Taken antibiotics since illness started, n(%)**	68 (7)	119 (6)	187 (6)
Missing, n(%)	0 (0)	1 (<1)	1 (0)
**Use of Healthcare services**			
**General Practitioner, n(%)**	237 (23)	496 (25)	733 (24)
**Other primary care services, n(%)**	100 (10)	152 (8)	252 (8)
**NHS 111, n(%)**	113 (11)	281 (14)	394 (13)
**A&E, n(%)**	18 (2)	29 (2)	47 (2)
**Other healthcare services, n(%)**	27 (3)	42 (2)	69 (2)
**Well-being (WHO5 Questionnaire)¶, mean(SD)**	47.2 (25·3)	47.7 (25·3)	47.6 (25.3)
Missing, n(%)	0 (0)	24 (<1)	24 (<1)

* Includes participants randomised before the inhaled budesonide arm was open.
† Data on ethnicity were collected retrospectively via notes review before July 2020
‡ E.g. angina, heart attack, heart failure, atrial fibrillation, valve problems
§ Such as Ramipril, Lisinopril, Perindopril, Captopril or Enalapril
¶ Well-being is measured using the WHO well-being index which includes 5 items relating to well-being measured on a five point scale. A total score is computed by summing the scores to the five individual questions to give a raw score ranging from 0 to 25 which is then multiplied by 4 to give the final score from 0 representing the worst imaginable well-being to 100 representing the best imaginable well-being.

Table S2 Secondary Outcomes (concurrent randomisation and eligible population irrespective of SARS-CoV-2 status)

Secondary outcomes	Inhaled Budesonide	Usual Care	Estimated treatment effect (95% CI)	P-value
Early sustained recovery, n/N (%)	345/983 (35)	256/982 (26)	1·39 (1·21 to 1·60)*	<0·0001
Sustained recovery, n/N (%)	606/990 (61)	517/986 (52)		
Time to sustained recovery (days), median (IQR)	22 (9 to -)	27 (12 to -)	1·34 (1·19 to 1·52)†	<0·0001
Alleviation of all symptoms, n/N (%)	795/875 (91)	811/889 (91)		
Time to alleviations of all symptoms (days), median (IQR)	4 (2 to 8)	4 (2 to 9)	1·08 (0·98 to 1·19) †	0·14
Sustained alleviation of all symptoms, n/N (%)	727/874 (83)	730/887 (82)		
Time to sustained alleviation of all symptoms (days), median (IQR)	8 (3 to 23)	12 (4 to 25)	1·12 (1·01 to 1·25)†	0·04
Initial reduction of severity of symptoms, n/N (%)	840/985 (85)	820/985 (83)		
Time to initial reduction of severity of symptoms (days), median (IQR)	7 (3 to 14)	7 (3 to 18)	1·14 (1·03 to 1·26)†	0·01
Rating of how well participant feels (1 worst, 10 best), mean (SD) [n]				
Day 7	7·0 (1·8) [944]	6·8 (1·9) [949]	0·29 (0·12 to 0·46)‡	0·001
Day 14	7·9 (1·7) [943]	7·5 (1·7) [951]	0·36 (0·18 to 0·55)‡	<0·0001
Day 21	8·3 (1·5) [796]	7·9 (1·6) [780]	0·35 (0·13 to 0·56)‡	0·002
Day 28	8·3 (1·5) [958]	8·2 (1·6) [957]	0·19 (-0·05 to 0·43)‡	0·12
Well-being (WHO5 Questionnaire), mean (SD)[n]				
Day 14	43·8 (24·6) [900]	40·4 (24·3) [901]	3·38 (1·30 to 5·47)‡	0·001
Day 28	54·3 (24·7) [898]	51·7 (24·5) [899]	2·52 (0·43 to 4·61)‡	0·02
Self-reported contact with ≥1 healthcare service, n/N (%)	502/979 (51)	546/975 (56)	0·88 (0·81 to 0·96)*	0·003
GP reported contact with ≥1 healthcare service, n/N (%)	381/753 (51)	422/759 (56)	0·88 (0·79 to 0·97)*	0·01
New infections in household, n/N (%)	232/971 (24)	244/970 (25)	0·94 (0·80 to 1·10)*	0·46
Prescription of antibiotics, n/N (%)	52/694 (8)	64/684 (9)	0·80 (0·56 to 1·14)*	0·24
Hospital assessment without admission, n/N (%)	28/989 (3)	24/985 (2)	1·16 (0·68 to 1·99)*	0·67
Oxygen Administration, n/N (%)	55/975 (6)	76/973 (8)	0·72 (0·52 to 1·01)*	0·06
Mechanical ventilation, n/N (%)	14/977 (1)	14/972 (1)	0·99 (0·48 to 2·08)§	>0·99
ICU admission, n/N (%)	12/972 (1)	21/966 (2)	0·57 (0·28 to 1·15)§	0·12
**WHO Ordinal Scale of Clinical Progression, n/N (%)**				
Not hospitalised	910 (92)	883 (90)	0·71 [0·51 to 0·97]¶	0·03
Hospitalised without need for supplemental oxygen	19 (2)	24 (2)		
Hospitalised with need for supplemental oxygen	38 (4)	59 (6)		
Hospitalised with need for non-invasive positive pressure ventilation or high-flow nasal cannula	0 (0)	1 (<1)		

Secondary outcomes	Inhaled Budesonide	Usual Care	Estimated treatment effect (95% CI)	P-value
Hospitalised with need for mechanical ventilation or extracorporeal membrane oxygenation	14 (1)	10 (1)		
Death	9 (1)	10 (1)		

* Relative risks adjusted for age, comorbidity at baseline, duration of illness, and vaccination status at baseline.

† Estimated hazard ratio derived from a Cox proportional hazard model adjusted for age, comorbidity at baseline, duration of illness, and vaccination status at baseline, with 95% confidence interval.

‡ Mixed effect model adjusting age, comorbidity, duration of illness, vaccination status at baseline, and time. Participant was fitted as a random effect. WHO well-being score was also adjusted for the score at baseline.

§ Unadjusted relative risks due to low event rate.

¶ Proportional odds ratio derived from ordinal logistic regression adjusted for age, comorbidity at baseline, duration of illness, and vaccination status at baseline.

Table S3: Reasons for hospitalisation unrelated to COVID-19

**Description**	**Inhaled Budesonide**	**Usual Care**
Lower limb fracture	1	
Alcohol induced pancreatitis	1	
Cholelithiasis		1
Atrial fibrillation		1
Heart valve surgery		1
Appendicitis		1
